# *In silico* identification of potential inhibitors targeting *Streptococcus mutans* sortase A

**DOI:** 10.1038/ijos.2016.58

**Published:** 2017-03-30

**Authors:** Hao Luo, Dan-Feng Liang, Min-Yue Bao, Rong Sun, Yuan-Yuan Li, Jian-Zong Li, Xin Wang, Kai-Min Lu, Jin-Ku Bao

**Affiliations:** 1 School of Life Sciences and Key Laboratory of Ministry of Education for Bio-Resources and Bio-Environment, Sichuan University, Chengdu, China; 2 State Key Laboratory of Biotherapy/Collaborative Innovation Centre for Biotherapy, West China Hospital, Sichuan University, Chengdu, China; 3 State Key Laboratory of Oral Diseases, National Clinical Research Center for Oral Diseases, West China Hospital of Stomatology, Sichuan University, Chengdu, China

**Keywords:** dental caries, molecular dynamics simulation, molecular docking, potential inhibitors, sortase A, *Streptococcus mutans*

## Abstract

Dental caries is one of the most common chronic diseases and is caused by acid fermentation of bacteria adhered to the teeth. *Streptococcus mutans* (*S. mutans*) utilizes sortase A (SrtA) to anchor surface proteins to the cell wall and forms a biofilm to facilitate its adhesion to the tooth surface. Some plant natural products, especially several flavonoids, are effective inhibitors of SrtA. However, given the limited number of inhibitors and the development of drug resistance, the discovery of new inhibitors is urgent. Here, the high-throughput virtual screening approach was performed to identify new potential inhibitors of *S. mutans* SrtA. Two libraries were used for screening, and nine compounds that had the lowest scores were chosen for further molecular dynamics simulation, binding free energy analysis and absorption, distribution, metabolism, excretion and toxicity (ADMET) properties analysis. The results revealed that several similar compounds composed of benzofuran, thiadiazole and pyrrole, which exhibited good affinities and appropriate pharmacokinetic parameters, were potential inhibitors to impede the catalysis of SrtA. In addition, the carbonyl of these compounds can have a key role in the inhibition mechanism. These findings can provide a new strategy for microbial infection disease therapy.

## Introduction

**Figure 1:**
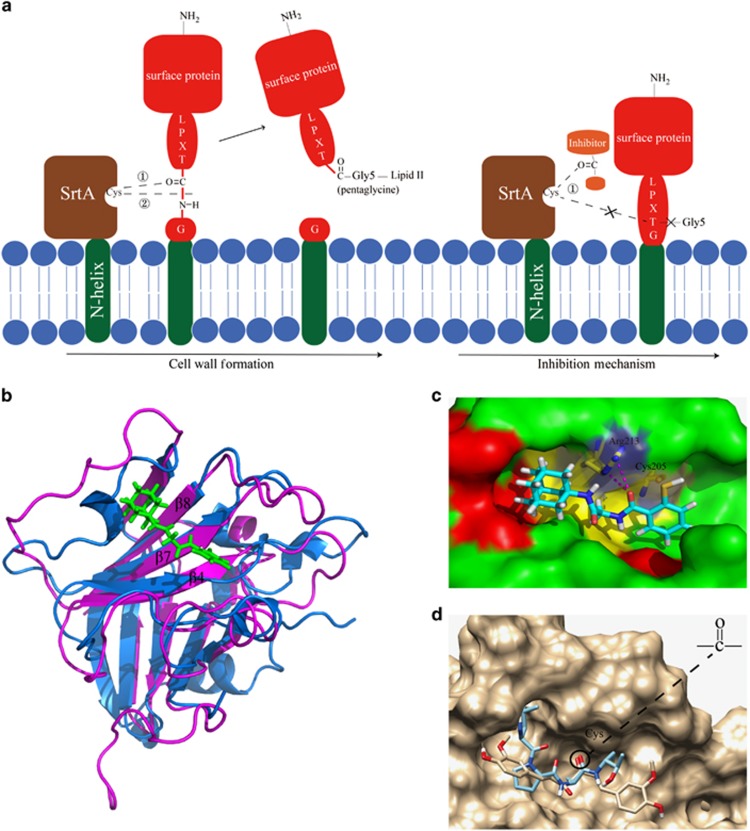
**Mechanism of SrtA catalysis and inhibition of SrtA and the active site determination of *S. mutans* SrtA in the catalytic domain using the crystal structure of *S. aureus* SrtA**. (**a**) In cell wall formation, ① Cys205 of SrtA recognizes the carbonyl of the LPXT-G motif of surface proteins. ② Cys205 attacks the peptide bond between threonine and glycine. Then, the exposed carboxyl of threonine is amide-linked to the pentaglycine cross-bridge to benefit biofilm formation. Regarding the inhibition mechanism, the carbonyl of inhibitors can replace that of surface proteins to inhibit catalysis. ① Cys205 recognizes the carbonyl of the inhibitor, then hydrogen bonds or Michael addition are formed between them to impede SrtA catalysis. (**b**) SrtA crystal structures of *S. mutans* (blue) and *S. aureus* (magentas) with its inhibitor (green). (**c**) The surface of *S. mutans* SrtA with the inhibitor of *S. aureus* SrtA in the active site after docking. Helix, sheet and loop surfaces are indicated in red, yellow and green, respectively. The colours of carbon, hydrogen, oxygen, nitrogen and sulfur atoms of the inhibitor are cyan, white, red, blue and orange, respectively. The colour of hydrogen bonds is magenta. (**d**) Curcumin (white) occupies the catalytic centre, and the LPXTG motif (cyan) does not enter the binding pocket. SrtA, sortase A.

Sortases, including sortase A, B, C, are a highly conserved transpeptidase family that widely exists in Gram-positive bacteria. Sortase A (SrtA), one of the membrane-associated sortase enzymes, is responsible for the covalent attachment of numerous virulence-associated surface proteins to host tissues.^1, 2, 3^ The anchoring of numerous surface proteins, including FruA, GbpC, Pac, WapA and Dex, requires a sorting signal with a conserved LPXTG motif (where X is any amino acid), a hydrophobic domain and a positively charged tail.^4^ In this mechanism, SrtA, which recognizes the hydrophobic motif at the C-terminus of surface proteins, cleaves the peptide bond between threonine and glycine. Then, the carboxyl of threonine is amide-linked to the pentaglycine cross-bridge of lipid II (Figure 1a). Finally, lipid II-surface protein complex is incorporated into peptidoglycan through transglycosylation and transpeptidation reactions, and this is involved in cell wall synthesis.^5, 6, 7, 8^ Several residues have important roles in the mechanism of specific recognition of *Staphylococcus aureus (S. aureus)* SrtA. For example, Cys184 attacks and cleaves the bond between the threonine and glycine of the LPXTG motif; Arg197 stabilizes the oxyanion-transition state; and His120 remains unprotonated in this process.^9^

Dental diseases, such as tooth decay, periapical periodontitis and endodontics, have gradually become major public health issues worldwide.^10, 11^ Risk for dental caries includes lifestyle-related, environmental and biological factors. Regarding biological factors, oral microflora located in both the crowns and roots of teeth have a key role in oral health.^12^ The primary pathogen of coronal and root caries are the mutans streptococci, especially *Streptococcus mutans* (*S. mutans*) and *Streptococcus sobrinus*, and some acid-tolerant strains, such as *Streptococcus sanguis*, *Streptococcus gordonii* and *Streptococcus oralis*, are also implicated in coronal caries.^13^ *S. mutans* is the major pathogen of dental caries.^14^ *S. mutans* utilizes SrtA to anchor some surface proteins to the cell wall, thus easily coalescing into host teeth. One of the principal virulence properties of *S. mutans* involves the formation of biofilms, also known as dental plaque, adhering to the tooth surface.^15, 16^ Because of the formation of biofilm and the acid-producing capacity of carbohydrates from bacterial fermentation, the teeth gradually exhibit enamel decalcification, ultimately leading to dental caries. Furthermore, a series of studies have demonstrated that after the deletion of *srtA*, most of surface proteins (Pac, Dex and GpbC) appear in the supernatant after centrifugation, whereas their presence in the cell wall is reduced.^17^ In addition, *srtA*-deficient *S. mutans* reduced the ability of mucoadhesion to oral mucosa and teeth.^18^ Hence, SrtA has an essential role in the interaction between *S. mutans* and its host and acts as a potential target to treat dental caries.

The crystal structure of the stable SrtA H139A mutant has been determined by DJ Wallock-Richards and his colleagues. This protein consists of a catalytic domain and an N-terminal helix transmembrane domain. The protein contains eight highly conserved β-strands, and three of these strands (β-4, β-7 and β-8) could form a hydrophobic pocket that is associated with the active site.^1^ Several SrtA inhibitors have been reported, and most of them are derived from flavonoids, such as morin^19^ and curcumin.^14^ Moreover, four compounds retain high inhibitory activity for SrtA.^20^ However, few small molecules (<12, including their isomers) are available that suppress the transpeptidases. Antibiotics used in dentistry appear to increase bacterial resistance, and natural products can make a favourable contribution in this field.^21^ Furthermore, computer-aided drug design is a popular method to perform high-throughput virtual screening of drugs, thus reducing time expenditures and experimental validations.^22^ In the present study, DOCK6 (http://dock.compbio.ucsf.edu/DOCK_6/index.htm) was utilized for molecular docking to screen potential SrtA inhibitors from the Specs library (http://www.specs.net/snpage.php?snpageid=home) and the TONGTIAN library (http://www.tautobiotech.com/). Second, molecular dynamics (MD) simulation, binding free energy prediction and energy decomposition were performed to estimate the stability and interaction of SrtA-inhibitor complexes. In addition, absorption, distribution, metabolism, excretion and toxicity (ADMET) prediction was performed to estimate basic pharmacological properties. The strategy of this study can provide an important measurement and perspective to better understand the inhibitory mechanism of SrtA for future dental caries therapies.

## Materials and methods

### Data preparation

Given the significant inhibitory activity of some plant extracts for SrtA, we adopted 32 791 compounds from the Specs library in the ZINC database. Moreover, the 2 172 mol2 files of compounds and their isomers were downloaded from TONGTIAN Chinese herb medicines library and ZINC database. The crystal structure of the SrtA H139A mutant^1^ (PDB code: 4TQX) with the ambiguous N-terminus 41–53 residues deleted was downloaded from the RCSB Protein Data Bank (PDB). However, there was no co-crystallized inhibitor in 4TQX. To provide precise position of the active site, the matchmaker function in UCSF Chimera^23^ was used to match 4TQX and SrtA from *S. aureus* (highly homologous,^14^ especially the active site) in complex with a benzo[d]isothiazol-3-one based inhibitor (PDB code: 2MLM). Then, *S. aureus* SrtA was deleted, and the *S. aureus* SrtA inhibitor and *S. mutans* SrtA H139A mutant remained. The localization of the inhibitor can be used as a docking site.

### Molecular docking and model validation

The receiver operating characteristic curve (ROC) and the area under ROC curve (AUC) were utilized to evaluate the accuracy of docking. Eleven inhibitors from the literature with IC_50_ values <100 μmol·L^−1^ were considered as the positive datasets.^1, 14, 19, 20^ Inhibitor structures that were not identified in the ZINC database were generated by ChemDraw, and energy was minimized by Chimera. In total, 210 decoys were identified as negative datasets by DecoyFinder according to the following parameters. Active ligand vs decoy Tanimoto threshold and decoy vs decoy Tanimoto threshold were 0.75 and 0.90, respectively. Acceptors and donors of hydrogen bonds were ±2 and ±1, respectively. Molecular weight was 25 Da and rotational bond was ±1. Both positive and negative datasets were docked against *S. mutans* SrtA, and then the docking result was estimated by ROC curve and AUC using pROC package in R.^24^ AUC >0.9 has high accuracy, 0.7–0.9 indicates moderate precision, and an AUC of 0.5 means a random event.^25^ DOCK6 and AutoDock (http://autodock.scripps.edu/) were performed to estimate the performance of the screening system with four algorithms: Grid score, Amber score, Descriptor score and Hawkins generalized born (GB)/surface area (SA) score. The last three algorithms were based on the Grid score in DOCK6 and the AutoDock algorithm in AutoDock using ROC performance. (1) If the performance of DOCK6 was better than AutoDock, a Grid score and an algorithm with favourable AUC values were selected to screen the large-scale libraries. (2) Conversely, AutoDock was selected to screen the large-scale libraries.

Chimera was adopted for structure preparation before docking, where hydrogens atoms and standard charges were added to SrtA and the *S. aureus* SrtA inhibitor. After determining the active centre by the coordinates of the *S. aureus* SrtA inhibitor, UCSF DOCK6 was used to screen the large-scale small molecule libraries (Specs and TONGTIAN) against SrtA according to DOCK 6.7 tutorials.^26^ To make as many molecules dock into the binding pocket as possible, spheres within 8.0 Å root mean square deviation (RMSD) from every atom of the inhibitor crystal structure and 1 000 maximum orientations were established. Other parameters were kept as default values. Similarly, AutoDock was used to screen compounds by AutoDock tutorials. (1) DOCK6 strategy: we selected top 20 compounds from each of the two algorithms (selected by ROC performance) for a total of 40 molecules, including repeated compounds, and several compounds exhibiting the lowest score in both algorithms were chosen for further analysis. (2) AutoDock strategy: 10 compounds exhibiting the lowest score were chosen for further analysis.

### Molecular dynamics simulations and MD trajectories analysis

GROMACS 4.5.5(ref. 27) software was used to evaluate the stability of the protein–ligand complex. From the beginning, the protein topology and the ligand topology were prepared by pdb2gmx of GROMACS and AmberTools,^28^ respectively, where the TIP3P water model and the Amber ff99SB force field were used for protein. Hydrogen atoms and charges were added to the ligand. Next, the system was defined under a periodic boundary condition using a dodecahedron box, which was set at a 1.0 Å minimal distance in the midst of the protein and the edge of the box with solvent. To make the system electrically neutral, sodium ions and chlorine ions were placed in the box at a concentration of 0.15 mol·L^−1^. Other parameters remained as defaults. Then, energy minimization was performed to ensure against steric clashes and inappropriate geometry in the system. Before starting the simulations, 100 ps NVT (isothermal-isochoric) and 100 ps NPT (isothermal-isobaric) were applied to equilibrate ions and the solvent surrounding the complex by position restraints to avoid collapse of the system. Finally, the 35 ns MD simulations began to run under the well-equilibrated system with a time step of 2 fs simulations of relieved position restraints by the mdrun program.

The MD trajectories analysis of the protein–ligand complex was enforced by GROMACS utilities in comparison with two reported inhibitors. The RMSD analysis was performed by the g_rms program with the entire 35 ns trajectories, and per-residue root mean square (RMSF) analysis was performed by the g_rmsf program with time ranges of relatively stable structures (slight fluctuation of RMSDs).

### Molecular mechanics (MM)/poisson-boltzmann surface area (PBSA) binding free energy calculations and energy decompositions

Molecular mechanics (MM)/poisson-boltzmann surface area (PBSA) possesses a considerably more efficient complementary role to traditional free energy calculations for drug discovery.^29^ This approach calculates binding free energy (ΔG_binding_) according to the following Equations:


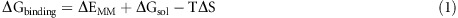



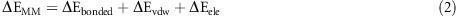



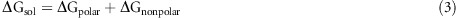


where ΔG_binding_ consists of the gas-phase interaction energy (ΔE_MM_) and the solvation free energy (ΔG_sol_). In addition, -TΔS, which represents conformational entropy change on ligand binding, is ignored due to expensive computational costs and low prediction accuracy. ΔE_MM_ includes van der Waals energy (ΔE_vdm_), electrostatic interaction energy (ΔE_ele_) and ΔE_bonded_; however, ΔE_bonded_, such as bond, angle and torsion energy, are typically considered as zero due to identity conformation of the protein–ligand complex in the single trajectory approach. In addition, ΔG_sol_ is quantified by two energies: the polar energy (ΔG_polar_ also called ΔG_PB_) estimated by the continuum solvent Poisson–Boltzmann model (PB) and the nonpolar energy (ΔG_nonpolar_ also called ΔG_SA_) estimated by the solvent accessible surface area.^30^ Hence, we adopted four energies to calculate the binding free energy in the following equation:


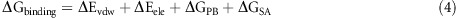


In the present study, the time range of structures with gentle RMSD during the 35 ns MD trajectories of each small molecule was selected to calculate the binding free energy with the g_mmpbsa program of the GROMACS tool using the above formula. To gain crucial residues in the protein–ligand interaction, we calculated the average binding free energy contribution of each residue, and the per-residual MM/PBSA free energy decomposition was also the sum of the van der Waals energy (ΔE_vdm_), electrostatic interaction energy (ΔE_ele_), polar energy (ΔG_PB_) and nonpolar energy (ΔG_SA_).

We selected the average conformations of SrtA-compounds obtained from stable MD state to analyse the hydrogen bonds and hydrophobic interactions between key residues using LIGPLOT^+^ (http://www.ebi.ac.uk/thornton-srv/software/LigPlus/).

### ADMET analysis

Properties of reported and potential inhibitors, including absorption, distribution, metabolism, excretion and toxicity, were predicted by the admetSAR online tool (http://lmmd.ecust.edu.cn:8000/predict/) or obtained from the Specs library and ChEMBL (https://www.ebi.ac.uk/chembl/). Prediction was conducted with analysis of the logP (octanol/water), rat oral LD_50_ and human intestinal absorption.

## Results

### Determining the active site

As shown in Figure 1b, structure matching between the crystal structure of *S. aureus* SrtA (magentas) with inhibitor (green) and *S. mutans* SrtA (blue) revealed that the localization of the active site was similar. Then, we docked the inhibitor from the *S. aureus* SrtA complex against *S. mutans* SrtA to confirm the reliability of the active site. The docking results demonstrated that several crucial residues, such as Cys205 and Arg213, were localized in this predicted active centre, and two hydrogen bonds between Arg213 of SrtA and the inhibitor were formed (Figure 1c). This finding indicates that the active site in *S. mutans* SrtA identified using DOCK 6.7 was relatively accurate and could be adopted to perform further large-scale screening.

### Model validation and molecular docking for potential inhibitors of *S. mutans* SrtA

**Figure 2:**
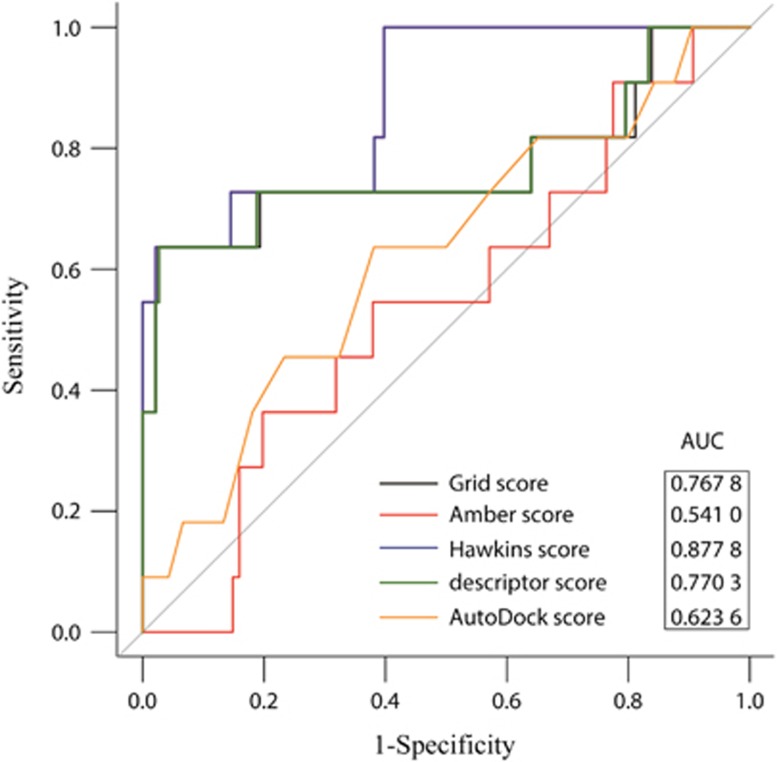
**ROC curves of docking test with 11 inhibitors and 210 decoys**. ROC, receiver operating characteristic curve.

We utilized testing sets containing 11 positive and 210 negative ligands to estimate the accuracy of DOCK6 and AutoDock for screening SrtA inhibitors. As shown in Figure 2, the Hawkins GB/SA score in DOCK6 was applied to screen compounds from libraries compared with other algorithm and AutoDock strategies. Given that the Hawkins GB/SA score is based on the Grid score, the top 60% ranked compounds from the Grid score docked by the Hawkins GB/SA score accounted for 0.945 (Supplementary Tables S1 and S2 and Supplementary Figure S1) of the highest AUC. All the above results reveal the substantial accuracy and applicability of the docking process.

The properties and scores of these putative compounds are provided in Tables 1 and 2. All of the selected compounds from the Specs library coincided with a general formula, and acteoside (ZINC95098840), oleuropein (ZINC98230413) and naringin (ZINC08681509), belonging to phenylpropanoid glycosides, secoiridoids and flavonoids, respectively, were screened out from the TONGTIAN library. Hydrogen bonding interactions of nine (six lowest score compounds from Specs library and all compounds except for the isomer of oleuropein from TONGTIAN library) molecules exhibited higher scores in these two libraries (Supplementary Figure S2), and several crucial reported residues, such as Cysteine and Arginine, were also identified in our analysis. Consequently, these nine compounds were selected for further analysis.

### MD simulations analysis

**Figure 3:**
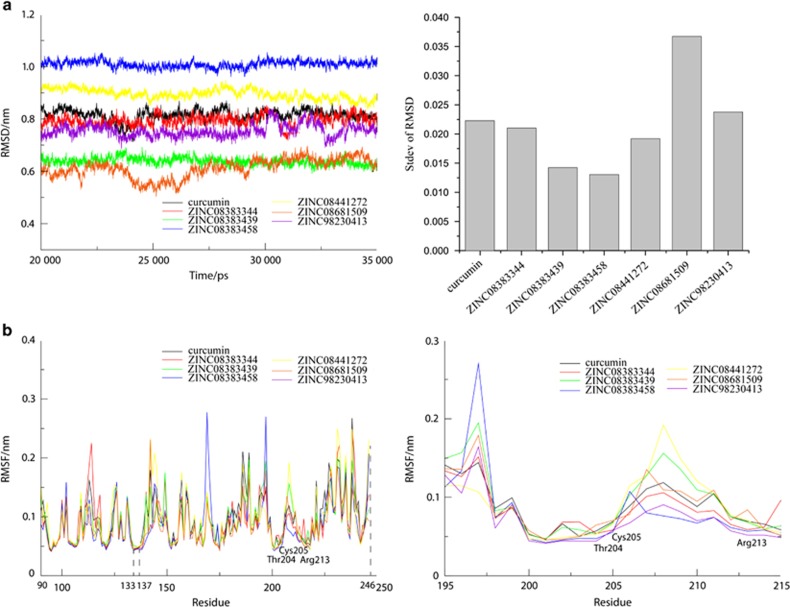
**RMSD and RMSF analyses of seven SrtA-ligand complexes**. (**a**) The backbone and standard deviation of RMSD for SrtA-ligand complexes during 20 to 35 ns MD trajectories. (**b**) Comparison of the backbone RMSF for each SrtA-ligand system during each stable MD trajectory and the crucial residue at the active site. MD, molecular dynamics; RMSD, root mean square deviation; RMSF, root mean square; SrtA, Sortase A.

To estimate the stability of SrtA-ligand complexes, two reported inhibitors, curcumin^14^ and kaempferol-3-rutinoside,^20^ were selected to compare with the nine compounds using the 35 ns MD simulations. As shown in Supplementary Figure S3, curcumin (red) attained high stability after 3 ns, whereas another inhibitor (black) exhibited large fluctuations during the MD trajectories. Several compounds exhibiting poor binding ability, including ZINC08383331 and ZINC08383950 from the Specs library and ZINC95098840 from the TONGTIAN library, were excluded. As noted in Figure 3a, compared with the known inhibitor curcumin, the remaining SrtA-ligand complexes exhibited enhanced binding stability during the 20–35 ns MD trajectories.

RMSF analysis was performed to estimate the average level of volatility of each residue. As represented in Figure 3a and 3b relatively stable timeframe (20-35 ns) was selected to analyse the flexibility of SrtA residues. These complexes have similar peak and low values. Two key residues, Cys205 and Arg213, showed low fluctuation, suggesting a tight interaction between compounds and SrtA.

### Binding free energy and energy decomposition analysis

Binding free energies of the six compounds were calculated by MM/PBSA algorithm during their steady state. Generally, van der Waals energy made the most contribution to the total binding free energy. As shown in Table 3, most potential inhibitors exhibited more favourable binding free energy compared with curcumin at >−30 kcal·mol^−1^, and ZINC08383458 possessed the best binding energy. Although naringin (ZIN08681509) belonged to flavonoids, the binding free energy was the lowest compared with other compounds and curcumin.

**Figure 4:**
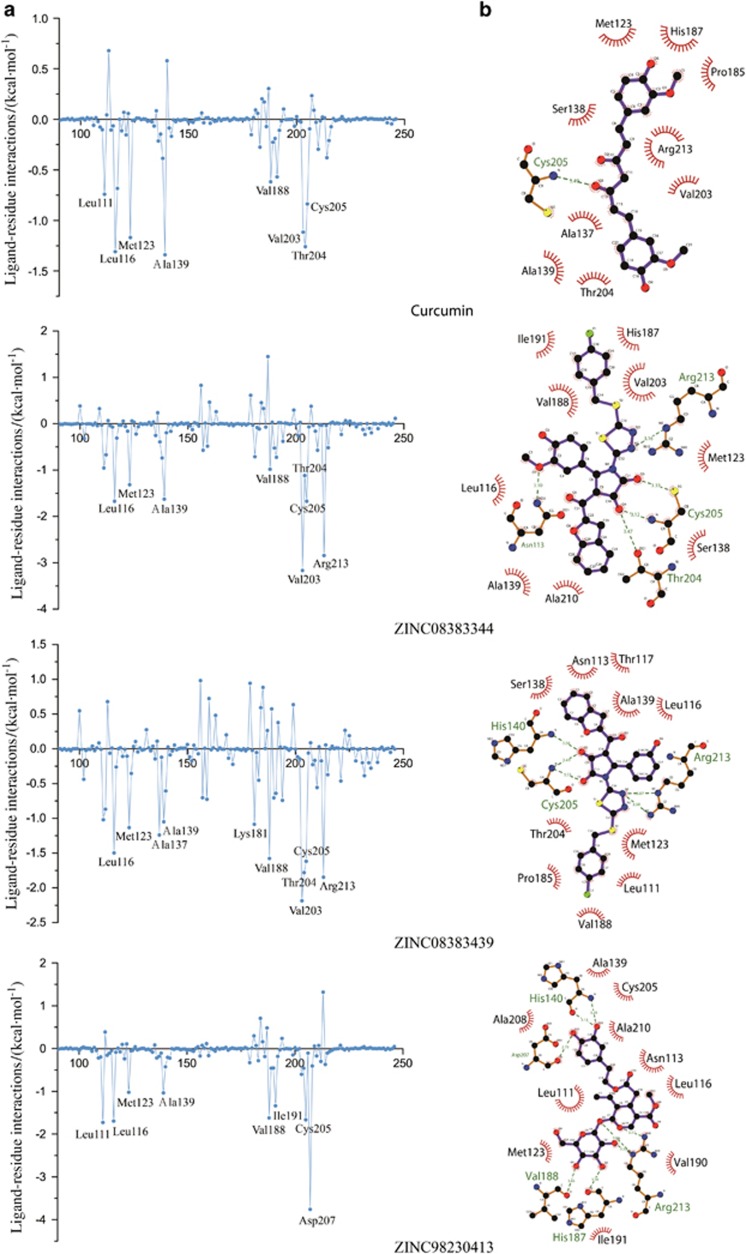
**Binding free energy decomposition for per residue and interactions of each SrtA-compound complex in comparison with curcumin (ZINC08383458 and ZINC08441272 are listed in Supplementary Information)**. (**a**) Several crucial residue contributions of SrtA-compounds complexes. (**b**) Hydrogen bonds and hydrophobic interactions between SrtA and compounds. Hydrogen bonds and hydrophobic interactions are presented as green dotted line and red arcs, respectively. SrtA, sortase A.

To calculate the energy contributions of key residues in SrtA-compounds complexes, the binding free energy of each residue was decomposed using the MM/PBSA method. As noted in Figure 4, the candidates (ZINC08383344, ZINC08383439, ZINC08441272 and ZINC08383458) that had stable RMSD in Specs exhibited remarkable interactions towards the critical residues of SrtA. Compared with curcumin, significant differences in compounds were noted in crucial residues, such as Cys205 and Arg213, and residues with great contributions, such as Leu116, Met123, Ala139, Val188, Val203 and Thr204, especially that of ZINC08383458 (*P*<0.006) and ZINC08383439 (*P*<0.009). Specifically, these energy contributions were largely attributed to Val203, Thr204, Cys205 and Arg213 at the active centre. However, oleuropein (ZINC98230413) exhibited minimal differences with the other compounds, given that Asp207 occupied most contributions in this system (Figure 4), suggesting it might form a new binding mechanism to inhibit the sortase activity.

As shown in Figure 4 and Supplementary Figure S4, Cys205 and Arg213 participated in the formation of hydrogen bonds between all potential inhibitors and SrtA except for oleuropein (ZINC98230413), whereas curcumin only formed a hydrogen bond with Cys205. In addition, almost all the carbonyl of compounds formed hydrogen bonds with Cys205. Moreover, His140 was involved in hydrogen bond formation for ZINC08383439 and ZINC08383458, and Asn113 and Thr204 were involved in hydrogen bond formation for ZINC08383344. Notably, compared with curcumin, these potential compounds held more powerful hydrophobic effects.

### ADMET properties

As shown in Table 4 and Supplementary Table S3, several important pharmacokinetic parameters were predicted by admetSAR. All screened compounds exhibited no AMES toxicity and carcinogenic effects and possessed better human intestinal absorption, except for oleuropein (ZINC98230413). Compared with curcumin and trans-chalcone, these compounds had low caco-2 permeability; however, all compounds exhibited reduced toxicity (especially ZINC08383344) and high partition coefficients (LogP). Our ADMET studies suggested that these potential inhibitors can be more efficacious to use for dental caries therapy.

## Discussion

Dental caries is a chronic disease affecting a significant proportion of the population worldwide. As a result of poor oral hygiene and dietary habits, microflora in teeth exhibit subtle variations.^13, 31^ *S. mutans*, one of the main pathogens of dental caries, adheres to the tooth surface via cell wall surface proteins and dental plaque, thus producing pathogenic acid metabolites by fermentation of carbohydrates and resulting in demineralization of tooth tissues.^12, 13, 32^ Recent studies demonstrated that some oral bacteria produce alkali to decrease mouth acidity and thus prevent cavities.^33^ Moreover, inhibiting SrtA activity may become an effective therapy for dental caries.^14, 19^ In the present study, we screened compounds against SrtA that catalysed surface protein binding to the cell wall. To estimate the accuracy of our screening system, we removed low AUCs for the Amber score, Descriptor score and the algorithm of AUTODOCK and selected the Hawkins GB/SA score combined with the Grid score to screen the compound libraries (Figure 2). The Grid score is based on the anchor-and-grow algorithm with a flexible ligand dock to a rigid receptor.^34^ The Hawkins GB/SA scoring algorithm is a pairwise descreening approximation algorithm using the Molecular Mechanics Generalized Born Surface Area (MM-GBSA) method.^35^ A combination of these two algorithms could increase screening accuracy. For the RMSD analysis, we observed a large fluctuation during the early stage of the MD simulations, which was caused by instability of the transmembrane domain in the N-terminal helix. However, some compounds displayed more stable equilibrium status at the later stage of the MD simulations (Supplementary Figure S3), suggesting that these compounds might have a strong affinity against SrtA, especially ZINC08383458 and ZINC08383439, which exhibit reduced standard deviations (stdev) of RMSD (0.013 0 and 0.014 2) compared with curcumin (0.022 3; Figure 3a). According to RMSF and energy decomposition analysis, several key residues (Cys205, Arg213) highly homologous to *S. aureus* SrtA (Cys184, Arg197) participated in identification and inhibition, such as the low RMSF values and significant contribution of Cys205 and Arg213 (Figures 3 and 4). Furthermore, most compound-SrtA complexes exhibited similar hydrogen-bonding patterns, hydrophobic interactions (Figure 4) and appropriate ADMET properties (Table 4) and appeared to be superior to curcumin. These results suggest that these potential inhibitors can act as novel drugs to inhibit biofilm formation.

Several studies have demonstrated that the groove formed by β-4, β-7 and β-8 of the sortase superfamily consists of a conserved catalytic active site. The LPXTG motif of surface proteins could be recognized by this site and thus anchor the protein to lipid II.^3^ Intriguingly, as shown in Figures 3, β-4 (N133-A137), β-7 (E199-C205) and Arg213 in β-8 in the catalytic centre exhibited low RMSF coinciding with the conserved active conformation, indicating high reliability and accuracy of the MD process. Recent studies have demonstrated that some inhibitors could target surface proteins, such as GTFs, to impede plaque formation.^36^ Moreover, a study has reported that LPXTG peptide blocker compounds targeting the Leu residue might inhibit SrtA-mediated transpeptidation reaction,^37^ and a point mutation of SrtA could also affect its catalysis.^38^ In contrast, various inhibitors, including curcumin and morin, could decrease the activity of SrtA by interacting with crucial cysteine that participates in breaking the T-G bond from the LPXTG motif. This finding suggests that these inhibitors occupy the LPXTG motif binding site to reduce transpeptidation and thus impact biofilm formation. In our study, this mechanism was adopted to screen potential inhibitors, which might directly and fundamentally decrease SrtA catalysis compared with the methods mentioned above (Figure 1d). Herein, two reported inhibitors were chosen as controls to occupy the binding site. One inhibitor (curcumin) exhibited good RMSD, whereas another (kaempferol-3-rutinoside) exhibited opposing values. The reason for this difference seemed to be that the inhibitory effect of kaempferol-3-rutinoside (IC_50_=60.7μmol·L^−1^±1.2 μmol·L^−1^ (ref. 20)) was reduced compared with curcumin (IC_50_=10.2μmol·L^−1^±0.7 μmol·L^−1^ (ref. 14)). Therefore, these results confirmed the accuracy of MD simulations as one of the methods to screen potential inhibitors.

Similar studies regarding the identification of inhibitors of *S. aureus* SrtA have been reported.^39, 40, 41^ Although there is high amino acid sequence homology (29%) between the SrtA of *S. aureus* and *S. mutans*,^14^ there is no evidence that homologous proteins have the same inhibitors. Moreover, compared with *S. mutans*, the major pathogen of dental caries, *S. aureus* is a leading cause of hospital- and community-acquired infections ranging from minor skin infections to osteomyelitis, meningitis, endocarditis, septicaemia and toxic shock syndrome.^41^ Therefore, targeting *S. mutans* SrtA may be more effective than targeting *S. aureus* SrtA. In contrast to these studies, we evaluated the docking model via ROC curves and utilized MM-PBSA to obtain the contribution of key residues, and all of these improved the performance of the screening model. This strategy can screen some of the missing inhibitors and exclude some of the useless compounds.

Several plant natural products, especially flavonoids, inhibit *S. mutans* SrtA through Michael addition^1^ or other methods. Branched chains of flavone can also decrease SrtA activity, such as kaempferol-3-rutinoside.^20^ In the TONGTIAN library, a flavonoid naringin (ZINC08681509) was excluded. However, all flavonoids in the TONGTIAN library were excluded after docking (Supplementary Table S2). The reasons for this finding seemed to be that the branched chain was long or/and contained heavy atoms resulting in steric clashes, or the compounds could never form great binding energy against SrtA. Several compounds with a general formula (Table 1) in the Specs library might also inhibit SrtA. As shown in Figure 4 and Supplementary Figure S4, five potential inhibitors in Specs indicated that the carbonyl group played an important role in forming hydrogen bonds with the cysteine or close to the residue. Coincidently, cysteine attacks the carbonyl of scissile Thr-Gly bond during the transpeptidation of SrtA.^9^ This finding suggests that the carbonyl of potential inhibitors can act as a “cheater” instead of the carbonyl of LPXT-G motif to reduce surface proteins anchoring to the cell wall (Figure 1a and 1d). Other compounds with the general formula had unstable RMSD, probably due to the large branched chains (ZINC08383331) or heavy atoms (ZINC08383950; Table 1). Moreover, in TONGTIAN, although oleuropein (ZINC98230413) had high binding free energy with SrtA, it probably could not serve as an inhibitor due to low contribution of the cysteine (Figure 4). Our results demonstrated that the formula of these compounds, including benzofuran that is associated with hydrophobic interactions, thiadiazole and pyrrole that are associated with hydrogen-bonding interactions and a crucial carbonyl that was recognized by SrtA, can act as novel drugs to inhibit biofilm formation.

## Conclusions

In the present study, a computer-aided drug screening method was performed to screen potential inhibitors of SrtA. Several preliminary compounds were screened in two libraries using DOCK6 and Gromacs. RMSD and RMSF analyses of the SrtA-compound complexes suggested that the selected compounds possessed high binding affinities with SrtA. Binding free energy calculations, energy decompositions and ADMET predictions were performed to determine drug-like candidates. Several compounds with a general formula can decrease dental caries, and the carbonyl of these compounds played a key role in inhibiting catalysis of SrtA. Hence, this study provides a new strategy for treating disease associated with bacterial adhesion and other invasive oral diseases.

## Figures and Tables

**Table 1:**
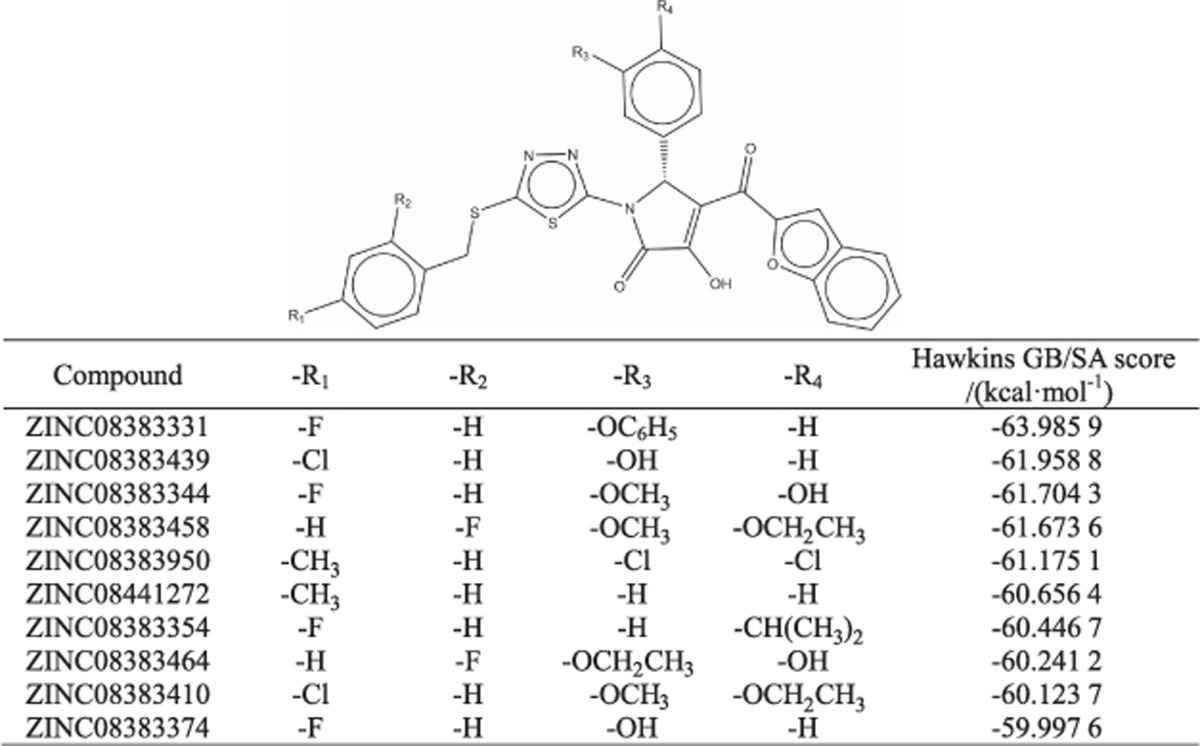
A general formula of preliminary compounds and their properties and Hawkins generalized born/surface area scores in Specs library after docking

**Table 2:**
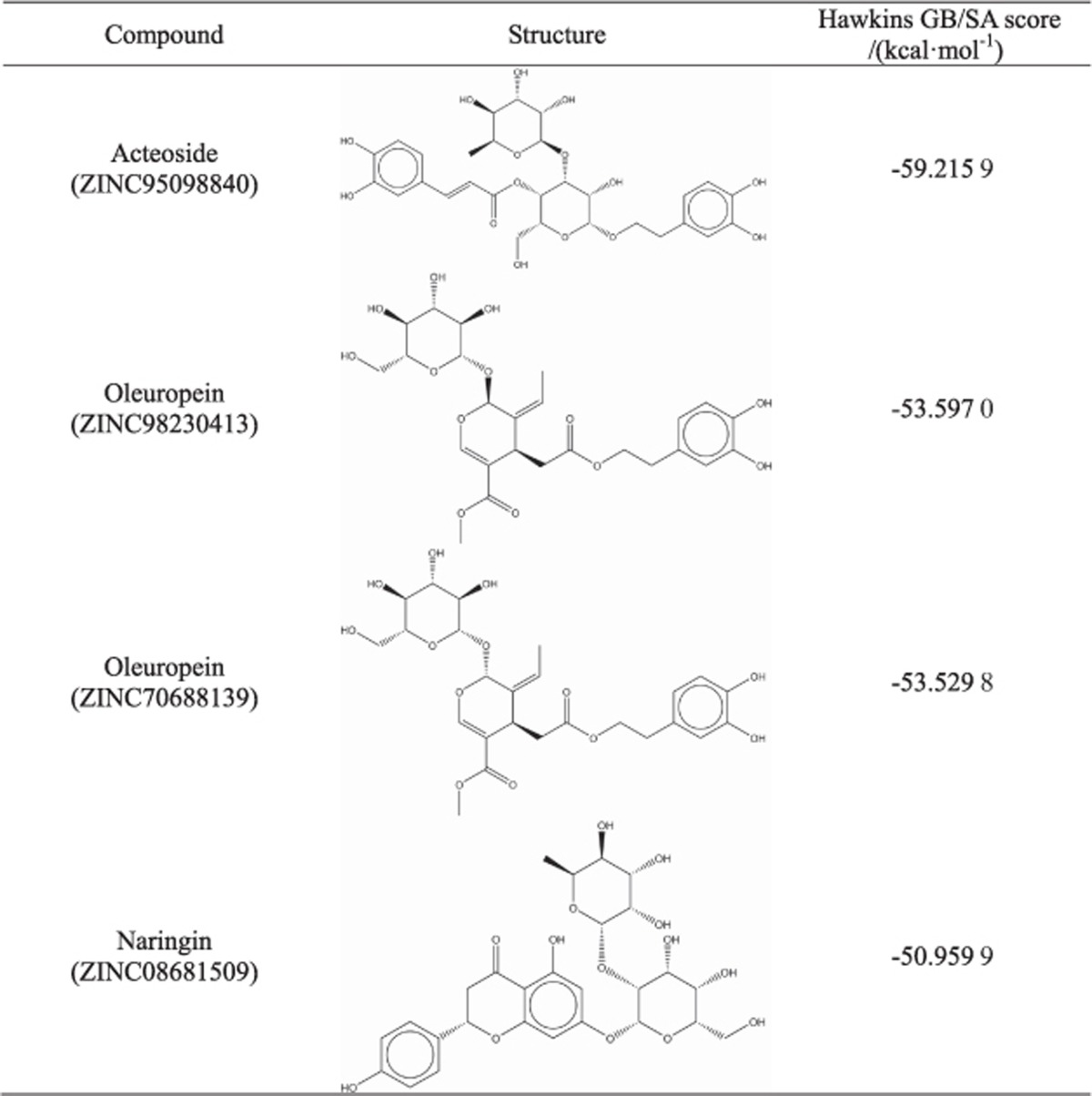
Structure and Hawkins generalized born/surface area score of preliminary compounds in TONGTIAN library after docking

**Table 3:** Binding free energy calculations of SrtA-compound complexes using the molecular mechanics/poisson-boltzmann surface area approach

Compound	Timeframe/ns	ΔE/(kcal·mol^−1^)				
		ΔE_vdw_	ΔE_ele_	ΔG_pb_	ΔG_sa_	ΔG_bind_
Curcumin	4–7	−40.89±3.23	−19.38±4.44	42.38±3.86	−4.48±0.24	−22.37±4.09
ZINC08383344	20–25	−56.53±3.35	−26.35±4.30	56.09±3.53	−5.53±0.21	−32.32±3.98
ZINC08383439	28–32	−52.23±2.92	−18.24±3.62	48.26±2.81	−4.86±0.19	−27.07±3.39
ZINC08383458	24–36	−55.16±2.73	−24.11±3.43	49.15±2.46	−5.03±0.25	−35.14±2.72
ZINC08441272	15–20	−55.77±2.70	−22.66±3.73	49.51±2.47	−4.96±0.20	−33.88±3.24
ZINC08681509	27–29	−28.37±2.87	−20.52±10.00	36.65±8.23	−3.30±0.30	−15.54±3.44
ZINC98230413	25–29	−51.07±3.29	−37.90±5.53	64.15±4.90	−5.50±0.22	−30.31±4.05

SrtA, sortase A.

**Table 4:** ADMET prediction of five potential inhibitors compared with curcumin

Compound	LogP*	LD_50_†/(mg·kg^−1^)	HIA‡	Caco-2§	AMES toxicity	Carcinogen
Curcumin	2.56	2 600	HIA+(0.953 9||)	Caco2+(0.709 3||)	Non-AMES toxic	Non-carcinogen
ZINC08383344	4.21	8 370	HIA+(0.971 3)	Caco2−(0.601 4)	Non-AMES toxic	Non-carcinogen
ZINC08383439	4.83	5 200	HIA+(0.985 5)	Caco2−(0.619 9)	Non-AMES toxic	Non-carcinogen
ZINC08383458	5.00	5 940	HIA+(0.981 5)	Caco2−(0.571 6)	Non-AMES toxic	Non-carcinogen
ZINC08441272	5.22	5 250	HIA+(0.993 6)	Caco2−(0.597 4)	Non-AMES toxic	Non-carcinogen
ZINC98230413	−0.87	4 102	HIA−(0.908 1)	Caco2−(0.765 5)	Non-AMES toxic	Non-carcinogen

ADMET, absorption, distribution, metabolism, excretion and toxicity; AMES, reverse mutation assay; HIA, human intestinal absorption; LD_50_, median lethal dose.

* Octanol/water partition coefficient, low logP indicates increased hydrophilicity.

† Rat oral LD_50_, low toxicity: 500 mg·kg^−1^<LD_50_<5 000 mg·kg^−1^. Actually non-toxic: 5 000 mg·kg^−1^<LD_50_<15 000 mg·kg^−1^.

‡ HIA+: >30% of HIA%, HIA-: <30% of HIA%.

§ Caco-2 permeability, Caco2+: high Caco-2 permeability (Papp ⩾ 8 × 10^−6^ cm·s^−1^), Caco2-: moderate-poor permeability (Papp <8 × 10^−6^ cm·s^−1^).

|| The probability of HIA and Caco-2 permeability.
