# Supplementary material for: In silico identification of potential inhibitors targeting Streptococcus mutans sortase A
Source: Int J Oral Sci. 2017 Mar 30;9(1):53–62. doi: 10.1038/ijos.2016.58 (PMC5379162; doi:10.1038/ijos.2016.58)
Supplement: Supplementary Information [file ijos201658x1.doc]

Supplementary tables and figures

Table S1 **Top 60% ranked compounds in Specs library (the yellow fill color of scores are top 20 of each score).**

Table S2. **Top 60% ranked compounds in TONGTIAN library (the yellow fill color of scores are top 20 of each score).** The compounds which belonged to flavonoids were labeled. Possible reasons to be eliminated of the flavonoids were also listed in this table.

Table S3 ADMET prediction of some inhibitors (their isomers have the same properties).

| Compound | LogP^a^ | LD_50_^b^  （mg kg^-1^） | HIA^c^ | Caco-2^d^ | AMES Toxicity | Carcinogen |
| --- | --- | --- | --- | --- | --- | --- |
| Curcumin | 2.56 | 2600 | HIA+(0.9539^e^) | Caco2+(0.7093^e^) | [Non AMES toxic](../T_AMES_I) | [Non-carcinogen](../T_Carc_I) |
| Morin | 4.21 | 3919 | [HIA+(0.9855)](../A_HIA_I) | [Caco2-(0.7447)](../A_Caco2_I) | [Non AMES toxic](../T_AMES_I) | [Non-carcinogen](../T_Carc_I) |
| trans-chalcone | 3.08 | 1048 | [HIA+(1.0000)](../A_HIA_I) | [Caco2+(0.9354)](../A_Caco2_I) | [Non AMES toxic](../T_AMES_I) | [Non-carcinogen](../T_Carc_I) |

Other four inhibitors^20^ were not predicted due to lack of SMILE format, but they comes from Korean folk medicines which were low toxicity.

Fig S1 **ROC curve of docking test for each percentage of Gride score of compounds.**

Fig S2 **Results of docking for Nine compounds with highest Grid scores and Hawkins GB/SA score.** The color of SrtA was green. The color of carbon, hydrogen, oxygen, nitrogen and sulfur atoms of the inhibitor were cyans, white, red, blue and orange, respectively. The color of hydrogen bonds was yellow.

Fig S3 **The backbone of RMSD of nine compounds in specs and TONGTIAN libraries in comparison with two reported inhibitors, curcumin (red) and kaempferol-3-rutinoside (black).** (a) RMSD for specs library. (b) RMSD for TONGTIAN library.

Fig S4 **Binding free energy decomposition for per residue and interactions of ZINC08383458- and ZINC08441272-SrtA complexes.** (**a**) Several crucial residues contributions of SrtA-compounds complexes. (**b**) Hydrogen bonds and hydrophobic interactions between SrtA and compounds. Hydrogen bonds and hydrophobic interactions are shown in green dotted line and red arcs.
