# Supplementary material for: In silico identification of potential inhibitors targeting Streptococcus mutans sortase A
Source: Int J Oral Sci. 2017 Mar 30;9(1):53–62. doi: 10.1038/ijos.2016.58 (PMC5379162; doi:10.1038/ijos.2016.58)
Supplement: Supplementary Table S1 [file ijos201658x2.pdf]

Table S1. Top 60% ranked compounds in Specs library (the yellow fill color of scores are top 20 of each score).

Compound	Gride score	Hawkins GB/SA score
ZINC08383331	−58.4854	−63.9859
ZINC19363139	−52.5207	−63.0967
ZINC19363139	−47.5318	−63.0967
ZINC08383439	−56.7497	−61.9588
ZINC08383344	−56.3738	−61.7043
ZINC08383458	−55.3566	−61.6736
ZINC08400598	−51.6119	−61.4801
ZINC08400598	−47.3713	−61.4801
ZINC08383403	−53.9105	−61.3154
ZINC08383950	−55.1959	−61.1751
ZINC08441272	−55.4029	−60.6564
ZINC08383354	−55.7322	−60.4467
ZINC16734748	−52.7473	−60.3347
ZINC08441222	−52.1914	−60.3107
ZINC08384028	−51.2257	−60.2734
ZINC08383464	−54.6832	−60.2412
ZINC08383410	−55.6401	−60.1237
ZINC08441236	−54.4623	−60.0035
ZINC08384010	−54.3376	−60.0028
ZINC08383374	−54.7432	−59.9976
ZINC08383984	−52.5452	−59.9737
ZINC08441255	−55.7307	−59.9325
ZINC08441253	−54.4504	−59.8137
ZINC08441673	−52.6277	−59.2837
ZINC00703059	−51.7763	−59.2637
ZINC00703059	−47.0136	−59.2637
ZINC08383400	−54.5275	−59.2030
ZINC08384082	−55.6249	−59.1979
ZINC08383419	−51.0070	−59.0924
ZINC08384060	−53.5838	−59.0233
ZINC08383973	−51.9406	−58.9539
ZINC08383610	−56.7727	−58.9223
ZINC08441219	−51.4592	−58.8194
ZINC08384072	−52.5903	−58.7800
ZINC08441208	−50.5722	−58.5178
ZINC08441749	−52.8353	−58.4420
ZINC08441751	−52.9033	−58.4291
ZINC06444294	−49.5489	−58.3927
ZINC06444294	−47.4087	−58.3927
ZINC08441265	−52.2549	−58.0508
ZINC08384031	−52.9767	−57.9939
ZINC08383479	−55.4941	−57.8434
ZINC08397081	−48.6629	−57.7973
ZINC22936966	−50.3096	−57.6507
ZINC08441512	−52.5306	−57.6394
ZINC08383964	−52.4433	−57.5557
ZINC08441817	−51.3616	−57.5030
ZINC19901366	−48.2663	−57.4721
ZINC19901366	−46.6385	−57.4721
ZINC08384014	−51.3369	−57.3795
ZINC08441505	−49.5790	−57.2027

ZINC32616960	-50.4322	-57.1489
ZINC19367353	-48.4745	-57.1283
ZINC19367353	-46.7154	-57.1283
ZINC08384001	-51.9616	-57.0581
ZINC08434942	-49.9634	-56.9563
ZINC08383358	-51.0880	-56.9469
ZINC08383364	-51.9103	-56.8753
ZINC00726465	-50.3928	-56.8278
ZINC00726465	-49.5551	-56.8278
ZINC00726465	-48.7327	-56.8278
ZINC02183610	-48.5980	-56.7957
ZINC04067720	-50.4154	-56.7780
ZINC19367694	-49.6936	-56.7737
ZINC08441216	-53.5556	-56.6885
ZINC08441502	-49.6811	-56.5830
ZINC08441502	-49.1338	-56.5830
ZINC08383952	-50.7062	-56.5492
ZINC08441204	-54.3688	-56.4995
ZINC02183634	-47.1083	-56.4703
ZINC13739356	-46.9755	-56.4184
ZINC13552519	-51.3090	-56.3358
ZINC08915016	-48.3277	-56.2994
ZINC08915016	-47.3965	-56.2994
ZINC08429886	-52.7499	-56.1577
ZINC08400642	-52.4823	-56.0700
ZINC08441228	-52.8076	-55.9572
ZINC08440516	-50.4913	-55.9519
ZINC04067719	-48.8480	-55.9011
ZINC04067719	-47.3168	-55.9011
ZINC04067719	-46.5511	-55.9011
ZINC08396526	-48.1434	-55.8955
ZINC08439511	-55.1759	-55.8300
ZINC19366911	-52.4075	-55.8203
ZINC08383388	-50.2756	-55.7700
ZINC22936963	-51.5743	-55.7492
ZINC08383877	-48.1787	-55.7367
ZINC08383877	-42.1428	-55.7367
ZINC08439543	-52.7121	-55.7347
ZINC20028406	-47.3357	-55.7090
ZINC08385068	-51.2143	-55.6531
ZINC08433387	-52.2370	-55.6350
ZINC08441660	-50.9072	-55.5658
ZINC05918671	-51.8760	-55.5630
ZINC06137427	-51.4856	-55.5559
ZINC06195502	-46.7039	-55.5266
ZINC06195502	-43.8526	-55.5266
ZINC19901351	-47.2530	-55.4141
ZINC19901351	-43.1121	-55.4141
ZINC08441735	-51.4732	-55.3786
ZINC05918573	-50.6902	-55.3771
ZINC05918573	-43.2437	-55.3771
ZINC08383589	-51.5861	-55.3450
ZINC08441663	-48.3227	-55.3393
ZINC08429981	-53.6372	-55.2534

ZINC08429981	-48.5754	-55.2534
ZINC08429981	-45.0206	-55.2534
ZINC05360472	-54.6987	-55.1946
ZINC06195873	-49.2050	-55.1690
ZINC08384331	-50.2346	-55.1630
ZINC09460905	-49.6780	-55.1277
ZINC04067956	-48.3773	-55.1197
ZINC04067956	-47.0466	-55.1197
ZINC04067956	-44.2390	-55.1197
ZINC19369740	-47.9991	-55.1152
ZINC19369740	-42.9588	-55.1152
ZINC00859160	-54.2448	-55.1144
ZINC00859160	-51.5841	-55.1144
ZINC08383977	-52.3812	-55.1139
ZINC08384008	-50.7802	-55.1024
ZINC20030859	-48.7813	-55.0742
ZINC20030859	-44.4801	-55.0742
ZINC20030859	-43.7744	-55.0742
ZINC08441670	-50.3586	-54.9839
ZINC08441661	-48.6919	-54.9610
ZINC08399190	-47.6623	-54.8112
ZINC08399190	-45.7942	-54.8112
ZINC19369883	-47.5704	-54.8023
ZINC01252584	-50.0444	-54.7722
ZINC08383461	-50.4063	-54.7088
ZINC08385065	-52.6454	-54.6266
ZINC05918839	-51.2696	-54.6097
ZINC05918839	-46.1162	-54.6097
ZINC04068158	-50.1185	-54.5653
ZINC04068158	-47.0152	-54.5653
ZINC04068158	-45.3904	-54.5653
ZINC08433349	-50.7916	-54.5552
ZINC08383605	-52.6108	-54.5388
ZINC19923757	-44.3931	-54.4312
ZINC19923757	-42.3379	-54.4312
ZINC08433162	-49.5462	-54.4259
ZINC08433162	-47.5319	-54.4259
ZINC08440930	-51.6957	-54.4005
ZINC13286514	-47.8842	-54.3896
ZINC08429889	-44.1691	-54.3118
ZINC08429889	-43.2310	-54.3118
ZINC08434752	-54.2542	-54.2902
ZINC08434752	-53.3160	-54.2902
ZINC18077932	-50.1021	-54.2714
ZINC08426318	-51.7628	-54.1946
ZINC08426318	-51.1469	-54.1946
ZINC00703137	-48.4068	-54.1940
ZINC08441652	-47.9994	-54.1915
ZINC13548585	-47.9792	-54.0790
ZINC13548585	-47.8178	-54.0790
ZINC08444349	-46.6466	-54.0697
ZINC08444349	-46.0407	-54.0697
ZINC19369768	-42.2006	-54.0667
ZINC19369768	-38.4642	-54.0667

ZINC08441712	-47.2075	-54.0201
ZINC06162481	-49.6638	-53.9909
ZINC00703115	-49.8356	-53.9882
ZINC00702688	-48.1557	-53.9880
ZINC00702688	-47.3512	-53.9880
ZINC08439491	-54.7875	-53.9716
ZINC08439491	-53.0870	-53.9716
ZINC08439491	-47.5496	-53.9716
ZINC08439476	-51.2460	-53.8874
ZINC08437289	-50.3098	-53.8716
ZINC08437289	-49.1293	-53.8716
ZINC08437289	-46.9373	-53.8716
ZINC09425045	-49.3658	-53.8456
ZINC09425045	-45.6801	-53.8456
ZINC04068160	-49.7762	-53.8269
ZINC04068160	-45.8057	-53.8269
ZINC04068160	-39.2673	-53.8269
ZINC00703135	-48.8771	-53.8258
ZINC06162320	-45.5382	-53.7833
ZINC06162320	-42.1801	-53.7833
ZINC03947241	-51.7353	-53.7735
ZINC05918767	-48.0523	-53.7534
ZINC19781902	-41.8614	-53.7462
ZINC19781902	-38.7022	-53.7462
ZINC09329159	-48.2524	-53.7451
ZINC00703119	-47.8027	-53.7442
ZINC08383371	-50.3904	-53.7403
ZINC05918830	-53.0897	-53.7338
ZINC08425696	-50.2785	-53.7223
ZINC08439478	-50.5202	-53.7057
ZINC08439478	-47.6382	-53.7057
ZINC22934483	-48.7977	-53.6997
ZINC22934483	-47.3890	-53.6997
ZINC08441667	-49.2467	-53.6948
ZINC08441716	-51.1928	-53.6913
ZINC08383986	-51.8522	-53.6573
ZINC04112133	-48.9902	-53.6553
ZINC08384077	-51.0273	-53.6388
ZINC08384084	-49.6034	-53.6348
ZINC08429986	-47.2655	-53.6220
ZINC08429986	-45.5601	-53.6220
ZINC08439564	-48.4634	-53.6048
ZINC08439564	-44.7377	-53.6048
ZINC08439493	-56.0480	-53.5634
ZINC08439493	-47.5795	-53.5634
ZINC08396259	-48.6496	-53.5354
ZINC08902375	-46.7410	-53.5246
ZINC08902375	-44.6133	-53.5246
ZINC08441742	-48.1050	-53.4989
ZINC08441209	-49.1875	-53.4953
ZINC05918780	-49.6594	-53.4823
ZINC05918780	-44.3270	-53.4823
ZINC08441675	-49.4930	-53.4816
ZINC08439508	-53.9016	-53.4747

ZINC08439508	-53.4866	-53.4747
ZINC08439508	-50.6247	-53.4747
ZINC19369751	-42.3738	-53.4574
ZINC19369751	-38.6915	-53.4574
ZINC04064377	-47.0592	-53.4472
ZINC08441737	-47.2548	-53.4411
ZINC04112118	-47.4675	-53.4332
ZINC04112118	-41.5356	-53.4332
ZINC04112118	-39.0903	-53.4332
ZINC08441530	-48.9365	-53.4273
ZINC08441530	-47.4470	-53.4273
ZINC08383466	-49.9519	-53.3564
ZINC08383450	-48.5670	-53.3466
ZINC04061863	-48.9378	-53.3180
ZINC04061863	-44.7395	-53.3180
ZINC04061863	-42.3321	-53.3180
ZINC04067868	-48.4622	-53.3063
ZINC04067868	-45.5895	-53.3063
ZINC06197307	-50.2817	-53.2946
ZINC04112126	-44.0701	-53.2672
ZINC04112126	-43.0657	-53.2672
ZINC19802163	-45.2335	-53.2342
ZINC19802163	-43.5823	-53.2342
ZINC08433354	-51.9789	-53.2039
ZINC00986477	-44.7775	-53.1926
ZINC08695221	-50.6249	-53.1828
ZINC08385356	-52.0455	-53.1510
ZINC19938586	-45.9839	-53.1174
ZINC09339584	-46.2756	-53.0821
ZINC02070164	-47.6309	-53.0740
ZINC04067958	-51.4872	-53.0739
ZINC04067958	-49.7621	-53.0739
ZINC04067958	-46.1666	-53.0739
ZINC08384224	-47.3201	-53.0728
ZINC08384224	-45.9605	-53.0728
ZINC08384224	-44.4739	-53.0728
ZINC19901364	-45.9939	-53.0507
ZINC19901364	-43.5617	-53.0507
ZINC04065351	-49.8832	-53.0494
ZINC04065351	-48.8892	-53.0494
ZINC08433372	-51.4934	-53.0457
ZINC19781845	-41.7177	-53.0405
ZINC09460904	-47.8537	-53.0321
ZINC06197224	-46.5105	-53.0240
ZINC19781610	-41.7466	-53.0239
ZINC19781610	-39.2378	-53.0239
ZINC08996584	-49.1410	-53.0144
ZINC08996584	-43.1275	-53.0144
ZINC08442199	-44.3438	-52.9762
ZINC08383347	-50.0249	-52.9600
ZINC08439434	-49.7064	-52.9439
ZINC08439434	-40.1614	-52.9439
ZINC08441510	-51.0214	-52.9403
ZINC08433375	-48.4826	-52.9346

ZINC04062018	-47.3517	-52.9052
ZINC04062018	-47.1042	-52.9052
ZINC08384052	-46.3258	-52.8941
ZINC06015481	-46.9474	-52.8631
ZINC08441240	-50.3759	-52.8624
ZINC13570886	-46.3912	-52.8600
ZINC05360467	-52.3033	-52.8546
ZINC06137486	-50.6011	-52.8365
ZINC00703117	-47.4313	-52.8352
ZINC06197201	-45.3735	-52.8211
ZINC06197201	-43.4183	-52.8211
ZINC22455022	-45.1867	-52.8175
ZINC22455022	-43.4624	-52.8175
ZINC22455022	-43.1032	-52.8175
ZINC22455022	-42.1759	-52.8175
ZINC00702974	-49.6227	-52.7968
ZINC00702974	-46.9868	-52.7968
ZINC08397075	-47.5059	-52.7945
ZINC08397075	-46.5285	-52.7945
ZINC08397075	-46.0005	-52.7945
ZINC08429983	-51.3359	-52.7710
ZINC08429983	-49.6482	-52.7710
ZINC08429983	-48.9233	-52.7710
ZINC19832136	-41.3005	-52.7473
ZINC19832136	-38.9479	-52.7473
ZINC08398380	-49.5653	-52.7327
ZINC04067618	-49.7187	-52.7234
ZINC04067618	-47.6812	-52.7234
ZINC06162053	-50.8699	-52.7187
ZINC00702555	-51.5026	-52.7081
ZINC08440788	-45.5953	-52.7044
ZINC08441775	-48.0823	-52.7023
ZINC19782009	-41.1049	-52.6834
ZINC19782009	-37.9616	-52.6834
ZINC04068105	-46.0126	-52.6777
ZINC32616969	-44.7846	-52.6653
ZINC08396627	-48.8961	-52.6620
ZINC36011813	-48.7211	-52.6612
ZINC08441656	-47.6156	-52.6573
ZINC08440547	-48.8670	-52.6558
ZINC06197100	-46.6149	-52.6523
ZINC19893170	-46.4375	-52.6455
ZINC19893170	-44.4944	-52.6455
ZINC08383414	-49.3699	-52.6379
ZINC19802479	-43.9834	-52.6374
ZINC19802479	-39.3440	-52.6374
ZINC08437891	-46.8042	-52.6281
ZINC17166889	-45.2658	-52.6141
ZINC08425703	-49.5283	-52.6084
ZINC06195515	-48.6697	-52.5931
ZINC06195515	-40.8052	-52.5931
ZINC08411637	-50.1674	-52.5888
ZINC08411637	-43.0509	-52.5888
ZINC08441137	-50.5170	-52.5834

ZINC08742453	-47.9250	-52.5721
ZINC08387147	-49.3927	-52.5212
ZINC06196064	-45.6783	-52.5146
ZINC08433389	-50.3891	-52.5059
ZINC08426809	-44.2259	-52.4953
ZINC08426809	-40.9225	-52.4953
ZINC08425691	-48.6900	-52.4773
ZINC19369757	-41.5096	-52.4767
ZINC19369757	-38.3434	-52.4767
ZINC09272792	-46.5275	-52.4733
ZINC09272792	-44.5465	-52.4733
ZINC09008309	-47.4521	-52.4703
ZINC09008309	-43.9256	-52.4703
ZINC08439609	-47.6765	-52.4548
ZINC08441050	-46.2801	-52.4358
ZINC08399640	-45.3409	-52.4278
ZINC08399640	-44.9337	-52.4278
ZINC08383967	-49.6802	-52.4200
ZINC34953308	-50.7179	-52.4050
ZINC06137488	-46.2608	-52.4005
ZINC08439501	-49.9736	-52.3911
ZINC08429883	-47.4158	-52.3887
ZINC04066064	-46.9929	-52.3619
ZINC04066064	-45.2896	-52.3619
ZINC08439277	-49.1542	-52.3330
ZINC08439277	-48.5805	-52.3330
ZINC19989832	-40.4683	-52.3101
ZINC19781717	-42.1051	-52.3070
ZINC04112128	-49.3496	-52.2852
ZINC04112128	-47.4285	-52.2852
ZINC04112194	-45.8296	-52.2718
ZINC04112194	-43.7816	-52.2718
ZINC08438750	-48.4077	-52.2635
ZINC03990180	-49.6076	-52.2593
ZINC03990180	-46.6750	-52.2593
ZINC03990180	-43.3964	-52.2593
ZINC08439523	-55.6326	-52.2542
ZINC08439523	-49.7500	-52.2542
ZINC22935456	-47.5508	-52.2508
ZINC19922745	-49.9513	-52.2476
ZINC08384134	-45.5993	-52.1996
ZINC08384134	-44.5928	-52.1996
ZINC08442191	-46.5177	-52.1976
ZINC00703085	-49.5686	-52.1746
ZINC00703085	-45.5272	-52.1746
ZINC00702942	-49.8940	-52.1621
ZINC09358334	-49.3284	-52.1529
ZINC09358334	-45.0177	-52.1529
ZINC09358334	-44.6460	-52.1529
ZINC09358334	-42.6444	-52.1529
ZINC18077935	-49.5213	-52.1473
ZINC00703121	-47.4297	-52.1174
ZINC08439495	-50.4402	-52.1142
ZINC08439495	-50.2175	-52.1142

ZINC09419395	-53.7049	-52.1103
ZINC06148759	-50.1675	-52.1050
ZINC19360259	-43.5193	-52.1024
ZINC19360259	-40.6487	-52.1024
ZINC19369770	-41.4784	-52.0971
ZINC19369770	-41.2596	-52.0971
ZINC08440104	-46.9605	-52.0925
ZINC08441202	-46.0562	-52.0810
ZINC08441202	-45.2890	-52.0810
ZINC19840241	-46.4848	-52.0599
ZINC19840241	-44.0688	-52.0599
ZINC08856305	-48.4353	-52.0521
ZINC08856305	-48.2729	-52.0521
ZINC00854092	-47.0667	-52.0420
ZINC00854092	-44.5663	-52.0420
ZINC04066095	-46.3511	-52.0317
ZINC08441490	-45.7669	-52.0311
ZINC08441490	-44.8162	-52.0311
ZINC08387165	-51.2302	-52.0279
ZINC08383621	-49.3646	-52.0133
ZINC16944268	-49.7061	-52.0094
ZINC08441218	-50.4866	-52.0023
ZINC08439520	-54.6626	-52.0019
ZINC19316052	-47.1749	-51.9908
ZINC19316052	-45.5380	-51.9908
ZINC04489061	-49.2433	-51.9842
ZINC08441242	-45.7398	-51.9804
ZINC06162025	-47.2569	-51.9754
ZINC06196100	-47.1751	-51.9583
ZINC08425685	-48.3964	-51.9557
ZINC08903739	-45.1051	-51.9537
ZINC19565349	-40.8203	-51.9528
ZINC19565349	-36.5585	-51.9528
ZINC08386169	-52.3251	-51.9508
ZINC00788084	-49.1304	-51.9501
ZINC08441278	-48.0800	-51.9406
ZINC00703111	-47.0273	-51.9326
ZINC08439442	-47.2428	-51.9326
ZINC08440108	-48.9289	-51.9277
ZINC08441534	-49.6709	-51.9230
ZINC08441534	-45.8556	-51.9230
ZINC22075045	-47.2206	-51.9197
ZINC08439473	-49.5886	-51.9184
ZINC08439473	-45.6049	-51.9184
ZINC19370899	-43.1293	-51.9123
ZINC19370899	-41.0938	-51.9123
ZINC08383334	-51.9263	-51.9059
ZINC08385360	-48.7132	-51.9049
ZINC15905827	-48.6666	-51.9008
ZINC02183391	-44.0672	-51.9008
ZINC06195387	-48.7099	-51.8994
ZINC06195387	-45.1455	-51.8994
ZINC08385363	-49.7675	-51.8924
ZINC06197322	-46.4575	-51.8812

ZINC06197322	-42.7700	-51.8812
ZINC00702586	-48.1423	-51.8595
ZINC00702586	-46.9522	-51.8595
ZINC22790361	-44.1793	-51.8556
ZINC22790361	-41.5866	-51.8556
ZINC08383443	-49.0747	-51.8478
ZINC19369765	-42.8720	-51.8427
ZINC19369765	-38.5074	-51.8427
ZINC09242603	-47.4237	-51.8378
ZINC19781611	-40.7410	-51.8196
ZINC19781611	-37.9088	-51.8196
ZINC08399198	-48.6430	-51.8123
ZINC19832104	-40.4245	-51.8081
ZINC19781672	-40.7416	-51.8080
ZINC19781672	-38.7468	-51.8080
ZINC08383425	-49.7453	-51.8019
ZINC08439541	-50.1137	-51.7973
ZINC08439541	-47.4232	-51.7973
ZINC08439541	-45.1077	-51.7973
ZINC08437317	-46.6761	-51.7961
ZINC04066030	-45.2218	-51.7925
ZINC04066030	-45.1182	-51.7925
ZINC04061246	-46.7016	-51.7865
ZINC04061246	-44.5444	-51.7865
ZINC08440907	-48.4156	-51.7699
ZINC05063676	-46.0861	-51.7649
ZINC08437307	-48.4751	-51.7624
ZINC08437307	-45.6617	-51.7624
ZINC08437307	-44.9123	-51.7624
ZINC08439612	-46.9594	-51.7559
ZINC00703131	-44.9752	-51.7532
ZINC08384044	-47.4966	-51.7516
ZINC04066411	-45.8867	-51.7492
ZINC19369774	-42.6894	-51.7461
ZINC19369774	-40.1038	-51.7461
ZINC04067848	-46.9167	-51.7311
ZINC04067381	-49.9766	-51.7307
ZINC04067381	-46.1722	-51.7307
ZINC04067381	-45.4050	-51.7307
ZINC04067770	-45.1766	-51.7252
ZINC08441542	-44.2021	-51.7204
ZINC17166748	-44.2783	-51.7080
ZINC17166748	-40.9343	-51.7080
ZINC17166748	-37.1905	-51.7080
ZINC08439443	-52.1326	-51.6999
ZINC08439443	-49.8534	-51.6999
ZINC08441732	-45.9858	-51.6829
ZINC08440053	-46.8625	-51.6588
ZINC08440053	-42.6315	-51.6588
ZINC08440053	-41.9243	-51.6588
ZINC08383835	-47.8047	-51.6379
ZINC08383835	-46.3000	-51.6379
ZINC06162502	-48.5734	-51.6248
ZINC08437285	-46.4600	-51.6238

ZINC08437285	-44.5901	-51.6238
ZINC02183627	-43.6803	-51.6228
ZINC22935454	-48.5943	-51.6184
ZINC08425699	-49.7873	-51.6128
ZINC04067429	-48.7135	-51.5984
ZINC04067429	-48.0371	-51.5984
ZINC04067374	-45.2598	-51.5884
ZINC04067866	-47.9692	-51.5822
ZINC04067866	-44.5659	-51.5822
ZINC04067866	-37.8883	-51.5822
ZINC08386152	-48.1777	-51.5813
ZINC04067855	-46.0775	-51.5680
ZINC08439542	-51.5776	-51.5372
ZINC08439542	-48.8541	-51.5372
ZINC08439542	-47.9284	-51.5372
ZINC04417587	-49.1932	-51.5174
ZINC11565510	-46.7422	-51.5165
ZINC11565510	-44.3051	-51.5165
ZINC11565510	-43.9294	-51.5165
ZINC11565510	-43.7096	-51.5165
ZINC00659305	-49.1733	-51.5157
ZINC32616946	-43.8790	-51.5022
ZINC08441514	-47.4622	-51.4988
ZINC08433350	-50.2966	-51.4973
ZINC08441270	-48.8910	-51.4857
ZINC19900184	-42.2173	-51.4793
ZINC19900184	-42.0775	-51.4793
ZINC08441345	-43.1274	-51.4601
ZINC08441345	-37.5717	-51.4601
ZINC08435026	-47.6963	-51.4591
ZINC08441287	-49.4330	-51.4526
ZINC08441287	-48.4840	-51.4526
ZINC08435523	-48.1361	-51.4359
ZINC08411274	-47.5489	-51.4309
ZINC08441340	-47.5595	-51.4196
ZINC01155301	-47.1961	-51.4165
ZINC18154321	-52.4496	-51.4006
ZINC18154321	-52.0423	-51.4006
ZINC18154321	-49.1254	-51.4006
ZINC06144027	-47.0930	-51.3916
ZINC08441811	-43.9034	-51.3819
ZINC08433279	-48.0189	-51.3818
ZINC06197655	-44.1074	-51.3745
ZINC05918675	-46.8343	-51.3674
ZINC05918675	-45.7676	-51.3674
ZINC09123616	-47.5902	-51.3611
ZINC04067731	-49.3196	-51.3539
ZINC04067731	-43.2612	-51.3539
ZINC13301864	-48.6113	-51.3368
ZINC08441434	-44.0958	-51.3363
ZINC08441434	-37.1774	-51.3363
ZINC09271557	-45.6898	-51.3292
ZINC09271557	-45.6267	-51.3292
ZINC00702556	-48.3412	-51.3283

ZINC08440649	-47.5753	-51.3046
ZINC08385297	-50.7262	-51.2828
ZINC09244208	-50.7321	-51.2776
ZINC09244208	-50.7223	-51.2776
ZINC09244208	-48.2759	-51.2776
ZINC04067897	-45.2142	-51.2732
ZINC04067897	-44.1727	-51.2732
ZINC04067897	-42.0323	-51.2732
ZINC17057162	-54.3118	-51.2688
ZINC30952358	-49.2934	-51.2625
ZINC08399176	-44.7718	-51.2614
ZINC08399176	-43.7277	-51.2614
ZINC08399176	-42.2851	-51.2614
ZINC08383695	-48.0223	-51.2596
ZINC00729046	-44.9963	-51.2351
ZINC08443061	-46.4613	-51.2238
ZINC08437286	-50.4892	-51.2161
ZINC08437286	-49.2854	-51.2161
ZINC08437286	-48.9460	-51.2161
ZINC19287393	-48.6243	-51.2147
ZINC19922814	-43.7733	-51.2025
ZINC15895155	-48.1011	-51.1998
ZINC15895155	-45.2731	-51.1998
ZINC08440751	-46.3933	-51.1943
ZINC04067199	-47.0505	-51.1794
ZINC04067199	-46.4593	-51.1794
ZINC04067199	-45.6509	-51.1794
ZINC08416137	-46.0810	-51.1715
ZINC08416137	-44.0739	-51.1715
ZINC04547568	-45.5456	-51.1516
ZINC32617181	-46.0165	-51.1397
ZINC08383782	-47.3645	-51.1240
ZINC09009198	-45.5610	-51.1202
ZINC09009198	-43.6528	-51.1202
ZINC19624668	-40.0290	-51.1129
ZINC19624668	-38.4759	-51.1129
ZINC08441677	-44.7682	-51.1098
ZINC08441677	-42.6704	-51.1098
ZINC08383598	-48.3082	-51.1013
ZINC19781923	-41.8185	-51.0899
ZINC19781923	-39.4211	-51.0899
ZINC06195475	-46.6019	-51.0839
ZINC08411403	-48.0692	-51.0772
ZINC08411403	-40.6272	-51.0772
ZINC04066558	-46.3330	-51.0713
ZINC02188291	-46.2019	-51.0674
ZINC06015551	-45.3918	-51.0664
ZINC23478159	-40.4678	-51.0661
ZINC23478159	-36.3627	-51.0661
ZINC06195505	-47.9996	-51.0548
ZINC36473810	-49.2641	-51.0435
ZINC08426357	-48.7291	-51.0435
ZINC05918609	-46.8793	-51.0382
ZINC05918609	-45.9210	-51.0382

ZINC04062016	-46.7459	-51.0200
ZINC04062016	-44.0766	-51.0200
ZINC04062016	-43.8142	-51.0200
ZINC08443409	-45.6234	-51.0163
ZINC08443409	-44.1537	-51.0163
ZINC08433278	-49.3863	-51.0008
ZINC17124565	-51.5972	-50.9979
ZINC08433388	-48.3734	-50.9940
ZINC08415258	-46.7559	-50.9915
ZINC08415258	-43.9259	-50.9915
ZINC85425826	-47.4928	-50.9744
ZINC85425826	-46.3181	-50.9744
ZINC19832138	-40.2530	-50.9547
ZINC19832138	-36.9942	-50.9547
ZINC00679104	-46.8230	-50.9484
ZINC08441595	-45.8935	-50.9464
ZINC08441595	-43.2461	-50.9464
ZINC08441025	-47.3637	-50.9436
ZINC08441025	-42.8251	-50.9436
ZINC00715947	-48.5687	-50.9384
ZINC00715947	-42.3064	-50.9384
ZINC00715947	-41.2237	-50.9384
ZINC08425687	-46.7125	-50.9381
ZINC04629663	-43.5160	-50.9367
ZINC04629663	-43.1381	-50.9367
ZINC08440213	-44.7827	-50.9341
ZINC08440213	-43.8896	-50.9341
ZINC01019963	-47.3899	-50.9340
ZINC09185693	-45.6292	-50.9323
ZINC00626241	-47.6043	-50.9234
ZINC00626241	-47.1994	-50.9234
ZINC08415249	-45.1889	-50.9134
ZINC08415249	-44.4234	-50.9134
ZINC05408089	-50.1369	-50.9130
ZINC08437426	-44.0180	-50.9114
ZINC22790008	-52.9147	-50.8943
ZINC22790008	-51.4226	-50.8943
ZINC22790008	-49.3905	-50.8943
ZINC22790008	-49.1689	-50.8943
ZINC22790008	-47.8454	-50.8943
ZINC18249716	-47.4937	-50.8925
ZINC19880692	-42.3661	-50.8843
ZINC05895015	-48.1709	-50.8763
ZINC08439471	-47.3635	-50.8725
ZINC08439471	-45.7596	-50.8725
ZINC15905826	-46.6617	-50.8677
ZINC15905826	-45.9978	-50.8677
ZINC04066412	-47.5422	-50.8509
ZINC04061865	-44.8812	-50.8475
ZINC04061865	-44.7698	-50.8475
ZINC04061865	-42.4115	-50.8475
ZINC06195437	-44.6868	-50.8458
ZINC06195437	-43.4730	-50.8458
ZINC08383327	-48.7883	-50.8438

ZINC06162501	-43.7229	-50.8322
ZINC19922787	-44.1587	-50.8295
ZINC04067664	-45.6330	-50.8257
ZINC08441504	-45.8100	-50.8165
ZINC08441504	-43.9220	-50.8165
ZINC08424359	-44.2206	-50.8078
ZINC08424359	-39.5681	-50.8078
ZINC08424359	-39.5558	-50.8078
ZINC04067856	-48.3662	-50.8041
ZINC04062204	-44.2568	-50.8016
ZINC04062204	-43.6402	-50.8016
ZINC02186457	-45.6166	-50.7972
ZINC00727918	-55.3835	-50.7965
ZINC08996627	-46.0619	-50.7894
ZINC05921188	-46.2017	-50.7845
ZINC00726452	-44.7735	-50.7831
ZINC08817379	-44.3917	-50.7828
ZINC04065694	-45.6885	-50.7807
ZINC04065694	-44.4962	-50.7807
ZINC08396635	-50.8359	-50.7768
ZINC19360224	-40.5760	-50.7739
ZINC19360224	-40.4460	-50.7739
ZINC08426359	-49.0461	-50.7725
ZINC19866300	-46.5660	-50.7508
ZINC04632279	-44.2167	-50.7474
ZINC06015315	-46.3451	-50.7308
ZINC06015315	-46.2490	-50.7308
ZINC06015315	-44.6451	-50.7308
ZINC19369763	-42.8150	-50.7299
ZINC19369763	-41.2824	-50.7299
ZINC08439486	-53.6140	-50.7243
ZINC08397400	-45.2864	-50.7195
ZINC08397400	-42.1061	-50.7195
ZINC19781684	-41.9609	-50.7157
ZINC19781684	-40.0063	-50.7157
ZINC08444357	-46.6111	-50.7073
ZINC08444357	-45.8349	-50.7073
ZINC04068031	-45.0434	-50.6872
ZINC04068031	-43.4904	-50.6872
ZINC04068031	-43.4782	-50.6872
ZINC06196104	-49.3852	-50.6784
ZINC19874799	-43.6369	-50.6770
ZINC04067957	-44.2928	-50.6596
ZINC04067957	-43.4723	-50.6596
ZINC04067957	-42.9377	-50.6596
ZINC08425686	-47.4927	-50.6522
ZINC06196042	-46.2203	-50.6503
ZINC06161967	-49.4857	-50.6503
ZINC06161967	-41.7481	-50.6503
ZINC08441059	-45.1268	-50.6343
ZINC02081645	-43.6902	-50.6326
ZINC00702743	-44.5672	-50.6224
ZINC00702743	-41.7990	-50.6224
ZINC06195592	-46.2371	-50.6098

ZINC06195592	-40.0156	-50.6098
ZINC04067663	-46.5621	-50.6073
ZINC00707933	-46.4252	-50.6054
ZINC00707933	-45.7586	-50.6054
ZINC00707933	-44.0391	-50.6054
ZINC85397625	-44.9015	-50.6051
ZINC08441296	-47.5983	-50.5942
ZINC08441296	-46.8555	-50.5942
ZINC08441296	-46.2375	-50.5942
ZINC06162321	-48.0449	-50.5938
ZINC06162321	-43.7626	-50.5938
ZINC09373975	-48.9422	-50.5891
ZINC09373975	-48.0440	-50.5891
ZINC08415254	-44.9768	-50.5866
ZINC04066332	-43.6868	-50.5686
ZINC08695187	-50.3736	-50.5614
ZINC08407237	-44.7440	-50.5611
ZINC01451981	-47.3416	-50.5410
ZINC01451981	-46.7167	-50.5410
ZINC09070616	-46.3003	-50.5401
ZINC09070616	-43.7527	-50.5401
ZINC00726467	-44.3037	-50.5387
ZINC08429974	-43.2198	-50.5220
ZINC08429974	-42.2567	-50.5220
ZINC08429974	-40.5504	-50.5220
ZINC00986479	-44.9110	-50.5116
ZINC08439468	-46.3569	-50.5097
ZINC08439468	-44.1343	-50.5097
ZINC08741003	-44.8529	-50.4961
ZINC19832120	-39.8241	-50.4807
ZINC02070496	-44.2988	-50.4701
ZINC06162109	-44.5637	-50.4686
ZINC08387161	-48.0626	-50.4680
ZINC08384131	-45.9359	-50.4679
ZINC08384131	-44.8312	-50.4679
ZINC08383735	-47.1006	-50.4639
ZINC08440793	-44.4945	-50.4615
ZINC04067729	-45.9040	-50.4575
ZINC04067729	-40.6998	-50.4575
ZINC04067729	-40.0137	-50.4575
ZINC08441697	-45.6656	-50.4522
ZINC08433346	-48.0932	-50.4480
ZINC08440571	-48.4274	-50.4375
ZINC06149861	-44.4529	-50.4350
ZINC19802471	-42.3080	-50.4317
ZINC19802471	-39.1741	-50.4317
ZINC04065354	-46.4528	-50.4263
ZINC04065354	-44.4701	-50.4263
ZINC04068030	-46.0066	-50.4222
ZINC04068030	-45.5214	-50.4222
ZINC04068030	-44.3655	-50.4222
ZINC19802475	-41.9832	-50.4220
ZINC19802475	-37.5470	-50.4220
ZINC19370890	-41.3323	-50.4157

ZINC19370890	-37.3852	-50.4157
ZINC06195517	-47.0505	-50.4123
ZINC06195517	-46.6592	-50.4123
ZINC13549197	-46.0495	-50.4061
ZINC18209674	-44.7550	-50.4034
ZINC18209674	-41.2854	-50.4034
ZINC08433361	-46.7999	-50.3976
ZINC08397071	-45.9315	-50.3940
ZINC08397071	-41.5446	-50.3940
ZINC19832129	-38.8238	-50.3919
ZINC08432403	-44.6524	-50.3917
ZINC08441756	-45.8919	-50.3842
ZINC00848044	-45.3746	-50.3812
ZINC06136930	-48.0520	-50.3809
ZINC05918729	-49.9019	-50.3772
ZINC08399145	-45.9695	-50.3673
ZINC06137205	-42.8882	-50.3643
ZINC08384740	-48.8650	-50.3620
ZINC08439481	-49.9369	-50.3509
ZINC08439481	-48.9440	-50.3509
ZINC08439481	-47.4658	-50.3509
ZINC13511515	-46.4298	-50.3463
ZINC08433362	-46.2363	-50.3367
ZINC04067959	-44.3763	-50.3363
ZINC04067959	-43.9550	-50.3363
ZINC08444347	-43.4608	-50.3262
ZINC08444347	-42.4868	-50.3262
ZINC06197240	-45.2103	-50.3221
ZINC06197240	-42.7505	-50.3221
ZINC13125591	-49.5966	-50.3189
ZINC19565337	-41.9582	-50.3123
ZINC19565337	-41.7945	-50.3123
ZINC06161966	-48.8052	-50.3056
ZINC09244209	-46.7586	-50.2981
ZINC09244209	-43.7938	-50.2981
ZINC08385743	-50.5245	-50.2896
ZINC06195553	-46.5899	-50.2818
ZINC06195553	-44.9157	-50.2818
ZINC08441617	-44.8850	-50.2768
ZINC08384254	-48.9992	-50.2759
ZINC06197203	-45.3627	-50.2668
ZINC06197203	-36.6245	-50.2668
ZINC00675212	-45.7704	-50.2667
ZINC00675212	-44.2500	-50.2667
ZINC85397614	-48.4775	-50.2635
ZINC08383614	-47.1391	-50.2634
ZINC04905405	-43.7845	-50.2617
ZINC19561088	-38.1692	-50.2487
ZINC00848043	-45.9175	-50.2444
ZINC00848043	-42.9967	-50.2444
ZINC08441480	-42.7617	-50.2318
ZINC08441480	-42.1637	-50.2318
ZINC06195473	-47.7818	-50.2291
ZINC06196393	-47.8877	-50.2275

ZINC85428083	-44.5224	-50.2251
ZINC17028917	-45.6616	-50.2187
ZINC08695215	-48.8410	-50.2095
ZINC08441601	-44.8874	-50.2082
ZINC08441601	-44.6912	-50.2082
ZINC08637015	-46.2192	-50.1991
ZINC00859158	-49.2960	-50.1923
ZINC08439440	-47.1245	-50.1872
ZINC08439440	-44.2872	-50.1872
ZINC19879890	-47.4372	-50.1863
ZINC19879890	-43.2834	-50.1863
ZINC04391410	-42.3529	-50.1739
ZINC04391410	-42.0626	-50.1739
ZINC08440042	-45.8639	-50.1726
ZINC08440042	-41.3911	-50.1726
ZINC08440042	-40.0775	-50.1726
ZINC08695219	-49.2167	-50.1659
ZINC08441724	-45.5432	-50.1562
ZINC08440545	-47.0315	-50.1559
ZINC00649312	-47.1616	-50.1357
ZINC06300107	-44.2507	-50.1347
ZINC20027304	-41.0138	-50.1337
ZINC20027304	-40.4179	-50.1337
ZINC00702937	-47.7214	-50.1274
ZINC06197197	-46.2058	-50.1190
ZINC08441686	-48.4658	-50.1093
ZINC04066245	-43.3633	-50.0943
ZINC04066245	-40.5386	-50.0943
ZINC04066245	-40.2615	-50.0943
ZINC08425701	-49.7575	-50.0926
ZINC08384750	-47.0817	-50.0837
ZINC08429953	-46.0454	-50.0777
ZINC06137342	-44.9979	-50.0705
ZINC08914540	-45.3323	-50.0686
ZINC08441543	-47.5854	-50.0675
ZINC19832118	-41.4883	-50.0673
ZINC19832118	-39.8889	-50.0673
ZINC19781838	-40.1783	-50.0502
ZINC19781838	-39.6649	-50.0502
ZINC02135508	-49.2875	-50.0325
ZINC08441638	-45.8757	-50.0271
ZINC08439469	-47.9254	-50.0139
ZINC08439469	-47.4917	-50.0139
ZINC08416328	-41.7101	-50.0031
ZINC02183639	-40.3923	-50.0017
ZINC08433209	-44.4004	-49.9941
ZINC08433209	-43.1703	-49.9941
ZINC08385742	-49.2191	-49.9940
ZINC08426358	-47.6735	-49.9903
ZINC19730674	-47.2817	-49.9875
ZINC08413287	-41.3665	-49.9779
ZINC08441077	-44.7375	-49.9725
ZINC08441838	-41.7711	-49.9551
ZINC06197199	-43.2909	-49.9503

ZINC08383378	-46.8205	-49.9438
ZINC19370372	-42.1935	-49.9410
ZINC19370372	-41.6476	-49.9410
ZINC08440126	-47.2422	-49.9399
ZINC08440126	-46.0428	-49.9399
ZINC08384036	-45.7587	-49.9385
ZINC06136922	-47.0815	-49.9367
ZINC06136922	-42.5814	-49.9367
ZINC06015260	-49.8879	-49.9262
ZINC08439855	-44.5668	-49.9247
ZINC08439855	-42.7635	-49.9247
ZINC08425689	-47.7045	-49.9218
ZINC04112599	-49.7097	-49.9151
ZINC04112599	-45.8795	-49.9151
ZINC08425688	-48.2115	-49.9145
ZINC00703042	-46.4053	-49.9046
ZINC00703042	-41.1792	-49.9046
ZINC08441508	-40.8560	-49.9028
ZINC08997041	-45.0701	-49.8897
ZINC08442207	-43.3035	-49.8839
ZINC02135563	-48.8036	-49.8798
ZINC06197510	-44.8615	-49.8794
ZINC19334219	-39.5028	-49.8768
ZINC08440518	-49.1103	-49.8745
ZINC06196079	-46.4567	-49.8693
ZINC04067633	-45.3272	-49.8671
ZINC04067633	-45.1229	-49.8671
ZINC04067633	-44.0889	-49.8671
ZINC04067393	-44.9361	-49.8660
ZINC04067393	-44.0010	-49.8660
ZINC04067043	-44.3701	-49.8587
ZINC04067043	-42.0566	-49.8587
ZINC04067043	-39.7211	-49.8587
ZINC08440044	-45.5888	-49.8573
ZINC08440044	-44.4468	-49.8573
ZINC08440044	-40.7842	-49.8573
ZINC31936770	-42.4740	-49.8573
ZINC19850932	-51.5118	-49.8559
ZINC06162391	-46.3566	-49.8530
ZINC19781727	-40.2952	-49.8466
ZINC19781727	-37.4111	-49.8466
ZINC08441344	-48.1507	-49.8436
ZINC08433414	-46.2203	-49.8414
ZINC08396594	-47.6780	-49.8397
ZINC08415568	-45.3779	-49.8316
ZINC08387281	-50.2686	-49.8312
ZINC02183390	-42.5557	-49.8293
ZINC19360193	-39.5674	-49.8284
ZINC08400605	-47.6046	-49.8197
ZINC08440645	-44.0272	-49.8100
ZINC08397170	-43.7453	-49.8093
ZINC08397170	-41.7018	-49.8093
ZINC08397170	-41.6566	-49.8093
ZINC08411367	-47.8471	-49.8061

ZINC08411367	-46.2884	-49.8061
ZINC08411367	-45.7322	-49.8061
ZINC18046209	-47.6383	-49.8061
ZINC18046209	-46.2259	-49.8061
ZINC18046209	-44.6523	-49.8061
ZINC08695247	-50.2429	-49.8029
ZINC04068106	-44.0605	-49.7965
ZINC19365327	-40.8852	-49.7960
ZINC19365327	-39.0842	-49.7960
ZINC08411382	-45.5061	-49.7911
ZINC06194160	-42.7170	-49.7895
ZINC06194160	-41.4979	-49.7895
ZINC08439680	-46.6055	-49.7829
ZINC08440848	-44.7529	-49.7825
ZINC06995628	-47.8429	-49.7708
ZINC08433248	-43.8509	-49.7704
ZINC19938591	-43.2221	-49.7573
ZINC00703130	-46.6400	-49.7551
ZINC08441188	-46.2727	-49.7547
ZINC19781696	-39.7888	-49.7473
ZINC06137204	-44.0588	-49.7408
ZINC09008467	-49.2556	-49.7382
ZINC09008467	-45.4623	-49.7382
ZINC09008467	-42.0695	-49.7382
ZINC06407376	-44.6808	-49.7340
ZINC08385361	-50.8457	-49.7221
ZINC17440252	-50.5452	-49.7186
ZINC08444362	-43.9626	-49.7160
ZINC08444362	-41.6516	-49.7160
ZINC19367876	-39.3329	-49.7154
ZINC19367876	-38.6989	-49.7154
ZINC19549848	-41.2362	-49.7152
ZINC08439498	-51.3452	-49.7122
ZINC04068139	-42.7796	-49.7013
ZINC08398997	-43.7621	-49.7006
ZINC08442058	-45.7175	-49.6975
ZINC08442058	-44.5327	-49.6975
ZINC08441483	-46.1351	-49.6886
ZINC08441483	-44.9466	-49.6886
ZINC04064852	-45.6225	-49.6819
ZINC04064852	-44.0892	-49.6819
ZINC04064852	-43.3221	-49.6819
ZINC04003967	-42.5415	-49.6699
ZINC08427630	-47.2295	-49.6621
ZINC08427630	-44.6796	-49.6621
ZINC06195423	-44.6267	-49.6511
ZINC06195423	-42.7899	-49.6511
ZINC08451662	-51.0923	-49.6511
ZINC06137207	-44.1727	-49.6482
ZINC04068124	-44.2446	-49.6457
ZINC04068124	-44.0625	-49.6457
ZINC08439669	-45.2608	-49.6439
ZINC08441998	-47.9840	-49.6439
ZINC08441998	-46.5072	-49.6439

ZINC08400636	-46.4077	-49.6401
ZINC08433385	-49.9938	-49.6374
ZINC06137654	-47.2425	-49.6313
ZINC09271668	-49.9335	-49.6300
ZINC00702931	-45.9027	-49.6278
ZINC20264069	-49.0350	-49.6189
ZINC04391415	-42.6664	-49.6149
ZINC04391415	-42.0539	-49.6149
ZINC08440205	-44.2484	-49.6137
ZINC08440205	-43.2021	-49.6137
ZINC08440205	-42.9581	-49.6137
ZINC09065134	-43.6904	-49.6116
ZINC08441388	-41.4342	-49.5905
ZINC08441388	-38.7483	-49.5905
ZINC19781924	-42.8678	-49.5822
ZINC19781924	-40.5563	-49.5822
ZINC08695220	-46.7348	-49.5794
ZINC04067993	-45.1708	-49.5773
ZINC04067993	-44.0889	-49.5773
ZINC04067993	-39.6466	-49.5773
ZINC08440589	-51.9735	-49.5763
ZINC08437414	-43.4862	-49.5761
ZINC17154914	-46.0632	-49.5752
ZINC04079997	-47.3239	-49.5749
ZINC08915059	-45.8653	-49.5629
ZINC08441175	-46.2946	-49.5615
ZINC08441175	-45.7784	-49.5615
ZINC20233039	-48.3929	-49.5605
ZINC20233039	-41.6597	-49.5605
ZINC20018594	-47.0700	-49.5563
ZINC04112197	-46.7471	-49.5426
ZINC05225812	-44.8036	-49.5369
ZINC19869027	-48.7154	-49.5349
ZINC08440259	-42.8966	-49.5304
ZINC19781576	-39.7647	-49.5300
ZINC19781576	-36.9304	-49.5300
ZINC08440118	-45.0005	-49.5273
ZINC06162113	-47.7312	-49.5218
ZINC17123459	-49.8349	-49.5204
ZINC09007505	-45.1161	-49.5199
ZINC08435389	-47.8819	-49.5141
ZINC08435389	-41.0997	-49.5141
ZINC08453805	-45.4357	-49.5108
ZINC19367073	-41.0115	-49.5067
ZINC19922843	-43.8936	-49.5020
ZINC08441372	-49.1620	-49.5004
ZINC04632284	-43.7129	-49.4915
ZINC04067259	-45.9189	-49.4894
ZINC04067259	-44.9876	-49.4894
ZINC06197272	-44.8964	-49.4873
ZINC19832111	-43.1469	-49.4799
ZINC19832111	-41.9873	-49.4799
ZINC08440055	-46.9707	-49.4724
ZINC08425690	-48.0767	-49.4706

ZINC19364633	-40.6880	-49.4596
ZINC19364633	-37.2508	-49.4596
ZINC15134446	-43.3740	-49.4548
ZINC08441312	-48.3109	-49.4540
ZINC09357684	-43.8453	-49.4535
ZINC09357684	-42.7247	-49.4535
ZINC00702738	-45.3936	-49.4477
ZINC00702738	-43.8052	-49.4477
ZINC08410379	-48.0826	-49.4458
ZINC08441539	-44.5634	-49.4403
ZINC08441539	-44.5149	-49.4403
ZINC04112282	-43.5764	-49.4399
ZINC04112282	-42.3319	-49.4399
ZINC04112282	-41.5925	-49.4399
ZINC15767577	-47.4783	-49.4355
ZINC19815797	-41.5376	-49.4264
ZINC19815797	-39.9460	-49.4264
ZINC08441668	-47.8570	-49.4203
ZINC08440149	-49.1681	-49.4189
ZINC08440149	-43.0033	-49.4189
ZINC04112111	-45.0246	-49.4154
ZINC09360903	-45.9715	-49.4149
ZINC09360903	-43.5524	-49.4149
ZINC16225716	-45.6184	-49.4097
ZINC22933524	-45.1745	-49.4085
ZINC08439667	-46.9805	-49.4023
ZINC08439667	-44.3107	-49.4023
ZINC08440760	-43.7228	-49.3948
ZINC08440760	-42.6948	-49.3948
ZINC08440760	-40.4456	-49.3948
ZINC18094203	-46.7552	-49.3907
ZINC09339496	-44.3441	-49.3894
ZINC09339496	-44.0652	-49.3894
ZINC06136863	-42.3155	-49.3869
ZINC00726672	-45.0632	-49.3836
ZINC00726672	-40.2656	-49.3836
ZINC00726672	-39.7469	-49.3836
ZINC08386162	-48.9763	-49.3764
ZINC08441709	-45.6790	-49.3719
ZINC20031951	-42.0141	-49.3699
ZINC05313297	-48.1919	-49.3693
ZINC02861191	-44.0491	-49.3559
ZINC08440114	-43.8303	-49.3427
ZINC08817359	-44.5308	-49.3401
ZINC19370888	-38.7268	-49.3242
ZINC19370888	-37.7561	-49.3242
ZINC06995653	-50.2470	-49.3196
ZINC00703153	-46.4148	-49.3129
ZINC06015311	-44.8934	-49.3084
ZINC06015311	-43.5358	-49.3084
ZINC01019727	-45.3318	-49.3078
ZINC08427631	-46.2414	-49.2991
ZINC08427631	-42.7106	-49.2991
ZINC08437251	-47.2723	-49.2956

ZINC00702934	-44.7667	-49.2889
ZINC08441723	-46.8442	-49.2863
ZINC06196101	-46.8594	-49.2821
ZINC08397040	-43.6283	-49.2776
ZINC08397040	-43.5214	-49.2776
ZINC08441193	-46.6326	-49.2723
ZINC08695209	-48.6191	-49.2701
ZINC00702740	-45.5659	-49.2697
ZINC08444502	-50.1117	-49.2668
ZINC04067895	-45.6600	-49.2664
ZINC04067895	-42.2151	-49.2664
ZINC17246565	-44.2888	-49.2638
ZINC17246565	-42.5234	-49.2638
ZINC04065938	-41.4536	-49.2583
ZINC06195958	-43.5720	-49.2544
ZINC09110088	-47.1472	-49.2537
ZINC08450281	-44.6060	-49.2522
ZINC08450281	-44.0835	-49.2522
ZINC22937062	-47.1563	-49.2514
ZINC08396631	-48.3089	-49.2500
ZINC08385358	-48.0530	-49.2494
ZINC08441616	-45.5481	-49.2490
ZINC08441616	-41.3911	-49.2490
ZINC09463371	-43.2345	-49.2476
ZINC09463371	-40.9588	-49.2476
ZINC06148679	-47.8740	-49.2444
ZINC06148679	-43.0512	-49.2444
ZINC08439859	-42.7611	-49.2364
ZINC08440718	-44.8677	-49.2342
ZINC08383646	-46.9168	-49.2262
ZINC08433243	-45.0010	-49.2221
ZINC08433243	-44.3869	-49.2221
ZINC08433243	-42.9435	-49.2221
ZINC02088183	-41.6351	-49.2175
ZINC02088183	-38.8647	-49.2175
ZINC19859204	-39.5085	-49.2126
ZINC01413336	-44.6932	-49.2043
ZINC08424586	-46.2033	-49.1930
ZINC08424586	-45.3008	-49.1930
ZINC08424586	-44.3533	-49.1930
ZINC06162092	-46.3239	-49.1914
ZINC12523687	-45.1486	-49.1854
ZINC13630063	-44.8164	-49.1837
ZINC04068148	-44.3957	-49.1830
ZINC04061548	-43.3354	-49.1829
ZINC04061548	-42.9682	-49.1829
ZINC04061548	-40.9380	-49.1829
ZINC37197664	-43.2910	-49.1737
ZINC08384621	-46.8410	-49.1705
ZINC08383914	-46.6746	-49.1679
ZINC08383914	-42.0097	-49.1679
ZINC08400607	-48.4345	-49.1633
ZINC08400607	-46.3684	-49.1633
ZINC00726539	-42.0742	-49.1617

ZINC08441036	-44.7145	-49.1591
ZINC08441036	-43.1080	-49.1591
ZINC08441036	-42.1016	-49.1591
ZINC00844233	-42.8137	-49.1566
ZINC00844233	-39.7034	-49.1566
ZINC04780830	-41.1361	-49.1538
ZINC04780830	-36.6848	-49.1538
ZINC19938296	-48.2631	-49.1481
ZINC00703044	-45.0569	-49.1446
ZINC00703044	-44.6575	-49.1446
ZINC19938585	-42.4563	-49.1434
ZINC04067730	-43.1360	-49.1331
ZINC04067730	-40.9444	-49.1331
ZINC08400344	-44.0515	-49.1324
ZINC08400652	-47.8974	-49.1320
ZINC00702575	-47.1791	-49.1319
ZINC08415550	-45.8556	-49.1230
ZINC13552517	-47.0045	-49.1115
ZINC08439755	-45.4765	-49.1098
ZINC08439755	-45.2102	-49.1098
ZINC04067995	-45.2363	-49.1072
ZINC19938590	-44.8755	-49.1025
ZINC06136979	-44.6804	-49.0965
ZINC19871839	-44.1723	-49.0951
ZINC08440789	-43.4221	-49.0921
ZINC08438670	-45.2677	-49.0886
ZINC08438670	-43.0288	-49.0886
ZINC00987526	-46.9802	-49.0825
ZINC00987526	-44.8868	-49.0825
ZINC00987526	-44.6785	-49.0825
ZINC08441671	-47.6498	-49.0799
ZINC13118813	-43.0539	-49.0549
ZINC02757621	-49.9402	-49.0511
ZINC05296296	-44.1495	-49.0500
ZINC05296296	-42.9198	-49.0500
ZINC05296296	-40.4038	-49.0500
ZINC08771332	-43.2282	-49.0461
ZINC04062338	-42.0281	-49.0442
ZINC04062338	-41.1895	-49.0442
ZINC00702686	-45.4209	-49.0425
ZINC00702686	-45.1048	-49.0425
ZINC04065479	-44.9329	-49.0393
ZINC04065479	-44.3532	-49.0393
ZINC04065479	-44.2067	-49.0393
ZINC09280995	-46.6032	-49.0358
ZINC09280995	-42.9004	-49.0358
ZINC06144551	-50.9316	-49.0353
ZINC08439467	-46.6804	-49.0319
ZINC08439467	-45.2895	-49.0319
ZINC08439467	-43.1500	-49.0319
ZINC20219828	-51.7852	-49.0318
ZINC08441499	-44.4394	-49.0271
ZINC08430009	-47.3998	-49.0266
ZINC06137469	-42.5788	-49.0230

ZINC71414405	-42.6942	-49.0200
ZINC01019957	-44.3400	-49.0124
ZINC09272735	-42.8738	-49.0068
ZINC09272735	-42.1728	-49.0068
ZINC08440976	-50.1144	-48.9944
ZINC08383541	-47.5855	-48.9934
ZINC08383541	-44.8205	-48.9934
ZINC08996628	-43.5015	-48.9836
ZINC04067430	-48.9693	-48.9821
ZINC04067430	-48.9387	-48.9821
ZINC04067430	-48.2544	-48.9821
ZINC08695213	-47.7684	-48.9800
ZINC04061928	-43.4053	-48.9739
ZINC08415269	-41.4111	-48.9677
ZINC08439480	-50.2738	-48.9614
ZINC08429912	-45.0574	-48.9566
ZINC08429912	-44.5951	-48.9566
ZINC08429912	-44.5045	-48.9566
ZINC00702747	-45.6090	-48.9539
ZINC00856906	-40.4359	-48.9524
ZINC08430270	-46.4738	-48.9507
ZINC08430270	-46.1614	-48.9507
ZINC08430270	-45.6683	-48.9507
ZINC08893127	-44.5738	-48.9467
ZINC06149830	-43.6157	-48.9390
ZINC06149830	-41.9542	-48.9390
ZINC38233064	-43.2091	-48.9283
ZINC01152937	-43.6156	-48.9240
ZINC04061924	-44.1512	-48.9186
ZINC08437412	-44.1095	-48.9169
ZINC00719680	-49.5304	-48.9134
ZINC20011089	-44.9532	-48.9087
ZINC20011089	-39.4796	-48.9087
ZINC08441699	-44.0121	-48.9054
ZINC06137115	-48.0660	-48.8997
ZINC06137115	-44.2628	-48.8997
ZINC06137115	-43.6934	-48.8997
ZINC04063538	-41.1060	-48.8927
ZINC13590325	-47.4247	-48.8923
ZINC02183635	-40.3528	-48.8906
ZINC02181885	-42.1979	-48.8877
ZINC17154943	-43.6379	-48.8853
ZINC17154943	-40.1419	-48.8853
ZINC16115616	-43.1174	-48.8816
ZINC16115616	-41.8475	-48.8816
ZINC16115616	-39.5942	-48.8816
ZINC08384040	-47.1429	-48.8776
ZINC04418295	-45.5549	-48.8751
ZINC04418295	-43.9752	-48.8751
ZINC08400415	-42.8714	-48.8743
ZINC19922781	-44.4338	-48.8738
ZINC08440794	-43.6303	-48.8690
ZINC04112129	-42.9145	-48.8601
ZINC04112129	-42.4758	-48.8601

ZINC08695241	-48.6408	-48.8544
ZINC08686432	-46.5122	-48.8542
ZINC06149873	-44.8559	-48.8434
ZINC06149873	-44.6209	-48.8434
ZINC00703087	-47.4887	-48.8417
ZINC06197216	-43.2588	-48.8368
ZINC00619794	-43.0526	-48.8277
ZINC08441738	-44.1837	-48.8249
ZINC02089024	-41.4504	-48.8215
ZINC19770785	-46.3943	-48.8207
ZINC08413351	-43.2728	-48.8185
ZINC08413351	-43.2270	-48.8185
ZINC08413351	-43.1275	-48.8185
ZINC08384348	-43.6959	-48.8180
ZINC08440845	-44.5642	-48.8154
ZINC09360068	-53.6934	-48.8151
ZINC09360068	-48.9869	-48.8151
ZINC09088349	-45.5541	-48.8029
ZINC00844236	-44.1383	-48.8005
ZINC00844236	-39.9965	-48.8005
ZINC08425700	-47.0826	-48.7985
ZINC19551581	-41.5469	-48.7955
ZINC19551581	-36.6603	-48.7955
ZINC08440717	-41.9610	-48.7864
ZINC08440717	-40.5493	-48.7864
ZINC04067420	-45.3806	-48.7852
ZINC04067420	-45.1011	-48.7852
ZINC04067420	-43.9484	-48.7852
ZINC08413436	-43.5109	-48.7849
ZINC08413436	-43.2959	-48.7849
ZINC04396004	-40.4007	-48.7849
ZINC06161972	-46.3935	-48.7799
ZINC06161972	-40.7579	-48.7799
ZINC20414408	-41.1522	-48.7785
ZINC08440526	-48.9584	-48.7759
ZINC08440526	-48.0043	-48.7759
ZINC08429944	-44.6531	-48.7748
ZINC08429899	-45.6225	-48.7743
ZINC08429899	-45.4301	-48.7743
ZINC08429899	-43.9829	-48.7743
ZINC04067649	-45.2855	-48.7722
ZINC04067649	-44.8619	-48.7722
ZINC04067649	-44.3762	-48.7722
ZINC06137415	-46.7702	-48.7690
ZINC06137415	-44.5072	-48.7690
ZINC06137415	-40.8030	-48.7690
ZINC08441462	-42.5946	-48.7645
ZINC08441462	-41.3576	-48.7645
ZINC20233049	-46.2613	-48.7560
ZINC20233049	-43.4551	-48.7560
ZINC08695205	-50.1571	-48.7521
ZINC08438751	-44.8199	-48.7448
ZINC19781944	-38.4429	-48.7443
ZINC19781944	-36.7059	-48.7443

ZINC00853757	-43.5286	-48.7421
ZINC00853757	-38.9901	-48.7421
ZINC22790367	-42.4174	-48.7417
ZINC22790367	-40.2137	-48.7417
ZINC04067388	-43.8730	-48.7377
ZINC04067388	-41.9375	-48.7377
ZINC00350499	-38.2743	-48.7340
ZINC05401914	-45.5401	-48.7293
ZINC05401914	-43.2701	-48.7293
ZINC05401914	-42.6786	-48.7293
ZINC13551390	-46.8549	-48.7220
ZINC19552396	-42.6598	-48.7205
ZINC19552396	-40.8880	-48.7205
ZINC08441517	-43.6547	-48.7160
ZINC08441517	-40.7942	-48.7160
ZINC19361480	-39.1727	-48.7124
ZINC19361480	-37.3341	-48.7124
ZINC08695239	-48.2089	-48.7074
ZINC08440566	-50.1282	-48.7000
ZINC08441310	-47.8559	-48.6977
ZINC02183629	-41.9398	-48.6957
ZINC04068299	-49.7977	-48.6860
ZINC13989286	-41.5946	-48.6833
ZINC09242602	-44.8252	-48.6746
ZINC04063844	-42.0172	-48.6738
ZINC08433234	-47.6949	-48.6673
ZINC00633299	-39.2762	-48.6610
ZINC19370782	-42.5792	-48.6581
ZINC19370782	-40.6404	-48.6581
ZINC06195650	-42.5557	-48.6558
ZINC08817981	-43.4609	-48.6519
ZINC08817981	-42.8411	-48.6519
ZINC00702948	-44.3474	-48.6487
ZINC17166755	-43.5021	-48.6438
ZINC17166755	-41.9352	-48.6438
ZINC08441840	-39.9003	-48.6432
ZINC08405929	-43.6271	-48.6419
ZINC04629652	-45.7878	-48.6356
ZINC04629652	-45.1113	-48.6356
ZINC05917235	-44.0826	-48.6351
ZINC00135729	-43.2223	-48.6173
ZINC08441140	-46.2206	-48.6148
ZINC00978743	-49.8695	-48.6138
ZINC08433373	-47.4034	-48.6130
ZINC13563601	-53.0553	-48.6032
ZINC09271081	-44.5981	-48.6002
ZINC06196103	-47.2205	-48.6001
ZINC17112358	-43.3830	-48.5996
ZINC04067408	-46.6748	-48.5960
ZINC04067398	-43.4379	-48.5931
ZINC04067398	-41.8977	-48.5931
ZINC08440936	-43.2071	-48.5875
ZINC08440936	-41.1595	-48.5875
ZINC08742873	-45.8396	-48.5841

ZINC08858106	-46.1736	-48.5793
ZINC06197279	-43.2615	-48.5789
ZINC06162483	-43.1821	-48.5741
ZINC08429890	-45.9260	-48.5739
ZINC08429890	-42.5824	-48.5739
ZINC09088350	-45.9386	-48.5452
ZINC08383636	-47.1930	-48.5419
ZINC19360137	-38.8001	-48.5416
ZINC13770152	-46.6794	-48.5401
ZINC00702998	-45.9381	-48.5378
ZINC08444350	-45.7132	-48.5376
ZINC08444350	-41.5852	-48.5376
ZINC08440715	-42.3963	-48.5361
ZINC08440715	-40.1218	-48.5361
ZINC06137372	-41.2664	-48.5265
ZINC06137372	-38.6263	-48.5265
ZINC08442458	-39.5250	-48.5185
ZINC09361244	-45.9925	-48.5137
ZINC04065858	-43.5492	-48.5123
ZINC08439458	-46.9534	-48.5101
ZINC00848045	-44.6139	-48.5056
ZINC06015503	-42.2703	-48.5038
ZINC08441064	-43.3941	-48.5014
ZINC01413449	-44.3091	-48.4957
ZINC01413449	-43.2520	-48.4957
ZINC17154957	-46.3550	-48.4952
ZINC20233044	-44.9608	-48.4842
ZINC20233044	-44.1695	-48.4842
ZINC04066574	-44.7368	-48.4817
ZINC04066574	-41.8598	-48.4817
ZINC09358782	-42.5626	-48.4794
ZINC08400653	-48.0280	-48.4711
ZINC08400653	-47.2159	-48.4711
ZINC08400653	-45.8327	-48.4711
ZINC06136978	-43.8288	-48.4680
ZINC13576125	-44.5715	-48.4655
ZINC13576125	-43.7756	-48.4655
ZINC04632425	-42.8163	-48.4629
ZINC04067245	-45.5515	-48.4591
ZINC04067245	-45.5141	-48.4591
ZINC04067245	-44.2189	-48.4591
ZINC06144028	-46.0748	-48.4585
ZINC08771328	-44.4819	-48.4548
ZINC08771328	-43.4747	-48.4548
ZINC19872202	-44.9563	-48.4524
ZINC04067707	-45.6740	-48.4516
ZINC04067707	-43.5226	-48.4516
ZINC04067707	-43.2916	-48.4516
ZINC00844260	-45.8303	-48.4492
ZINC00844260	-44.6367	-48.4492
ZINC08441720	-47.9448	-48.4428
ZINC02183631	-40.1843	-48.4267
ZINC19880698	-39.1257	-48.4252
ZINC19880698	-36.9340	-48.4252

ZINC18249663	-44.1229	-48.4241
ZINC08695198	-48.2105	-48.4164
ZINC00702932	-44.6588	-48.4160
ZINC00679175	-43.1067	-48.4073
ZINC00679175	-41.4520	-48.4073
ZINC09071905	-43.9390	-48.4067
ZINC09071905	-42.9629	-48.4067
ZINC08439412	-44.2724	-48.4052
ZINC08396630	-47.7749	-48.4031
ZINC19802462	-45.8351	-48.3999
ZINC19802462	-45.4706	-48.3999
ZINC09414099	-45.7096	-48.3990
ZINC09244237	-42.1938	-48.3948
ZINC08400428	-47.1970	-48.3891
ZINC08400428	-43.9329	-48.3891
ZINC08400428	-41.7311	-48.3891
ZINC19781607	-37.8217	-48.3856
ZINC19781607	-36.5059	-48.3856
ZINC04632292	-44.3666	-48.3851
ZINC04048087	-43.5986	-48.3840
ZINC04048087	-43.1659	-48.3840
ZINC04048087	-40.8280	-48.3840
ZINC00703051	-45.3996	-48.3826
ZINC00703051	-38.2324	-48.3826
ZINC19781580	-37.8368	-48.3816
ZINC06137080	-44.7910	-48.3785
ZINC19366916	-42.7876	-48.3779
ZINC09076145	-47.1424	-48.3728
ZINC09076145	-45.2891	-48.3728
ZINC08441682	-44.3761	-48.3654
ZINC00703127	-44.9012	-48.3649
ZINC00999559	-45.1058	-48.3634
ZINC05894965	-44.3877	-48.3606
ZINC17258392	-39.3729	-48.3599
ZINC08429973	-45.1544	-48.3586
ZINC08429973	-43.9847	-48.3586
ZINC08429973	-41.9010	-48.3586
ZINC08695245	-49.0976	-48.3557
ZINC04065472	-43.8248	-48.3556
ZINC04065472	-42.0018	-48.3556
ZINC04065472	-41.0481	-48.3556
ZINC08441503	-42.9754	-48.3503
ZINC08441503	-41.2932	-48.3503
ZINC06195640	-42.2713	-48.3456
ZINC04905323	-42.1882	-48.3418
ZINC04065990	-42.1992	-48.3377
ZINC04065990	-39.9427	-48.3377
ZINC08416188	-42.7905	-48.3323
ZINC00702928	-44.1416	-48.3262
ZINC08400639	-46.3185	-48.3243
ZINC04067615	-43.2241	-48.3216
ZINC05894971	-45.3908	-48.3075
ZINC09413458	-51.0311	-48.3014
ZINC02753344	-43.5328	-48.3006

ZINC06195410	-42.3805	-48.2999
ZINC85425761	-43.9265	-48.2916
ZINC85425761	-41.2080	-48.2916
ZINC00998842	-44.3139	-48.2811
ZINC08386166	-46.6723	-48.2771
ZINC54178315	-46.6337	-48.2759
ZINC09282460	-47.2013	-48.2749
ZINC09282460	-44.7344	-48.2749
ZINC08439762	-47.2546	-48.2706
ZINC08439762	-46.2728	-48.2706
ZINC04060581	-39.2664	-48.2621
ZINC08445205	-49.9454	-48.2620
ZINC08450289	-44.3652	-48.2559
ZINC08450289	-43.3867	-48.2559
ZINC12523689	-50.6071	-48.2544
ZINC08426159	-42.5091	-48.2403
ZINC08426159	-41.7755	-48.2403
ZINC08426159	-41.3277	-48.2403
ZINC04066347	-44.4060	-48.2390
ZINC00626276	-46.8917	-48.2369
ZINC06196089	-46.5162	-48.2259
ZINC04062496	-42.1188	-48.2256
ZINC04062496	-40.4314	-48.2256
ZINC19797527	-46.4728	-48.2255
ZINC00826267	-43.8019	-48.2251
ZINC00826267	-43.0833	-48.2251
ZINC06162107	-42.6895	-48.2149
ZINC08444363	-40.1331	-48.2107
ZINC04066049	-38.7469	-48.2052
ZINC04066049	-37.7118	-48.2052
ZINC06162489	-42.9514	-48.2043
ZINC00704635	-45.0904	-48.1960
ZINC00704635	-44.9915	-48.1960
ZINC06197294	-44.0546	-48.1949
ZINC08740264	-42.7866	-48.1939
ZINC09379959	-44.7554	-48.1935
ZINC09007706	-44.0897	-48.1916
ZINC04067569	-39.7560	-48.1913
ZINC08453969	-47.7427	-48.1873
ZINC04066276	-44.9812	-48.1866
ZINC04066276	-42.0870	-48.1866
ZINC17154948	-42.7591	-48.1801
ZINC17154948	-39.1285	-48.1801
ZINC00716660	-43.3009	-48.1788
ZINC00716660	-42.4674	-48.1788
ZINC00716660	-42.4285	-48.1788
ZINC08430036	-42.5851	-48.1775
ZINC08430036	-42.5057	-48.1775
ZINC09374115	-42.9703	-48.1759
ZINC08439413	-43.7448	-48.1727
ZINC08439413	-41.2060	-48.1727
ZINC08439413	-41.1960	-48.1727
ZINC08441284	-40.3856	-48.1680
ZINC08441284	-39.6575	-48.1680

ZINC08441284	-39.4182	-48.1680
ZINC06162098	-42.0641	-48.1658
ZINC00844256	-43.8962	-48.1558
ZINC00844256	-41.2652	-48.1558
ZINC04068108	-43.2287	-48.1544
ZINC19456343	-45.8456	-48.1455
ZINC19456343	-41.1099	-48.1455
ZINC04067510	-43.4787	-48.1425
ZINC04067510	-43.1524	-48.1425
ZINC04635181	-44.1302	-48.1389
ZINC06197212	-41.7792	-48.1362
ZINC08383797	-50.6038	-48.1334
ZINC06162091	-43.0251	-48.1331
ZINC00644358	-46.7061	-48.1297
ZINC08433337	-46.9584	-48.1269
ZINC08433337	-46.5906	-48.1269
ZINC19781733	-41.8038	-48.1219
ZINC19781733	-39.4512	-48.1219
ZINC08443079	-42.4434	-48.1209
ZINC06161992	-44.1633	-48.1188
ZINC08996430	-45.6302	-48.1173
ZINC05226008	-42.7091	-48.1143
ZINC04067631	-43.8929	-48.1097
ZINC04067631	-43.4938	-48.1097
ZINC04067631	-42.6698	-48.1097
ZINC08441679	-44.1735	-48.1097
ZINC19555364	-39.6691	-48.1058
ZINC19555364	-36.3880	-48.1058
ZINC05226015	-41.9443	-48.1054
ZINC05226015	-39.4288	-48.1054
ZINC08837798	-45.9898	-48.1031
ZINC00703122	-42.9086	-48.1010
ZINC08399194	-42.8300	-48.0980
ZINC08399194	-40.5915	-48.0980
ZINC00703251	-45.9808	-48.0927
ZINC04391419	-42.0218	-48.0884
ZINC18203891	-47.0524	-48.0872
ZINC09783046	-45.1221	-48.0799
ZINC08414942	-42.3268	-48.0788
ZINC08414942	-41.9766	-48.0788
ZINC08414942	-40.2460	-48.0788
ZINC06162275	-41.4631	-48.0773
ZINC06162275	-40.7517	-48.0773
ZINC08440498	-46.0422	-48.0758
ZINC08440498	-42.5254	-48.0758
ZINC08433386	-46.3763	-48.0754
ZINC08425612	-43.2371	-48.0742
ZINC04066233	-43.0786	-48.0674
ZINC04066233	-42.7696	-48.0674
ZINC09339552	-45.6801	-48.0661
ZINC17166872	-42.1211	-48.0655
ZINC17166872	-40.3874	-48.0655
ZINC17166872	-38.3419	-48.0655
ZINC00678574	-44.3229	-48.0613

ZINC05313299	-48.7437	-48.0599
ZINC08441015	-43.4325	-48.0566
ZINC08383938	-47.2072	-48.0541
ZINC08905392	-42.4924	-48.0489
ZINC08426818	-42.4948	-48.0458
ZINC08426818	-41.6653	-48.0458
ZINC08441559	-41.4326	-48.0451
ZINC04670894	-43.6166	-48.0439
ZINC04067028	-44.2404	-48.0430
ZINC04067028	-40.9408	-48.0430
ZINC04067028	-40.2903	-48.0430
ZINC02183636	-40.7065	-48.0417
ZINC09243862	-51.8388	-48.0362
ZINC09243862	-51.1675	-48.0362
ZINC08440736	-42.0295	-48.0356
ZINC08441299	-47.3984	-48.0353
ZINC08441299	-47.2732	-48.0353
ZINC00703049	-43.6223	-48.0322
ZINC00703049	-40.9513	-48.0322
ZINC08399170	-41.0806	-48.0303
ZINC08399170	-37.9962	-48.0303
ZINC00626290	-45.8667	-48.0291
ZINC08415213	-39.3362	-48.0251
ZINC08415213	-37.7753	-48.0251
ZINC02388691	-39.7302	-48.0231
ZINC08440888	-49.8565	-48.0224
ZINC08441179	-42.3953	-48.0213
ZINC08441179	-41.7804	-48.0213
ZINC00726674	-41.1498	-48.0196
ZINC08439530	-45.3755	-48.0195
ZINC08440759	-43.7080	-48.0154
ZINC08440759	-41.4488	-48.0154
ZINC08440759	-39.5151	-48.0154
ZINC09324555	-45.3816	-48.0139
ZINC09324555	-44.2456	-48.0139
ZINC08396804	-43.7544	-48.0034
ZINC08396804	-42.5002	-48.0034
ZINC08396804	-39.5957	-48.0034
ZINC06148585	-43.6750	-48.0010
ZINC19802136	-38.6818	-47.9985
ZINC22076681	-41.9674	-47.9958
ZINC08383560	-43.4753	-47.9939
ZINC04066242	-43.5495	-47.9898
ZINC04066242	-40.1211	-47.9898
ZINC08441231	-42.9001	-47.9897
ZINC08439615	-44.2243	-47.9890
ZINC08439615	-43.4491	-47.9890
ZINC00631530	-46.5182	-47.9875
ZINC19360167	-36.7995	-47.9828
ZINC08383633	-47.3221	-47.9785
ZINC08440758	-43.2367	-47.9760
ZINC08440758	-38.3878	-47.9760
ZINC08387280	-49.3225	-47.9739
ZINC09471295	-44.6499	-47.9726

ZINC09471295	-42.6753	-47.9726
ZINC08396652	-47.9207	-47.9571
ZINC08444354	-46.3368	-47.9564
ZINC08444354	-44.6736	-47.9564
ZINC08437425	-43.8756	-47.9563
ZINC08441857	-39.5911	-47.9546
ZINC06137375	-41.6039	-47.9544
ZINC06137375	-40.4462	-47.9544
ZINC08441511	-42.9123	-47.9479
ZINC09329127	-43.4098	-47.9420
ZINC09329127	-41.9660	-47.9420
ZINC09446494	-44.7333	-47.9419
ZINC08915008	-45.0063	-47.9325
ZINC08915008	-43.1942	-47.9325
ZINC04629627	-42.6907	-47.9312
ZINC04629627	-41.8935	-47.9312
ZINC00703180	-45.4285	-47.9293
ZINC06196115	-46.0896	-47.9292
ZINC19781908	-40.4031	-47.9270
ZINC19781908	-39.7144	-47.9270
ZINC08996446	-48.1512	-47.9254
ZINC04068107	-46.1934	-47.9242
ZINC06195573	-42.0497	-47.9204
ZINC08429938	-43.2144	-47.9184
ZINC08441605	-42.9094	-47.9154
ZINC08439482	-47.6624	-47.9135
ZINC08439482	-45.9469	-47.9135
ZINC08439482	-43.2126	-47.9135
ZINC08397031	-41.0028	-47.9061
ZINC09507451	-46.5820	-47.9050
ZINC06137376	-44.3082	-47.8973
ZINC08415912	-44.4610	-47.8819
ZINC08415912	-38.7885	-47.8819
ZINC00850070	-47.8783	-47.8802
ZINC19370749	-41.8220	-47.8755
ZINC19370749	-38.1784	-47.8755
ZINC09243142	-45.0864	-47.8749
ZINC09243142	-41.8301	-47.8749
ZINC17207069	-40.9973	-47.8732
ZINC04018574	-45.5219	-47.8711
ZINC08383648	-46.2418	-47.8668
ZINC17532934	-45.9441	-47.8627
ZINC19904606	-43.9790	-47.8619
ZINC18203417	-48.4859	-47.8575
ZINC08383638	-43.2811	-47.8569
ZINC08433428	-46.1838	-47.8514
ZINC08433433	-42.5121	-47.8495
ZINC04114845	-42.8640	-47.8482
ZINC06149699	-46.8079	-47.8456
ZINC08439858	-44.4200	-47.8449
ZINC08439858	-43.8456	-47.8449
ZINC08988144	-45.0749	-47.8436
ZINC09301885	-41.8732	-47.8435
ZINC04067753	-43.4077	-47.8429

ZINC08415533	-45.1983	-47.8355
ZINC08387669	-40.7120	-47.8340
ZINC08387669	-39.9669	-47.8340
ZINC08439604	-43.3100	-47.8308
ZINC08439604	-43.1806	-47.8308
ZINC09009213	-49.5938	-47.8299
ZINC09009213	-49.2333	-47.8299
ZINC09009213	-48.3969	-47.8299
ZINC09009213	-44.1303	-47.8299
ZINC09009213	-43.9385	-47.8299
ZINC00702744	-43.7182	-47.8288
ZINC00702744	-43.4329	-47.8288
ZINC08386781	-45.7652	-47.8268
ZINC08386781	-44.0299	-47.8268
ZINC08439840	-45.7236	-47.8205
ZINC08439840	-41.7105	-47.8205
ZINC06149831	-42.4079	-47.8203
ZINC19943530	-47.8031	-47.8200
ZINC04067709	-45.9234	-47.8153
ZINC04067709	-45.6621	-47.8153
ZINC08400633	-43.8892	-47.8127
ZINC08400633	-43.3967	-47.8127
ZINC08441664	-44.9993	-47.8083
ZINC09462477	-45.3093	-47.8075
ZINC19794745	-44.0611	-47.8070
ZINC00702939	-48.7254	-47.8062
ZINC08413307	-43.3334	-47.7917
ZINC08413307	-40.2107	-47.7917
ZINC06015284	-44.2157	-47.7897
ZINC06015284	-44.0670	-47.7897
ZINC06194437	-43.0358	-47.7894
ZINC06194437	-42.5485	-47.7894
ZINC04284869	-44.0863	-47.7886
ZINC04284869	-41.9564	-47.7886
ZINC00729420	-44.2685	-47.7881
ZINC00729420	-44.1974	-47.7881
ZINC00729420	-40.9345	-47.7881
ZINC06442970	-42.0599	-47.7872
ZINC08439578	-42.3866	-47.7826
ZINC08441820	-41.6331	-47.7814
ZINC18168438	-44.9500	-47.7813
ZINC04065855	-43.5818	-47.7797
ZINC04065855	-38.7847	-47.7797
ZINC09301925	-44.0162	-47.7786
ZINC09301925	-41.1660	-47.7786
ZINC04068138	-45.0092	-47.7780
ZINC09380390	-44.9734	-47.7779
ZINC08996198	-43.2198	-47.7749
ZINC08996198	-42.9635	-47.7749
ZINC08450305	-45.1290	-47.7736
ZINC08988147	-45.6404	-47.7699
ZINC08988147	-44.9302	-47.7699
ZINC04661420	-39.0182	-47.7666
ZINC20219827	-49.6203	-47.7649

ZINC06194156	-41.2225	-47.7587
ZINC06194156	-41.1014	-47.7587
ZINC13551393	-46.5890	-47.7555
ZINC13685270	-45.2077	-47.7522
ZINC19872213	-40.7309	-47.7497
ZINC00968742	-41.9601	-47.7484
ZINC00968742	-39.7999	-47.7484
ZINC00968742	-38.2046	-47.7484
ZINC08433426	-45.8641	-47.7479
ZINC05296301	-43.7927	-47.7430
ZINC05296301	-42.3294	-47.7430
ZINC05296301	-40.8821	-47.7430
ZINC08415263	-44.6389	-47.7423
ZINC08415263	-43.8022	-47.7423
ZINC00691627	-42.5877	-47.7409
ZINC06162103	-43.3423	-47.7408
ZINC09135492	-42.7320	-47.7377
ZINC08441685	-46.0487	-47.7324
ZINC20111195	-38.7118	-47.7324
ZINC08440101	-44.7258	-47.7323
ZINC09068368	-45.5215	-47.7310
ZINC00702952	-44.6183	-47.7297
ZINC00702952	-42.8775	-47.7297
ZINC00702952	-41.7948	-47.7297
ZINC06196036	-42.9140	-47.7271
ZINC08400251	-42.7568	-47.7241
ZINC08400251	-42.2560	-47.7241
ZINC16115872	-43.5498	-47.7211
ZINC16115872	-40.1540	-47.7211
ZINC19872226	-39.6498	-47.7127
ZINC08437249	-45.0370	-47.7122
ZINC19832137	-37.5977	-47.7112
ZINC19832137	-36.8156	-47.7112
ZINC08445173	-43.8673	-47.7043
ZINC08441217	-42.8072	-47.7011
ZINC08441217	-40.8608	-47.7011
ZINC00727072	-46.5978	-47.7002
ZINC08414948	-42.0118	-47.6983
ZINC08414948	-37.3912	-47.6983
ZINC08414948	-37.3706	-47.6983
ZINC09357466	-45.7369	-47.6936
ZINC09357466	-44.4370	-47.6936
ZINC09357466	-43.4590	-47.6936
ZINC08441702	-43.6603	-47.6892
ZINC04417692	-42.0557	-47.6855
ZINC04417692	-41.7684	-47.6855
ZINC00847629	-44.3465	-47.6784
ZINC08439505	-44.3335	-47.6782
ZINC08441629	-43.1861	-47.6775
ZINC08441629	-41.4504	-47.6775
ZINC08385298	-48.7150	-47.6761
ZINC08985983	-43.4959	-47.6756
ZINC08985983	-41.8695	-47.6756
ZINC19771981	-43.5541	-47.6721

ZINC01019496	-45.1366	-47.6673
ZINC01019496	-45.0358	-47.6673
ZINC13042975	-44.3293	-47.6667
ZINC19880649	-44.6134	-47.6661
ZINC19880649	-43.7470	-47.6661
ZINC08444667	-42.3223	-47.6649
ZINC08444667	-39.6021	-47.6649
ZINC02093283	-41.2568	-47.6636
ZINC04068157	-43.4460	-47.6620
ZINC04068157	-42.6296	-47.6620
ZINC04068157	-39.5087	-47.6620
ZINC08440727	-42.7094	-47.6602
ZINC08440727	-39.6185	-47.6602
ZINC06137511	-40.0277	-47.6572
ZINC08441327	-39.4868	-47.6469
ZINC08384228	-46.7583	-47.6441
ZINC08384228	-44.2069	-47.6441
ZINC08384228	-43.4565	-47.6441
ZINC09359780	-47.2098	-47.6422
ZINC09359780	-45.0969	-47.6422
ZINC04067804	-43.0946	-47.6380
ZINC08397390	-43.9745	-47.6339
ZINC08397390	-42.1922	-47.6339
ZINC08383507	-45.2398	-47.6292
ZINC08383507	-44.2285	-47.6292
ZINC00658279	-43.5477	-47.6268
ZINC00703183	-48.3426	-47.6254
ZINC00703183	-45.3078	-47.6254
ZINC08439426	-44.1260	-47.6250
ZINC08439426	-43.2601	-47.6250
ZINC08439426	-40.8214	-47.6250
ZINC09324695	-44.2297	-47.6241
ZINC08439322	-41.6479	-47.6237
ZINC08439322	-38.5871	-47.6237
ZINC06197640	-44.7134	-47.6151
ZINC08400654	-46.8175	-47.6148
ZINC08400654	-45.0015	-47.6148
ZINC08858184	-46.5098	-47.6131
ZINC08858184	-43.6684	-47.6131
ZINC08430092	-43.4623	-47.6124
ZINC08430092	-43.3666	-47.6124
ZINC08430092	-42.6850	-47.6124
ZINC06162293	-44.5949	-47.6109
ZINC08444352	-40.1186	-47.6086
ZINC08441856	-44.5237	-47.6079
ZINC08440595	-48.4535	-47.6047
ZINC00722087	-40.9096	-47.6019
ZINC06149696	-47.8180	-47.5982
ZINC08715654	-45.0390	-47.5971
ZINC18181228	-47.6955	-47.5954
ZINC18181228	-44.9027	-47.5954
ZINC08441320	-41.0988	-47.5882
ZINC13896346	-47.9773	-47.5865
ZINC13896346	-47.7378	-47.5865

ZINC19909088	-42.5325	-47.5768
ZINC19909088	-39.9537	-47.5768
ZINC06137340	-44.1688	-47.5750
ZINC06162080	-40.0934	-47.5745
ZINC18141313	-44.0735	-47.5735
ZINC18141313	-40.9992	-47.5735
ZINC18141313	-39.2632	-47.5735
ZINC00727073	-47.1771	-47.5721
ZINC06197444	-42.9484	-47.5713
ZINC08837889	-42.5378	-47.5664
ZINC19781798	-38.0878	-47.5643
ZINC08398852	-45.0640	-47.5639
ZINC08444336	-43.8793	-47.5584
ZINC08439537	-47.6875	-47.5580
ZINC08439537	-45.6831	-47.5580
ZINC19852486	-49.7304	-47.5540
ZINC08441639	-42.6183	-47.5487
ZINC08441639	-37.1457	-47.5487
ZINC08383892	-42.8818	-47.5453
ZINC08383892	-41.9518	-47.5453
ZINC02086040	-40.5758	-47.5424
ZINC08399650	-44.3358	-47.5420
ZINC08399650	-43.8035	-47.5420
ZINC04905441	-41.7664	-47.5363
ZINC08440714	-44.0127	-47.5322
ZINC08440714	-42.2843	-47.5322
ZINC08450403	-45.6742	-47.5281
ZINC08399484	-42.3504	-47.5258
ZINC08399484	-41.9233	-47.5258
ZINC04067992	-40.3447	-47.5223
ZINC04067992	-39.7419	-47.5223
ZINC04067992	-36.7890	-47.5223
ZINC08440486	-45.3906	-47.5187
ZINC00719485	-45.9098	-47.5135
ZINC08442104	-44.9471	-47.5085
ZINC08442104	-42.7835	-47.5085
ZINC08396759	-46.1055	-47.5076
ZINC09046597	-46.6032	-47.5037
ZINC09046597	-41.4394	-47.5037
ZINC09046597	-40.7914	-47.5037
ZINC04061341	-38.8375	-47.5027
ZINC19781969	-36.4809	-47.5011
ZINC09360907	-42.7641	-47.5003
ZINC09360907	-40.4533	-47.5003
ZINC19369747	-38.9456	-47.4981
ZINC19369747	-37.1931	-47.4981
ZINC04062033	-41.5867	-47.4959
ZINC08435163	-43.0341	-47.4929
ZINC19360161	-39.2516	-47.4921
ZINC09411248	-46.0208	-47.4900
ZINC08440732	-41.0562	-47.4900
ZINC08441100	-36.7481	-47.4884
ZINC04065844	-42.2996	-47.4866
ZINC04065844	-38.7231	-47.4866

ZINC20028398	-41.6626	-47.4865
ZINC08430357	-44.7246	-47.4855
ZINC08430357	-44.2878	-47.4855
ZINC08430357	-42.6406	-47.4855
ZINC06197200	-42.4128	-47.4854
ZINC04100182	-42.4012	-47.4851
ZINC04100182	-37.7893	-47.4851
ZINC08996429	-47.1514	-47.4834
ZINC17166826	-42.9160	-47.4828
ZINC17166826	-42.6489	-47.4828
ZINC17166826	-37.1510	-47.4828
ZINC08695186	-50.0558	-47.4758
ZINC06442977	-44.5535	-47.4707
ZINC08427628	-46.9654	-47.4702
ZINC08430203	-44.9076	-47.4672
ZINC08430203	-42.9789	-47.4672
ZINC08430203	-40.4328	-47.4672
ZINC04579494	-45.4498	-47.4666
ZINC04066208	-44.0313	-47.4651
ZINC04066208	-40.1064	-47.4651
ZINC04067683	-44.5588	-47.4583
ZINC04067683	-44.1129	-47.4583
ZINC04067683	-44.0143	-47.4583
ZINC08416231	-43.4147	-47.4547
ZINC19802108	-37.0906	-47.4466
ZINC19924475	-45.2881	-47.4403
ZINC19924475	-42.8519	-47.4403
ZINC06162283	-41.3338	-47.4366
ZINC06162283	-40.0322	-47.4366
ZINC04059940	-41.5482	-47.4364
ZINC03663592	-53.1125	-47.4352
ZINC02054350	-43.4332	-47.4352
ZINC08441719	-42.5068	-47.4314
ZINC08438668	-45.9352	-47.4297
ZINC08438668	-45.2145	-47.4297
ZINC08429918	-44.2037	-47.4204
ZINC08429918	-39.9215	-47.4204
ZINC08383771	-52.2674	-47.4174
ZINC08921198	-45.1629	-47.4158
ZINC08921198	-41.7079	-47.4158
ZINC19802432	-40.5422	-47.4147
ZINC19802432	-37.3296	-47.4147
ZINC08383881	-46.0448	-47.4082
ZINC08383881	-45.5467	-47.4082
ZINC08396628	-46.6461	-47.4075
ZINC00984800	-48.5881	-47.3987
ZINC06149679	-43.2207	-47.3961
ZINC17118078	-39.9846	-47.3895
ZINC00847633	-41.2885	-47.3849
ZINC08437306	-44.5855	-47.3845
ZINC08383706	-46.4756	-47.3805
ZINC04067754	-41.2687	-47.3778
ZINC04065692	-44.9568	-47.3742
ZINC04065692	-43.8853	-47.3742

ZINC08448904	-44.2845	-47.3709
ZINC08400426	-48.9614	-47.3681
ZINC08400426	-41.7408	-47.3681
ZINC08400426	-41.4566	-47.3681
ZINC09425015	-45.7439	-47.3673
ZINC08413316	-43.0137	-47.3673
ZINC09275704	-45.9228	-47.3646
ZINC09275704	-45.3391	-47.3646
ZINC09275704	-45.2502	-47.3646
ZINC00702936	-44.5276	-47.3617
ZINC00702999	-47.0376	-47.3590
ZINC05918614	-42.9027	-47.3547
ZINC05918614	-42.8308	-47.3547
ZINC02088441	-45.0519	-47.3508
ZINC19360089	-43.7747	-47.3458
ZINC06197206	-43.2296	-47.3448
ZINC06197206	-43.0386	-47.3448
ZINC08441776	-46.1801	-47.3396
ZINC85378490	-45.8328	-47.3358
ZINC06015533	-42.0135	-47.3350
ZINC06015533	-37.7134	-47.3350
ZINC06015533	-37.3121	-47.3350
ZINC06197185	-43.6750	-47.3339
ZINC06197185	-40.4177	-47.3339
ZINC06195599	-43.5649	-47.3338
ZINC06195599	-38.3782	-47.3338
ZINC08437368	-43.0543	-47.3300
ZINC05450566	-44.4305	-47.3283
ZINC19781992	-37.2647	-47.3264
ZINC04301722	-44.6385	-47.3249
ZINC08441845	-46.3615	-47.3210
ZINC18113452	-40.8784	-47.3176
ZINC08383664	-47.6804	-47.3170
ZINC09379960	-44.6104	-47.3165
ZINC09379960	-43.9085	-47.3165
ZINC08416196	-44.0572	-47.3150
ZINC08416196	-43.6971	-47.3150
ZINC08416196	-42.7530	-47.3150
ZINC00679171	-43.0596	-47.3143
ZINC00679171	-41.6398	-47.3143
ZINC06781925	-43.7720	-47.3135
ZINC06781925	-38.5353	-47.3135
ZINC06197161	-43.0154	-47.3124
ZINC02058056	-45.3205	-47.3092
ZINC08425429	-43.5740	-47.3083
ZINC08741004	-41.0520	-47.3053
ZINC06197293	-43.6817	-47.3030
ZINC08441227	-44.5171	-47.3025
ZINC08441227	-40.7920	-47.3025
ZINC02071244	-46.8873	-47.3007
ZINC06196075	-44.0038	-47.2995
ZINC08397159	-41.7146	-47.2988
ZINC04066404	-40.0905	-47.2979
ZINC08440852	-43.6681	-47.2974

ZINC09008310	-43.1074	-47.2893
ZINC09008310	-42.4843	-47.2893
ZINC19938391	-45.2671	-47.2878
ZINC13942802	-43.0409	-47.2779
ZINC13942802	-41.2169	-47.2779
ZINC13942802	-39.8489	-47.2779
ZINC13942802	-39.2405	-47.2779
ZINC06162261	-45.7591	-47.2738
ZINC16672037	-45.7701	-47.2737
ZINC16672037	-45.5927	-47.2737
ZINC06195434	-42.9350	-47.2623
ZINC05225806	-43.3528	-47.2615
ZINC08416034	-43.1574	-47.2586
ZINC08416034	-42.6151	-47.2586
ZINC19871855	-39.0997	-47.2581
ZINC08022162	-41.5588	-47.2569
ZINC08440113	-43.3073	-47.2497
ZINC08440113	-42.7229	-47.2497
ZINC04066346	-41.7086	-47.2496
ZINC06195635	-43.4842	-47.2490
ZINC08441392	-44.4874	-47.2474
ZINC08441392	-42.6634	-47.2474
ZINC09349788	-45.3385	-47.2473
ZINC09349788	-41.7821	-47.2473
ZINC08450402	-45.5374	-47.2347
ZINC08441690	-42.8910	-47.2327
ZINC08415539	-45.0628	-47.2311
ZINC08415539	-44.2655	-47.2311
ZINC08415539	-37.4351	-47.2311
ZINC20031582	-42.9362	-47.2300
ZINC20031582	-41.9580	-47.2300
ZINC19926546	-42.3310	-47.2213
ZINC09046787	-46.6890	-47.2195
ZINC09046787	-46.3664	-47.2195
ZINC09046787	-45.7973	-47.2195
ZINC08695207	-47.0899	-47.2184
ZINC00702759	-41.1669	-47.2070
ZINC08383827	-45.9292	-47.2039
ZINC08383827	-44.2354	-47.2039
ZINC08386161	-47.8284	-47.2033
ZINC04780897	-39.6558	-47.2023
ZINC19832284	-44.6279	-47.2018
ZINC19832284	-43.1465	-47.2018
ZINC08441283	-49.1925	-47.1993
ZINC08441283	-48.0486	-47.1993
ZINC08441283	-46.1938	-47.1993
ZINC22130537	-39.5012	-47.1973
ZINC22130537	-37.6104	-47.1973
ZINC08440507	-45.4019	-47.1971
ZINC08440507	-44.5309	-47.1971
ZINC68734845	-41.2048	-47.1962
ZINC00629958	-44.4442	-47.1960
ZINC08441839	-43.6558	-47.1956
ZINC04065690	-45.1519	-47.1943

ZINC04065690	-43.6607	-47.1943
ZINC04065690	-43.2153	-47.1943
ZINC08440401	-43.4190	-47.1936
ZINC08440401	-41.0270	-47.1936
ZINC08415997	-43.6559	-47.1907
ZINC08415997	-39.1785	-47.1907
ZINC08415997	-37.5187	-47.1907
ZINC00726689	-43.8675	-47.1893
ZINC00726689	-42.6294	-47.1893
ZINC00726689	-42.2705	-47.1893
ZINC08996588	-44.8643	-47.1849
ZINC06195580	-43.8817	-47.1825
ZINC05396702	-41.9119	-47.1818
ZINC05396702	-40.3874	-47.1818
ZINC06137177	-43.3239	-47.1770
ZINC06137177	-42.2334	-47.1770
ZINC06137177	-39.3866	-47.1770
ZINC04066223	-42.6360	-47.1736
ZINC04066223	-42.2518	-47.1736
ZINC19908770	-43.6200	-47.1722
ZINC08742475	-42.4274	-47.1712
ZINC00709905	-44.7627	-47.1699
ZINC00709905	-44.2090	-47.1699
ZINC00709905	-42.3334	-47.1699
ZINC08721233	-43.1803	-47.1688
ZINC08441220	-38.3398	-47.1651
ZINC02066986	-44.0042	-47.1631
ZINC08860016	-45.4827	-47.1629
ZINC08860016	-42.8702	-47.1629
ZINC00823541	-42.4583	-47.1609
ZINC06444803	-47.5903	-47.1603
ZINC04066448	-43.3991	-47.1600
ZINC04066448	-42.6701	-47.1600
ZINC08384170	-47.3392	-47.1574
ZINC08384170	-45.1970	-47.1574
ZINC19366853	-45.3493	-47.1530
ZINC08896625	-44.9050	-47.1516
ZINC09359468	-43.8740	-47.1511
ZINC09425160	-44.0899	-47.1510
ZINC09425160	-43.7828	-47.1510
ZINC06195556	-46.9835	-47.1509
ZINC09015106	-45.2844	-47.1460
ZINC09425131	-44.7106	-47.1446
ZINC09425131	-44.0897	-47.1446
ZINC13045551	-47.5329	-47.1433
ZINC13045551	-46.5677	-47.1433
ZINC04067246	-42.6804	-47.1425
ZINC04067246	-41.5900	-47.1425
ZINC04067246	-41.4184	-47.1425
ZINC04065136	-43.7645	-47.1413
ZINC04065136	-43.7142	-47.1413
ZINC02103515	-39.7242	-47.1408
ZINC00703105	-42.6041	-47.1392
ZINC00703105	-39.8153	-47.1392

ZINC04067854	-45.5617	-47.1376
ZINC15658715	-47.9970	-47.1352
ZINC06137405	-43.9117	-47.1351
ZINC06137405	-36.7477	-47.1351
ZINC08450341	-43.4009	-47.1332
ZINC08450341	-39.7726	-47.1332
ZINC08695242	-46.3683	-47.1294
ZINC08416045	-44.0627	-47.1272
ZINC08416045	-43.8213	-47.1272
ZINC08416045	-39.7855	-47.1272
ZINC08951383	-49.0854	-47.1234
ZINC09455307	-44.3491	-47.1176
ZINC09455307	-43.9456	-47.1176
ZINC06197104	-44.8758	-47.1157
ZINC00703126	-44.5708	-47.1107
ZINC08441246	-42.3941	-47.1094
ZINC08441246	-39.9017	-47.1094
ZINC06195471	-43.2172	-47.1079
ZINC06195471	-42.4591	-47.1079
ZINC04067846	-44.2812	-47.1071
ZINC13045547	-43.9505	-47.1005
ZINC19880646	-40.5438	-47.0995
ZINC06195500	-46.3533	-47.0977
ZINC09272353	-44.5888	-47.0973
ZINC08441960	-46.6956	-47.0967
ZINC08441960	-44.2746	-47.0967
ZINC08441960	-42.2953	-47.0967
ZINC04417960	-42.6050	-47.0961
ZINC04417960	-41.3671	-47.0961
ZINC08384220	-41.6083	-47.0959
ZINC08442202	-43.9310	-47.0909
ZINC06136856	-42.5676	-47.0882
ZINC00678546	-46.8693	-47.0879
ZINC19872535	-41.1218	-47.0858
ZINC06144032	-43.7533	-47.0855
ZINC04905512	-43.1346	-47.0835
ZINC09358641	-45.8375	-47.0804
ZINC09358641	-45.7668	-47.0804
ZINC06162114	-45.6089	-47.0801
ZINC37866590	-47.3531	-47.0772
ZINC18087471	-43.1838	-47.0747
ZINC18087471	-43.0324	-47.0747
ZINC08440933	-47.0037	-47.0716
ZINC08440901	-46.7754	-47.0713
ZINC04391376	-45.1993	-47.0708
ZINC19922790	-46.6516	-47.0702
ZINC08416154	-42.4247	-47.0700
ZINC08416154	-41.6386	-47.0700
ZINC04867797	-43.2199	-47.0677
ZINC13548583	-41.9964	-47.0655
ZINC13548583	-40.7015	-47.0655
ZINC04019579	-42.1068	-47.0654
ZINC04019579	-42.0738	-47.0654
ZINC08441861	-45.6751	-47.0641

ZINC04066482	-41.4676	-47.0617
ZINC04066482	-38.6243	-47.0617
ZINC08416235	-42.0845	-47.0613
ZINC04629639	-41.1618	-47.0598
ZINC04629639	-40.9657	-47.0598
ZINC13161534	-47.3944	-47.0572
ZINC13161534	-46.4623	-47.0572
ZINC00702446	-44.6987	-47.0546
ZINC00702446	-44.4853	-47.0546
ZINC00702446	-43.2524	-47.0546
ZINC00702446	-42.7152	-47.0546
ZINC00702446	-41.7482	-47.0546
ZINC19831785	-37.9129	-47.0542
ZINC19831785	-37.1788	-47.0542
ZINC04067819	-40.7560	-47.0510
ZINC04067819	-39.0522	-47.0510
ZINC08439603	-42.8293	-47.0494
ZINC04067341	-43.2039	-47.0487
ZINC06195678	-42.5754	-47.0484
ZINC09324554	-42.2138	-47.0480
ZINC09324554	-42.1663	-47.0480
ZINC08439461	-46.2275	-47.0398
ZINC08439461	-43.4526	-47.0398
ZINC08439461	-43.2494	-47.0398
ZINC08997042	-43.5677	-47.0369
ZINC08997042	-43.1513	-47.0369
ZINC08396808	-41.8279	-47.0366
ZINC08441610	-38.8155	-47.0359
ZINC08441610	-37.4436	-47.0359
ZINC08441610	-36.9191	-47.0359
ZINC15952853	-46.4923	-47.0353
ZINC36026114	-44.6250	-47.0331
ZINC06162111	-43.3468	-47.0329
ZINC08439457	-44.8765	-47.0291
ZINC08413347	-45.6630	-47.0255
ZINC08413347	-42.5148	-47.0255
ZINC08440749	-38.7539	-47.0238
ZINC08440749	-37.4602	-47.0238
ZINC06197091	-44.8962	-47.0228
ZINC08433403	-48.0572	-47.0222
ZINC08439971	-45.3573	-47.0198
ZINC02088186	-42.2860	-47.0160
ZINC02088186	-38.4182	-47.0160
ZINC08715720	-43.2276	-47.0099
ZINC08715720	-40.5193	-47.0099
ZINC08441367	-49.2707	-47.0057
ZINC06149694	-47.7988	-47.0035
ZINC04064264	-42.8007	-47.0034
ZINC04064264	-42.7144	-47.0034
ZINC04064264	-42.0508	-47.0034
ZINC06137123	-44.5413	-46.9997
ZINC02055395	-42.9442	-46.9989
ZINC08383945	-47.0701	-46.9957
ZINC04632424	-43.2449	-46.9913

ZINC04632424	-42.4350	-46.9913
ZINC04068140	-44.2000	-46.9907
ZINC08440739	-38.6775	-46.9887
ZINC08440739	-37.3491	-46.9887
ZINC08439862	-44.9041	-46.9838
ZINC04061267	-42.2322	-46.9827
ZINC04061267	-40.6682	-46.9827
ZINC00678549	-46.0984	-46.9800
ZINC08741466	-47.9366	-46.9787
ZINC08741466	-45.3518	-46.9787
ZINC19901678	-38.5552	-46.9775
ZINC05130508	-46.4481	-46.9774
ZINC00714611	-41.5636	-46.9757
ZINC00714611	-39.8446	-46.9757
ZINC00714611	-38.8634	-46.9757
ZINC06659822	-47.3079	-46.9755
ZINC19366920	-41.1290	-46.9752
ZINC08439388	-40.4942	-46.9731
ZINC15162745	-46.1553	-46.9710
ZINC15879999	-43.3414	-46.9698
ZINC02841631	-40.0483	-46.9678
ZINC08440687	-45.9583	-46.9661
ZINC17136176	-41.0754	-46.9626
ZINC08396861	-45.0870	-46.9559
ZINC04063823	-38.4697	-46.9553
ZINC00844272	-41.9226	-46.9551
ZINC00844272	-38.1836	-46.9551
ZINC06015346	-42.8683	-46.9521
ZINC19790752	-44.8741	-46.9468
ZINC08415528	-44.0028	-46.9461
ZINC08415528	-43.0581	-46.9461
ZINC08415528	-37.9773	-46.9461
ZINC04170770	-41.5977	-46.9425
ZINC04170770	-41.4927	-46.9425
ZINC04170770	-41.0691	-46.9425
ZINC09241169	-43.1165	-46.9419
ZINC09241169	-42.7461	-46.9419
ZINC00853739	-43.4363	-46.9401
ZINC08385576	-48.8228	-46.9382
ZINC12447502	-42.4844	-46.9362
ZINC39180816	-40.6788	-46.9346
ZINC08442187	-38.5618	-46.9333
ZINC09358780	-40.9227	-46.9239
ZINC04417649	-41.3236	-46.9228
ZINC04417649	-37.7173	-46.9228
ZINC19832123	-39.2258	-46.9222
ZINC19832123	-38.7984	-46.9222
ZINC08441513	-40.5474	-46.9221
ZINC08441513	-40.2944	-46.9221
ZINC08442216	-39.9143	-46.9217
ZINC19908384	-44.0864	-46.9214
ZINC17166817	-42.8803	-46.9207
ZINC17166817	-41.7947	-46.9207
ZINC17166817	-38.4764	-46.9207

ZINC04019576	-42.5707	-46.9194
ZINC04019576	-42.0445	-46.9194
ZINC06162104	-40.6574	-46.9153
ZINC15134452	-40.7483	-46.9150
ZINC08441801	-46.5986	-46.9147
ZINC08438677	-44.3381	-46.9132
ZINC08438677	-43.8394	-46.9132
ZINC04066239	-42.6277	-46.9091
ZINC08441207	-42.0748	-46.9075
ZINC08441207	-40.2896	-46.9075
ZINC04114964	-40.1649	-46.9059
ZINC06997165	-44.9117	-46.9041
ZINC06162358	-43.2489	-46.9025
ZINC06995622	-47.1110	-46.9022
ZINC06995622	-41.8552	-46.9022
ZINC06149693	-49.6339	-46.8929
ZINC00702742	-45.9025	-46.8913
ZINC00702742	-43.2450	-46.8913
ZINC08416407	-42.2721	-46.8901
ZINC19938430	-48.4240	-46.8886
ZINC13551445	-49.7580	-46.8819
ZINC13551445	-45.8943	-46.8819
ZINC13551445	-42.2727	-46.8819
ZINC06137220	-44.4990	-46.8798
ZINC08440443	-43.7689	-46.8796
ZINC04067419	-42.4624	-46.8777
ZINC04067419	-41.5639	-46.8777
ZINC04067419	-39.2850	-46.8777
ZINC13475852	-43.7823	-46.8762
ZINC13475852	-41.9856	-46.8762
ZINC13475852	-39.1465	-46.8762
ZINC05920825	-44.6568	-46.8755
ZINC05920825	-43.1666	-46.8755
ZINC08440744	-42.4694	-46.8729
ZINC08440744	-40.8239	-46.8729
ZINC04067544	-44.2586	-46.8675
ZINC19361219	-38.1072	-46.8651
ZINC19361219	-36.5545	-46.8651
ZINC04068013	-42.9340	-46.8648
ZINC04068013	-39.0662	-46.8648
ZINC08440721	-43.0536	-46.8600
ZINC06197205	-43.1041	-46.8569
ZINC06197205	-39.9417	-46.8569
ZINC08440792	-43.3352	-46.8560
ZINC06137422	-42.0814	-46.8558
ZINC19790730	-41.5962	-46.8514
ZINC19781716	-39.2915	-46.8448
ZINC19781716	-38.7038	-46.8448
ZINC00628984	-42.1644	-46.8438
ZINC00628984	-39.5764	-46.8438
ZINC08791939	-48.8938	-46.8435
ZINC19360128	-39.9008	-46.8431
ZINC19360128	-39.7115	-46.8431
ZINC00998841	-43.9294	-46.8424

ZINC04112073	-40.5682	-46.8416
ZINC08383521	-45.3829	-46.8391
ZINC08439831	-41.1048	-46.8382
ZINC04067404	-41.5643	-46.8284
ZINC04067404	-39.6806	-46.8284
ZINC04067404	-38.9254	-46.8284
ZINC18191134	-49.6727	-46.8283
ZINC18191134	-47.8840	-46.8283
ZINC18191134	-44.5749	-46.8283
ZINC18191134	-43.8615	-46.8283
ZINC09312388	-44.1013	-46.8188
ZINC09462224	-48.8955	-46.8182
ZINC09462224	-48.2936	-46.8182
ZINC06196398	-40.2582	-46.8180
ZINC08411678	-42.7970	-46.8168
ZINC08411678	-41.5870	-46.8168
ZINC08439842	-41.5903	-46.8158
ZINC09272834	-43.3907	-46.8158
ZINC06136984	-42.5590	-46.8156
ZINC08383883	-44.8199	-46.8132
ZINC08383883	-42.4522	-46.8132
ZINC08444359	-43.1017	-46.8124
ZINC08444359	-40.5762	-46.8124
ZINC06161997	-45.8902	-46.8107
ZINC06161997	-39.5150	-46.8107
ZINC08435313	-46.1807	-46.8098
ZINC00703184	-48.3635	-46.8077
ZINC00703184	-46.0317	-46.8077
ZINC08441614	-43.9149	-46.8064
ZINC08441614	-38.2957	-46.8064
ZINC06015249	-46.8507	-46.8061
ZINC06015249	-40.1535	-46.8061
ZINC06015249	-38.0707	-46.8061
ZINC06196545	-41.4379	-46.8042
ZINC08411093	-43.4559	-46.8017
ZINC08411093	-42.2335	-46.8017
ZINC19944959	-38.8759	-46.8007
ZINC19944959	-37.7542	-46.8007
ZINC08430271	-44.9977	-46.7990
ZINC08430271	-44.1288	-46.7990
ZINC08430271	-41.8888	-46.7990
ZINC08996757	-41.1954	-46.7968
ZINC08996757	-40.4996	-46.7968
ZINC19720188	-46.1578	-46.7954
ZINC19720188	-39.0795	-46.7954
ZINC09462480	-48.5719	-46.7907
ZINC09462480	-45.6149	-46.7907
ZINC04067035	-42.9322	-46.7903
ZINC04067035	-42.6776	-46.7903
ZINC08441051	-42.4651	-46.7867
ZINC08441051	-36.7112	-46.7867
ZINC08442050	-44.4830	-46.7849
ZINC08442050	-42.4243	-46.7849
ZINC08442050	-42.0220	-46.7849

ZINC05360299	-51.6340	-46.7829
ZINC08441547	-41.1797	-46.7828
ZINC08441547	-41.1470	-46.7828
ZINC08441547	-39.1311	-46.7828
ZINC08433357	-46.8389	-46.7741
ZINC08433357	-46.3727	-46.7741
ZINC04067033	-44.0510	-46.7718
ZINC04067033	-43.0619	-46.7718
ZINC04067033	-40.0595	-46.7718
ZINC12523688	-44.3986	-46.7716
ZINC08413210	-44.5636	-46.7673
ZINC08413210	-44.2364	-46.7673
ZINC08442197	-41.9117	-46.7640
ZINC20031586	-43.5914	-46.7638
ZINC20031586	-42.3975	-46.7638
ZINC04067821	-41.2846	-46.7638
ZINC04067821	-41.0698	-46.7638
ZINC08429930	-40.9128	-46.7628
ZINC08441062	-45.6293	-46.7611
ZINC08441062	-44.5768	-46.7611
ZINC08996386	-43.2332	-46.7610
ZINC04629162	-40.7911	-46.7588
ZINC04629162	-37.3676	-46.7588
ZINC04123601	-41.0620	-46.7573
ZINC06195643	-41.8641	-46.7572
ZINC06195643	-36.3331	-46.7572
ZINC13590324	-45.5763	-46.7565
ZINC04066070	-41.7310	-46.7558
ZINC04066070	-40.3788	-46.7558
ZINC05918769	-48.0782	-46.7547
ZINC00844253	-45.4167	-46.7540
ZINC00844253	-41.2230	-46.7540
ZINC04067917	-41.9549	-46.7533
ZINC08416016	-41.5723	-46.7525
ZINC08695244	-48.2762	-46.7514
ZINC04067387	-42.4102	-46.7501
ZINC04067387	-41.5556	-46.7501
ZINC04067387	-40.7456	-46.7501
ZINC08904986	-42.7292	-46.7487
ZINC08904986	-40.3327	-46.7487
ZINC08969911	-43.1346	-46.7481
ZINC06148893	-41.9765	-46.7439
ZINC04068126	-42.4261	-46.7438
ZINC04068126	-42.3147	-46.7438
ZINC19781806	-36.8846	-46.7438
ZINC19781806	-36.3013	-46.7438
ZINC00708042	-42.0801	-46.7393
ZINC08413595	-42.5031	-46.7332
ZINC08413595	-39.1302	-46.7332
ZINC23116712	-40.9510	-46.7325
ZINC04068116	-42.8670	-46.7315
ZINC04068116	-41.4686	-46.7315
ZINC04068116	-41.3827	-46.7315
ZINC03889998	-42.9886	-46.7259

ZINC03889998	-40.4726	-46.7259
ZINC08972680	-38.8773	-46.7235
ZINC04067684	-42.4461	-46.7187
ZINC04067684	-41.9157	-46.7187
ZINC04067684	-41.2110	-46.7187
ZINC08441535	-44.5079	-46.7143
ZINC71414407	-41.4138	-46.7105
ZINC08416020	-42.7974	-46.7072
ZINC12844990	-42.8980	-46.7036
ZINC00702684	-43.5658	-46.6993
ZINC00702684	-42.8529	-46.6993
ZINC19872547	-40.2528	-46.6971
ZINC08996367	-48.4901	-46.6961
ZINC08996367	-46.7715	-46.6961
ZINC04066243	-45.4673	-46.6961
ZINC04066243	-43.9406	-46.6961
ZINC04066243	-41.6047	-46.6961
ZINC08437291	-42.8904	-46.6947
ZINC08439466	-49.7066	-46.6936
ZINC08439466	-48.3665	-46.6936
ZINC19360163	-37.2377	-46.6906
ZINC19360163	-37.1770	-46.6906
ZINC20390990	-50.5542	-46.6889
ZINC13634072	-39.6623	-46.6856
ZINC19790685	-42.8437	-46.6853
ZINC19790685	-41.6182	-46.6853
ZINC04067764	-45.1450	-46.6830
ZINC04067764	-42.2292	-46.6830
ZINC08444355	-39.7950	-46.6823
ZINC08444355	-39.1276	-46.6823
ZINC04061436	-39.1595	-46.6762
ZINC04061436	-38.2879	-46.6762
ZINC08439617	-43.9195	-46.6762
ZINC08439617	-42.9988	-46.6762
ZINC00702673	-45.1308	-46.6758
ZINC00702673	-44.4059	-46.6758
ZINC08413177	-42.8904	-46.6741
ZINC08413177	-39.6171	-46.6741
ZINC06195411	-43.6670	-46.6741
ZINC08398606	-46.6592	-46.6720
ZINC09009199	-44.1745	-46.6710
ZINC09009199	-40.5824	-46.6710
ZINC08440763	-41.7448	-46.6692
ZINC08440763	-41.1343	-46.6692
ZINC19359373	-43.9733	-46.6671
ZINC08441060	-41.0316	-46.6656
ZINC13896934	-42.5632	-46.6603
ZINC13896934	-39.2581	-46.6603
ZINC36024701	-49.2190	-46.6596
ZINC02768646	-45.6750	-46.6574
ZINC09471299	-42.2652	-46.6571
ZINC09471299	-42.0619	-46.6571
ZINC08440986	-41.8380	-46.6546
ZINC04066174	-42.8855	-46.6534

ZINC19552841	-39.0882	-46.6525
ZINC13958098	-42.7811	-46.6482
ZINC13958098	-42.5528	-46.6482
ZINC13958098	-40.6242	-46.6482
ZINC19781620	-38.3171	-46.6469
ZINC08427634	-45.4483	-46.6440
ZINC02054465	-48.6168	-46.6436
ZINC19872270	-41.4927	-46.6381
ZINC08439607	-42.9540	-46.6355
ZINC08439607	-40.0831	-46.6355
ZINC08433415	-41.6889	-46.6335
ZINC08437270	-42.2354	-46.6326
ZINC00702392	-46.5473	-46.6296
ZINC00702392	-45.4534	-46.6296
ZINC08440710	-41.4443	-46.6292
ZINC04065842	-42.9921	-46.6246
ZINC06144018	-48.3495	-46.6227
ZINC06144018	-48.2264	-46.6227
ZINC06136997	-42.3700	-46.6199
ZINC03666814	-46.5047	-46.6188
ZINC06197281	-41.7972	-46.6176
ZINC08386817	-48.4196	-46.6163
ZINC04066467	-42.7590	-46.6143
ZINC04066467	-42.4353	-46.6143
ZINC17166771	-42.1284	-46.6135
ZINC17166771	-40.0868	-46.6135
ZINC00707610	-41.7922	-46.6107
ZINC00707610	-41.7851	-46.6107
ZINC00707610	-39.6041	-46.6107
ZINC04065653	-42.2445	-46.6106
ZINC04065653	-41.4903	-46.6106
ZINC04065653	-40.8833	-46.6106
ZINC08439502	-48.5350	-46.6082
ZINC04067200	-42.3434	-46.6059
ZINC04067200	-40.7356	-46.6059
ZINC04067200	-40.6555	-46.6059
ZINC09008016	-45.3160	-46.6036
ZINC09008016	-45.1921	-46.6036
ZINC09008016	-44.2003	-46.6036
ZINC09042855	-41.5858	-46.6011
ZINC19370532	-44.6105	-46.6002
ZINC19370532	-41.7749	-46.6002
ZINC08396868	-46.3587	-46.5997
ZINC08441437	-40.7385	-46.5961
ZINC08441437	-38.8731	-46.5961
ZINC08441437	-37.4317	-46.5961
ZINC00708006	-41.6870	-46.5914
ZINC00708006	-38.8755	-46.5914
ZINC00708006	-38.4010	-46.5914
ZINC08384306	-40.7361	-46.5911
ZINC06149904	-42.3488	-46.5829
ZINC08383929	-45.5888	-46.5816
ZINC08435327	-47.2181	-46.5789
ZINC37868271	-48.7903	-46.5786

ZINC19691242	-42.3488	-46.5745
ZINC09008592	-42.4273	-46.5734
ZINC09008592	-41.1379	-46.5734
ZINC13161731	-43.2734	-46.5731
ZINC00702987	-43.0958	-46.5730
ZINC08384125	-43.2258	-46.5653
ZINC04014123	-44.4898	-46.5648
ZINC04014123	-43.7600	-46.5648
ZINC08440493	-41.8843	-46.5635
ZINC09046514	-45.6909	-46.5600
ZINC09046514	-45.0163	-46.5600
ZINC09046514	-44.8622	-46.5600
ZINC09046514	-44.3935	-46.5600
ZINC09046514	-43.3830	-46.5600
ZINC08415561	-44.9047	-46.5596
ZINC08415561	-43.2725	-46.5596
ZINC08415561	-38.3688	-46.5596
ZINC00639903	-44.5609	-46.5513
ZINC19802498	-36.8265	-46.5512
ZINC19938484	-46.3265	-46.5502
ZINC08416200	-40.8788	-46.5491
ZINC06148754	-43.1456	-46.5478
ZINC04067391	-43.8555	-46.5462
ZINC00625531	-42.0471	-46.5447
ZINC00625531	-41.3860	-46.5447
ZINC08440858	-40.9428	-46.5423
ZINC08440858	-40.7863	-46.5423
ZINC02183640	-39.7192	-46.5413
ZINC08439369	-45.1491	-46.5411
ZINC08439369	-44.8788	-46.5411
ZINC08439369	-44.2960	-46.5411
ZINC08442074	-49.9453	-46.5410
ZINC08442074	-49.1582	-46.5410
ZINC06195979	-43.9337	-46.5381
ZINC08411646	-44.7690	-46.5331
ZINC08411646	-44.2470	-46.5331
ZINC19731711	-43.7139	-46.5295
ZINC08439630	-42.7677	-46.5247
ZINC08439630	-41.2729	-46.5247
ZINC00998839	-43.6359	-46.5206
ZINC06137345	-41.1350	-46.5205
ZINC08439410	-44.8068	-46.5191
ZINC08439410	-41.7694	-46.5191
ZINC08439410	-41.1645	-46.5191
ZINC08409955	-43.5913	-46.5185
ZINC08409955	-41.0233	-46.5185
ZINC06137186	-42.3392	-46.5129
ZINC06137186	-42.1352	-46.5129
ZINC09046918	-43.9397	-46.5107
ZINC06195487	-43.8963	-46.5052
ZINC04065966	-43.1453	-46.5042
ZINC08439421	-43.3257	-46.5024
ZINC08439421	-41.9894	-46.5024
ZINC08439421	-41.8742	-46.5024

ZINC08383758	-48.6894	-46.5023
ZINC00702448	-43.7029	-46.5023
ZINC00702448	-42.6399	-46.5023
ZINC00702448	-42.3179	-46.5023
ZINC00702448	-41.5583	-46.5023
ZINC00702448	-41.4277	-46.5023
ZINC00792888	-40.1319	-46.5011
ZINC04062856	-41.8751	-46.5001
ZINC13510160	-44.5534	-46.4985
ZINC02055873	-44.7734	-46.4984
ZINC06195671	-41.4328	-46.4971
ZINC07616287	-42.0398	-46.4954
ZINC04065344	-42.8621	-46.4942
ZINC04065344	-42.7401	-46.4942
ZINC04065344	-42.7066	-46.4942
ZINC08439462	-46.2464	-46.4933
ZINC08439462	-43.5422	-46.4933
ZINC08399005	-41.3246	-46.4925
ZINC08384322	-44.7383	-46.4924
ZINC08399989	-43.1716	-46.4924
ZINC08399989	-41.4301	-46.4924
ZINC08399989	-39.1320	-46.4924
ZINC06196434	-45.2352	-46.4903
ZINC08397410	-41.2416	-46.4829
ZINC08397410	-41.2378	-46.4829
ZINC08397410	-40.4358	-46.4829
ZINC00702681	-46.2253	-46.4791
ZINC00702681	-43.5029	-46.4791
ZINC04066422	-42.2381	-46.4789
ZINC04066422	-42.0048	-46.4789
ZINC16115618	-41.5346	-46.4711
ZINC16115618	-37.1930	-46.4711
ZINC06162417	-43.4385	-46.4688
ZINC08440820	-41.5901	-46.4685
ZINC08440820	-40.4613	-46.4685
ZINC08429916	-45.7793	-46.4678
ZINC08426819	-43.7190	-46.4657
ZINC08426819	-43.1200	-46.4657
ZINC08426819	-42.6886	-46.4657
ZINC06195455	-46.8236	-46.4633
ZINC06195455	-45.8245	-46.4633
ZINC00625974	-48.9509	-46.4623
ZINC08441743	-41.4905	-46.4607
ZINC08439475	-40.1941	-46.4604
ZINC08439475	-39.1629	-46.4604
ZINC08439475	-38.5793	-46.4604
ZINC06195646	-44.5243	-46.4529
ZINC06195646	-41.2287	-46.4529
ZINC08425209	-43.8685	-46.4523
ZINC15015750	-44.6314	-46.4520
ZINC06194434	-41.8874	-46.4508
ZINC06194434	-40.1185	-46.4508
ZINC08438730	-43.0392	-46.4435
ZINC00629951	-44.5297	-46.4422

ZINC06015239	-41.6473	-46.4412
ZINC08442511	-41.6011	-46.4390
ZINC08442511	-40.0888	-46.4390
ZINC09271584	-41.1297	-46.4374
ZINC09271584	-41.0684	-46.4374
ZINC09487345	-41.8194	-46.4222
ZINC08399180	-42.2123	-46.4201
ZINC08399180	-40.3921	-46.4201
ZINC08399180	-39.7118	-46.4201
ZINC00728001	-46.6479	-46.4201
ZINC00728001	-46.1651	-46.4201
ZINC04067632	-43.8167	-46.4186
ZINC04067632	-42.4011	-46.4186
ZINC04067632	-41.8289	-46.4186
ZINC04074989	-43.9011	-46.4180
ZINC04074989	-41.4310	-46.4180
ZINC00707959	-41.5465	-46.4161
ZINC00707959	-40.9231	-46.4161
ZINC00707959	-39.4904	-46.4161
ZINC06137350	-39.8517	-46.4155
ZINC00850551	-45.4569	-46.4137
ZINC00678577	-43.4487	-46.4136
ZINC08442370	-43.8866	-46.4073
ZINC15952854	-48.7414	-46.4067
ZINC08893119	-44.6704	-46.4060
ZINC08893119	-44.3447	-46.4060
ZINC04068011	-41.1810	-46.4060
ZINC06160634	-41.7656	-46.4011
ZINC08399141	-45.7245	-46.3998
ZINC08399141	-44.1934	-46.3998
ZINC13124609	-44.5869	-46.3974
ZINC04066274	-42.9629	-46.3934
ZINC04066274	-42.4922	-46.3934
ZINC04065132	-41.3385	-46.3914
ZINC04065132	-40.9691	-46.3914
ZINC06196542	-43.6482	-46.3889
ZINC19360190	-38.8787	-46.3886
ZINC19360190	-36.2700	-46.3886
ZINC09089962	-44.8442	-46.3883
ZINC00703098	-41.8962	-46.3852
ZINC00703098	-37.9694	-46.3852
ZINC08416258	-43.8763	-46.3760
ZINC08416258	-42.9909	-46.3760
ZINC08416258	-42.0067	-46.3760
ZINC19832254	-44.0708	-46.3754
ZINC08432406	-42.0483	-46.3714
ZINC08432406	-40.0676	-46.3714
ZINC19815158	-38.2709	-46.3697
ZINC19815158	-36.8154	-46.3697
ZINC55552105	-38.8801	-46.3682
ZINC08896619	-41.8915	-46.3660
ZINC18244239	-41.8361	-46.3658
ZINC00703058	-43.4179	-46.3640
ZINC00678544	-45.3735	-46.3590

ZINC02079746	-38.7121	-46.3568
ZINC06136919	-44.0407	-46.3514
ZINC68752434	-40.1071	-46.3502
ZINC08426105	-41.9471	-46.3492
ZINC08426105	-41.6490	-46.3492
ZINC08426105	-40.7030	-46.3492
ZINC04418301	-44.2332	-46.3451
ZINC19366919	-41.7318	-46.3438
ZINC00729268	-37.0028	-46.3438
ZINC04067710	-43.7959	-46.3431
ZINC04067710	-43.3452	-46.3431
ZINC00726655	-41.4607	-46.3409
ZINC00726655	-41.0110	-46.3409
ZINC06162046	-41.0911	-46.3403
ZINC00847630	-42.4430	-46.3281
ZINC00847630	-41.9403	-46.3281
ZINC08715717	-45.8139	-46.3275
ZINC08715717	-44.6891	-46.3275
ZINC00702930	-44.0904	-46.3260
ZINC00728567	-41.0224	-46.3238
ZINC00728567	-40.4438	-46.3238
ZINC19551587	-42.3171	-46.3189
ZINC19551587	-37.4525	-46.3189
ZINC37197667	-42.8710	-46.3189
ZINC04060240	-44.9388	-46.3180
ZINC04060240	-41.0457	-46.3180
ZINC00642406	-47.4063	-46.3133
ZINC09456663	-44.1015	-46.3103
ZINC09456663	-43.9785	-46.3103
ZINC08399161	-41.0262	-46.3096
ZINC08399161	-39.5002	-46.3096
ZINC08399161	-39.0560	-46.3096
ZINC09271295	-42.3691	-46.3055
ZINC85425787	-43.7790	-46.3044
ZINC04614562	-40.1104	-46.3031
ZINC08439835	-42.0442	-46.3009
ZINC09312575	-48.6858	-46.2976
ZINC09312575	-46.0248	-46.2976
ZINC09312575	-44.1659	-46.2976
ZINC04065662	-41.0213	-46.2944
ZINC04065662	-40.3464	-46.2944
ZINC04065662	-38.8418	-46.2944
ZINC08437279	-41.6281	-46.2940
ZINC08437279	-38.5799	-46.2940
ZINC08440886	-46.3500	-46.2931
ZINC00726682	-37.9953	-46.2922
ZINC19361806	-42.2259	-46.2908
ZINC04067425	-42.9152	-46.2895
ZINC02092036	-41.2877	-46.2854
ZINC02092036	-36.7172	-46.2854
ZINC08426808	-43.0657	-46.2828
ZINC08426808	-42.3649	-46.2828
ZINC06197571	-40.4168	-46.2827
ZINC06197571	-38.3861	-46.2827

ZINC19794744	-42.3041	-46.2815
ZINC08440934	-46.3627	-46.2814
ZINC12468405	-46.5539	-46.2743
ZINC12468405	-44.2696	-46.2743
ZINC04064840	-42.1062	-46.2721
ZINC04064840	-41.5914	-46.2721
ZINC04067031	-39.9863	-46.2711
ZINC04067031	-39.9336	-46.2711
ZINC04067031	-38.2758	-46.2711
ZINC38139089	-44.6040	-46.2648
ZINC20264072	-45.8641	-46.2632
ZINC06197218	-43.6603	-46.2628
ZINC06197218	-43.5286	-46.2628
ZINC08384605	-45.9565	-46.2608
ZINC00726597	-41.0790	-46.2573
ZINC00726597	-39.2042	-46.2573
ZINC00726597	-38.8141	-46.2573
ZINC09424860	-46.0512	-46.2568
ZINC13055570	-43.7083	-46.2551
ZINC08442178	-41.1190	-46.2547
ZINC08691890	-44.6153	-46.2537
ZINC08400597	-45.7534	-46.2522
ZINC00703003	-45.3511	-46.2515
ZINC32616971	-43.3664	-46.2504
ZINC08439588	-42.3549	-46.2479
ZINC08439588	-41.6656	-46.2479
ZINC08383655	-45.0087	-46.2462
ZINC13370638	-49.9177	-46.2455
ZINC06015552	-41.8934	-46.2444
ZINC09302189	-43.6596	-46.2444
ZINC13372064	-42.2527	-46.2429
ZINC13372064	-39.7954	-46.2429
ZINC19360125	-37.6094	-46.2421
ZINC19360125	-37.5170	-46.2421
ZINC00844250	-41.4341	-46.2419
ZINC00844250	-41.0787	-46.2419
ZINC00844250	-39.7446	-46.2419
ZINC08996239	-41.0157	-46.2400
ZINC08996239	-40.2575	-46.2400
ZINC00703125	-41.8917	-46.2371
ZINC19908677	-41.3734	-46.2335
ZINC19908677	-41.2998	-46.2335
ZINC19359997	-41.0347	-46.2311
ZINC19359997	-39.1171	-46.2311
ZINC06197290	-42.2966	-46.2303
ZINC06015480	-43.1509	-46.2290
ZINC18209804	-44.7079	-46.2289
ZINC02729092	-42.2439	-46.2281
ZINC02729092	-39.6641	-46.2281
ZINC04112198	-41.7597	-46.2262
ZINC04112198	-40.9013	-46.2262
ZINC17242211	-45.7836	-46.2251
ZINC17242211	-44.1998	-46.2251
ZINC15931827	-40.9339	-46.2246

ZINC00724037	-46.1195	-46.2185
ZINC00724037	-44.6664	-46.2185
ZINC08398850	-44.5919	-46.2184
ZINC08425428	-42.3152	-46.2160
ZINC00725583	-44.2017	-46.2157
ZINC08437229	-42.5133	-46.2128
ZINC08437229	-40.6858	-46.2128
ZINC07078917	-36.4330	-46.2093
ZINC00703118	-44.2075	-46.2088
ZINC06015324	-47.3474	-46.2008
ZINC06015324	-47.2532	-46.2008
ZINC06015324	-46.4342	-46.2008
ZINC08439678	-45.3932	-46.2006
ZINC08384659	-45.7579	-46.1997
ZINC05433234	-47.1788	-46.1941
ZINC08817360	-41.6539	-46.1935
ZINC00828780	-45.5771	-46.1932
ZINC06194087	-41.4905	-46.1926
ZINC13563598	-53.7348	-46.1905
ZINC00973050	-42.9975	-46.1890
ZINC09462515	-44.3599	-46.1862
ZINC09462515	-43.9635	-46.1862
ZINC09462515	-43.6744	-46.1862
ZINC09462515	-43.2003	-46.1862
ZINC09462515	-42.8434	-46.1862
ZINC19832197	-41.9099	-46.1838
ZINC19899177	-44.6518	-46.1832
ZINC19899177	-42.3446	-46.1832
ZINC19899177	-41.2177	-46.1832
ZINC00710405	-37.7771	-46.1829
ZINC00710405	-37.6628	-46.1829
ZINC00710405	-37.4716	-46.1829
ZINC00349940	-39.1657	-46.1813
ZINC09359472	-43.4264	-46.1808
ZINC09359472	-41.8303	-46.1808
ZINC04066099	-41.4906	-46.1795
ZINC04066099	-39.6502	-46.1795
ZINC00180995	-40.9589	-46.1786
ZINC06195673	-43.9318	-46.1775
ZINC06195673	-37.8496	-46.1775
ZINC08438763	-49.0251	-46.1772
ZINC08438763	-47.4898	-46.1772
ZINC08439504	-45.4043	-46.1759
ZINC08439504	-45.3581	-46.1759
ZINC09379916	-43.8438	-46.1709
ZINC09361247	-44.1325	-46.1624
ZINC08444353	-41.4137	-46.1621
ZINC08444353	-40.2407	-46.1621
ZINC06162108	-42.9908	-46.1556
ZINC08441444	-40.2971	-46.1551
ZINC08441444	-38.2521	-46.1551
ZINC04112114	-43.2504	-46.1541
ZINC04112114	-42.4973	-46.1541
ZINC04112114	-39.6932	-46.1541

ZINC08440098	-46.5283	-46.1535
ZINC08440098	-42.1642	-46.1535
ZINC00726612	-40.5634	-46.1521
ZINC00726612	-39.7918	-46.1521
ZINC00726612	-38.7743	-46.1521
ZINC08450294	-43.1210	-46.1496
ZINC08743643	-45.4177	-46.1493
ZINC19802215	-37.9396	-46.1475
ZINC08440926	-45.0589	-46.1463
ZINC19364243	-41.4445	-46.1440
ZINC06137145	-43.1564	-46.1436
ZINC18077530	-52.0851	-46.1408
ZINC18077530	-51.1960	-46.1408
ZINC18077530	-47.0704	-46.1408
ZINC18077530	-46.7881	-46.1408
ZINC06195457	-40.9031	-46.1397
ZINC13532878	-42.7784	-46.1383
ZINC09354151	-42.3636	-46.1380
ZINC09354151	-39.6785	-46.1380
ZINC08430085	-42.9565	-46.1350
ZINC08439649	-42.7595	-46.1350
ZINC08439649	-41.6201	-46.1350
ZINC08439649	-40.9031	-46.1350
ZINC04067732	-42.6239	-46.1318
ZINC04067732	-41.3699	-46.1318
ZINC08441837	-42.8678	-46.1268
ZINC18213356	-40.7110	-46.1250
ZINC18213356	-38.3152	-46.1250
ZINC18028589	-43.4732	-46.1233
ZINC18028589	-42.8470	-46.1233
ZINC20176051	-41.9610	-46.1182
ZINC08440935	-47.4234	-46.1143
ZINC06197134	-41.4680	-46.1106
ZINC13120287	-46.4621	-46.1072
ZINC08415575	-42.1201	-46.1065
ZINC08415575	-37.5531	-46.1065
ZINC08415575	-36.9234	-46.1065
ZINC06162037	-42.1610	-46.1040
ZINC04391243	-41.8154	-46.0999
ZINC06197289	-42.8523	-46.0981
ZINC06197289	-39.0876	-46.0981
ZINC17154960	-41.0831	-46.0902
ZINC17154960	-37.9547	-46.0902
ZINC17154960	-37.8505	-46.0902
ZINC09046271	-41.0840	-46.0879
ZINC09088329	-43.5829	-46.0868
ZINC08437254	-41.2387	-46.0840
ZINC06187105	-41.2799	-46.0804
ZINC02055291	-43.6169	-46.0753
ZINC04059656	-42.4514	-46.0749
ZINC08437469	-42.8792	-46.0717
ZINC08437469	-41.8410	-46.0717
ZINC08437469	-41.0340	-46.0717
ZINC06745855	-41.6214	-46.0709

ZINC00998635	-43.5490	-46.0705
ZINC02071241	-47.5333	-46.0697
ZINC00659307	-45.1717	-46.0693
ZINC09374792	-42.6586	-46.0693
ZINC08441962	-46.7429	-46.0669
ZINC08441962	-46.1443	-46.0669
ZINC08441962	-41.7760	-46.0669
ZINC20032122	-45.1606	-46.0665
ZINC08440575	-45.2808	-46.0645
ZINC06273700	-44.0674	-46.0638
ZINC06273700	-43.5045	-46.0638
ZINC06273700	-42.6425	-46.0638
ZINC19909180	-40.9499	-46.0603
ZINC17167378	-45.1688	-46.0601
ZINC09374529	-43.8860	-46.0601
ZINC17166908	-45.3466	-46.0599
ZINC17166908	-43.1033	-46.0599
ZINC17166908	-42.9925	-46.0599
ZINC08396650	-48.8560	-46.0587
ZINC08437246	-45.1047	-46.0578
ZINC08440365	-42.4480	-46.0554
ZINC08440365	-38.2238	-46.0554
ZINC06136932	-44.4667	-46.0547
ZINC17196736	-44.8616	-46.0545
ZINC06196096	-44.9189	-46.0535
ZINC17992648	-43.4120	-46.0517
ZINC00823619	-41.8288	-46.0505
ZINC04066062	-42.5601	-46.0502
ZINC04649901	-42.7919	-46.0502
ZINC04067650	-43.6107	-46.0454
ZINC04067650	-41.1355	-46.0454
ZINC04067650	-41.0663	-46.0454
ZINC00702674	-46.4318	-46.0400
ZINC00702674	-46.0501	-46.0400
ZINC19781787	-42.0911	-46.0354
ZINC19781787	-40.9830	-46.0354
ZINC06197431	-45.5973	-46.0336
ZINC06197431	-40.5664	-46.0336
ZINC05360685	-49.8377	-46.0314
ZINC04067379	-43.9795	-46.0313
ZINC04067379	-42.4243	-46.0313
ZINC08439513	-47.5693	-46.0289
ZINC04060741	-42.3204	-46.0276
ZINC04060741	-40.8225	-46.0276
ZINC09313261	-46.6163	-46.0265
ZINC09313261	-46.2544	-46.0265
ZINC04066589	-42.2016	-46.0247
ZINC04066589	-40.9418	-46.0247
ZINC04019554	-41.9569	-46.0230
ZINC04019554	-39.2626	-46.0230
ZINC08439479	-44.1017	-46.0221
ZINC08439479	-43.5281	-46.0221
ZINC08439479	-41.4910	-46.0221
ZINC00726613	-43.7155	-46.0219

ZINC00726613	-43.2816	-46.0219
ZINC00726613	-42.4595	-46.0219
ZINC09086302	-44.6374	-46.0201
ZINC09086302	-42.9610	-46.0201
ZINC04163045	-38.4981	-46.0195
ZINC04065819	-39.4415	-46.0174
ZINC04065819	-37.6695	-46.0174
ZINC05918698	-47.7443	-46.0122
ZINC08431354	-46.9737	-46.0098
ZINC06195461	-42.3170	-46.0056
ZINC06195461	-41.7703	-46.0056
ZINC17154951	-39.3999	-46.0048
ZINC17154951	-38.4439	-46.0048
ZINC17154951	-37.4518	-46.0048
ZINC08439503	-41.9074	-46.0045
ZINC08440728	-39.5313	-46.0041
ZINC06194582	-40.2371	-46.0038
ZINC13127256	-44.1179	-46.0036
ZINC04559915	-42.9871	-46.0028
ZINC09007983	-43.0257	-45.9957
ZINC09007983	-41.8031	-45.9957
ZINC09007983	-39.8678	-45.9957
ZINC09007983	-38.9619	-45.9957
ZINC19555377	-39.0747	-45.9945
ZINC19555377	-39.0359	-45.9945
ZINC08442183	-41.8123	-45.9933
ZINC08442183	-36.4444	-45.9933
ZINC00679374	-44.2287	-45.9918
ZINC06444324	-45.8344	-45.9797
ZINC06444324	-42.9083	-45.9797
ZINC00726688	-44.2426	-45.9784
ZINC00726688	-42.4350	-45.9784
ZINC00726688	-38.9779	-45.9784
ZINC04068147	-42.5554	-45.9781
ZINC08441065	-40.2895	-45.9776
ZINC08441065	-40.2060	-45.9776
ZINC06149675	-43.9164	-45.9769
ZINC06149675	-39.8364	-45.9769
ZINC04065950	-41.6433	-45.9767
ZINC04065950	-40.9499	-45.9767
ZINC08438776	-45.9548	-45.9761
ZINC08438776	-44.8883	-45.9761
ZINC00350361	-39.0042	-45.9737
ZINC18009992	-44.3773	-45.9729
ZINC18009992	-40.4900	-45.9729
ZINC09065886	-48.7365	-45.9706
ZINC08439492	-51.5501	-45.9679
ZINC08439492	-47.2988	-45.9679
ZINC00823704	-37.1316	-45.9633
ZINC00722083	-43.8556	-45.9616
ZINC00722083	-41.3705	-45.9616
ZINC08741467	-47.6036	-45.9615
ZINC08741467	-46.3084	-45.9615
ZINC09271585	-41.4810	-45.9609

ZINC00726478	-38.1523	-45.9606
ZINC08437262	-41.6640	-45.9592
ZINC00702947	-43.7137	-45.9552
ZINC08416151	-40.5595	-45.9551
ZINC08416151	-39.3702	-45.9551
ZINC08416151	-38.0934	-45.9551
ZINC00709904	-40.8313	-45.9546
ZINC00709904	-40.7664	-45.9546
ZINC08441298	-50.3417	-45.9451
ZINC08441298	-48.1905	-45.9451
ZINC19872050	-44.4091	-45.9447
ZINC04067190	-44.7839	-45.9446
ZINC04067190	-44.0234	-45.9446
ZINC06196092	-44.8890	-45.9424
ZINC06197443	-42.3984	-45.9416
ZINC06197443	-41.0249	-45.9416
ZINC08400434	-43.1209	-45.9408
ZINC08400434	-39.1877	-45.9408
ZINC20036528	-41.2685	-45.9376
ZINC09374527	-46.7885	-45.9342
ZINC00678579	-46.1349	-45.9213
ZINC00703114	-43.5373	-45.9198
ZINC08441573	-43.1630	-45.9194
ZINC08441573	-42.8505	-45.9194
ZINC00628998	-43.6718	-45.9175
ZINC00628998	-43.2574	-45.9175
ZINC09043841	-43.6064	-45.9172
ZINC09043841	-41.3864	-45.9172
ZINC04067401	-43.4527	-45.9172
ZINC04067401	-41.6855	-45.9172
ZINC00726520	-37.7246	-45.9156
ZINC00726520	-36.2887	-45.9156
ZINC06195435	-43.2581	-45.9141
ZINC06195435	-41.3954	-45.9141
ZINC08425420	-42.5296	-45.9106
ZINC02064472	-50.3049	-45.9069
ZINC09123183	-42.9093	-45.9047
ZINC09123183	-40.8131	-45.9047
ZINC08440085	-41.6177	-45.9043
ZINC19832126	-37.2046	-45.8980
ZINC08439484	-46.5251	-45.8962
ZINC08439484	-46.5029	-45.8962
ZINC08439484	-41.3990	-45.8962
ZINC06136874	-43.4747	-45.8941
ZINC04067373	-44.4120	-45.8918
ZINC04681481	-41.8806	-45.8917
ZINC09312126	-44.4710	-45.8868
ZINC08996344	-40.6359	-45.8794
ZINC08430382	-39.5979	-45.8793
ZINC06668085	-42.7076	-45.8790
ZINC06668085	-41.0979	-45.8790
ZINC08433345	-44.3804	-45.8782
ZINC08441303	-41.3677	-45.8775
ZINC08441303	-39.9594	-45.8775

ZINC04068137	-39.7234	-45.8753
ZINC08399164	-41.7677	-45.8746
ZINC08399164	-41.1479	-45.8746
ZINC08399164	-39.9209	-45.8746
ZINC00848094	-39.8821	-45.8720
ZINC08397163	-42.1890	-45.8694
ZINC08397163	-41.4839	-45.8694
ZINC02135498	-42.2902	-45.8681
ZINC15952861	-46.7723	-45.8660
ZINC08413096	-41.7241	-45.8633
ZINC08413096	-41.1521	-45.8633
ZINC08440983	-41.1976	-45.8621
ZINC08440983	-38.8609	-45.8621
ZINC08441123	-41.0546	-45.8589
ZINC08441123	-40.5303	-45.8589
ZINC08441123	-40.0466	-45.8589
ZINC04066222	-42.3355	-45.8562
ZINC04066222	-41.1985	-45.8562
ZINC08426811	-43.8633	-45.8541
ZINC19830677	-43.4487	-45.8535
ZINC03666559	-46.9430	-45.8524
ZINC08440058	-40.9300	-45.8506
ZINC08440058	-40.4001	-45.8506
ZINC08440058	-38.3251	-45.8506
ZINC00726675	-41.7632	-45.8505
ZINC09312125	-44.7858	-45.8488
ZINC09122096	-46.4993	-45.8485
ZINC09122096	-43.7587	-45.8485
ZINC09122096	-43.0288	-45.8485
ZINC00626568	-43.5686	-45.8476
ZINC00626568	-41.8961	-45.8476
ZINC05285969	-44.9684	-45.8471
ZINC08695240	-48.2977	-45.8439
ZINC19943713	-40.3841	-45.8433
ZINC19943713	-38.6944	-45.8433
ZINC09350695	-50.4301	-45.8429
ZINC09350695	-42.1920	-45.8429
ZINC00625913	-41.7936	-45.8351
ZINC19369476	-40.2634	-45.8342
ZINC19369476	-38.4897	-45.8342
ZINC00702588	-43.8159	-45.8329
ZINC08440570	-42.1712	-45.8309
ZINC04066218	-39.6009	-45.8306
ZINC04066218	-37.7684	-45.8306
ZINC08440120	-43.9158	-45.8302
ZINC08440120	-42.2871	-45.8302
ZINC06197159	-43.4972	-45.8287
ZINC04019577	-41.2481	-45.8267
ZINC04019577	-40.4786	-45.8267
ZINC00626153	-43.4326	-45.8264
ZINC16115455	-40.2428	-45.8231
ZINC00725557	-45.4573	-45.8217
ZINC13231931	-44.6001	-45.8209
ZINC00844251	-42.6768	-45.8163

ZINC00844251	-42.4452	-45.8163
ZINC00844251	-42.4341	-45.8163
ZINC08413561	-40.7854	-45.8156
ZINC08413561	-39.6848	-45.8156
ZINC08413561	-39.2944	-45.8156
ZINC02736309	-46.9220	-45.8149
ZINC04418095	-42.1240	-45.8064
ZINC02756072	-41.9548	-45.8047
ZINC08416198	-42.1518	-45.8029
ZINC04067948	-45.8880	-45.8027
ZINC04067948	-43.1060	-45.8027
ZINC04065939	-42.5787	-45.8010
ZINC04065939	-40.9437	-45.8010
ZINC09086142	-42.0464	-45.7995
ZINC09086142	-41.0730	-45.7995
ZINC13282401	-43.0331	-45.7989
ZINC13282401	-41.1277	-45.7989
ZINC08440796	-41.1830	-45.7986
ZINC09043845	-44.8659	-45.7955
ZINC09043845	-44.1440	-45.7955
ZINC08416059	-39.5153	-45.7912
ZINC08416059	-38.3720	-45.7912
ZINC05490362	-39.8872	-45.7862
ZINC04417590	-44.9812	-45.7851
ZINC08416321	-40.4513	-45.7832
ZINC04065055	-38.2278	-45.7828
ZINC13552843	-43.7787	-45.7820
ZINC08439888	-40.1971	-45.7808
ZINC08439888	-37.2454	-45.7808
ZINC09071899	-43.2631	-45.7775
ZINC09185694	-42.0603	-45.7750
ZINC08396757	-45.7678	-45.7736
ZINC00626131	-41.1949	-45.7705
ZINC19938520	-45.8004	-45.7677
ZINC08440889	-45.6767	-45.7656
ZINC08441564	-39.0078	-45.7653
ZINC17179592	-42.3184	-45.7645
ZINC08742874	-43.7816	-45.7639
ZINC08399482	-42.8511	-45.7631
ZINC08399482	-38.3251	-45.7631
ZINC04067766	-44.5198	-45.7631
ZINC04067766	-44.1409	-45.7631
ZINC04067766	-43.2445	-45.7631
ZINC08444666	-43.4896	-45.7610
ZINC08444666	-42.4008	-45.7610
ZINC06148867	-41.1746	-45.7587
ZINC05918590	-46.2166	-45.7564
ZINC05918590	-44.8183	-45.7564
ZINC19361365	-42.5039	-45.7561
ZINC08384559	-45.8145	-45.7549
ZINC08441313	-43.7119	-45.7546
ZINC08441313	-41.6894	-45.7546
ZINC04067865	-42.8151	-45.7506
ZINC04067865	-42.4197	-45.7506

ZINC04067865	-41.4481	-45.7506
ZINC09273838	-41.4191	-45.7482
ZINC09273838	-39.6350	-45.7482
ZINC20624329	-46.6064	-45.7446
ZINC16944327	-44.5112	-45.7430
ZINC06162480	-43.2441	-45.7429
ZINC09311925	-41.2168	-45.7416
ZINC02183677	-39.8204	-45.7401
ZINC00702444	-42.4262	-45.7371
ZINC00702444	-41.6595	-45.7371
ZINC19832097	-38.2772	-45.7364
ZINC06015290	-45.1400	-45.7354
ZINC06015290	-43.6035	-45.7354
ZINC06015290	-43.2439	-45.7354
ZINC08440512	-42.6699	-45.7353
ZINC00970964	-41.4828	-45.7341
ZINC06194522	-42.3093	-45.7324
ZINC06194522	-40.7534	-45.7324
ZINC05737807	-46.7061	-45.7316
ZINC04066067	-44.9354	-45.7308
ZINC04066067	-43.6916	-45.7308
ZINC08439949	-41.2877	-45.7304
ZINC04066588	-42.2892	-45.7300
ZINC09240497	-41.4937	-45.7295
ZINC08442211	-41.7523	-45.7267
ZINC06445732	-41.4529	-45.7266
ZINC06194581	-44.4847	-45.7194
ZINC06194581	-41.0583	-45.7194
ZINC20546847	-45.8960	-45.7178
ZINC08384188	-42.6186	-45.7161
ZINC08441763	-43.5265	-45.7151
ZINC06137404	-42.0607	-45.7136
ZINC08441133	-50.0371	-45.7112
ZINC09425046	-44.9322	-45.7093
ZINC08440117	-42.9867	-45.7093
ZINC08440117	-42.3809	-45.7093
ZINC19781662	-37.0321	-45.7072
ZINC08439416	-41.7139	-45.7050
ZINC08439416	-41.2500	-45.7050
ZINC02057959	-45.4134	-45.7049
ZINC08440737	-37.0877	-45.7042
ZINC08440737	-36.6767	-45.7042
ZINC37857406	-46.0492	-45.7020
ZINC37857406	-43.1087	-45.7020
ZINC04068118	-41.6965	-45.7011
ZINC04068118	-41.4259	-45.7011
ZINC04068118	-40.0923	-45.7011
ZINC18033437	-42.3033	-45.6994
ZINC18033437	-42.2982	-45.6994
ZINC18033437	-41.7763	-45.6994
ZINC08440503	-43.2118	-45.6989
ZINC08440092	-41.8837	-45.6987
ZINC08383353	-41.5668	-45.6984
ZINC19781945	-36.7106	-45.6974

ZINC04065151	-43.1155	-45.6971
ZINC04065151	-37.3657	-45.6971
ZINC08440748	-41.9282	-45.6968
ZINC00702750	-44.7508	-45.6934
ZINC00702750	-40.9853	-45.6934
ZINC17747151	-41.1672	-45.6922
ZINC02478363	-42.7121	-45.6905
ZINC02478363	-42.4843	-45.6905
ZINC02478363	-42.2155	-45.6905
ZINC08440132	-41.1625	-45.6871
ZINC08440132	-40.5789	-45.6871
ZINC08440132	-38.4287	-45.6871
ZINC08441579	-40.7545	-45.6863
ZINC08441579	-37.9340	-45.6863
ZINC04629642	-42.1580	-45.6847
ZINC08431472	-44.4455	-45.6832
ZINC04284874	-43.6810	-45.6812
ZINC04284874	-42.2871	-45.6812
ZINC19922808	-43.6590	-45.6810
ZINC08438666	-44.1882	-45.6797
ZINC08438666	-42.5788	-45.6797
ZINC04418352	-41.6562	-45.6763
ZINC04418352	-40.5671	-45.6763
ZINC08416347	-41.2313	-45.6745
ZINC04060634	-42.5467	-45.6705
ZINC04060634	-40.3269	-45.6705
ZINC09403374	-43.2846	-45.6703
ZINC09403374	-41.9920	-45.6703
ZINC09065246	-41.1759	-45.6671
ZINC09065246	-39.9960	-45.6671
ZINC17143889	-40.2344	-45.6656
ZINC00626146	-41.5426	-45.6651
ZINC19782423	-44.7298	-45.6637
ZINC19782423	-44.6272	-45.6637
ZINC06195491	-42.9407	-45.6623
ZINC06195491	-37.0493	-45.6623
ZINC08429904	-44.2427	-45.6613
ZINC08429904	-40.5568	-45.6613
ZINC08396814	-41.1537	-45.6592
ZINC08396814	-39.4136	-45.6592
ZINC08411139	-44.0692	-45.6575
ZINC05225802	-43.1852	-45.6571
ZINC08440501	-44.2055	-45.6568
ZINC08433356	-42.6127	-45.6563
ZINC08433356	-41.9461	-45.6563
ZINC00717024	-40.2060	-45.6552
ZINC00717024	-39.9751	-45.6552
ZINC00717024	-39.8995	-45.6552
ZINC17242724	-45.7767	-45.6526
ZINC09471294	-43.0995	-45.6519
ZINC08384029	-47.3738	-45.6474
ZINC08384029	-45.3994	-45.6474
ZINC00726516	-42.3733	-45.6473
ZINC00726516	-41.9656	-45.6473

ZINC05918802	-43.6828	-45.6465
ZINC08439414	-42.4549	-45.6406
ZINC08439414	-41.8962	-45.6406
ZINC06137152	-38.6218	-45.6391
ZINC19339153	-40.8702	-45.6355
ZINC09459132	-43.3597	-45.6343
ZINC09459132	-42.5927	-45.6343
ZINC19872377	-39.8700	-45.6327
ZINC08429894	-40.6582	-45.6320
ZINC08429894	-38.5152	-45.6320
ZINC08383712	-45.6010	-45.6311
ZINC08440899	-46.4842	-45.6297
ZINC04649846	-39.6496	-45.6297
ZINC05921386	-43.3508	-45.6289
ZINC05921386	-40.4667	-45.6289
ZINC04067660	-41.4246	-45.6289
ZINC04067660	-38.5581	-45.6289
ZINC06195631	-41.8645	-45.6281
ZINC09059587	-47.6293	-45.6258
ZINC04064598	-46.6962	-45.6231
ZINC04064598	-44.9297	-45.6231
ZINC04064598	-43.5063	-45.6231
ZINC17017034	-44.5022	-45.6224
ZINC04067380	-42.2233	-45.6214
ZINC04067380	-41.9470	-45.6214
ZINC06137425	-41.4725	-45.6170
ZINC02870284	-41.8853	-45.6148
ZINC04062029	-41.8934	-45.6145
ZINC08440912	-47.3075	-45.6093
ZINC06015287	-45.4804	-45.6080
ZINC06015287	-41.7735	-45.6080
ZINC00702989	-42.4944	-45.6045
ZINC08415659	-41.4469	-45.6030
ZINC18022571	-43.5301	-45.5998
ZINC18022571	-41.2457	-45.5998
ZINC00826297	-40.4549	-45.5998
ZINC00826297	-37.4482	-45.5998
ZINC19691198	-40.5284	-45.5979
ZINC05220257	-39.9519	-45.5970
ZINC19872206	-41.0380	-45.5926
ZINC04059705	-41.2126	-45.5924
ZINC04059705	-40.0031	-45.5924
ZINC04059705	-39.5166	-45.5924
ZINC08441812	-41.5228	-45.5906
ZINC09424819	-42.5848	-45.5887
ZINC08411087	-41.2043	-45.5839
ZINC08411087	-40.5502	-45.5839
ZINC13577215	-43.8287	-45.5802
ZINC16677349	-46.8686	-45.5782
ZINC16677349	-46.3274	-45.5782
ZINC08424927	-40.7351	-45.5772
ZINC04391426	-41.6888	-45.5765
ZINC06196402	-43.6708	-45.5736
ZINC22916528	-38.3702	-45.5722

ZINC22916528	-36.3853	-45.5722
ZINC04062344	-42.4425	-45.5712
ZINC04062344	-39.7081	-45.5712
ZINC08440920	-48.1554	-45.5707
ZINC04111961	-38.5773	-45.5703
ZINC04067739	-43.5958	-45.5686
ZINC04067739	-41.5605	-45.5686
ZINC04067739	-41.2347	-45.5686
ZINC00729509	-43.2164	-45.5673
ZINC00729509	-42.8770	-45.5673
ZINC00729509	-42.6304	-45.5673
ZINC00844259	-42.9948	-45.5607
ZINC00844259	-39.0503	-45.5607
ZINC08400644	-43.1094	-45.5594
ZINC04285241	-40.0589	-45.5555
ZINC04019626	-47.8009	-45.5544
ZINC04019626	-47.4703	-45.5544
ZINC04019626	-45.9276	-45.5544
ZINC09313113	-43.8099	-45.5542
ZINC06161977	-41.0141	-45.5538
ZINC08426810	-42.3217	-45.5502
ZINC02181924	-40.6831	-45.5476
ZINC04060739	-40.6597	-45.5374
ZINC04060739	-37.4001	-45.5374
ZINC08425986	-46.0583	-45.5328
ZINC19802263	-41.6390	-45.5320
ZINC19549844	-36.9142	-45.5318
ZINC09458162	-44.2701	-45.5289
ZINC09458162	-42.3191	-45.5289
ZINC08431458	-42.6257	-45.5236
ZINC68736166	-42.6706	-45.5215
ZINC19792944	-48.8362	-45.5183
ZINC19781705	-36.4239	-45.5175
ZINC08440798	-37.8754	-45.5129
ZINC36022873	-46.8115	-45.5110
ZINC08444504	-47.9668	-45.5109
ZINC04417950	-39.4460	-45.5104
ZINC04417950	-38.3253	-45.5104
ZINC04059941	-41.0621	-45.5097
ZINC04059941	-38.4521	-45.5097
ZINC08415758	-39.7691	-45.5067
ZINC06162318	-39.1083	-45.5036
ZINC19872120	-38.9186	-45.5022
ZINC06197641	-41.6366	-45.4974
ZINC04067634	-43.6922	-45.4949
ZINC04067634	-41.3156	-45.4949
ZINC04067634	-40.7982	-45.4949
ZINC08441093	-40.8995	-45.4943
ZINC19371917	-37.4908	-45.4917
ZINC17166810	-41.9714	-45.4876
ZINC17197831	-43.9422	-45.4863
ZINC19940207	-42.4474	-45.4841
ZINC19940207	-42.1725	-45.4841
ZINC08439438	-47.5727	-45.4809

ZINC08439438	-45.5206	-45.4809
ZINC08439438	-44.5906	-45.4809
ZINC00627126	-44.3905	-45.4804
ZINC09186710	-41.8060	-45.4772
ZINC06195630	-42.4054	-45.4753
ZINC06195630	-42.1174	-45.4753
ZINC06197296	-42.8810	-45.4748
ZINC02503571	-41.6302	-45.4747
ZINC02503571	-40.9529	-45.4747
ZINC08429901	-43.8309	-45.4716
ZINC08715760	-37.9616	-45.4681
ZINC08715760	-37.1299	-45.4681
ZINC08399202	-41.6902	-45.4679
ZINC09477310	-46.4890	-45.4676
ZINC09477310	-44.8789	-45.4676
ZINC05504555	-39.3738	-45.4644
ZINC00847634	-43.9980	-45.4630
ZINC00847634	-42.8588	-45.4630
ZINC00847634	-39.0581	-45.4630
ZINC08441127	-44.4172	-45.4608
ZINC00716997	-39.7719	-45.4594
ZINC00716997	-39.2535	-45.4594
ZINC00716997	-38.5167	-45.4594
ZINC00655183	-43.5133	-45.4586
ZINC04126056	-41.2408	-45.4583
ZINC19871853	-44.5203	-45.4558
ZINC04068117	-45.0986	-45.4554
ZINC04068117	-44.5048	-45.4554
ZINC04068117	-44.4352	-45.4554
ZINC04473314	-44.3243	-45.4543
ZINC06136870	-41.7055	-45.4517
ZINC02188296	-41.8713	-45.4514
ZINC19640056	-44.2044	-45.4480
ZINC09271558	-43.5609	-45.4478
ZINC09271558	-42.7145	-45.4478
ZINC00844252	-44.4563	-45.4474
ZINC00844252	-41.9942	-45.4474
ZINC00844252	-40.9184	-45.4474
ZINC04065473	-43.3683	-45.4467
ZINC04065473	-42.2283	-45.4467
ZINC04065473	-41.7947	-45.4467
ZINC08837392	-50.4932	-45.4442
ZINC08837392	-48.9367	-45.4442
ZINC04417958	-40.2141	-45.4370
ZINC04417958	-38.3389	-45.4370
ZINC01413488	-44.2750	-45.4361
ZINC08439544	-46.8773	-45.4339
ZINC08437267	-40.8748	-45.4338
ZINC08437267	-39.8179	-45.4338
ZINC08437267	-37.1794	-45.4338
ZINC08440707	-39.8282	-45.4325
ZINC08440707	-37.9673	-45.4325
ZINC08440707	-37.3536	-45.4325
ZINC68712922	-45.1429	-45.4309

ZINC08440723	-42.3438	-45.4309
ZINC08440723	-39.9982	-45.4309
ZINC08440723	-39.7712	-45.4309
ZINC08397395	-45.9594	-45.4293
ZINC19938433	-46.8429	-45.4246
ZINC08922000	-45.5987	-45.4229
ZINC00970965	-46.5954	-45.4195
ZINC08429971	-44.1142	-45.4174
ZINC08429971	-43.8854	-45.4174
ZINC08440756	-40.8702	-45.4151
ZINC08440756	-38.7480	-45.4151
ZINC08437424	-39.5888	-45.4147
ZINC04066606	-41.9163	-45.4145
ZINC04066606	-40.7679	-45.4145
ZINC22935925	-39.5522	-45.4121
ZINC03878056	-42.2513	-45.4114
ZINC04067914	-40.3430	-45.4092
ZINC05433238	-46.8017	-45.4076
ZINC06148796	-40.1808	-45.4073
ZINC06148796	-37.3833	-45.4073
ZINC08415605	-42.3646	-45.4073
ZINC08415605	-41.8441	-45.4073
ZINC16311777	-39.9344	-45.4064
ZINC19832109	-38.7845	-45.4052
ZINC19832109	-36.9457	-45.4052
ZINC09192696	-43.4761	-45.4050
ZINC09192696	-41.3830	-45.4050
ZINC02055153	-42.8337	-45.4049
ZINC04067512	-42.4027	-45.4035
ZINC04067512	-41.1379	-45.4035
ZINC04067512	-39.4692	-45.4035
ZINC00722296	-43.9018	-45.4021
ZINC09086303	-42.4617	-45.3982
ZINC09086303	-41.9026	-45.3982
ZINC08743942	-45.2411	-45.3971
ZINC04068146	-37.4516	-45.3968
ZINC06015335	-42.5786	-45.3957
ZINC19872294	-39.5179	-45.3955
ZINC04063523	-41.7513	-45.3862
ZINC04063523	-41.2097	-45.3862
ZINC04063523	-40.1352	-45.3862
ZINC04065869	-40.4673	-45.3821
ZINC04065869	-40.3357	-45.3821
ZINC04065869	-39.2670	-45.3821
ZINC02105801	-38.3007	-45.3796
ZINC08972397	-42.3939	-45.3783
ZINC08425084	-43.9859	-45.3723
ZINC08425084	-40.1569	-45.3723
ZINC08431356	-44.7366	-45.3719
ZINC08441681	-40.3483	-45.3710
ZINC09135490	-46.0306	-45.3699
ZINC09135490	-44.1425	-45.3699
ZINC08440387	-43.4987	-45.3679
ZINC08440387	-42.6745	-45.3679

ZINC08440387	-39.8538	-45.3679
ZINC18162084	-41.3941	-45.3674
ZINC04658901	-43.5535	-45.3628
ZINC09311924	-41.3943	-45.3626
ZINC13206167	-47.3539	-45.3610
ZINC32541288	-39.6324	-45.3605
ZINC08415868	-40.3230	-45.3603
ZINC00969334	-38.3225	-45.3579
ZINC19796845	-39.6950	-45.3561
ZINC19796845	-36.8693	-45.3561
ZINC12382604	-42.0255	-45.3553
ZINC08780047	-42.4775	-45.3511
ZINC06197211	-46.2053	-45.3505
ZINC06197211	-41.7980	-45.3505
ZINC00714277	-48.6861	-45.3468
ZINC01413469	-46.8771	-45.3452
ZINC01413469	-42.7195	-45.3452
ZINC08396867	-45.7555	-45.3449
ZINC09329513	-42.0562	-45.3446
ZINC08440271	-40.5494	-45.3440
ZINC08440271	-38.8777	-45.3440
ZINC00729511	-39.6518	-45.3434
ZINC00729511	-39.1363	-45.3434
ZINC00729511	-38.8470	-45.3434
ZINC00625969	-49.1885	-45.3433
ZINC08396871	-45.8511	-45.3413
ZINC08415587	-43.0144	-45.3405
ZINC08415587	-41.8681	-45.3405
ZINC08415587	-38.2773	-45.3405
ZINC04066702	-40.2897	-45.3396
ZINC04066702	-37.4945	-45.3396
ZINC08441131	-40.6956	-45.3393
ZINC08441131	-36.5908	-45.3393
ZINC08441131	-36.2713	-45.3393
ZINC09271991	-40.0894	-45.3383
ZINC04066217	-39.2592	-45.3345
ZINC04066217	-38.9675	-45.3345
ZINC06137503	-39.8456	-45.3314
ZINC08433245	-44.4659	-45.3309
ZINC08441624	-41.0168	-45.3303
ZINC08441624	-38.6860	-45.3303
ZINC04067662	-40.5808	-45.3272
ZINC04067662	-40.4754	-45.3272
ZINC04067662	-40.1572	-45.3272
ZINC19325070	-38.2608	-45.3251
ZINC19325070	-37.7842	-45.3251
ZINC05918873	-51.4285	-45.3203
ZINC05918873	-46.9063	-45.3203
ZINC00726544	-41.9260	-45.3202
ZINC00726544	-39.3289	-45.3202
ZINC00726544	-37.7390	-45.3202
ZINC09271084	-43.7667	-45.3182
ZINC09271084	-41.5771	-45.3182
ZINC08740584	-44.2157	-45.3172

ZINC04780874	-42.3634	-45.3165
ZINC04780874	-38.5959	-45.3165
ZINC04780874	-36.9546	-45.3165
ZINC04067635	-42.9468	-45.3157
ZINC04067635	-41.8762	-45.3157
ZINC04067635	-40.4058	-45.3157
ZINC09110366	-41.8709	-45.3121
ZINC00844153	-41.3538	-45.3121
ZINC00844153	-36.5111	-45.3121
ZINC04068081	-37.7927	-45.3119
ZINC04068081	-36.5156	-45.3119
ZINC00703104	-42.1856	-45.3104
ZINC04059815	-41.3792	-45.3100
ZINC04059815	-41.0515	-45.3100
ZINC04592698	-41.2706	-45.3092
ZINC19360016	-42.3874	-45.3085
ZINC08441576	-39.6697	-45.3078
ZINC08441576	-38.6150	-45.3078
ZINC36646094	-41.9382	-45.3067
ZINC00976957	-45.1239	-45.2966
ZINC06162266	-40.9921	-45.2966
ZINC06162266	-38.3941	-45.2966
ZINC08441063	-43.2735	-45.2916
ZINC08441063	-40.2097	-45.2916
ZINC06162101	-41.5869	-45.2895
ZINC08397086	-41.5777	-45.2863
ZINC19938384	-47.2985	-45.2843
ZINC09043299	-40.6430	-45.2802
ZINC04065958	-41.3549	-45.2783
ZINC04065958	-40.9421	-45.2783
ZINC08383503	-45.7028	-45.2773
ZINC08383503	-42.2982	-45.2773
ZINC06162290	-39.9212	-45.2772
ZINC06162290	-38.5456	-45.2772
ZINC19832279	-39.1053	-45.2744
ZINC06149892	-41.9082	-45.2712
ZINC06149892	-39.2374	-45.2712
ZINC08430293	-42.5465	-45.2700
ZINC08430293	-39.9770	-45.2700
ZINC08430293	-39.8659	-45.2700
ZINC04067211	-42.5807	-45.2698
ZINC04067211	-41.9193	-45.2698
ZINC08424647	-46.2069	-45.2694
ZINC09011969	-43.4630	-45.2670
ZINC09072872	-45.9445	-45.2664
ZINC09072872	-45.6078	-45.2664
ZINC08440818	-42.6055	-45.2658
ZINC08440818	-41.9594	-45.2658
ZINC19790650	-40.4725	-45.2647
ZINC08410211	-39.2932	-45.2635
ZINC08415417	-39.6898	-45.2623
ZINC08415417	-39.0953	-45.2623
ZINC08438704	-42.0095	-45.2621
ZINC08455851	-43.6662	-45.2616

ZINC08439528	-46.3690	-45.2595
ZINC08439528	-46.1753	-45.2595
ZINC08439528	-45.3531	-45.2595
ZINC09186801	-47.7642	-45.2593
ZINC09186801	-45.4661	-45.2593
ZINC09186801	-43.0008	-45.2593
ZINC08437370	-40.9452	-45.2591
ZINC19852637	-45.5157	-45.2582
ZINC04095420	-42.0969	-45.2542
ZINC08429891	-43.5493	-45.2541
ZINC04417695	-41.5319	-45.2541
ZINC04417695	-39.5101	-45.2541
ZINC08429891	-38.0748	-45.2541
ZINC08416402	-41.7892	-45.2489
ZINC08416402	-41.6459	-45.2489
ZINC08416402	-39.6717	-45.2489
ZINC08441554	-39.7032	-45.2480
ZINC08441554	-39.6662	-45.2480
ZINC08441554	-38.5831	-45.2480
ZINC08433291	-44.8274	-45.2474
ZINC00674239	-40.9485	-45.2473
ZINC02204614	-48.9801	-45.2461
ZINC08441996	-42.3660	-45.2419
ZINC19314631	-41.2712	-45.2414
ZINC13206173	-44.5078	-45.2394
ZINC04066894	-39.5103	-45.2353
ZINC04064586	-46.9975	-45.2284
ZINC04064586	-45.1124	-45.2284
ZINC08383688	-45.4808	-45.2260
ZINC04063851	-38.9245	-45.2257
ZINC04063851	-37.1729	-45.2257
ZINC04063851	-37.1128	-45.2257
ZINC08398990	-40.7832	-45.2248
ZINC04418113	-41.3170	-45.2222
ZINC04418113	-41.0564	-45.2222
ZINC04066033	-42.2984	-45.2186
ZINC04066033	-42.0666	-45.2186
ZINC04063820	-38.8945	-45.2086
ZINC04065813	-41.0162	-45.2040
ZINC04065813	-40.9883	-45.2040
ZINC04065813	-39.8352	-45.2040
ZINC00673823	-42.9181	-45.2006
ZINC00726621	-39.0502	-45.1986
ZINC00726621	-36.5577	-45.1986
ZINC06162083	-42.3730	-45.1970
ZINC08425381	-41.2010	-45.1961
ZINC08425381	-37.2404	-45.1961
ZINC08441305	-39.9947	-45.1955
ZINC08441305	-36.4146	-45.1955
ZINC08430037	-43.0562	-45.1908
ZINC08430037	-37.1162	-45.1908
ZINC06149906	-43.4216	-45.1882
ZINC06144548	-48.5809	-45.1882
ZINC08440598	-43.9895	-45.1844

ZINC08440598	-41.5998	-45.1844
ZINC06162260	-42.1567	-45.1832
ZINC06162260	-40.1148	-45.1832
ZINC09350325	-40.2331	-45.1800
ZINC09350325	-39.7780	-45.1800
ZINC09071221	-43.4341	-45.1772
ZINC09071221	-43.0993	-45.1772
ZINC09071221	-42.7909	-45.1772
ZINC09071221	-42.7507	-45.1772
ZINC09071221	-42.6731	-45.1772
ZINC06148676	-45.5630	-45.1752
ZINC00674031	-44.2850	-45.1733
ZINC00674031	-42.7777	-45.1733
ZINC00647046	-43.0662	-45.1727
ZINC05894945	-40.9793	-45.1725
ZINC00704192	-44.5468	-45.1724
ZINC04067801	-41.2685	-45.1715
ZINC17154930	-44.3177	-45.1713
ZINC06136868	-40.7508	-45.1704
ZINC06136868	-39.9052	-45.1704
ZINC06136868	-39.1686	-45.1704
ZINC00815215	-43.1986	-45.1669
ZINC00666759	-43.8857	-45.1643
ZINC20357730	-40.8739	-45.1633
ZINC20357730	-37.3811	-45.1633
ZINC22862432	-45.0228	-45.1627
ZINC08437157	-37.1181	-45.1616
ZINC00706815	-41.8243	-45.1602
ZINC06195583	-42.0705	-45.1559
ZINC00702997	-45.9898	-45.1537
ZINC09437157	-47.1084	-45.1502
ZINC04059728	-41.2461	-45.1485
ZINC04059728	-38.2010	-45.1485
ZINC04059642	-40.6360	-45.1483
ZINC04066399	-41.7013	-45.1476
ZINC04066399	-40.7503	-45.1476
ZINC00703120	-44.8726	-45.1408
ZINC00708818	-38.6248	-45.1358
ZINC09236448	-45.3896	-45.1341
ZINC09236448	-43.4616	-45.1341
ZINC09236448	-42.9290	-45.1341
ZINC09236448	-39.1085	-45.1341
ZINC09236448	-38.9415	-45.1341
ZINC04412475	-42.4857	-45.1307
ZINC08430439	-41.6418	-45.1293
ZINC08430439	-38.3019	-45.1293
ZINC00726529	-39.6392	-45.1291
ZINC00726529	-39.3114	-45.1291
ZINC00726529	-36.5062	-45.1291
ZINC08397265	-42.1670	-45.1289
ZINC08397265	-41.6721	-45.1289
ZINC08397265	-41.1322	-45.1289
ZINC13124118	-49.9677	-45.1284
ZINC08429968	-43.6705	-45.1275

ZINC08429968	-43.3725	-45.1275
ZINC00708816	-39.2626	-45.1271
ZINC03901588	-39.9056	-45.1230
ZINC08384361	-44.4287	-45.1221
ZINC17154946	-40.4666	-45.1190
ZINC17154946	-38.6981	-45.1190
ZINC08439383	-40.9622	-45.1189
ZINC08439383	-39.6185	-45.1189
ZINC12138285	-42.3233	-45.1179
ZINC04417626	-39.7619	-45.1174
ZINC06137346	-39.9690	-45.1162
ZINC08415546	-41.6324	-45.1136
ZINC08415546	-40.2593	-45.1136
ZINC04068000	-39.0998	-45.1128
ZINC00726474	-36.7543	-45.1123
ZINC00726547	-40.6052	-45.1100
ZINC00726547	-39.6636	-45.1100
ZINC00726547	-39.0943	-45.1100
ZINC08915025	-41.8886	-45.1067
ZINC08915025	-41.8204	-45.1067
ZINC07037094	-40.1977	-45.1055
ZINC04065914	-42.1787	-45.1047
ZINC04065914	-38.6387	-45.1047
ZINC50861464	-48.4197	-45.1030
ZINC08416333	-40.0987	-45.1006
ZINC13739318	-42.9664	-45.0994
ZINC08441859	-40.6781	-45.0985
ZINC08440754	-41.6129	-45.0980
ZINC08440754	-40.8182	-45.0980
ZINC08440754	-40.4309	-45.0980
ZINC06143940	-44.8627	-45.0963
ZINC09362779	-41.4342	-45.0956
ZINC08441211	-39.2932	-45.0955
ZINC04065817	-40.8382	-45.0937
ZINC19809303	-40.7659	-45.0897
ZINC19809303	-40.3805	-45.0897
ZINC02060838	-43.7611	-45.0869
ZINC05887026	-44.2309	-45.0861
ZINC00678552	-45.6660	-45.0860
ZINC06149695	-47.2867	-45.0858
ZINC08441371	-40.5608	-45.0857
ZINC08441371	-39.0610	-45.0857
ZINC00674027	-43.5841	-45.0851
ZINC00674027	-38.6509	-45.0851
ZINC09236116	-44.2061	-45.0850
ZINC09236116	-43.1470	-45.0850
ZINC09236116	-42.5790	-45.0850
ZINC05360676	-50.9486	-45.0820
ZINC17193930	-37.3804	-45.0811
ZINC08739149	-41.4567	-45.0809
ZINC02095921	-43.5100	-45.0808
ZINC08396764	-47.7646	-45.0797
ZINC08440757	-39.0618	-45.0761
ZINC08440757	-38.8930	-45.0761

ZINC08442173	-41.2039	-45.0755
ZINC01414786	-44.2378	-45.0755
ZINC01414786	-43.3479	-45.0755
ZINC08437273	-44.0229	-45.0755
ZINC08437273	-37.7185	-45.0755
ZINC06442997	-43.8950	-45.0704
ZINC17154924	-41.2075	-45.0698
ZINC04065959	-42.6554	-45.0686
ZINC04067780	-40.5204	-45.0657
ZINC08903502	-41.1731	-45.0651
ZINC13370895	-45.5167	-45.0645
ZINC06136911	-39.5910	-45.0601
ZINC06195962	-41.7052	-45.0600
ZINC04067977	-40.3202	-45.0590
ZINC09353165	-43.8100	-45.0561
ZINC09477314	-44.0142	-45.0557
ZINC09477314	-42.7447	-45.0557
ZINC08425969	-39.9110	-45.0556
ZINC08425969	-37.5350	-45.0556
ZINC04066450	-42.7350	-45.0555
ZINC04066450	-42.6749	-45.0555
ZINC00641709	-45.0549	-45.0473
ZINC04067063	-41.4511	-45.0459
ZINC04067063	-40.7811	-45.0459
ZINC04067063	-40.2122	-45.0459
ZINC08440724	-40.9036	-45.0444
ZINC08440724	-40.7723	-45.0444
ZINC08440724	-38.6476	-45.0444
ZINC18163229	-41.3527	-45.0433
ZINC06195947	-41.3148	-45.0430
ZINC18209799	-40.6223	-45.0386
ZINC18209799	-37.7115	-45.0386
ZINC15952852	-47.5691	-45.0336
ZINC08441061	-40.5094	-45.0319
ZINC06137149	-38.2123	-45.0285
ZINC06137149	-37.0700	-45.0285
ZINC08434860	-43.6253	-45.0264
ZINC23114613	-44.1988	-45.0254
ZINC06162004	-41.2745	-45.0243
ZINC00728572	-42.4855	-45.0211
ZINC00728572	-42.0827	-45.0211
ZINC06015502	-44.2917	-45.0176
ZINC06015502	-41.1063	-45.0176
ZINC06015502	-39.9108	-45.0176
ZINC06196087	-42.2614	-45.0159
ZINC09244248	-44.6442	-45.0147
ZINC09244248	-44.3709	-45.0147
ZINC04418289	-40.6346	-45.0126
ZINC09243939	-42.7472	-45.0113
ZINC09243939	-38.9590	-45.0113
ZINC08416185	-42.3696	-45.0082
ZINC08416185	-40.0531	-45.0082
ZINC06701469	-40.4484	-45.0074
ZINC08439439	-42.7072	-45.0068

ZINC08439439	-40.8060	-45.0068
ZINC08439439	-40.2022	-45.0068
ZINC00724036	-42.8576	-45.0067
ZINC16114947	-40.1416	-45.0052
ZINC08411390	-42.3889	-44.9980
ZINC08411390	-42.3531	-44.9980
ZINC08411390	-41.0948	-44.9980
ZINC05295145	-45.9716	-44.9974
ZINC28428187	-38.4536	-44.9971
ZINC19781983	-36.7491	-44.9946
ZINC19880637	-36.5287	-44.9941
ZINC19802350	-41.6518	-44.9936
ZINC19802350	-41.2725	-44.9936
ZINC31810955	-46.8797	-44.9931
ZINC31810955	-45.8507	-44.9931
ZINC31810955	-45.6170	-44.9931
ZINC31810955	-44.0337	-44.9931
ZINC08439860	-42.4437	-44.9918
ZINC08439860	-37.1469	-44.9918
ZINC08440554	-39.9051	-44.9883
ZINC15931612	-41.2004	-44.9852
ZINC04066420	-40.2488	-44.9851
ZINC05918870	-45.9066	-44.9850
ZINC05918870	-43.6717	-44.9850
ZINC08439857	-40.3644	-44.9847
ZINC08439857	-40.0147	-44.9847
ZINC19781995	-38.1576	-44.9845
ZINC19781995	-38.0570	-44.9845
ZINC08384743	-47.3366	-44.9820
ZINC06148664	-43.1019	-44.9812
ZINC06148664	-39.8507	-44.9812
ZINC06148664	-37.9044	-44.9812
ZINC08442179	-39.3954	-44.9789
ZINC04067636	-41.1487	-44.9764
ZINC04649902	-38.6103	-44.9742
ZINC08442509	-43.0999	-44.9733
ZINC08442509	-41.7022	-44.9733
ZINC08442509	-40.4586	-44.9733
ZINC00647634	-41.4580	-44.9695
ZINC06195429	-41.3303	-44.9691
ZINC06195429	-40.9528	-44.9691
ZINC06161971	-41.4418	-44.9675
ZINC01221290	-41.5237	-44.9673
ZINC08442181	-40.0321	-44.9668
ZINC04068033	-44.6278	-44.9643
ZINC04068033	-43.2484	-44.9643
ZINC04631265	-42.6518	-44.9634
ZINC18145459	-43.3696	-44.9627
ZINC18145459	-41.7309	-44.9627
ZINC00717668	-40.9309	-44.9624
ZINC00717668	-40.2355	-44.9624
ZINC00726615	-40.9216	-44.9621
ZINC00726615	-40.8556	-44.9621
ZINC00726615	-40.1950	-44.9621

ZINC04067777	-40.5414	-44.9596
ZINC00716705	-45.3937	-44.9593
ZINC00716705	-42.2218	-44.9593
ZINC13388414	-40.3507	-44.9578
ZINC13388414	-39.4686	-44.9578
ZINC19369718	-42.2922	-44.9562
ZINC19369718	-39.5642	-44.9562
ZINC04065202	-42.5473	-44.9555
ZINC08440504	-43.9117	-44.9530
ZINC06148668	-42.9405	-44.9504
ZINC06148668	-39.2990	-44.9504
ZINC06148668	-36.7466	-44.9504
ZINC12771305	-41.8437	-44.9492
ZINC09044104	-42.8055	-44.9482
ZINC19802460	-42.4013	-44.9478
ZINC19802460	-38.8138	-44.9478
ZINC09008754	-45.4882	-44.9463
ZINC08441020	-40.4484	-44.9449
ZINC08441053	-41.6962	-44.9439
ZINC04383956	-43.1334	-44.9430
ZINC08695246	-46.4126	-44.9424
ZINC02069342	-42.0837	-44.9423
ZINC06015294	-43.5383	-44.9416
ZINC06015294	-42.6092	-44.9416
ZINC06015294	-42.3437	-44.9416
ZINC00135293	-38.3798	-44.9390
ZINC04060767	-40.5646	-44.9368
ZINC04060767	-38.5091	-44.9368
ZINC00702574	-41.9642	-44.9364
ZINC06785991	-41.3064	-44.9354
ZINC05918806	-44.9086	-44.9349
ZINC05918806	-43.1489	-44.9349
ZINC08450707	-38.7973	-44.9345
ZINC09339585	-39.4328	-44.9336
ZINC08451956	-43.3079	-44.9335
ZINC06444263	-42.0673	-44.9312
ZINC08438523	-44.4203	-44.9301
ZINC59817697	-38.9043	-44.9261
ZINC06197273	-39.3553	-44.9259
ZINC19596664	-44.8568	-44.9257
ZINC19596664	-42.3270	-44.9257
ZINC06162089	-42.8489	-44.9256
ZINC09271190	-41.2584	-44.9247
ZINC06162102	-41.1235	-44.9244
ZINC06204736	-39.4331	-44.9244
ZINC08439594	-42.1464	-44.9225
ZINC08439594	-41.4372	-44.9225
ZINC04067915	-39.0516	-44.9224
ZINC17196864	-47.7418	-44.9223
ZINC06197288	-42.8346	-44.9212
ZINC06197288	-41.6854	-44.9212
ZINC08397755	-42.3188	-44.9207
ZINC09065882	-49.3375	-44.9205
ZINC00681651	-40.6753	-44.9203

ZINC00717020	-41.1567	-44.9174
ZINC00717020	-37.5643	-44.9174
ZINC00628991	-42.6876	-44.9165
ZINC00628991	-42.2104	-44.9165
ZINC08413440	-41.6240	-44.9140
ZINC08413440	-39.9998	-44.9140
ZINC04285041	-47.9409	-44.9126
ZINC09046842	-40.0739	-44.9098
ZINC09046842	-37.8800	-44.9098
ZINC36026169	-40.6912	-44.9066
ZINC36026169	-40.4949	-44.9066
ZINC04067279	-44.2879	-44.9055
ZINC04067279	-41.5090	-44.9055
ZINC04067279	-41.4946	-44.9055
ZINC19871698	-42.0454	-44.9003
ZINC25336422	-41.0983	-44.8993
ZINC25336422	-38.5596	-44.8993
ZINC06162230	-40.9175	-44.8985
ZINC06136869	-41.1064	-44.8975
ZINC08384219	-42.9440	-44.8975
ZINC06702955	-41.1162	-44.8958
ZINC06197415	-38.7128	-44.8957
ZINC08441574	-40.7575	-44.8941
ZINC08441574	-39.8670	-44.8941
ZINC06137366	-42.0680	-44.8925
ZINC06137366	-41.6549	-44.8925
ZINC06137366	-40.9956	-44.8925
ZINC19922784	-47.1051	-44.8921
ZINC23358726	-41.8867	-44.8921
ZINC04670896	-41.3335	-44.8899
ZINC17136160	-44.1133	-44.8894
ZINC19872244	-41.0513	-44.8887
ZINC08440925	-45.8401	-44.8872
ZINC09122097	-44.7329	-44.8849
ZINC09122097	-43.2635	-44.8849
ZINC08441657	-44.1254	-44.8843
ZINC02088443	-43.3278	-44.8826
ZINC00674827	-42.6181	-44.8821
ZINC00674827	-37.7715	-44.8821
ZINC08384241	-43.2971	-44.8820
ZINC13571381	-49.6592	-44.8808
ZINC13571381	-49.1813	-44.8808
ZINC08413059	-41.5987	-44.8801
ZINC04067944	-39.1800	-44.8789
ZINC08739862	-41.8701	-44.8782
ZINC19790731	-41.8590	-44.8770
ZINC08441212	-44.4918	-44.8768
ZINC03768128	-41.6763	-44.8735
ZINC04059946	-39.2567	-44.8715
ZINC04017106	-37.1553	-44.8707
ZINC04017106	-37.0425	-44.8707
ZINC08396858	-44.1973	-44.8678
ZINC22922793	-41.7795	-44.8663
ZINC13382061	-37.5235	-44.8659

ZINC19360132	-36.9499	-44.8657
ZINC08743797	-46.3060	-44.8654
ZINC08743797	-44.5298	-44.8654
ZINC02455393	-42.2021	-44.8651
ZINC22107518	-38.7029	-44.8651
ZINC04065031	-38.7468	-44.8634
ZINC19801734	-36.3112	-44.8598
ZINC08430630	-42.0277	-44.8546
ZINC08430630	-38.2126	-44.8546
ZINC19781701	-36.3692	-44.8544
ZINC13161554	-43.2727	-44.8514
ZINC18324666	-42.2659	-44.8513
ZINC04065903	-41.4596	-44.8509
ZINC08433247	-43.2300	-44.8496
ZINC08441827	-41.5715	-44.8488
ZINC06197522	-37.8502	-44.8455
ZINC17122624	-44.1526	-44.8448
ZINC17122624	-44.1030	-44.8448
ZINC08397276	-37.9853	-44.8446
ZINC08397276	-36.4318	-44.8446
ZINC00726616	-38.0453	-44.8421
ZINC04649849	-41.0869	-44.8420
ZINC04066342	-42.1304	-44.8416
ZINC19827979	-41.9132	-44.8415
ZINC00844246	-40.3683	-44.8402
ZINC00844246	-38.6002	-44.8402
ZINC00844246	-38.2558	-44.8402
ZINC06194177	-39.9485	-44.8394
ZINC06691758	-42.4527	-44.8350
ZINC06691758	-36.3833	-44.8350
ZINC08439441	-44.9142	-44.8327
ZINC08439441	-44.1565	-44.8327
ZINC06162499	-41.5489	-44.8323
ZINC08441366	-39.8609	-44.8306
ZINC08398414	-42.3581	-44.8302
ZINC19901143	-45.4425	-44.8301
ZINC33908953	-46.0918	-44.8301
ZINC08439751	-44.9372	-44.8296
ZINC08439751	-43.6656	-44.8296
ZINC08439751	-42.9086	-44.8296
ZINC08439751	-42.9086	-44.8296
ZINC08440938	-40.4792	-44.8263
ZINC08440938	-37.5323	-44.8263
ZINC00850905	-41.2580	-44.8252
ZINC04065969	-41.3288	-44.8238
ZINC04065969	-39.5147	-44.8238
ZINC08438563	-40.4967	-44.8237
ZINC08438563	-37.9827	-44.8237
ZINC04568447	-43.5348	-44.8237
ZINC04568447	-43.4352	-44.8237
ZINC06159425	-39.7768	-44.8194
ZINC00709901	-40.3470	-44.8165
ZINC08416175	-40.1877	-44.8138
ZINC04065139	-37.9876	-44.8133

ZINC04065139	-36.4263	-44.8133
ZINC19909174	-42.8723	-44.8131
ZINC08430288	-38.3375	-44.8130
ZINC08442214	-41.9674	-44.8120
ZINC04066827	-38.2453	-44.8092
ZINC04066827	-37.5320	-44.8092
ZINC04066827	-36.8348	-44.8092
ZINC08441352	-41.3072	-44.8082
ZINC08441352	-39.9808	-44.8082
ZINC08441352	-38.1502	-44.8082
ZINC09243266	-40.9010	-44.8061
ZINC09243266	-40.8342	-44.8061
ZINC04065960	-41.7971	-44.8057
ZINC04065960	-40.7696	-44.8057
ZINC08441350	-45.0447	-44.8056
ZINC08430715	-40.8567	-44.8055
ZINC08430715	-39.9457	-44.8055
ZINC08430715	-39.7429	-44.8055
ZINC06162020	-40.9000	-44.8011
ZINC08440521	-39.4348	-44.7995
ZINC08440521	-37.7499	-44.7995
ZINC04062955	-38.4802	-44.7974
ZINC04062955	-37.2899	-44.7974
ZINC08440500	-43.1707	-44.7971
ZINC08440500	-37.3351	-44.7971
ZINC08712165	-43.6719	-44.7969
ZINC06195966	-40.8171	-44.7952
ZINC09380341	-39.5871	-44.7931
ZINC17145497	-44.8108	-44.7926
ZINC17145497	-43.1578	-44.7926
ZINC17145497	-42.5240	-44.7926
ZINC17145497	-42.4552	-44.7926
ZINC17145497	-42.1000	-44.7926
ZINC08415744	-39.3367	-44.7924
ZINC08415744	-38.9377	-44.7924
ZINC13067946	-43.3631	-44.7920
ZINC13067946	-36.4461	-44.7920
ZINC04066466	-38.9178	-44.7895
ZINC08439577	-43.2183	-44.7867
ZINC08439577	-40.9070	-44.7867
ZINC06137423	-43.8101	-44.7858
ZINC02055872	-45.8671	-44.7849
ZINC06137419	-40.4120	-44.7833
ZINC13549195	-42.0966	-44.7819
ZINC13176775	-38.3598	-44.7812
ZINC08439599	-40.8070	-44.7790
ZINC04285242	-39.0802	-44.7781
ZINC00702369	-46.2434	-44.7756
ZINC00702369	-40.7757	-44.7756
ZINC08439782	-41.2179	-44.7752
ZINC08439782	-39.9636	-44.7752
ZINC08425388	-40.0518	-44.7737
ZINC08425388	-38.8685	-44.7737
ZINC08425388	-37.1597	-44.7737

ZINC08444358	-40.6157	-44.7731
ZINC06137403	-42.9029	-44.7717
ZINC06137403	-38.6377	-44.7717
ZINC19851389	-43.2410	-44.7708
ZINC00702729	-40.7230	-44.7702
ZINC00702729	-39.3386	-44.7702
ZINC06148529	-41.3942	-44.7701
ZINC04067040	-40.7553	-44.7681
ZINC04067040	-40.4882	-44.7681
ZINC08439459	-41.0057	-44.7672
ZINC08439459	-40.8439	-44.7672
ZINC08439459	-40.2808	-44.7672
ZINC19815851	-42.8667	-44.7666
ZINC19815851	-40.1831	-44.7666
ZINC09411238	-42.7180	-44.7664
ZINC06195386	-39.0472	-44.7653
ZINC09424793	-41.9908	-44.7643
ZINC85425814	-38.8554	-44.7635
ZINC85425814	-38.3907	-44.7635
ZINC08415666	-41.5115	-44.7634
ZINC08415666	-40.8636	-44.7634
ZINC09328857	-43.2300	-44.7608
ZINC06197169	-42.6867	-44.7607
ZINC15773885	-44.6840	-44.7592
ZINC08438758	-45.3803	-44.7586
ZINC08438758	-43.7343	-44.7586
ZINC19832122	-38.5742	-44.7584
ZINC19832122	-38.4240	-44.7584
ZINC19897298	-38.0382	-44.7583
ZINC08384184	-43.1614	-44.7579
ZINC08384184	-42.4798	-44.7579
ZINC09271669	-44.7898	-44.7577
ZINC08441769	-45.5880	-44.7576
ZINC19840893	-43.1958	-44.7576
ZINC04112280	-42.5158	-44.7561
ZINC04112280	-39.1116	-44.7561
ZINC04112280	-37.1519	-44.7561
ZINC04067428	-41.7574	-44.7541
ZINC04067428	-40.9927	-44.7541
ZINC04067428	-39.6183	-44.7541
ZINC06197270	-39.8212	-44.7516
ZINC09015419	-40.7060	-44.7514
ZINC08396796	-42.0024	-44.7498
ZINC19889539	-41.2179	-44.7491
ZINC19889539	-40.0917	-44.7491
ZINC19889539	-37.3984	-44.7491
ZINC19889539	-37.3668	-44.7491
ZINC17150191	-47.6818	-44.7483
ZINC05595980	-40.5094	-44.7468
ZINC05595980	-39.6742	-44.7468
ZINC05894960	-41.3976	-44.7426
ZINC08438732	-43.4206	-44.7406
ZINC08438739	-42.0789	-44.7392
ZINC00708008	-41.1988	-44.7389

ZINC00708008	-41.0827	-44.7389
ZINC00708008	-40.0452	-44.7389
ZINC19316049	-44.2252	-44.7378
ZINC08416394	-43.5769	-44.7364
ZINC08416394	-43.3313	-44.7364
ZINC08416394	-36.9629	-44.7364
ZINC06196435	-39.1804	-44.7348
ZINC12468075	-43.6916	-44.7339
ZINC04066097	-38.5462	-44.7323
ZINC00671297	-47.8611	-44.7296
ZINC01413516	-47.2065	-44.7278
ZINC01413516	-46.7175	-44.7278
ZINC08440722	-41.3919	-44.7244
ZINC00823437	-41.5563	-44.7241
ZINC00823437	-39.6115	-44.7241
ZINC00823437	-36.8531	-44.7241
ZINC04062872	-42.5104	-44.7231
ZINC04062872	-41.8941	-44.7231
ZINC04062872	-41.7164	-44.7231
ZINC08439779	-40.4896	-44.7230
ZINC08439779	-39.4599	-44.7230
ZINC09360736	-41.8865	-44.7230
ZINC04119356	-41.5569	-44.7227
ZINC00669685	-45.9938	-44.7194
ZINC04067751	-40.4171	-44.7177
ZINC01821349	-42.2362	-44.7163
ZINC08444691	-40.7942	-44.7143
ZINC08429991	-42.4092	-44.7113
ZINC08429991	-40.8921	-44.7113
ZINC08429991	-40.8369	-44.7113
ZINC06194274	-44.0853	-44.7091
ZINC08413226	-42.2360	-44.7087
ZINC08413226	-41.8924	-44.7087
ZINC08438747	-42.5250	-44.7077
ZINC00711130	-41.6697	-44.7076
ZINC08441625	-41.9682	-44.7066
ZINC08441625	-38.5594	-44.7066
ZINC00857661	-40.0606	-44.7062
ZINC08441501	-45.3188	-44.7056
ZINC16115797	-39.8284	-44.7018
ZINC16115797	-39.7661	-44.7018
ZINC09272672	-44.0201	-44.6978
ZINC00180991	-41.5558	-44.6965
ZINC06162019	-40.2044	-44.6963
ZINC00822771	-38.3032	-44.6956
ZINC08413126	-44.0755	-44.6942
ZINC08413126	-40.9120	-44.6942
ZINC08439515	-42.2114	-44.6915
ZINC08439515	-39.9526	-44.6915
ZINC08387672	-40.1775	-44.6908
ZINC08387672	-39.0006	-44.6908
ZINC08387672	-37.5781	-44.6908
ZINC39067137	-40.1329	-44.6868
ZINC08740585	-45.3771	-44.6858

ZINC08740585	-45.2201	-44.6858
ZINC08415996	-39.7672	-44.6807
ZINC08415996	-39.3658	-44.6807
ZINC08415996	-37.0503	-44.6807
ZINC04067074	-41.3787	-44.6773
ZINC04067074	-39.4847	-44.6773
ZINC04629631	-40.5584	-44.6771
ZINC04629631	-40.3504	-44.6771
ZINC19871450	-42.5326	-44.6759
ZINC08441069	-39.9760	-44.6750
ZINC09358274	-40.0334	-44.6738
ZINC09358274	-39.7364	-44.6738
ZINC00726685	-39.9714	-44.6734
ZINC04417946	-38.5677	-44.6712
ZINC04417946	-36.7879	-44.6712
ZINC04284846	-41.8523	-44.6639
ZINC04284846	-40.5400	-44.6639
ZINC08444348	-42.5507	-44.6635
ZINC08444348	-40.6259	-44.6635
ZINC06149893	-39.8067	-44.6613
ZINC08432499	-39.9177	-44.6612
ZINC00703116	-43.5322	-44.6593
ZINC16115801	-39.9658	-44.6583
ZINC08437322	-42.9377	-44.6564
ZINC01413494	-42.9785	-44.6509
ZINC09350765	-40.2580	-44.6460
ZINC00703675	-39.6909	-44.6458
ZINC08416229	-41.9412	-44.6444
ZINC08416229	-41.7118	-44.6444
ZINC08416229	-41.1688	-44.6444
ZINC08439419	-45.2380	-44.6436
ZINC08445127	-43.3397	-44.6435
ZINC08445127	-42.5218	-44.6435
ZINC08435039	-45.4912	-44.6411
ZINC04905474	-41.9298	-44.6407
ZINC08415600	-40.4358	-44.6401
ZINC09282459	-42.7551	-44.6367
ZINC09282459	-42.4387	-44.6367
ZINC09244134	-42.3666	-44.6345
ZINC09244134	-41.3460	-44.6345
ZINC09270915	-40.1875	-44.6344
ZINC09270915	-39.2622	-44.6344
ZINC18244237	-41.7792	-44.6312
ZINC08440217	-40.5127	-44.6311
ZINC08440217	-40.3035	-44.6311
ZINC09243149	-43.2583	-44.6309
ZINC09243149	-42.7324	-44.6309
ZINC00702433	-41.5994	-44.6240
ZINC00702433	-39.5698	-44.6240
ZINC09009196	-44.5855	-44.6220
ZINC09009196	-44.3747	-44.6220
ZINC08416385	-39.1975	-44.6219
ZINC06148778	-41.4229	-44.6184
ZINC06148778	-38.6980	-44.6184

ZINC06148778	-37.9098	-44.6184
ZINC08435048	-38.9853	-44.6168
ZINC02075469	-40.5295	-44.6163
ZINC08744092	-40.6126	-44.6115
ZINC09240619	-45.1078	-44.6103
ZINC09240619	-43.9560	-44.6103
ZINC09240619	-43.7653	-44.6103
ZINC04066190	-37.7813	-44.6099
ZINC02789028	-40.4196	-44.6090
ZINC06197214	-41.9181	-44.6087
ZINC06197214	-40.9035	-44.6087
ZINC01413517	-48.0246	-44.6087
ZINC01413517	-45.9489	-44.6087
ZINC08415635	-42.0574	-44.6084
ZINC08415635	-41.9640	-44.6084
ZINC08415635	-40.6531	-44.6084
ZINC08433253	-41.7010	-44.6033
ZINC08433253	-41.6103	-44.6033
ZINC19872282	-41.3794	-44.6022
ZINC00826316	-40.6220	-44.6018
ZINC00826316	-39.7729	-44.6018
ZINC08439618	-42.4568	-44.6017
ZINC08439618	-41.5325	-44.6017
ZINC00865687	-43.0173	-44.6012
ZINC08387278	-46.3975	-44.6002
ZINC06015359	-40.3792	-44.5991
ZINC00726614	-40.9672	-44.5989
ZINC00726614	-39.0052	-44.5989
ZINC04067943	-39.1035	-44.5982
ZINC08444407	-45.1599	-44.5945
ZINC19872208	-40.5756	-44.5932
ZINC08442167	-42.3604	-44.5929
ZINC01413523	-47.9924	-44.5910
ZINC00944850	-41.5512	-44.5907
ZINC06162003	-42.0700	-44.5895
ZINC85389533	-40.8695	-44.5876
ZINC06195694	-41.5843	-44.5856
ZINC04067397	-41.9220	-44.5836
ZINC04067397	-41.5655	-44.5836
ZINC04067397	-40.8553	-44.5836
ZINC00726543	-38.9804	-44.5833
ZINC00726543	-37.9909	-44.5833
ZINC08398049	-42.4638	-44.5825
ZINC05220348	-41.2652	-44.5799
ZINC05220348	-40.5376	-44.5799
ZINC00865689	-42.8920	-44.5765
ZINC08440808	-43.5001	-44.5762
ZINC08440808	-42.2241	-44.5762
ZINC19909177	-43.2674	-44.5740
ZINC09243236	-42.2080	-44.5715
ZINC04063108	-36.5231	-44.5705
ZINC08440485	-44.0042	-44.5702
ZINC04066825	-39.3099	-44.5701
ZINC38191026	-43.9295	-44.5668

ZINC06697363	-38.7742	-44.5639
ZINC06196039	-42.6771	-44.5589
ZINC08386153	-46.3206	-44.5584
ZINC04064854	-42.4275	-44.5579
ZINC04064854	-40.0667	-44.5579
ZINC00702434	-42.4420	-44.5578
ZINC00702434	-42.3467	-44.5578
ZINC08424641	-45.6564	-44.5565
ZINC09178284	-43.5906	-44.5560
ZINC04418014	-40.9342	-44.5515
ZINC04418014	-40.6819	-44.5515
ZINC08384369	-43.4429	-44.5505
ZINC05806943	-40.1343	-44.5445
ZINC04666921	-37.5659	-44.5428
ZINC08450313	-42.8482	-44.5422
ZINC08450313	-41.6796	-44.5422
ZINC22972183	-40.5218	-44.5404
ZINC04065482	-43.5669	-44.5404
ZINC04065482	-42.5557	-44.5404
ZINC04065482	-41.7089	-44.5404
ZINC19802260	-37.6767	-44.5365
ZINC19802260	-36.4924	-44.5365
ZINC09060336	-46.3993	-44.5341
ZINC09437321	-43.1369	-44.5341
ZINC68734835	-39.4675	-44.5327
ZINC68734835	-38.8934	-44.5327
ZINC68734835	-38.6123	-44.5327
ZINC12970180	-40.0638	-44.5312
ZINC08972644	-43.8613	-44.5306
ZINC08415620	-42.7327	-44.5304
ZINC08415620	-41.1251	-44.5304
ZINC08415620	-40.6041	-44.5304
ZINC08415653	-40.3367	-44.5220
ZINC08415653	-39.9576	-44.5220
ZINC08415653	-39.8523	-44.5220
ZINC02088442	-41.4415	-44.5217
ZINC00844232	-40.3730	-44.5201
ZINC00844232	-39.1367	-44.5201
ZINC06090161	-43.9949	-44.5199
ZINC06137202	-39.6683	-44.5167
ZINC06137202	-39.0892	-44.5167
ZINC00984823	-43.2465	-44.5160
ZINC20101048	-45.0481	-44.5158
ZINC20262879	-45.9637	-44.5148
ZINC06161990	-41.5052	-44.5112
ZINC06161990	-38.1002	-44.5112
ZINC00703000	-41.3178	-44.5105
ZINC08382821	-39.2152	-44.5102
ZINC04065697	-44.0720	-44.5073
ZINC04065697	-43.3160	-44.5073
ZINC04065697	-42.1733	-44.5073
ZINC08431434	-42.5643	-44.5068
ZINC17478824	-42.5566	-44.5055
ZINC17478824	-38.0495	-44.5055

ZINC04068048	-37.4326	-44.5005
ZINC04068048	-36.2624	-44.5005
ZINC32617194	-39.6036	-44.5001
ZINC04649502	-40.6425	-44.4990
ZINC06196241	-37.9670	-44.4964
ZINC05806868	-41.2123	-44.4953
ZINC05806868	-40.0919	-44.4953
ZINC00726511	-38.6246	-44.4945
ZINC00726511	-37.8447	-44.4945
ZINC04067672	-42.2078	-44.4934
ZINC04067672	-37.9839	-44.4934
ZINC04067672	-37.7225	-44.4934
ZINC09424269	-43.3945	-44.4927
ZINC09424269	-42.4343	-44.4927
ZINC08414964	-39.8002	-44.4920
ZINC08414964	-36.8558	-44.4920
ZINC08439571	-48.6007	-44.4911
ZINC08439571	-47.8157	-44.4911
ZINC08439571	-40.8461	-44.4911
ZINC00626234	-44.5849	-44.4890
ZINC00626234	-41.7691	-44.4890
ZINC19944962	-38.4765	-44.4877
ZINC06195652	-43.0500	-44.4821
ZINC06195652	-39.9702	-44.4821
ZINC08439861	-40.3822	-44.4815
ZINC08439861	-39.1291	-44.4815
ZINC01413499	-47.3275	-44.4802
ZINC01413499	-45.1490	-44.4802
ZINC22936094	-40.8627	-44.4799
ZINC08398591	-45.8028	-44.4736
ZINC00998840	-42.4165	-44.4718
ZINC04067402	-43.1613	-44.4701
ZINC04067402	-40.4385	-44.4701
ZINC15773883	-42.4916	-44.4691
ZINC13108381	-41.2609	-44.4676
ZINC08434864	-45.7813	-44.4652
ZINC19938364	-44.4667	-44.4646
ZINC06446604	-40.8161	-44.4633
ZINC04066001	-40.6111	-44.4574
ZINC04066001	-39.4563	-44.4574
ZINC08837659	-44.1099	-44.4537
ZINC08837659	-43.5053	-44.4537
ZINC08837659	-43.3319	-44.4537
ZINC03905785	-42.8508	-44.4533
ZINC03905785	-41.4496	-44.4533
ZINC08440741	-40.4163	-44.4482
ZINC08440741	-39.3098	-44.4482
ZINC08440741	-37.2965	-44.4482
ZINC08383889	-42.7913	-44.4470
ZINC08383889	-40.9023	-44.4470
ZINC16607697	-37.6617	-44.4430
ZINC02455406	-37.1850	-44.4426
ZINC02455406	-36.5374	-44.4426
ZINC06621779	-39.5274	-44.4394

ZINC00703067	-42.9583	-44.4361
ZINC00703067	-41.4040	-44.4361
ZINC06444965	-46.4482	-44.4316
ZINC04649899	-41.3772	-44.4292
ZINC02088185	-38.2084	-44.4276
ZINC19802253	-38.3443	-44.4239
ZINC08462319	-42.2960	-44.4237
ZINC04617704	-39.1926	-44.4235
ZINC06697362	-38.3391	-44.4219
ZINC06197315	-41.9547	-44.4217
ZINC08415740	-40.3083	-44.4214
ZINC08415740	-39.3022	-44.4214
ZINC08415740	-39.0167	-44.4214
ZINC06148733	-43.1520	-44.4193
ZINC00674909	-41.2427	-44.4178
ZINC04068051	-39.4102	-44.4170
ZINC04068051	-38.7954	-44.4170
ZINC04068051	-37.0204	-44.4170
ZINC00708832	-39.6960	-44.4147
ZINC00708832	-39.3627	-44.4147
ZINC00708832	-39.2867	-44.4147
ZINC00671399	-47.8792	-44.4130
ZINC08441080	-39.8112	-44.4119
ZINC09046270	-39.5790	-44.4095
ZINC09328856	-41.9971	-44.4087
ZINC04855405	-40.8531	-44.4083
ZINC04855405	-40.2043	-44.4083
ZINC06197156	-41.6521	-44.4080
ZINC20026408	-43.7268	-44.4072
ZINC08384145	-40.3941	-44.4066
ZINC08384145	-39.0367	-44.4066
ZINC01413489	-47.0085	-44.4061
ZINC01413489	-43.3815	-44.4061
ZINC08442000	-41.7605	-44.4060
ZINC08442000	-41.2464	-44.4060
ZINC09165131	-44.2328	-44.4054
ZINC04149911	-48.7071	-44.4044
ZINC22922783	-48.7861	-44.4032
ZINC22922783	-48.2801	-44.4032
ZINC22922783	-46.7363	-44.4032
ZINC22922783	-46.5406	-44.4032
ZINC22922783	-41.5728	-44.4032
ZINC06136880	-41.4061	-44.4026
ZINC08695218	-49.4440	-44.4025
ZINC08437445	-38.5208	-44.4021
ZINC05045111	-40.7026	-44.3998
ZINC05045111	-39.7678	-44.3998
ZINC06195459	-44.3822	-44.3917
ZINC08827585	-40.4488	-44.3904
ZINC08441590	-39.8618	-44.3902
ZINC08441590	-38.6512	-44.3902
ZINC08441335	-41.5181	-44.3872
ZINC08396203	-39.4423	-44.3864
ZINC08396203	-36.9102	-44.3864

ZINC18009208	-42.4232	-44.3859
ZINC18009208	-41.8932	-44.3859
ZINC04905456	-38.9729	-44.3858
ZINC00726596	-41.5711	-44.3838
ZINC00726596	-39.6411	-44.3838
ZINC00726596	-36.6499	-44.3838
ZINC08440361	-39.2455	-44.3815
ZINC08440361	-37.6021	-44.3815
ZINC08442391	-42.1638	-44.3810
ZINC08442391	-41.9596	-44.3810
ZINC08903230	-41.0237	-44.3808
ZINC08903230	-37.9123	-44.3808
ZINC59817729	-43.0430	-44.3803
ZINC06136879	-41.0719	-44.3796
ZINC00726771	-39.8663	-44.3761
ZINC06137337	-39.1473	-44.3743
ZINC06137337	-39.0521	-44.3743
ZINC08441790	-40.4205	-44.3742
ZINC06444800	-46.8273	-44.3738
ZINC04649774	-45.7768	-44.3716
ZINC04649774	-44.6134	-44.3716
ZINC08465288	-39.5832	-44.3686
ZINC33350092	-46.2141	-44.3684
ZINC09240205	-46.6131	-44.3653
ZINC09240205	-46.0634	-44.3653
ZINC09240205	-44.4072	-44.3653
ZINC09240205	-43.4475	-44.3653
ZINC09240205	-43.2573	-44.3653
ZINC01003745	-38.3597	-44.3651
ZINC08440307	-41.9410	-44.3626
ZINC08695210	-49.7135	-44.3625
ZINC08439676	-44.6188	-44.3612
ZINC19800181	-38.9882	-44.3600
ZINC04067626	-39.2626	-44.3589
ZINC04067626	-38.8471	-44.3589
ZINC08442192	-41.4640	-44.3558
ZINC00645066	-41.0727	-44.3557
ZINC08440599	-41.2473	-44.3538
ZINC04019580	-39.9682	-44.3536
ZINC04019580	-39.9044	-44.3536
ZINC00702475	-47.0771	-44.3533
ZINC06813277	-41.5964	-44.3532
ZINC04067896	-41.2199	-44.3531
ZINC04067896	-40.9058	-44.3531
ZINC04067896	-39.4604	-44.3531
ZINC08437423	-37.9615	-44.3500
ZINC08440831	-41.5012	-44.3500
ZINC08424587	-42.6928	-44.3490
ZINC08424587	-41.6638	-44.3490
ZINC06137188	-41.9452	-44.3469
ZINC06137188	-41.6567	-44.3469
ZINC06137188	-38.4865	-44.3469
ZINC04067708	-43.8887	-44.3465
ZINC04067708	-43.1071	-44.3465

ZINC04067708	-42.3476	-44.3465
ZINC06161965	-38.1844	-44.3462
ZINC00702752	-43.7575	-44.3461
ZINC00702752	-43.5610	-44.3461
ZINC13896931	-40.1263	-44.3448
ZINC13896931	-39.3240	-44.3448
ZINC00658281	-40.6439	-44.3417
ZINC06197274	-40.6337	-44.3409
ZINC04060197	-41.4967	-44.3401
ZINC08434867	-40.3321	-44.3391
ZINC08425576	-40.7341	-44.3332
ZINC08425576	-40.1952	-44.3332
ZINC06059930	-46.6618	-44.3309
ZINC08441467	-45.1960	-44.3304
ZINC08441467	-44.6460	-44.3304
ZINC04066096	-40.9740	-44.3287
ZINC13522615	-38.2263	-44.3287
ZINC06162282	-40.5715	-44.3284
ZINC06162282	-39.2042	-44.3284
ZINC00998743	-37.5403	-44.3269
ZINC08413329	-41.4720	-44.3266
ZINC06407301	-40.4636	-44.3254
ZINC06196060	-43.3345	-44.3249
ZINC08397393	-45.9782	-44.3229
ZINC20414330	-41.7438	-44.3198
ZINC13476703	-38.4443	-44.3168
ZINC04062723	-42.0876	-44.3143
ZINC04062723	-39.0954	-44.3143
ZINC04066354	-39.4928	-44.3138
ZINC08437714	-41.4202	-44.3133
ZINC08437714	-36.5970	-44.3133
ZINC08426285	-37.6643	-44.3125
ZINC08426285	-37.3037	-44.3125
ZINC08426285	-37.1486	-44.3125
ZINC01019940	-41.2794	-44.3123
ZINC01019940	-41.2411	-44.3123
ZINC00801129	-43.0861	-44.3118
ZINC00801129	-42.0285	-44.3118
ZINC00801129	-41.8579	-44.3118
ZINC08440673	-40.9288	-44.3115
ZINC08440673	-40.7637	-44.3115
ZINC08432407	-41.2715	-44.3106
ZINC08432407	-41.2131	-44.3106
ZINC02184950	-41.8632	-44.3105
ZINC09271438	-45.5509	-44.3092
ZINC08424353	-39.5539	-44.3089
ZINC19897416	-39.8304	-44.3083
ZINC19897416	-36.4257	-44.3083
ZINC19841207	-38.5129	-44.3082
ZINC00702992	-43.3429	-44.3054
ZINC09313126	-49.3295	-44.3054
ZINC09313126	-48.6369	-44.3054
ZINC08396439	-43.4060	-44.3036
ZINC08396439	-40.9311	-44.3036

ZINC08433239	-44.1106	-44.3007
ZINC04066562	-39.0209	-44.3006
ZINC04066562	-37.1171	-44.3006
ZINC09243610	-43.3145	-44.2975
ZINC09243610	-42.4855	-44.2975
ZINC09009887	-40.5133	-44.2972
ZINC09009887	-37.4242	-44.2972
ZINC02075829	-42.2638	-44.2970
ZINC00629916	-41.1555	-44.2957
ZINC19932939	-47.6111	-44.2951
ZINC04065945	-39.0339	-44.2926
ZINC04065945	-38.0398	-44.2926
ZINC04065945	-37.9140	-44.2926
ZINC08442062	-43.5361	-44.2908
ZINC08442062	-42.1603	-44.2908
ZINC02475759	-43.0477	-44.2840
ZINC19989827	-40.4892	-44.2827
ZINC06162257	-41.1381	-44.2823
ZINC19552407	-36.8887	-44.2742
ZINC04066658	-39.6449	-44.2731
ZINC04066658	-39.6130	-44.2731
ZINC06197572	-41.7889	-44.2711
ZINC06197572	-41.5418	-44.2711
ZINC06197416	-38.3162	-44.2698
ZINC02768417	-42.7612	-44.2687
ZINC06197181	-42.3495	-44.2673
ZINC04693657	-39.1190	-44.2653
ZINC19790686	-41.8050	-44.2623
ZINC19790686	-39.0861	-44.2623
ZINC04066353	-39.5330	-44.2575
ZINC04066353	-38.2783	-44.2575
ZINC08435283	-44.9609	-44.2562
ZINC08817982	-38.9197	-44.2555
ZINC08437261	-39.3125	-44.2550
ZINC06445949	-41.0248	-44.2534
ZINC06445949	-38.5004	-44.2534
ZINC00727075	-42.2332	-44.2524
ZINC04067403	-39.1424	-44.2509
ZINC09424861	-46.7358	-44.2506
ZINC00675263	-41.4053	-44.2489
ZINC19825677	-48.1904	-44.2483
ZINC06137467	-40.5105	-44.2469
ZINC08439579	-42.8069	-44.2464
ZINC08415370	-37.5529	-44.2454
ZINC08415370	-36.8884	-44.2454
ZINC06148677	-43.9657	-44.2449
ZINC06148677	-38.4301	-44.2449
ZINC00703142	-40.2206	-44.2445
ZINC04391432	-43.6982	-44.2437
ZINC04391432	-40.5114	-44.2437
ZINC04391432	-37.8405	-44.2437
ZINC02768185	-41.9549	-44.2425
ZINC06137516	-36.4914	-44.2397
ZINC08413253	-37.9647	-44.2388

ZINC08416193	-39.3226	-44.2342
ZINC09354088	-41.4906	-44.2300
ZINC09354088	-40.7761	-44.2300
ZINC08444364	-41.4602	-44.2290
ZINC08444364	-41.0027	-44.2290
ZINC08441285	-41.1513	-44.2272
ZINC08441285	-41.0725	-44.2272
ZINC08441285	-39.2394	-44.2272
ZINC19938305	-49.9114	-44.2267
ZINC08444634	-43.8967	-44.2253
ZINC04065957	-40.6111	-44.2250
ZINC04065957	-39.5153	-44.2250
ZINC09243863	-50.3267	-44.2179
ZINC09243863	-47.4464	-44.2179
ZINC08450291	-40.8662	-44.2134
ZINC08450291	-39.7658	-44.2134
ZINC08918947	-47.0708	-44.2101
ZINC08918947	-46.4274	-44.2101
ZINC08918947	-44.7265	-44.2101
ZINC08441007	-38.8105	-44.2087
ZINC08441007	-38.4273	-44.2087
ZINC09014833	-39.6524	-44.2079
ZINC04649847	-41.1155	-44.2075
ZINC19901322	-41.6656	-44.2065
ZINC08384775	-43.9024	-44.2046
ZINC08384775	-40.8092	-44.2046
ZINC17166874	-39.7710	-44.2045
ZINC17166874	-38.5195	-44.2045
ZINC09123184	-40.7030	-44.2035
ZINC09123184	-40.3680	-44.2035
ZINC04065989	-41.6441	-44.2020
ZINC04065989	-41.2089	-44.2020
ZINC09471274	-44.9848	-44.2013
ZINC09471274	-43.8601	-44.2013
ZINC15952859	-47.8282	-44.2000
ZINC09358836	-42.3614	-44.2000
ZINC09358836	-41.1871	-44.2000
ZINC09240371	-40.7629	-44.1993
ZINC08439221	-45.6802	-44.1990
ZINC08439221	-41.4057	-44.1990
ZINC12447504	-45.1822	-44.1966
ZINC08415677	-41.6073	-44.1963
ZINC08415677	-40.0879	-44.1963
ZINC08416404	-42.2496	-44.1944
ZINC08397156	-43.0894	-44.1934
ZINC08397156	-41.2278	-44.1934
ZINC04066572	-38.5832	-44.1915
ZINC04066572	-38.0332	-44.1915
ZINC13756966	-43.4442	-44.1914
ZINC05387584	-40.0147	-44.1902
ZINC00850075	-42.6367	-44.1893
ZINC19366905	-43.2286	-44.1891
ZINC06195489	-43.9085	-44.1875
ZINC04112065	-41.4777	-44.1855

ZINC06136964	-39.3743	-44.1851
ZINC19802458	-37.5542	-44.1830
ZINC17166746	-42.1841	-44.1826
ZINC05918668	-42.1266	-44.1809
ZINC05918668	-41.8647	-44.1809
ZINC08439450	-40.1317	-44.1796
ZINC08441161	-39.5174	-44.1771
ZINC08441161	-39.1060	-44.1771
ZINC08441161	-38.8634	-44.1771
ZINC04066098	-41.7907	-44.1756
ZINC04066098	-41.2631	-44.1756
ZINC15952576	-45.2902	-44.1741
ZINC15952576	-44.0262	-44.1741
ZINC15952576	-42.7599	-44.1741
ZINC15952576	-42.7486	-44.1741
ZINC08440119	-41.4573	-44.1739
ZINC85430897	-51.4916	-44.1736
ZINC01795475	-45.6140	-44.1735
ZINC04067740	-41.9204	-44.1730
ZINC09272925	-42.4602	-44.1719
ZINC09272925	-41.7416	-44.1719
ZINC09272925	-40.0998	-44.1719
ZINC00704278	-38.9469	-44.1718
ZINC08441074	-42.8139	-44.1688
ZINC08441074	-42.3324	-44.1688
ZINC08441074	-41.5796	-44.1688
ZINC08440052	-42.7375	-44.1675
ZINC08440052	-40.7942	-44.1675
ZINC08440052	-39.9319	-44.1675
ZINC08385209	-39.9446	-44.1655
ZINC08385209	-36.9531	-44.1655
ZINC08442205	-41.9213	-44.1654
ZINC00703148	-41.5448	-44.1648
ZINC08415674	-41.0609	-44.1646
ZINC08397816	-38.7477	-44.1639
ZINC08397816	-37.1952	-44.1639
ZINC04066138	-40.0771	-44.1636
ZINC04066138	-39.5060	-44.1636
ZINC06784144	-40.0072	-44.1627
ZINC06784144	-39.1882	-44.1627
ZINC08444408	-43.8851	-44.1580
ZINC02055377	-41.6721	-44.1574
ZINC00870893	-36.7106	-44.1560
ZINC08442809	-42.5720	-44.1553
ZINC04060636	-40.8202	-44.1546
ZINC04060636	-40.6951	-44.1546
ZINC08430773	-40.2536	-44.1531
ZINC08430773	-39.8960	-44.1531
ZINC08430773	-39.3111	-44.1531
ZINC06197317	-42.2282	-44.1520
ZINC08440968	-44.1291	-44.1519
ZINC06149863	-41.3851	-44.1503
ZINC00386314	-42.3545	-44.1495
ZINC08435388	-43.0408	-44.1481

ZINC13990435	-39.9142	-44.1444
ZINC00673922	-42.4401	-44.1443
ZINC12417837	-39.9104	-44.1432
ZINC12417837	-39.4050	-44.1432
ZINC08439424	-42.3807	-44.1423
ZINC08439424	-41.1378	-44.1423
ZINC08439424	-40.4038	-44.1423
ZINC04123603	-41.9337	-44.1417
ZINC04123603	-41.7321	-44.1417
ZINC04123603	-41.0918	-44.1417
ZINC08444591	-41.3778	-44.1390
ZINC08444591	-38.6222	-44.1390
ZINC20028405	-45.9232	-44.1381
ZINC06148737	-43.4634	-44.1363
ZINC19902543	-41.8952	-44.1354
ZINC19902543	-39.8063	-44.1354
ZINC00369689	-38.4661	-44.1335
ZINC17118134	-49.2572	-44.1330
ZINC06444297	-40.5805	-44.1316
ZINC06444297	-39.9029	-44.1316
ZINC06137296	-41.8282	-44.1313
ZINC06321511	-40.5683	-44.1311
ZINC00719700	-42.6915	-44.1305
ZINC04019585	-41.4238	-44.1300
ZINC04019585	-40.4187	-44.1300
ZINC28251964	-45.8208	-44.1292
ZINC06196097	-39.3874	-44.1285
ZINC19797005	-41.8636	-44.1280
ZINC06445943	-42.7593	-44.1250
ZINC06445943	-40.7191	-44.1250
ZINC00626705	-47.5757	-44.1193
ZINC04112109	-39.2981	-44.1187
ZINC59817693	-42.4828	-44.1185
ZINC08434862	-44.6045	-44.1175
ZINC08415616	-39.9397	-44.1158
ZINC32541287	-38.9443	-44.1153
ZINC04059703	-40.8089	-44.1124
ZINC04059703	-39.5881	-44.1124
ZINC04059703	-39.5049	-44.1124
ZINC08383902	-42.4081	-44.1083
ZINC08383902	-39.9529	-44.1083
ZINC00726683	-40.1446	-44.1083
ZINC06162380	-42.1871	-44.1077
ZINC06162380	-38.7723	-44.1077
ZINC19938429	-47.6620	-44.1074
ZINC08440508	-42.6195	-44.1064
ZINC08440125	-38.2917	-44.1031
ZINC00205957	-39.8809	-44.1030
ZINC06162487	-40.9788	-44.1021
ZINC13510077	-43.7298	-44.1019
ZINC06197570	-40.1315	-44.1015
ZINC06197570	-38.3123	-44.1015
ZINC00703123	-40.4886	-44.0955
ZINC08430218	-39.8751	-44.0942

ZINC04032168	-36.8242	-44.0932
ZINC04066139	-41.3034	-44.0931
ZINC04066139	-41.1489	-44.0931
ZINC02071242	-45.7008	-44.0919
ZINC04649773	-43.6094	-44.0914
ZINC04649773	-41.7049	-44.0914
ZINC04066453	-39.8735	-44.0914
ZINC04066453	-37.9290	-44.0914
ZINC06137402	-40.7095	-44.0911
ZINC12463187	-41.3297	-44.0886
ZINC12463187	-40.9800	-44.0886
ZINC06162509	-38.4995	-44.0869
ZINC06195656	-42.6838	-44.0857
ZINC06267493	-49.4098	-44.0857
ZINC08439535	-44.0617	-44.0843
ZINC08439535	-40.6212	-44.0843
ZINC06194509	-40.0749	-44.0840
ZINC06194509	-36.9435	-44.0840
ZINC04066570	-39.3934	-44.0823
ZINC04066570	-39.3310	-44.0823
ZINC04112166	-42.3373	-44.0813
ZINC19904608	-39.1730	-44.0813
ZINC06195571	-41.6185	-44.0786
ZINC00726504	-37.2877	-44.0758
ZINC04060787	-40.0970	-44.0750
ZINC04060787	-38.2940	-44.0750
ZINC04060787	-38.1684	-44.0750
ZINC06195485	-42.6708	-44.0738
ZINC04719068	-41.8496	-44.0722
ZINC04719068	-41.0513	-44.0722
ZINC06015494	-41.1964	-44.0720
ZINC00724844	-41.2465	-44.0710
ZINC00726514	-40.7925	-44.0705
ZINC00726514	-40.7700	-44.0705
ZINC00726514	-39.8531	-44.0705
ZINC08425393	-40.9669	-44.0668
ZINC08425393	-39.9951	-44.0668
ZINC08425393	-37.3581	-44.0668
ZINC15837046	-42.8712	-44.0668
ZINC08439596	-41.0335	-44.0650
ZINC08439596	-40.2626	-44.0650
ZINC17044931	-42.0042	-44.0631
ZINC17044931	-41.4523	-44.0631
ZINC19796846	-37.4044	-44.0630
ZINC04059672	-41.1764	-44.0624
ZINC19909086	-39.6618	-44.0607
ZINC18164825	-41.6754	-44.0603
ZINC08996368	-43.5785	-44.0591
ZINC08996368	-42.6142	-44.0591
ZINC04060827	-40.8667	-44.0561
ZINC12468084	-43.0738	-44.0552
ZINC19894473	-40.6132	-44.0549
ZINC01221414	-43.6239	-44.0536
ZINC13571514	-40.4768	-44.0516

ZINC08415592	-43.2891	-44.0510
ZINC08415592	-41.1917	-44.0510
ZINC13477081	-48.5278	-44.0510
ZINC13477081	-45.3557	-44.0510
ZINC00707958	-40.2266	-44.0501
ZINC00707958	-39.7392	-44.0501
ZINC00707958	-38.2202	-44.0501
ZINC08440851	-40.3541	-44.0487
ZINC08996929	-39.5703	-44.0481
ZINC08996929	-36.6991	-44.0481
ZINC16671903	-41.3751	-44.0475
ZINC16671903	-41.1714	-44.0475
ZINC04064845	-42.1287	-44.0470
ZINC04064845	-41.4637	-44.0470
ZINC04064845	-40.6633	-44.0470
ZINC06137209	-40.3327	-44.0465
ZINC08454939	-45.3962	-44.0445
ZINC08416367	-39.7799	-44.0424
ZINC08416367	-38.6876	-44.0424
ZINC08416367	-38.6446	-44.0424
ZINC02082365	-41.6108	-44.0388
ZINC04067779	-40.9960	-44.0376
ZINC08440061	-39.7682	-44.0346
ZINC08440061	-36.9666	-44.0346
ZINC09009105	-40.4009	-44.0346
ZINC09329128	-40.8976	-44.0327
ZINC08415796	-40.6360	-44.0280
ZINC04067029	-39.2494	-44.0262
ZINC09329737	-43.8154	-44.0236
ZINC00703176	-44.6052	-44.0208
ZINC09358271	-39.9711	-44.0202
ZINC09358271	-39.7978	-44.0202
ZINC02071243	-46.2093	-44.0197
ZINC19884633	-41.9415	-44.0194
ZINC19884633	-38.8557	-44.0194
ZINC00631111	-45.5791	-44.0179
ZINC08437422	-38.4928	-44.0162
ZINC08441765	-41.6417	-44.0161
ZINC09373756	-41.0867	-44.0134
ZINC22936097	-42.3288	-44.0104
ZINC19519999	-36.4069	-44.0092
ZINC00710645	-38.6823	-44.0084
ZINC00710645	-38.3647	-44.0084
ZINC04114980	-41.0894	-44.0070
ZINC04114980	-39.7162	-44.0070
ZINC00853773	-38.1605	-44.0045
ZINC32576986	-44.9991	-44.0042
ZINC08444690	-42.0297	-44.0036
ZINC19802256	-39.4084	-44.0023
ZINC08386663	-43.8872	-44.0016
ZINC08386663	-41.2296	-44.0016
ZINC08386663	-40.6762	-44.0016
ZINC01413505	-47.5525	-43.9993
ZINC01413505	-45.0531	-43.9993

ZINC04065134	-38.4554	-43.9976
ZINC04065134	-36.7721	-43.9976
ZINC06137088	-40.1289	-43.9954
ZINC08384488	-43.1556	-43.9947
ZINC04067571	-41.4389	-43.9938
ZINC09123614	-39.1665	-43.9923
ZINC09123614	-38.5898	-43.9923
ZINC08413446	-41.7749	-43.9885
ZINC08387219	-44.1368	-43.9874
ZINC00970963	-47.2490	-43.9867
ZINC17918223	-40.2932	-43.9853
ZINC06197213	-43.4014	-43.9842
ZINC06197213	-42.9613	-43.9842
ZINC09302057	-43.4922	-43.9841
ZINC19801790	-37.9838	-43.9838
ZINC00866539	-41.8325	-43.9807
ZINC00866539	-39.4088	-43.9807
ZINC08743985	-39.5320	-43.9797
ZINC19364417	-45.3374	-43.9778
ZINC18193061	-43.2784	-43.9754
ZINC18193061	-39.0306	-43.9754
ZINC02057192	-43.6050	-43.9754
ZINC08996385	-40.8727	-43.9753
ZINC08996385	-39.6304	-43.9753
ZINC08439735	-39.5966	-43.9752
ZINC08439735	-39.1978	-43.9752
ZINC08439735	-37.8998	-43.9752
ZINC05044908	-37.0356	-43.9724
ZINC19911612	-39.4802	-43.9719
ZINC09349791	-45.0045	-43.9718
ZINC20290577	-47.4287	-43.9686
ZINC06149852	-41.4924	-43.9659
ZINC06149852	-41.3924	-43.9659
ZINC18254916	-41.0183	-43.9658
ZINC22802779	-41.8205	-43.9648
ZINC22936465	-37.8349	-43.9629
ZINC08437293	-42.5890	-43.9624
ZINC19363166	-37.1380	-43.9593
ZINC00703050	-42.9637	-43.9579
ZINC00703050	-42.6983	-43.9579
ZINC02843341	-41.0706	-43.9556
ZINC08425389	-39.1624	-43.9530
ZINC04067189	-41.1870	-43.9513
ZINC04067189	-41.1538	-43.9513
ZINC01413498	-44.0313	-43.9494
ZINC01413498	-43.7018	-43.9494
ZINC08440596	-40.7974	-43.9484
ZINC08440596	-38.7444	-43.9484
ZINC08440596	-37.8950	-43.9484
ZINC19872514	-41.6979	-43.9480
ZINC06194190	-39.4614	-43.9470
ZINC18146134	-40.7510	-43.9467
ZINC18146134	-40.5268	-43.9467
ZINC08441770	-41.2663	-43.9467

ZINC06138410	-37.2077	-43.9441
ZINC08441262	-41.0959	-43.9413
ZINC08441262	-39.8842	-43.9413
ZINC19323813	-39.0701	-43.9410
ZINC09379915	-43.1817	-43.9398
ZINC09379915	-42.6199	-43.9398
ZINC04417989	-40.8630	-43.9333
ZINC04417989	-38.1324	-43.9333
ZINC09379795	-40.3394	-43.9317
ZINC09379795	-40.1177	-43.9317
ZINC19938350	-49.4979	-43.9310
ZINC08383318	-38.3567	-43.9308
ZINC06444686	-41.1413	-43.9295
ZINC06444686	-40.4235	-43.9295
ZINC13388583	-39.5137	-43.9290
ZINC06149843	-41.4260	-43.9250
ZINC06149843	-37.7197	-43.9250
ZINC19790956	-38.6210	-43.9233
ZINC02183689	-38.7857	-43.9232
ZINC16672099	-41.7322	-43.9197
ZINC16672099	-41.4138	-43.9197
ZINC04067803	-43.4334	-43.9189
ZINC00703149	-42.4981	-43.9187
ZINC05490363	-40.1423	-43.9151
ZINC09463630	-43.7164	-43.9110
ZINC04060822	-40.2206	-43.9087
ZINC08981598	-45.8749	-43.9083
ZINC08981598	-44.4919	-43.9083
ZINC08981598	-43.2497	-43.9083
ZINC08981598	-42.7372	-43.9083
ZINC08442177	-36.3823	-43.9072
ZINC06195589	-41.4429	-43.9033
ZINC06195589	-39.3080	-43.9033
ZINC06162005	-39.7875	-43.9028
ZINC06197661	-37.2983	-43.9024
ZINC13451192	-45.9208	-43.9020
ZINC13451192	-44.8086	-43.9020
ZINC06015484	-37.6689	-43.9020
ZINC08384140	-40.5622	-43.9017
ZINC08384140	-39.7195	-43.9017
ZINC00639927	-46.3049	-43.9003
ZINC04065986	-38.6637	-43.8998
ZINC04125960	-40.6818	-43.8996
ZINC04125960	-38.5470	-43.8996
ZINC08441440	-39.6124	-43.8992
ZINC09271193	-45.2985	-43.8949
ZINC09271193	-41.0411	-43.8949
ZINC03877827	-40.4605	-43.8876
ZINC08695206	-48.3699	-43.8874
ZINC09354092	-40.1051	-43.8872
ZINC04066902	-36.7359	-43.8862
ZINC00703124	-42.3326	-43.8861
ZINC04112281	-42.7890	-43.8834
ZINC04112281	-41.1555	-43.8834

ZINC18209796	-41.3991	-43.8789
ZINC18209796	-36.8837	-43.8789
ZINC22937034	-42.7884	-43.8776
ZINC06196099	-40.3904	-43.8679
ZINC04285207	-39.8717	-43.8670
ZINC00707961	-39.3588	-43.8645
ZINC00707961	-39.1631	-43.8645
ZINC04044194	-39.2064	-43.8637
ZINC18007503	-39.4479	-43.8622
ZINC18007503	-36.8420	-43.8622
ZINC08415967	-40.3635	-43.8622
ZINC04065897	-40.4800	-43.8579
ZINC04065897	-38.4857	-43.8579
ZINC09360289	-41.0683	-43.8564
ZINC06187051	-40.8475	-43.8557
ZINC13958096	-40.3788	-43.8553
ZINC13958096	-37.1862	-43.8553
ZINC85425765	-42.9380	-43.8548
ZINC06149648	-38.7328	-43.8547
ZINC00386316	-40.6712	-43.8529
ZINC08416009	-41.0310	-43.8527
ZINC08416009	-40.7921	-43.8527
ZINC08416009	-39.9952	-43.8527
ZINC08996455	-42.6137	-43.8516
ZINC06162021	-40.3291	-43.8514
ZINC00702731	-40.5431	-43.8512
ZINC08399137	-42.3476	-43.8511
ZINC08399137	-40.3568	-43.8511
ZINC20547487	-44.7118	-43.8509
ZINC20547487	-42.2599	-43.8509
ZINC20547487	-41.3337	-43.8509
ZINC08410578	-37.9812	-43.8500
ZINC01414773	-42.7893	-43.8469
ZINC01414773	-41.1765	-43.8469
ZINC09380391	-43.8065	-43.8469
ZINC09380391	-41.5221	-43.8469
ZINC13470893	-44.9366	-43.8454
ZINC13470893	-43.8038	-43.8454
ZINC09236150	-44.5205	-43.8438
ZINC09236150	-40.7172	-43.8438
ZINC04112168	-41.9697	-43.8404
ZINC04112168	-38.8457	-43.8404
ZINC08426355	-44.2969	-43.8390
ZINC06812803	-41.2531	-43.8356
ZINC06194157	-40.4593	-43.8337
ZINC06194157	-38.9653	-43.8337
ZINC08425387	-40.6143	-43.8329
ZINC09042785	-41.5165	-43.8321
ZINC09042785	-40.0715	-43.8321
ZINC00702749	-43.0380	-43.8298
ZINC00702749	-41.3548	-43.8298
ZINC06137515	-40.4743	-43.8294
ZINC04066090	-40.9970	-43.8279
ZINC04066090	-36.8875	-43.8279

ZINC04067562	-38.1056	-43.8279
ZINC04067562	-36.9144	-43.8279
ZINC08996789	-41.2789	-43.8251
ZINC08996789	-39.8926	-43.8251
ZINC02100280	-46.1593	-43.8247
ZINC06194435	-40.8215	-43.8247
ZINC06194435	-37.2762	-43.8247
ZINC20109754	-37.9138	-43.8246
ZINC00853875	-41.4147	-43.8226
ZINC00853875	-41.2908	-43.8226
ZINC00853875	-39.5265	-43.8226
ZINC19872254	-39.3448	-43.8183
ZINC06197277	-40.0219	-43.8140
ZINC17720436	-41.4120	-43.8130
ZINC17720436	-40.8901	-43.8130
ZINC00968726	-39.3670	-43.8093
ZINC08914642	-42.8808	-43.8071
ZINC08914642	-40.9293	-43.8071
ZINC06162286	-39.8998	-43.8031
ZINC06162286	-39.1786	-43.8031
ZINC19371719	-38.1068	-43.7997
ZINC04066279	-42.4863	-43.7991
ZINC04066279	-41.3832	-43.7991
ZINC08715701	-42.6434	-43.7987
ZINC08715701	-36.7361	-43.7987
ZINC06161996	-40.8062	-43.7985
ZINC06161996	-39.4393	-43.7985
ZINC08411668	-39.9212	-43.7969
ZINC08411668	-39.3881	-43.7969
ZINC00689392	-38.8514	-43.7961
ZINC00894444	-41.5822	-43.7961
ZINC00894444	-37.7749	-43.7961
ZINC00625480	-39.1159	-43.7958
ZINC00625480	-38.6284	-43.7958
ZINC09374214	-41.3306	-43.7941
ZINC08442209	-42.5788	-43.7931
ZINC01414764	-42.3294	-43.7928
ZINC08440202	-44.2279	-43.7928
ZINC13774955	-44.6641	-43.7907
ZINC09272860	-40.2241	-43.7904
ZINC09272860	-40.2241	-43.7904
ZINC09272860	-38.4461	-43.7904
ZINC19938472	-46.7858	-43.7903
ZINC09110448	-41.2207	-43.7901
ZINC09110448	-38.8484	-43.7901
ZINC05578299	-40.4139	-43.7884
ZINC08860015	-41.4601	-43.7883
ZINC08397814	-36.4289	-43.7870
ZINC08440973	-43.3602	-43.7868
ZINC08440964	-46.9716	-43.7864
ZINC00386315	-42.4923	-43.7851
ZINC33921650	-39.9503	-43.7774
ZINC08450383	-39.1781	-43.7771
ZINC18182993	-40.0577	-43.7768

ZINC05800391	-39.3376	-43.7767
ZINC06303157	-38.7117	-43.7760
ZINC05920823	-45.2605	-43.7733
ZINC05920823	-41.8445	-43.7733
ZINC06013559	-50.2018	-43.7729
ZINC06195744	-39.4094	-43.7690
ZINC06162044	-41.0801	-43.7647
ZINC09354156	-40.6482	-43.7581
ZINC09186812	-45.8586	-43.7578
ZINC09186812	-43.1528	-43.7578
ZINC09186812	-42.7535	-43.7578
ZINC09186812	-42.5482	-43.7578
ZINC04148572	-39.4584	-43.7559
ZINC04148572	-37.9274	-43.7559
ZINC08439834	-39.6760	-43.7536
ZINC08444590	-38.8804	-43.7514
ZINC13424226	-39.2350	-43.7512
ZINC06195534	-40.3264	-43.7484
ZINC02181900	-39.6812	-43.7479
ZINC08399177	-39.4826	-43.7475
ZINC08399177	-37.9054	-43.7475
ZINC04048079	-41.1845	-43.7468
ZINC04048079	-39.0021	-43.7468
ZINC09411626	-42.0505	-43.7462
ZINC09411626	-39.0738	-43.7462
ZINC08837393	-43.4730	-43.7434
ZINC08437428	-39.0199	-43.7411
ZINC08439375	-42.1760	-43.7406
ZINC08439375	-41.9917	-43.7406
ZINC08439375	-41.8619	-43.7406
ZINC09273572	-44.0442	-43.7402
ZINC06196080	-42.5685	-43.7401
ZINC08438548	-39.6459	-43.7380
ZINC08817380	-40.0054	-43.7379
ZINC04060759	-39.0729	-43.7371
ZINC04060759	-37.8531	-43.7371
ZINC19582941	-45.8023	-43.7331
ZINC19802428	-36.3295	-43.7305
ZINC19852804	-44.6916	-43.7304
ZINC04438884	-41.8729	-43.7284
ZINC04438884	-39.6608	-43.7284
ZINC19374581	-39.3539	-43.7275
ZINC85433940	-39.2531	-43.7269
ZINC04681760	-40.6053	-43.7251
ZINC04063000	-41.0766	-43.7238
ZINC04063000	-40.6364	-43.7238
ZINC04063000	-40.4575	-43.7238
ZINC06197379	-42.1984	-43.7231
ZINC06197379	-42.0938	-43.7231
ZINC10313190	-43.1003	-43.7227
ZINC04065840	-41.2930	-43.7197
ZINC09374352	-41.9704	-43.7192
ZINC09374352	-41.5569	-43.7192
ZINC08399188	-42.5261	-43.7192

ZINC06194187	-39.9276	-43.7170
ZINC06194187	-36.6296	-43.7170
ZINC00702733	-42.1269	-43.7163
ZINC00702733	-41.8010	-43.7163
ZINC04350907	-41.6949	-43.7146
ZINC04660097	-43.5538	-43.7146
ZINC00724932	-40.3745	-43.7131
ZINC08441551	-39.6028	-43.7130
ZINC00726513	-39.1630	-43.7130
ZINC00726513	-39.1541	-43.7130
ZINC06148626	-40.7455	-43.7118
ZINC04066102	-37.1659	-43.7059
ZINC04066102	-36.8319	-43.7059
ZINC08410677	-44.4900	-43.7054
ZINC08416060	-38.4294	-43.7053
ZINC00726649	-40.0768	-43.7045
ZINC00726649	-38.7713	-43.7045
ZINC00726649	-38.2119	-43.7045
ZINC00647152	-43.2603	-43.7030
ZINC09403376	-45.3541	-43.7023
ZINC09403376	-43.6156	-43.7023
ZINC02058004	-43.6119	-43.7016
ZINC04067440	-41.9405	-43.6980
ZINC04067440	-39.5714	-43.6980
ZINC15971612	-43.1187	-43.6890
ZINC00625470	-38.9711	-43.6859
ZINC00625470	-37.5815	-43.6859
ZINC08441761	-42.3796	-43.6843
ZINC04067143	-41.3844	-43.6839
ZINC04067143	-40.6999	-43.6839
ZINC16115515	-41.0795	-43.6829
ZINC16115515	-37.6977	-43.6829
ZINC00707611	-39.5454	-43.6806
ZINC00707611	-38.3787	-43.6806
ZINC00707611	-36.6507	-43.6806
ZINC02183624	-38.4446	-43.6799
ZINC08625459	-41.6776	-43.6797
ZINC04061537	-40.4205	-43.6796
ZINC04061537	-37.5829	-43.6796
ZINC04061537	-36.6962	-43.6796
ZINC00724846	-43.2733	-43.6793
ZINC08439856	-41.0074	-43.6763
ZINC08439856	-40.6661	-43.6763
ZINC09328510	-45.1207	-43.6757
ZINC08437234	-42.2270	-43.6755
ZINC08437419	-38.0439	-43.6732
ZINC00626165	-44.2819	-43.6703
ZINC19533498	-41.0623	-43.6698
ZINC00680132	-38.3841	-43.6664
ZINC19907585	-43.2659	-43.6657
ZINC19907585	-41.4599	-43.6657
ZINC19907585	-40.6791	-43.6657
ZINC09009155	-40.3503	-43.6647
ZINC09009155	-40.0462	-43.6647

ZINC06194173	-38.4692	-43.6644
ZINC04114979	-39.8834	-43.6643
ZINC04114979	-39.1757	-43.6643
ZINC12867889	-39.8239	-43.6638
ZINC04065980	-40.9133	-43.6591
ZINC04065980	-39.1251	-43.6591
ZINC16946073	-42.6919	-43.6560
ZINC06197088	-42.3486	-43.6554
ZINC04114384	-37.3365	-43.6544
ZINC08439500	-39.0442	-43.6511
ZINC04066593	-39.5872	-43.6510
ZINC04066593	-39.1547	-43.6510
ZINC08438743	-42.2054	-43.6508
ZINC19901324	-42.9025	-43.6479
ZINC06195463	-39.3711	-43.6477
ZINC06161960	-38.2463	-43.6468
ZINC06442958	-39.3508	-43.6436
ZINC08430694	-43.0899	-43.6415
ZINC08430694	-43.0706	-43.6415
ZINC08430694	-41.3395	-43.6415
ZINC06162444	-40.7097	-43.6404
ZINC04067260	-44.4463	-43.6382
ZINC05409055	-40.9648	-43.6379
ZINC05409055	-39.5585	-43.6379
ZINC08439319	-40.1447	-43.6378
ZINC08439319	-38.8855	-43.6378
ZINC08996589	-42.2425	-43.6369
ZINC02078825	-41.8236	-43.6352
ZINC02078825	-41.4102	-43.6352
ZINC02078825	-40.8020	-43.6352
ZINC02078825	-38.8100	-43.6352
ZINC17124561	-46.5193	-43.6345
ZINC00972605	-40.2365	-43.6337
ZINC10279511	-37.4203	-43.6299
ZINC08715697	-43.9207	-43.6290
ZINC08715697	-42.9905	-43.6290
ZINC19552823	-37.0114	-43.6285
ZINC13554814	-39.0104	-43.6276
ZINC09272835	-45.8673	-43.6235
ZINC09272835	-45.5579	-43.6235
ZINC09330721	-45.7043	-43.6233
ZINC17197669	-40.2857	-43.6228
ZINC04067478	-40.4025	-43.6223
ZINC04632295	-40.8899	-43.6221
ZINC04632295	-40.7095	-43.6221
ZINC08440897	-40.9124	-43.6214
ZINC08416388	-39.9955	-43.6197
ZINC04284875	-42.9358	-43.6192
ZINC04284875	-41.6201	-43.6192
ZINC08411353	-42.1082	-43.6187
ZINC06195542	-39.7681	-43.6182
ZINC19287399	-44.2945	-43.6172
ZINC00999397	-42.5190	-43.6167
ZINC13174336	-44.0502	-43.6162

ZINC08425321	-39.2792	-43.6148
ZINC08425321	-38.9945	-43.6148
ZINC08425321	-38.6276	-43.6148
ZINC04062294	-39.8592	-43.6135
ZINC04062294	-38.3005	-43.6135
ZINC06161973	-42.3453	-43.6124
ZINC06161973	-39.8892	-43.6124
ZINC06196111	-38.9287	-43.6087
ZINC04066552	-38.8116	-43.6069
ZINC04066552	-37.3198	-43.6069
ZINC19323816	-39.2098	-43.6063
ZINC05408946	-38.7701	-43.6050
ZINC05408946	-38.6555	-43.6050
ZINC04059659	-40.8920	-43.6030
ZINC19781865	-37.4172	-43.6020
ZINC08383544	-46.6985	-43.5967
ZINC08383544	-44.5481	-43.5967
ZINC04067386	-41.3532	-43.5952
ZINC04067386	-40.8952	-43.5952
ZINC04067386	-38.7110	-43.5952
ZINC05895022	-39.5481	-43.5939
ZINC08415631	-40.6868	-43.5911
ZINC06195735	-38.1905	-43.5902
ZINC02080288	-42.4478	-43.5885
ZINC00626098	-43.7634	-43.5873
ZINC00708321	-39.4888	-43.5869
ZINC13566932	-42.2890	-43.5857
ZINC09243849	-41.9371	-43.5844
ZINC00702477	-37.9665	-43.5839
ZINC00702477	-37.4941	-43.5839
ZINC00702477	-37.0183	-43.5839
ZINC04061555	-40.5298	-43.5823
ZINC04061555	-39.7566	-43.5823
ZINC04061555	-39.5467	-43.5823
ZINC06444495	-39.8209	-43.5820
ZINC09349789	-44.6179	-43.5803
ZINC09349789	-43.4672	-43.5803
ZINC00841900	-41.9929	-43.5801
ZINC00841900	-41.7174	-43.5801
ZINC00841900	-40.3335	-43.5801
ZINC00726632	-38.0061	-43.5797
ZINC08413142	-42.7753	-43.5791
ZINC08413142	-42.2905	-43.5791
ZINC00708158	-39.6655	-43.5761
ZINC06781550	-40.1460	-43.5756
ZINC08416362	-40.7138	-43.5726
ZINC17201290	-41.0366	-43.5704
ZINC08914039	-45.2262	-43.5697
ZINC09008979	-40.0618	-43.5683
ZINC06148728	-40.1139	-43.5676
ZINC08439764	-37.5276	-43.5670
ZINC08439764	-36.4374	-43.5670
ZINC06196510	-40.1888	-43.5663
ZINC05919684	-40.3579	-43.5661

ZINC05919684	-40.1841	-43.5661
ZINC08440918	-43.8372	-43.5655
ZINC05396721	-39.9383	-43.5628
ZINC05396721	-39.7727	-43.5628
ZINC05396721	-39.0798	-43.5628
ZINC09460780	-45.0592	-43.5617
ZINC00820672	-40.2617	-43.5602
ZINC00820672	-36.7743	-43.5602
ZINC08440386	-38.8822	-43.5600
ZINC08440386	-38.0293	-43.5600
ZINC08440386	-37.1001	-43.5600
ZINC20477791	-38.3065	-43.5582
ZINC19880820	-40.5673	-43.5571
ZINC00723933	-37.9771	-43.5541
ZINC09045848	-43.5053	-43.5534
ZINC08442168	-39.8560	-43.5534
ZINC06197499	-41.1253	-43.5531
ZINC08816970	-46.8284	-43.5525
ZINC04068159	-42.0509	-43.5518
ZINC04068159	-39.5447	-43.5518
ZINC04068159	-38.9276	-43.5518
ZINC00641070	-43.3305	-43.5498
ZINC08441375	-37.5242	-43.5482
ZINC08441375	-36.3382	-43.5482
ZINC12373955	-43.7030	-43.5476
ZINC04626480	-39.8459	-43.5440
ZINC08396517	-39.1982	-43.5432
ZINC02082891	-41.9639	-43.5430
ZINC08437340	-41.8187	-43.5427
ZINC08437340	-39.7477	-43.5427
ZINC10313278	-48.6777	-43.5423
ZINC08444346	-40.6690	-43.5417
ZINC08444346	-39.9681	-43.5417
ZINC04059731	-38.8223	-43.5409
ZINC04059731	-37.9781	-43.5409
ZINC12373962	-41.8808	-43.5387
ZINC13372066	-40.6477	-43.5366
ZINC13372066	-39.2566	-43.5366
ZINC01413491	-46.3711	-43.5327
ZINC04066091	-38.8961	-43.5287
ZINC04066091	-37.2181	-43.5287
ZINC06136920	-41.0687	-43.5271
ZINC06136920	-38.0668	-43.5271
ZINC06136920	-37.3558	-43.5271
ZINC09302188	-40.1969	-43.5265
ZINC09302188	-40.0054	-43.5265
ZINC04457673	-39.2725	-43.5244
ZINC06321516	-42.5797	-43.5233
ZINC04064593	-38.7435	-43.5218
ZINC19363163	-38.7244	-43.5201
ZINC32541291	-41.4131	-43.5200
ZINC08680885	-46.6408	-43.5186
ZINC00709903	-40.8447	-43.5173
ZINC02270223	-40.1084	-43.5138

ZINC49200986	-40.1939	-43.5098
ZINC04066306	-41.3044	-43.5092
ZINC04066306	-40.8964	-43.5092
ZINC08416012	-43.3093	-43.5091
ZINC08441032	-39.4777	-43.5085
ZINC08441032	-38.8129	-43.5085
ZINC08441032	-38.6262	-43.5085
ZINC03368323	-50.4000	-43.5083
ZINC09272673	-44.3648	-43.5048
ZINC00716659	-38.4283	-43.5011
ZINC00716659	-37.0731	-43.5011
ZINC04284847	-40.1513	-43.4996
ZINC04284847	-38.8858	-43.4996
ZINC08415351	-38.6791	-43.4988
ZINC09110530	-39.2820	-43.4974
ZINC08439423	-39.9177	-43.4942
ZINC08439423	-39.9016	-43.4942
ZINC08439423	-38.8386	-43.4942
ZINC08413592	-39.3925	-43.4931
ZINC19369760	-40.8985	-43.4929
ZINC19369760	-40.3726	-43.4929
ZINC06238819	-39.4978	-43.4929
ZINC06238819	-38.1897	-43.4929
ZINC09244238	-42.0180	-43.4916
ZINC09244238	-41.5850	-43.4916
ZINC06195123	-40.5536	-43.4916
ZINC06195123	-37.2840	-43.4916
ZINC08440869	-38.7551	-43.4905
ZINC08440869	-38.5854	-43.4905
ZINC19871617	-38.3434	-43.4894
ZINC08440100	-45.4985	-43.4884
ZINC08440100	-44.4862	-43.4884
ZINC04067493	-37.9415	-43.4868
ZINC01413501	-46.9278	-43.4862
ZINC01413501	-45.3739	-43.4862
ZINC09088877	-40.7475	-43.4829
ZINC09088877	-40.1611	-43.4829
ZINC19832135	-37.2113	-43.4751
ZINC08439487	-48.1033	-43.4750
ZINC05631607	-39.3287	-43.4748
ZINC18031835	-42.2486	-43.4745
ZINC06195637	-40.3917	-43.4740
ZINC04112162	-41.8071	-43.4738
ZINC04112162	-39.9085	-43.4738
ZINC04112162	-39.2548	-43.4738
ZINC08744093	-42.2900	-43.4720
ZINC04284876	-41.1479	-43.4708
ZINC04066621	-42.1451	-43.4683
ZINC04066621	-40.7787	-43.4683
ZINC06467881	-37.4545	-43.4661
ZINC00703129	-41.7871	-43.4656
ZINC09359266	-42.2200	-43.4649
ZINC09359266	-42.1908	-43.4649
ZINC17180540	-44.5698	-43.4623

ZINC17180540	-44.0899	-43.4623
ZINC17180540	-43.3654	-43.4623
ZINC17180540	-41.4932	-43.4623
ZINC17180540	-41.2812	-43.4623
ZINC08414893	-39.3488	-43.4620
ZINC08414893	-37.9304	-43.4620
ZINC04613418	-40.0544	-43.4614
ZINC09242360	-41.0703	-43.4603
ZINC19872216	-40.9566	-43.4599
ZINC08439767	-37.2672	-43.4596
ZINC08439767	-36.4610	-43.4596
ZINC09243545	-41.2841	-43.4593
ZINC09243545	-39.3226	-43.4593
ZINC05947024	-44.9764	-43.4585
ZINC08425578	-39.3869	-43.4580
ZINC08425578	-38.6486	-43.4580
ZINC08425578	-38.4426	-43.4580
ZINC19527247	-36.7804	-43.4550
ZINC08416254	-41.4027	-43.4547
ZINC08416254	-39.1633	-43.4547
ZINC19841519	-40.6170	-43.4526
ZINC08441473	-43.4809	-43.4521
ZINC08441473	-43.3407	-43.4521
ZINC08444533	-43.0186	-43.4518
ZINC08437416	-41.5811	-43.4517
ZINC08437416	-38.8670	-43.4517
ZINC59817733	-39.5141	-43.4472
ZINC08413452	-45.2679	-43.4443
ZINC08413452	-42.9574	-43.4443
ZINC06027824	-47.0861	-43.4433
ZINC18276309	-40.1449	-43.4429
ZINC18276309	-38.8282	-43.4429
ZINC18276309	-37.5802	-43.4429
ZINC16115513	-37.7564	-43.4419
ZINC08383649	-42.3912	-43.4404
ZINC19533496	-37.1959	-43.4397
ZINC20414331	-39.1583	-43.4386
ZINC08426368	-45.1016	-43.4380
ZINC08426368	-42.4663	-43.4380
ZINC08426368	-42.3674	-43.4380
ZINC20032118	-43.0467	-43.4369
ZINC16579300	-42.7758	-43.4364
ZINC16579300	-41.0036	-43.4364
ZINC00708235	-38.4320	-43.4356
ZINC01246478	-42.7561	-43.4356
ZINC01414772	-45.1292	-43.4338
ZINC01414772	-42.7026	-43.4338
ZINC02063828	-44.0178	-43.4320
ZINC08430164	-41.3269	-43.4292
ZINC08425205	-38.7137	-43.4289
ZINC18121755	-40.0679	-43.4276
ZINC06015475	-39.8203	-43.4269
ZINC12138428	-39.6645	-43.4267
ZINC19901146	-47.7264	-43.4262

ZINC19550255	-40.1851	-43.4225
ZINC19922802	-41.3105	-43.4213
ZINC08425570	-38.9909	-43.4195
ZINC08425570	-38.2498	-43.4195
ZINC08425570	-36.9568	-43.4195
ZINC09235804	-41.4843	-43.4147
ZINC18114177	-37.1714	-43.4131
ZINC16115457	-41.2363	-43.4127
ZINC16115457	-38.6083	-43.4127
ZINC08439316	-39.7077	-43.4086
ZINC01269362	-43.3506	-43.4078
ZINC04396931	-41.8583	-43.4064
ZINC08450384	-38.9113	-43.4029
ZINC09012381	-42.9661	-43.3934
ZINC06144031	-44.5174	-43.3903
ZINC06144031	-44.2460	-43.3903
ZINC00707869	-40.6318	-43.3896
ZINC08441150	-39.4616	-43.3894
ZINC08441150	-37.0619	-43.3894
ZINC08441150	-36.2797	-43.3894
ZINC00799449	-37.9251	-43.3889
ZINC85425768	-42.8466	-43.3860
ZINC85425768	-39.6441	-43.3860
ZINC00844271	-37.1556	-43.3837
ZINC09015844	-38.4669	-43.3821
ZINC28251942	-40.7685	-43.3821
ZINC04065964	-36.6378	-43.3813
ZINC05488882	-39.6298	-43.3789
ZINC05488882	-38.4438	-43.3789
ZINC04313814	-43.8499	-43.3777
ZINC08442031	-40.0685	-43.3766
ZINC04454195	-41.1585	-43.3763
ZINC08686431	-41.7308	-43.3746
ZINC06195464	-42.1586	-43.3745
ZINC06195464	-39.8939	-43.3745
ZINC09360324	-39.6651	-43.3740
ZINC09360324	-38.9699	-43.3740
ZINC06137170	-38.5701	-43.3738
ZINC00674247	-40.8139	-43.3731
ZINC05898173	-46.6959	-43.3712
ZINC59186199	-40.2556	-43.3711
ZINC22936823	-39.2873	-43.3698
ZINC04062924	-40.1180	-43.3682
ZINC04062924	-37.8716	-43.3682
ZINC04062924	-36.9605	-43.3682
ZINC04068319	-38.6937	-43.3648
ZINC04068319	-36.9201	-43.3648
ZINC06194132	-41.9372	-43.3641
ZINC04064374	-38.5358	-43.3637
ZINC00801131	-42.5318	-43.3636
ZINC00801131	-40.3197	-43.3636
ZINC09424272	-43.3439	-43.3627
ZINC09424272	-43.3244	-43.3627
ZINC00851613	-42.7731	-43.3612

ZINC08438693	-41.8541	-43.3563
ZINC08438693	-41.7370	-43.3563
ZINC19369478	-40.9050	-43.3556
ZINC08837498	-43.9669	-43.3553
ZINC08837498	-43.4830	-43.3553
ZINC08996610	-39.9256	-43.3552
ZINC02060069	-46.9100	-43.3542
ZINC08439494	-37.3149	-43.3537
ZINC08439494	-36.9071	-43.3537
ZINC09329846	-41.3814	-43.3523
ZINC19360192	-37.6307	-43.3519
ZINC19360192	-37.2109	-43.3519
ZINC08996974	-45.5744	-43.3507
ZINC08399155	-39.5689	-43.3502
ZINC08399155	-38.8274	-43.3502
ZINC01414774	-44.9370	-43.3496
ZINC01414774	-43.9003	-43.3496
ZINC06195951	-38.5691	-43.3486
ZINC22107710	-42.8082	-43.3471
ZINC00631024	-45.8772	-43.3450
ZINC05691045	-38.4931	-43.3445
ZINC05691045	-37.3508	-43.3445
ZINC08413539	-40.2213	-43.3437
ZINC08413539	-39.0665	-43.3437
ZINC09014931	-40.5160	-43.3434
ZINC00726577	-40.3847	-43.3429
ZINC00726577	-40.2214	-43.3429
ZINC00726577	-39.4923	-43.3429
ZINC16114949	-39.9112	-43.3428
ZINC06162484	-40.7903	-43.3419
ZINC00626544	-44.2036	-43.3414
ZINC00626544	-43.7983	-43.3414
ZINC04067500	-37.2435	-43.3395
ZINC04067500	-36.9035	-43.3395
ZINC08441451	-39.3724	-43.3370
ZINC08441451	-38.9974	-43.3370
ZINC04068010	-42.3770	-43.3369
ZINC04068010	-41.8363	-43.3369
ZINC04068010	-41.0355	-43.3369
ZINC08413407	-39.1176	-43.3368
ZINC08413407	-37.5071	-43.3368
ZINC06144555	-43.5349	-43.3362
ZINC00702988	-40.5919	-43.3354
ZINC04062876	-40.2585	-43.3353
ZINC04062876	-40.1858	-43.3353
ZINC04062876	-40.1452	-43.3353
ZINC09411400	-45.8955	-43.3343
ZINC09411400	-41.9619	-43.3343
ZINC15952862	-46.4452	-43.3332
ZINC08413908	-41.2119	-43.3323
ZINC08413908	-40.9439	-43.3323
ZINC05488584	-39.6499	-43.3283
ZINC04114965	-41.3148	-43.3268
ZINC00847631	-43.3386	-43.3265

ZINC00847631	-42.9351	-43.3265
ZINC18045543	-40.6899	-43.3264
ZINC18045543	-39.6630	-43.3264
ZINC05450562	-42.4146	-43.3256
ZINC05450562	-41.5047	-43.3256
ZINC08836022	-47.1631	-43.3248
ZINC08383731	-45.2597	-43.3234
ZINC08439428	-39.6136	-43.3232
ZINC08439428	-38.8143	-43.3232
ZINC03833255	-39.2966	-43.3230
ZINC18156250	-44.1117	-43.3222
ZINC08399184	-40.8637	-43.3210
ZINC08440769	-39.4277	-43.3201
ZINC08440769	-38.2524	-43.3201
ZINC00726541	-37.0015	-43.3201
ZINC55231645	-41.6683	-43.3200
ZINC08996345	-40.3625	-43.3189
ZINC01414766	-44.9498	-43.3178
ZINC01414766	-41.9015	-43.3178
ZINC04066609	-41.0551	-43.3133
ZINC04066609	-37.7425	-43.3133
ZINC09403375	-43.5208	-43.3132
ZINC09403375	-41.1088	-43.3132
ZINC08440750	-40.1099	-43.3132
ZINC06148826	-40.9887	-43.3125
ZINC08440998	-38.2078	-43.3124
ZINC04067277	-46.2092	-43.3094
ZINC04067277	-45.7022	-43.3094
ZINC04067277	-45.4432	-43.3094
ZINC75273129	-38.7093	-43.3081
ZINC08415751	-37.4544	-43.3068
ZINC04067247	-40.4279	-43.3068
ZINC08385414	-40.9997	-43.3053
ZINC08385414	-40.1013	-43.3053
ZINC08399171	-42.0516	-43.3020
ZINC06197508	-38.5593	-43.3019
ZINC17154953	-40.9895	-43.3019
ZINC01413335	-42.1715	-43.3015
ZINC08695208	-47.9812	-43.3012
ZINC04019582	-43.4097	-43.3009
ZINC04019582	-40.8093	-43.3009
ZINC09042923	-43.3698	-43.2998
ZINC09042923	-43.1021	-43.2998
ZINC11197760	-41.1387	-43.2992
ZINC19366854	-43.2651	-43.2991
ZINC06148608	-38.1840	-43.2975
ZINC37858802	-40.8463	-43.2955
ZINC37858802	-39.8073	-43.2955
ZINC18137618	-39.0299	-43.2945
ZINC18137618	-37.9098	-43.2945
ZINC04060008	-39.5043	-43.2920
ZINC04066191	-37.5885	-43.2913
ZINC04412478	-42.3738	-43.2860
ZINC32497010	-39.9906	-43.2843

ZINC08441254	-39.7389	-43.2829
ZINC08441254	-36.4397	-43.2829
ZINC08441254	-36.3147	-43.2829
ZINC08437223	-36.4211	-43.2824
ZINC00989518	-42.6487	-43.2816
ZINC06136914	-39.7257	-43.2816
ZINC06136914	-38.6672	-43.2816
ZINC06136914	-37.7065	-43.2816
ZINC08440894	-44.1818	-43.2807
ZINC00861135	-40.7634	-43.2762
ZINC00861135	-39.9607	-43.2762
ZINC04067994	-38.7978	-43.2760
ZINC04067994	-38.4316	-43.2760
ZINC04067994	-37.3483	-43.2760
ZINC00726629	-41.1038	-43.2750
ZINC00726629	-40.5117	-43.2750
ZINC00726629	-39.8029	-43.2750
ZINC08396793	-42.4156	-43.2725
ZINC04112279	-39.7761	-43.2718
ZINC04112279	-39.6591	-43.2718
ZINC08439454	-45.7390	-43.2714
ZINC08439454	-44.8789	-43.2714
ZINC08432887	-36.3808	-43.2713
ZINC08430353	-38.5298	-43.2674
ZINC13000365	-39.8918	-43.2673
ZINC04062844	-40.7088	-43.2669
ZINC04062844	-40.4051	-43.2669
ZINC04062844	-39.6294	-43.2669
ZINC13108858	-46.2559	-43.2657
ZINC06195675	-39.9486	-43.2652
ZINC06195675	-39.5828	-43.2652
ZINC04019652	-43.1322	-43.2629
ZINC08837497	-48.4304	-43.2604
ZINC01019953	-40.7341	-43.2600
ZINC01019953	-40.1824	-43.2600
ZINC08970006	-39.5289	-43.2600
ZINC04065656	-39.1459	-43.2586
ZINC04065656	-37.8370	-43.2586
ZINC04065656	-37.2305	-43.2586
ZINC08438771	-44.6821	-43.2567
ZINC04632423	-47.1102	-43.2565
ZINC04632423	-44.3064	-43.2565
ZINC06015531	-37.8721	-43.2553
ZINC06015531	-36.8580	-43.2553
ZINC04067344	-38.7701	-43.2551
ZINC04047855	-37.0332	-43.2520
ZINC08410114	-38.8760	-43.2502
ZINC08410114	-38.7369	-43.2502
ZINC06162507	-39.2869	-43.2495
ZINC09110467	-40.9309	-43.2474
ZINC08384546	-45.1140	-43.2468
ZINC09353935	-44.4438	-43.2450
ZINC04066409	-39.5012	-43.2436
ZINC17835778	-43.9532	-43.2432

ZINC17835778	-42.6111	-43.2432
ZINC17835778	-42.3901	-43.2432
ZINC13127857	-43.6374	-43.2431
ZINC06161959	-40.5864	-43.2400
ZINC06161959	-38.3402	-43.2400
ZINC08996143	-42.1528	-43.2358
ZINC04065812	-39.8805	-43.2347
ZINC04065812	-39.5362	-43.2347
ZINC04065812	-39.3344	-43.2347
ZINC08996636	-41.6548	-43.2342
ZINC01414784	-45.4192	-43.2334
ZINC01414784	-43.1555	-43.2334
ZINC08413221	-43.4446	-43.2327
ZINC08413221	-41.7826	-43.2327
ZINC08743913	-47.3129	-43.2318
ZINC00663514	-41.7091	-43.2298
ZINC13553032	-44.4940	-43.2298
ZINC13553032	-43.0038	-43.2298
ZINC13553032	-42.5717	-43.2298
ZINC00678023	-45.1875	-43.2288
ZINC04416017	-37.5169	-43.2283
ZINC04416017	-36.7725	-43.2283
ZINC00626750	-46.7161	-43.2279
ZINC00626750	-45.5236	-43.2279
ZINC08440043	-41.1378	-43.2276
ZINC08440043	-39.5596	-43.2276
ZINC08440043	-38.8256	-43.2276
ZINC06162418	-41.1284	-43.2273
ZINC06162418	-39.7582	-43.2273
ZINC57263083	-41.1087	-43.2262
ZINC06162308	-37.2927	-43.2261
ZINC06148804	-37.4990	-43.2259
ZINC06148586	-42.5145	-43.2224
ZINC08438737	-39.2213	-43.2224
ZINC06442936	-39.0110	-43.2222
ZINC09354097	-40.1060	-43.2216
ZINC15989939	-40.5941	-43.2206
ZINC15989939	-39.7935	-43.2206
ZINC15989939	-38.1421	-43.2206
ZINC08445128	-44.1598	-43.2171
ZINC08445128	-44.1567	-43.2171
ZINC04068003	-40.5234	-43.2141
ZINC08426813	-39.3709	-43.2128
ZINC04391378	-42.3183	-43.2122
ZINC04391378	-41.0394	-43.2122
ZINC08440872	-39.3309	-43.2108
ZINC08440872	-37.4384	-43.2108
ZINC08441145	-45.3356	-43.2104
ZINC08440045	-40.7798	-43.2101
ZINC08440045	-40.6471	-43.2101
ZINC08440045	-40.0948	-43.2101
ZINC08715656	-42.7861	-43.2097
ZINC06444654	-40.9021	-43.2093
ZINC09436571	-39.6508	-43.2090

ZINC05807081	-37.0020	-43.2067
ZINC06137156	-39.9278	-43.2047
ZINC06137156	-39.2809	-43.2047
ZINC06137156	-38.6413	-43.2047
ZINC13126534	-37.6148	-43.2026
ZINC08450307	-38.9346	-43.2025
ZINC00708159	-38.3697	-43.2024
ZINC08413411	-41.4485	-43.2012
ZINC08413411	-40.0004	-43.2012
ZINC00823696	-38.8644	-43.2002
ZINC00823696	-37.7323	-43.2002
ZINC00823696	-37.5288	-43.2002
ZINC85425772	-38.5257	-43.2000
ZINC57263068	-38.5699	-43.1978
ZINC19911936	-41.7809	-43.1963
ZINC19911936	-38.5554	-43.1963
ZINC00848137	-36.9999	-43.1947
ZINC12406087	-38.9952	-43.1939
ZINC04066172	-41.9037	-43.1932
ZINC04066172	-40.4525	-43.1932
ZINC08439757	-44.7623	-43.1902
ZINC06242043	-40.5921	-43.1899
ZINC00821070	-38.1035	-43.1857
ZINC00821070	-37.8118	-43.1857
ZINC02201761	-41.2260	-43.1849
ZINC20874162	-42.1211	-43.1849
ZINC08400260	-41.0596	-43.1838
ZINC08400260	-37.9406	-43.1838
ZINC00726638	-39.0420	-43.1833
ZINC00726638	-38.0584	-43.1833
ZINC00726638	-37.4381	-43.1833
ZINC08854614	-40.6372	-43.1831
ZINC19830678	-43.0794	-43.1816
ZINC04417620	-41.8737	-43.1802
ZINC04066597	-40.8883	-43.1791
ZINC04066597	-40.6479	-43.1791
ZINC08416355	-37.5563	-43.1772
ZINC20171822	-50.8625	-43.1759
ZINC20171822	-50.4294	-43.1759
ZINC13388465	-39.6302	-43.1731
ZINC13570888	-40.3992	-43.1719
ZINC08440116	-39.0855	-43.1711
ZINC08413504	-36.4423	-43.1684
ZINC08386721	-45.8130	-43.1639
ZINC06197518	-36.3391	-43.1635
ZINC04067610	-39.6903	-43.1596
ZINC04067280	-43.0433	-43.1570
ZINC04067280	-42.3343	-43.1570
ZINC04067280	-41.4382	-43.1570
ZINC04629668	-43.9714	-43.1530
ZINC04629668	-41.9598	-43.1530
ZINC06195980	-42.5085	-43.1526
ZINC08427645	-39.3512	-43.1511
ZINC20533627	-43.6894	-43.1500

ZINC20533627	-43.1118	-43.1500
ZINC20533627	-41.6018	-43.1500
ZINC08433355	-43.3124	-43.1498
ZINC08433355	-42.8307	-43.1498
ZINC08433355	-42.1300	-43.1498
ZINC57356047	-37.7770	-43.1492
ZINC08424618	-39.3528	-43.1486
ZINC01413497	-46.8434	-43.1484
ZINC01413497	-45.5582	-43.1484
ZINC00707962	-40.5148	-43.1456
ZINC22589643	-42.5156	-43.1443
ZINC00726570	-39.7517	-43.1424
ZINC00726570	-38.9982	-43.1424
ZINC00726570	-37.3474	-43.1424
ZINC00708332	-39.1776	-43.1409
ZINC00703045	-43.3852	-43.1408
ZINC00703045	-42.1004	-43.1408
ZINC19200307	-42.5140	-43.1393
ZINC12757672	-45.6106	-43.1360
ZINC12757672	-43.6280	-43.1360
ZINC08384616	-46.8222	-43.1346
ZINC04067623	-38.7861	-43.1341
ZINC04067623	-37.3579	-43.1341
ZINC01413495	-46.9627	-43.1326
ZINC01413495	-43.7755	-43.1326
ZINC15188285	-37.9125	-43.1302
ZINC04258313	-40.9694	-43.1285
ZINC04719059	-40.3358	-43.1259
ZINC18154614	-41.0314	-43.1251
ZINC09110317	-38.1370	-43.1241
ZINC08827782	-41.9203	-43.1239
ZINC08439841	-39.5492	-43.1216
ZINC00792938	-39.4330	-43.1214
ZINC00792938	-38.0308	-43.1214
ZINC00792938	-37.9532	-43.1214
ZINC08743943	-47.0127	-43.1201
ZINC04060757	-38.0278	-43.1182
ZINC04060757	-36.8095	-43.1182
ZINC23477246	-39.7963	-43.1172
ZINC23477246	-39.2403	-43.1172
ZINC04626408	-39.4065	-43.1154
ZINC01019728	-44.8624	-43.1141
ZINC08396937	-41.8868	-43.1140
ZINC06015595	-37.7443	-43.1125
ZINC19801732	-36.2947	-43.1112
ZINC18179947	-43.1142	-43.1106
ZINC00673821	-40.7335	-43.1097
ZINC08740789	-42.4299	-43.1047
ZINC04419250	-41.9938	-43.1038
ZINC13958094	-40.9165	-43.1024
ZINC13958094	-39.8153	-43.1024
ZINC13958094	-38.6480	-43.1024
ZINC05225798	-40.6464	-43.1022
ZINC04417967	-38.3616	-43.0998

ZINC06196081	-41.5155	-43.0983
ZINC00707957	-40.3514	-43.0963
ZINC00707957	-39.5659	-43.0963
ZINC08903365	-39.8626	-43.0954
ZINC00703106	-40.9331	-43.0936
ZINC00703106	-39.3742	-43.0936
ZINC06162085	-39.4362	-43.0928
ZINC19366940	-37.9778	-43.0907
ZINC22574398	-40.7461	-43.0903
ZINC09413628	-42.8393	-43.0899
ZINC09413628	-40.2250	-43.0899
ZINC02191190	-37.2440	-43.0899
ZINC19781854	-38.4964	-43.0880
ZINC19781854	-37.1338	-43.0880
ZINC17167364	-41.5429	-43.0843
ZINC01414770	-45.3557	-43.0842
ZINC01414770	-44.6670	-43.0842
ZINC04112063	-40.2683	-43.0839
ZINC04112063	-37.5494	-43.0839
ZINC09235090	-42.0881	-43.0815
ZINC01414765	-43.3344	-43.0809
ZINC09243050	-45.2946	-43.0803
ZINC00674594	-42.5113	-43.0799
ZINC08400656	-43.7360	-43.0794
ZINC08400656	-41.2510	-43.0794
ZINC08400656	-41.0779	-43.0794
ZINC08827584	-42.6148	-43.0793
ZINC04066581	-38.9400	-43.0791
ZINC08442172	-39.6132	-43.0788
ZINC08442172	-38.1927	-43.0788
ZINC08426812	-41.9581	-43.0769
ZINC08426812	-40.6628	-43.0769
ZINC08426812	-40.4608	-43.0769
ZINC04062429	-37.0772	-43.0764
ZINC06162253	-39.0223	-43.0752
ZINC08413426	-38.7577	-43.0750
ZINC08413426	-37.1210	-43.0750
ZINC09272036	-39.8866	-43.0744
ZINC09272036	-38.5523	-43.0744
ZINC00703140	-42.6741	-43.0740
ZINC19369714	-38.4944	-43.0728
ZINC00726677	-41.0333	-43.0721
ZINC00726677	-40.2345	-43.0721
ZINC04067509	-40.1141	-43.0714
ZINC04067509	-39.8770	-43.0714
ZINC04067509	-38.9439	-43.0714
ZINC08439620	-39.6636	-43.0711
ZINC08439620	-36.2881	-43.0711
ZINC13569396	-41.4886	-43.0698
ZINC13569396	-40.5189	-43.0698
ZINC27346175	-37.8769	-43.0675
ZINC04395669	-37.5504	-43.0666
ZINC06194175	-36.3802	-43.0658
ZINC09087643	-42.0999	-43.0654

ZINC08440770	-43.5339	-43.0650
ZINC00827958	-42.7460	-43.0639
ZINC06195780	-39.4179	-43.0631
ZINC05408806	-38.0050	-43.0628
ZINC08416242	-41.2044	-43.0602
ZINC08416242	-40.7073	-43.0602
ZINC01776001	-36.8418	-43.0602
ZINC08441040	-39.1909	-43.0593
ZINC08441040	-37.2049	-43.0593
ZINC13388401	-39.3438	-43.0591
ZINC08441294	-39.5117	-43.0566
ZINC08441294	-38.0779	-43.0566
ZINC08441294	-37.8079	-43.0566
ZINC19901650	-44.2018	-43.0555
ZINC08430624	-43.9490	-43.0528
ZINC08430624	-43.2420	-43.0528
ZINC08430624	-42.4177	-43.0528
ZINC00647311	-42.5806	-43.0525
ZINC19636339	-45.1646	-43.0511
ZINC19923525	-37.7258	-43.0503
ZINC06197087	-38.4713	-43.0488
ZINC02071920	-48.4952	-43.0488
ZINC09413653	-39.9200	-43.0475
ZINC08415623	-39.5096	-43.0469
ZINC04674290	-37.4762	-43.0401
ZINC04048226	-43.0515	-43.0387
ZINC04048226	-43.0323	-43.0387
ZINC04048226	-41.6400	-43.0387
ZINC05246618	-39.6850	-43.0341
ZINC19802566	-42.6850	-43.0340
ZINC19802566	-39.1222	-43.0340
ZINC00826315	-37.6025	-43.0335
ZINC00707977	-39.2695	-43.0322
ZINC00707977	-38.6852	-43.0322
ZINC00707977	-37.5574	-43.0322
ZINC17969459	-40.1950	-43.0271
ZINC17969459	-37.7927	-43.0271
ZINC08440190	-41.2673	-43.0251
ZINC08440190	-38.0865	-43.0251
ZINC08440190	-37.2805	-43.0251
ZINC08441401	-39.3032	-43.0245
ZINC08426796	-40.2408	-43.0239
ZINC08426796	-37.8878	-43.0239
ZINC08426796	-36.9427	-43.0239
ZINC19324072	-38.7628	-43.0235
ZINC17145559	-39.7839	-43.0196
ZINC05894986	-39.1855	-43.0159
ZINC08429961	-41.5469	-43.0150
ZINC08429961	-40.8496	-43.0150
ZINC09354270	-45.2297	-43.0132
ZINC09354270	-43.3072	-43.0132
ZINC09354270	-39.5318	-43.0132
ZINC08440479	-37.9406	-43.0118
ZINC19832323	-46.1154	-43.0114

ZINC06195800	-38.7750	-43.0098
ZINC04067607	-38.7057	-43.0074
ZINC06996093	-38.5693	-43.0066
ZINC00726491	-40.3410	-43.0062
ZINC00726491	-36.4655	-43.0062
ZINC04439337	-37.5424	-43.0058
ZINC00702711	-41.4904	-43.0022
ZINC08438691	-39.2163	-42.9971
ZINC00628981	-40.7184	-42.9963
ZINC00628981	-40.0928	-42.9963
ZINC22171321	-40.8218	-42.9957
ZINC00707955	-38.8698	-42.9951
ZINC00707955	-38.0459	-42.9951
ZINC00707955	-37.9115	-42.9951
ZINC00716605	-44.6377	-42.9947
ZINC08440686	-39.6913	-42.9936
ZINC08440686	-39.0458	-42.9936
ZINC05220344	-40.7167	-42.9914
ZINC05220344	-38.5444	-42.9914
ZINC00853715	-38.9349	-42.9890
ZINC00853715	-38.8145	-42.9890
ZINC85406431	-38.0852	-42.9889
ZINC08439847	-37.5439	-42.9883
ZINC08439847	-36.4616	-42.9883
ZINC06499093	-44.1660	-42.9866
ZINC19922748	-44.5596	-42.9865
ZINC00703084	-44.1483	-42.9861
ZINC00703084	-43.9469	-42.9861
ZINC18116853	-39.5756	-42.9860
ZINC06715112	-38.9673	-42.9855
ZINC19871631	-40.9240	-42.9808
ZINC06162382	-39.4670	-42.9734
ZINC06162382	-36.5587	-42.9734
ZINC04068047	-40.3992	-42.9734
ZINC04019557	-40.2079	-42.9728
ZINC04019557	-39.3145	-42.9728
ZINC85406295	-37.5426	-42.9726
ZINC04059959	-37.6922	-42.9695
ZINC08425427	-40.3778	-42.9686
ZINC08425427	-36.8031	-42.9686
ZINC08425427	-36.5724	-42.9686
ZINC08433277	-41.9616	-42.9663
ZINC00861574	-39.8623	-42.9642
ZINC00702925	-43.2300	-42.9639
ZINC13470617	-39.4956	-42.9622
ZINC04061882	-40.5852	-42.9619
ZINC17117953	-42.6321	-42.9605
ZINC19897471	-43.5662	-42.9600
ZINC13388520	-39.2611	-42.9578
ZINC00357898	-37.7820	-42.9535
ZINC04068125	-38.2423	-42.9532
ZINC08384308	-44.9500	-42.9531
ZINC08426054	-36.7289	-42.9524
ZINC08441300	-41.6729	-42.9521

ZINC08441300	-40.4798	-42.9521
ZINC08744148	-41.9204	-42.9512
ZINC08744148	-41.0270	-42.9512
ZINC06137161	-43.4118	-42.9508
ZINC06137161	-42.2219	-42.9508
ZINC13634070	-41.7941	-42.9487
ZINC13634070	-39.4909	-42.9487
ZINC04068044	-38.4909	-42.9486
ZINC08396503	-44.8193	-42.9480
ZINC08396503	-43.2570	-42.9480
ZINC08396503	-38.5364	-42.9480
ZINC08897821	-43.0311	-42.9438
ZINC08897821	-42.9530	-42.9438
ZINC08897821	-42.3450	-42.9438
ZINC08715692	-42.5187	-42.9431
ZINC08715692	-42.2306	-42.9431
ZINC36646180	-43.2765	-42.9425
ZINC33335662	-41.9463	-42.9423
ZINC00703016	-45.5195	-42.9414
ZINC06015303	-37.3485	-42.9410
ZINC06015303	-37.2835	-42.9410
ZINC00703110	-41.4332	-42.9399
ZINC01019485	-40.1426	-42.9393
ZINC08386824	-46.5709	-42.9376
ZINC09312700	-40.5755	-42.9373
ZINC09312700	-40.3301	-42.9373
ZINC04060009	-37.7979	-42.9352
ZINC02157862	-46.0961	-42.9331
ZINC19781737	-37.1906	-42.9326
ZINC04067608	-38.5037	-42.9300
ZINC00702985	-43.0500	-42.9292
ZINC09270978	-46.5269	-42.9276
ZINC09270978	-44.2281	-42.9276
ZINC09270978	-44.0708	-42.9276
ZINC09270978	-42.4695	-42.9276
ZINC09270978	-42.0210	-42.9276
ZINC08439489	-44.4000	-42.9248
ZINC08439489	-37.8294	-42.9248
ZINC05490082	-37.9794	-42.9233
ZINC05807046	-41.0885	-42.9232
ZINC05807046	-37.7387	-42.9232
ZINC08383626	-41.8386	-42.9232
ZINC00674025	-42.4581	-42.9203
ZINC04068104	-39.2927	-42.9195
ZINC04068104	-37.4820	-42.9195
ZINC00721236	-40.5540	-42.9174
ZINC00721236	-40.3258	-42.9174
ZINC19926613	-36.5813	-42.9174
ZINC04066548	-39.8741	-42.9161
ZINC19797020	-40.5004	-42.9147
ZINC04780876	-39.4660	-42.9129
ZINC04067673	-40.5488	-42.9123
ZINC04067673	-38.8856	-42.9123
ZINC04067673	-37.6618	-42.9123

ZINC06162406	-40.3703	-42.9075
ZINC02192886	-46.6894	-42.9061
ZINC09271294	-41.2911	-42.9061
ZINC15952858	-45.4011	-42.9042
ZINC02069956	-45.3561	-42.9035
ZINC00726576	-39.7169	-42.9032
ZINC00726576	-38.7864	-42.9032
ZINC00726576	-37.6107	-42.9032
ZINC08411613	-40.5303	-42.9024
ZINC08411613	-39.2071	-42.9024
ZINC08400612	-43.2799	-42.9018
ZINC04067034	-45.1021	-42.9008
ZINC04067034	-44.6890	-42.9008
ZINC04067034	-44.4279	-42.9008
ZINC19852641	-46.5019	-42.8997
ZINC06196088	-42.4098	-42.8983
ZINC22797782	-41.4410	-42.8979
ZINC17750770	-40.5803	-42.8947
ZINC17750770	-39.4308	-42.8947
ZINC04065657	-40.9167	-42.8939
ZINC04065657	-40.9075	-42.8939
ZINC04065657	-40.8052	-42.8939
ZINC00364806	-37.4038	-42.8928
ZINC04113086	-44.3651	-42.8927
ZINC04113086	-43.3184	-42.8927
ZINC04113086	-42.5025	-42.8927
ZINC06197397	-41.2993	-42.8915
ZINC06197397	-40.9592	-42.8915
ZINC04417625	-39.2209	-42.8914
ZINC06700979	-41.9908	-42.8913
ZINC00702994	-42.4666	-42.8907
ZINC08441210	-43.0333	-42.8902
ZINC08817654	-46.4575	-42.8900
ZINC02890976	-41.1291	-42.8884
ZINC05806859	-36.8143	-42.8878
ZINC13569394	-39.5202	-42.8835
ZINC05807235	-40.3671	-42.8827
ZINC05807235	-38.6711	-42.8827
ZINC09272790	-40.9028	-42.8826
ZINC09272790	-40.1113	-42.8826
ZINC06442930	-38.3760	-42.8822
ZINC02055866	-46.7651	-42.8814
ZINC04019556	-39.5063	-42.8801
ZINC04019556	-38.5207	-42.8801
ZINC09244009	-39.8858	-42.8791
ZINC09244009	-39.5392	-42.8791
ZINC06668084	-43.7278	-42.8752
ZINC06668084	-43.5948	-42.8752
ZINC08441984	-42.0063	-42.8743
ZINC06732134	-47.0745	-42.8736
ZINC08430713	-40.1343	-42.8734
ZINC08430713	-38.1908	-42.8734
ZINC08430713	-37.6434	-42.8734
ZINC09271670	-40.9775	-42.8720

ZINC05118783	-37.0709	-42.8719
ZINC08450349	-38.7793	-42.8677
ZINC08450349	-38.6687	-42.8677
ZINC22075535	-39.3350	-42.8664
ZINC08425431	-37.7679	-42.8657
ZINC09014491	-41.1270	-42.8640
ZINC08430195	-40.8858	-42.8632
ZINC00823435	-39.3311	-42.8631
ZINC00823435	-38.5580	-42.8631
ZINC00823435	-37.5240	-42.8631
ZINC19802676	-42.5401	-42.8612
ZINC19367908	-36.6846	-42.8591
ZINC13388420	-40.4951	-42.8577
ZINC08413524	-37.8352	-42.8576
ZINC00844261	-42.6864	-42.8559
ZINC00844261	-42.4312	-42.8559
ZINC08416056	-39.7147	-42.8538
ZINC00727074	-43.5105	-42.8521
ZINC04044196	-40.8871	-42.8501
ZINC04066701	-38.4393	-42.8483
ZINC68712924	-43.3207	-42.8461
ZINC20190809	-46.6030	-42.8456
ZINC85381127	-44.0898	-42.8456
ZINC18085129	-42.8257	-42.8433
ZINC18085129	-41.7258	-42.8433
ZINC18085129	-41.6210	-42.8433
ZINC18085129	-41.5739	-42.8433
ZINC13570646	-43.7998	-42.8415
ZINC13570646	-42.6400	-42.8415
ZINC08448591	-40.5760	-42.8342
ZINC08448591	-39.8832	-42.8342
ZINC19324521	-39.3652	-42.8341
ZINC04067616	-40.6825	-42.8332
ZINC08442206	-41.0183	-42.8306
ZINC12507975	-48.6932	-42.8288
ZINC09360155	-42.0842	-42.8260
ZINC09360155	-37.7240	-42.8260
ZINC08453845	-44.4228	-42.8260
ZINC08453845	-43.3547	-42.8260
ZINC08453845	-41.7848	-42.8260
ZINC04068083	-36.6887	-42.8256
ZINC06195431	-41.1008	-42.8255
ZINC06195431	-41.0892	-42.8255
ZINC20134987	-44.7254	-42.8216
ZINC08444552	-40.6974	-42.8211
ZINC04123604	-40.7837	-42.8194
ZINC04123604	-40.2534	-42.8194
ZINC04123604	-39.0407	-42.8194
ZINC02181872	-38.1760	-42.8170
ZINC08440556	-44.4372	-42.8157
ZINC08440556	-42.6509	-42.8157
ZINC06162411	-40.9900	-42.8151
ZINC06136969	-37.6243	-42.8142
ZINC08413608	-38.9220	-42.8132

ZINC08413608	-38.2315	-42.8132
ZINC08413608	-37.7162	-42.8132
ZINC09453208	-43.7114	-42.8119
ZINC09453208	-43.3905	-42.8119
ZINC08425564	-41.0264	-42.8111
ZINC08425564	-40.8126	-42.8111
ZINC08425564	-39.9167	-42.8111
ZINC04065157	-38.9169	-42.8107
ZINC04065157	-38.1295	-42.8107
ZINC20233174	-46.7784	-42.8102
ZINC20036549	-41.8915	-42.8064
ZINC20036549	-38.0761	-42.8064
ZINC00674039	-40.6950	-42.8052
ZINC08442188	-40.7545	-42.8040
ZINC08441705	-38.9779	-42.8033
ZINC08384364	-44.6140	-42.7989
ZINC12404462	-38.9998	-42.7975
ZINC08437280	-40.4594	-42.7970
ZINC08438534	-44.7665	-42.7967
ZINC00714612	-40.0149	-42.7941
ZINC00714612	-38.9410	-42.7941
ZINC00714612	-38.1391	-42.7941
ZINC04067570	-38.0935	-42.7919
ZINC00730060	-40.4940	-42.7903
ZINC09186729	-39.6057	-42.7893
ZINC06197103	-40.2329	-42.7892
ZINC15321019	-42.3047	-42.7890
ZINC02135504	-41.7403	-42.7851
ZINC13424055	-42.7558	-42.7847
ZINC05408796	-42.8050	-42.7846
ZINC05408796	-42.2294	-42.7846
ZINC05408796	-42.1950	-42.7846
ZINC00719484	-42.9984	-42.7839
ZINC00702554	-44.2029	-42.7818
ZINC00702554	-41.8500	-42.7818
ZINC06442990	-38.2389	-42.7811
ZINC00726653	-40.7414	-42.7804
ZINC00726653	-37.5225	-42.7804
ZINC00726653	-37.5217	-42.7804
ZINC33293687	-42.0923	-42.7794
ZINC05919169	-40.1419	-42.7794
ZINC08425424	-39.1400	-42.7787
ZINC08425424	-38.1360	-42.7787
ZINC09009037	-40.2756	-42.7783
ZINC09009037	-36.7621	-42.7783
ZINC09088383	-40.3765	-42.7779
ZINC09088383	-39.2310	-42.7779
ZINC00800745	-37.4731	-42.7773
ZINC20573681	-42.3158	-42.7761
ZINC20573681	-39.6235	-42.7761
ZINC20573681	-36.8215	-42.7761
ZINC06149857	-39.5222	-42.7760
ZINC04681640	-40.1153	-42.7742
ZINC04681640	-39.8291	-42.7742

ZINC06444655	-40.6840	-42.7736
ZINC06444655	-40.5076	-42.7736
ZINC06241713	-42.0994	-42.7734
ZINC06444409	-39.3022	-42.7718
ZINC06444409	-38.2317	-42.7718
ZINC17028292	-43.9833	-42.7682
ZINC08415609	-43.0404	-42.7680
ZINC08415609	-38.9617	-42.7680
ZINC00641757	-37.7674	-42.7668
ZINC08440553	-42.4703	-42.7665
ZINC08440553	-40.8931	-42.7665
ZINC05806942	-42.1605	-42.7655
ZINC05806942	-40.9740	-42.7655
ZINC04067439	-38.9361	-42.7603
ZINC04067439	-37.1967	-42.7603
ZINC19938340	-45.8860	-42.7599
ZINC13509961	-41.9742	-42.7596
ZINC08439749	-44.7521	-42.7584
ZINC08439749	-44.3723	-42.7584
ZINC08439749	-44.3183	-42.7584
ZINC05800394	-37.3759	-42.7583
ZINC08440060	-42.6055	-42.7583
ZINC08440060	-42.2387	-42.7583
ZINC08440060	-39.9272	-42.7583
ZINC06148619	-41.5030	-42.7547
ZINC06148619	-41.4233	-42.7547
ZINC06148619	-41.1892	-42.7547
ZINC08983960	-47.4311	-42.7525
ZINC08983960	-44.6062	-42.7525
ZINC08983960	-41.5470	-42.7525
ZINC08434983	-39.3596	-42.7503
ZINC32609480	-37.4651	-42.7501
ZINC06015534	-39.1671	-42.7493
ZINC00826268	-40.8881	-42.7479
ZINC00826268	-39.2505	-42.7479
ZINC04671279	-38.7489	-42.7478
ZINC18043207	-40.3964	-42.7476
ZINC06197505	-42.0465	-42.7462
ZINC06197505	-38.1184	-42.7462
ZINC19209001	-41.1459	-42.7453
ZINC08455008	-43.4683	-42.7452
ZINC08429895	-42.9310	-42.7447
ZINC08429895	-41.6740	-42.7447
ZINC06015495	-38.7862	-42.7442
ZINC08439616	-38.7742	-42.7432
ZINC08439616	-37.2954	-42.7432
ZINC08439616	-36.4133	-42.7432
ZINC15015695	-40.3275	-42.7428
ZINC06783384	-42.2644	-42.7421
ZINC19814703	-36.5816	-42.7393
ZINC06148735	-41.2562	-42.7382
ZINC06148735	-38.2452	-42.7382
ZINC08440573	-44.8587	-42.7379
ZINC00866536	-39.0342	-42.7352

ZINC00866536	-38.2261	-42.7352
ZINC00866536	-37.3759	-42.7352
ZINC08442422	-38.2422	-42.7344
ZINC08442422	-36.8244	-42.7344
ZINC04494355	-37.7020	-42.7335
ZINC00702440	-43.9317	-42.7329
ZINC00702440	-43.3549	-42.7329
ZINC02105616	-37.5521	-42.7327
ZINC08439409	-40.0838	-42.7311
ZINC08439409	-39.3753	-42.7311
ZINC04066101	-40.3506	-42.7280
ZINC01413487	-46.3732	-42.7278
ZINC01413487	-44.9160	-42.7278
ZINC06783974	-41.1461	-42.7270
ZINC06783974	-39.1766	-42.7270
ZINC13569109	-49.7806	-42.7266
ZINC04067349	-40.5889	-42.7229
ZINC04067349	-40.1247	-42.7229
ZINC00681649	-40.5496	-42.7208
ZINC09507782	-40.0601	-42.7199
ZINC09507782	-39.5245	-42.7199
ZINC04061032	-38.9675	-42.7197
ZINC04061032	-38.1160	-42.7197
ZINC04061032	-37.1891	-42.7197
ZINC08441731	-40.8255	-42.7169
ZINC19532278	-40.0791	-42.7152
ZINC00721228	-40.0724	-42.7149
ZINC00721228	-39.8869	-42.7149
ZINC08439483	-41.4059	-42.7124
ZINC08439483	-39.4943	-42.7124
ZINC08439483	-39.2838	-42.7124
ZINC05408889	-40.0007	-42.7089
ZINC05408889	-39.3379	-42.7089
ZINC19832117	-36.4374	-42.7063
ZINC02055385	-42.2645	-42.7063
ZINC00679183	-41.7255	-42.7009
ZINC00679183	-41.6081	-42.7009
ZINC00625874	-38.5395	-42.7005
ZINC08413419	-41.7483	-42.6998
ZINC04068320	-39.6718	-42.6998
ZINC08413419	-38.0140	-42.6998
ZINC04068320	-36.3978	-42.6998
ZINC04417646	-40.9517	-42.6989
ZINC04417646	-37.5004	-42.6989
ZINC13569556	-41.9287	-42.6972
ZINC06149639	-41.0125	-42.6938
ZINC00702701	-40.4547	-42.6931
ZINC00702701	-40.0672	-42.6931
ZINC17196730	-42.9868	-42.6931
ZINC17196730	-41.2591	-42.6931
ZINC22937073	-42.9633	-42.6926
ZINC18143659	-43.0336	-42.6923
ZINC08439863	-41.7831	-42.6899
ZINC08396801	-40.4403	-42.6899

ZINC08396801	-39.9005	-42.6899
ZINC08396801	-39.7299	-42.6899
ZINC08439863	-39.6925	-42.6899
ZINC12468409	-46.4516	-42.6890
ZINC12468409	-42.3991	-42.6890
ZINC13387848	-40.8830	-42.6876
ZINC00853701	-40.1409	-42.6873
ZINC00853701	-39.5522	-42.6873
ZINC00853701	-38.9351	-42.6873
ZINC08462813	-42.1697	-42.6854
ZINC02075472	-39.6753	-42.6832
ZINC04062434	-37.9982	-42.6815
ZINC08386822	-43.7171	-42.6808
ZINC09312128	-45.6945	-42.6807
ZINC04066050	-39.7153	-42.6803
ZINC04066050	-39.1512	-42.6803
ZINC06162040	-40.9658	-42.6792
ZINC04067495	-38.6479	-42.6762
ZINC04067495	-38.2935	-42.6762
ZINC04067495	-37.5983	-42.6762
ZINC02055867	-44.5077	-42.6749
ZINC57411588	-41.5292	-42.6720
ZINC19892163	-42.2731	-42.6697
ZINC00726648	-39.4346	-42.6688
ZINC00726648	-37.6562	-42.6688
ZINC17166794	-38.8400	-42.6685
ZINC17166794	-38.7426	-42.6685
ZINC38246111	-41.6926	-42.6683
ZINC18189067	-39.1508	-42.6678
ZINC16115870	-44.1964	-42.6667
ZINC16115870	-43.6960	-42.6667
ZINC16115870	-42.2041	-42.6667
ZINC06195514	-40.4234	-42.6661
ZINC01035618	-42.9764	-42.6657
ZINC04019916	-43.3948	-42.6654
ZINC04019916	-43.3453	-42.6654
ZINC04019916	-41.8949	-42.6654
ZINC06015434	-41.7329	-42.6654
ZINC08996853	-48.9578	-42.6637
ZINC08996853	-46.6336	-42.6637
ZINC00674243	-39.2294	-42.6585
ZINC09089201	-47.5953	-42.6584
ZINC09089201	-45.7706	-42.6584
ZINC09089201	-44.4034	-42.6584
ZINC08430629	-46.0475	-42.6579
ZINC08430629	-44.8948	-42.6579
ZINC08430629	-43.6886	-42.6579
ZINC06162329	-38.4037	-42.6576
ZINC08854618	-38.2030	-42.6571
ZINC06136850	-40.2764	-42.6569
ZINC08986693	-47.4229	-42.6560
ZINC08986693	-46.2517	-42.6560
ZINC02136501	-45.6587	-42.6522
ZINC00850073	-42.3000	-42.6504

ZINC08444501	-43.1094	-42.6478
ZINC06137362	-40.3904	-42.6463
ZINC06137362	-37.8905	-42.6463
ZINC06137362	-36.6960	-42.6463
ZINC04568526	-40.2718	-42.6454
ZINC08414930	-37.7036	-42.6413
ZINC08440555	-37.6319	-42.6405
ZINC00675026	-40.3409	-42.6381
ZINC09455274	-42.4789	-42.6363
ZINC19873373	-37.1712	-42.6356
ZINC00820671	-37.8378	-42.6350
ZINC06196077	-40.5363	-42.6340
ZINC00866538	-39.5380	-42.6309
ZINC00866538	-36.8638	-42.6309
ZINC01019952	-42.2700	-42.6301
ZINC01019952	-40.8208	-42.6301
ZINC00726550	-39.4573	-42.6301
ZINC00726550	-39.0162	-42.6301
ZINC03902117	-40.5266	-42.6295
ZINC03902117	-40.2958	-42.6295
ZINC03902117	-39.9365	-42.6295
ZINC08440606	-41.9553	-42.6292
ZINC08440606	-39.5742	-42.6292
ZINC06195974	-41.1110	-42.6287
ZINC08441958	-41.7382	-42.6283
ZINC08439748	-41.1449	-42.6282
ZINC08439748	-39.5733	-42.6282
ZINC08439748	-38.3366	-42.6282
ZINC06197645	-40.5513	-42.6281
ZINC06149698	-42.4153	-42.6272
ZINC08440787	-41.1972	-42.6270
ZINC08411606	-39.2708	-42.6260
ZINC08411606	-38.1219	-42.6260
ZINC08867425	-41.4719	-42.6244
ZINC00848995	-41.8644	-42.6242
ZINC17166883	-44.8953	-42.6231
ZINC08827361	-44.3743	-42.6221
ZINC08827361	-42.8477	-42.6221
ZINC08827361	-42.6118	-42.6221
ZINC06786265	-39.3128	-42.6218
ZINC08407686	-42.5405	-42.6167
ZINC08440121	-39.4423	-42.6148
ZINC08440121	-38.7511	-42.6148
ZINC06162256	-40.2896	-42.6134
ZINC00626094	-39.1392	-42.6112
ZINC08413430	-40.3740	-42.6100
ZINC05578287	-40.6465	-42.6090
ZINC05578287	-39.1265	-42.6090
ZINC06267233	-39.3168	-42.6081
ZINC08425327	-38.2246	-42.6075
ZINC08425327	-38.0842	-42.6075
ZINC08425327	-37.1362	-42.6075
ZINC04060511	-38.9557	-42.6057
ZINC04102452	-40.4042	-42.6049

ZINC04102452	-39.2004	-42.6049
ZINC04632282	-37.9677	-42.6020
ZINC06137100	-39.3072	-42.6015
ZINC17166824	-43.4831	-42.5973
ZINC17166824	-41.3250	-42.5973
ZINC08453804	-43.2488	-42.5970
ZINC19904103	-41.3349	-42.5937
ZINC00691630	-41.1139	-42.5933
ZINC19878119	-36.5143	-42.5933
ZINC09236155	-45.4256	-42.5927
ZINC04067916	-40.6561	-42.5894
ZINC06149871	-42.6763	-42.5887
ZINC02340381	-39.2811	-42.5881
ZINC08450710	-39.1982	-42.5873
ZINC08441470	-47.1173	-42.5847
ZINC08441470	-46.3535	-42.5847
ZINC18087975	-41.7491	-42.5834
ZINC06148692	-45.1214	-42.5832
ZINC08384256	-44.4640	-42.5827
ZINC08904984	-40.6306	-42.5818
ZINC08904984	-39.7311	-42.5818
ZINC19938423	-47.8397	-42.5817
ZINC00728223	-37.0401	-42.5807
ZINC06442952	-39.6044	-42.5805
ZINC04065815	-40.9592	-42.5793
ZINC04065815	-40.9314	-42.5793
ZINC04067032	-45.1028	-42.5793
ZINC04067032	-43.2750	-42.5793
ZINC04067032	-40.5694	-42.5793
ZINC00707975	-39.6539	-42.5771
ZINC00707975	-39.6313	-42.5771
ZINC00707975	-39.2971	-42.5771
ZINC04453198	-36.8004	-42.5753
ZINC00726606	-40.4458	-42.5739
ZINC00726606	-40.1211	-42.5739
ZINC04059881	-39.9311	-42.5739
ZINC04062289	-36.5302	-42.5730
ZINC08416344	-43.9128	-42.5726
ZINC04065922	-38.0689	-42.5699
ZINC04065922	-37.7309	-42.5699
ZINC00844257	-42.0058	-42.5696
ZINC00844257	-41.4809	-42.5696
ZINC00844257	-40.9299	-42.5696
ZINC08440378	-40.4020	-42.5692
ZINC04094624	-39.7604	-42.5678
ZINC04094624	-36.5226	-42.5678
ZINC08430770	-40.2470	-42.5675
ZINC08430770	-40.2402	-42.5675
ZINC08430770	-40.0274	-42.5675
ZINC19831059	-38.1020	-42.5662
ZINC18176432	-42.6555	-42.5661
ZINC09312723	-39.1649	-42.5661
ZINC09312723	-38.3574	-42.5661
ZINC00666657	-37.6575	-42.5652

ZINC08440778	-39.7942	-42.5652
ZINC06238877	-39.2607	-42.5651
ZINC06238877	-38.8099	-42.5651
ZINC08439759	-44.7835	-42.5649
ZINC06197495	-38.9412	-42.5647
ZINC06137620	-41.9432	-42.5631
ZINC06194361	-39.5230	-42.5631
ZINC00675332	-40.2173	-42.5605
ZINC04066485	-38.5155	-42.5598
ZINC04066485	-37.9718	-42.5598
ZINC06720081	-38.0254	-42.5593
ZINC08829005	-39.8451	-42.5590
ZINC22937031	-38.9874	-42.5570
ZINC19802169	-42.4526	-42.5569
ZINC19802169	-40.2422	-42.5569
ZINC19832130	-36.8726	-42.5558
ZINC08398845	-48.9963	-42.5538
ZINC19369110	-37.4025	-42.5524
ZINC09042799	-45.3570	-42.5511
ZINC13230703	-48.8420	-42.5508
ZINC00625910	-42.1294	-42.5496
ZINC00726548	-39.9283	-42.5483
ZINC00726548	-39.7381	-42.5483
ZINC00726548	-39.2914	-42.5483
ZINC22075719	-43.5829	-42.5474
ZINC00845287	-38.7845	-42.5463
ZINC08426288	-40.7447	-42.5454
ZINC19790777	-38.9441	-42.5436
ZINC04780859	-40.6585	-42.5422
ZINC04780859	-39.7207	-42.5422
ZINC20036553	-42.3706	-42.5416
ZINC04626487	-37.6282	-42.5390
ZINC19732754	-37.8039	-42.5383
ZINC00853824	-40.0026	-42.5361
ZINC00853824	-39.0007	-42.5361
ZINC00853824	-38.4627	-42.5361
ZINC18223126	-38.6981	-42.5350
ZINC18223126	-38.2962	-42.5350
ZINC08434861	-44.3580	-42.5346
ZINC04114994	-40.0425	-42.5342
ZINC04114994	-39.4600	-42.5342
ZINC15880041	-41.1694	-42.5328
ZINC08439285	-40.2949	-42.5313
ZINC08439285	-36.8192	-42.5313
ZINC06195467	-44.9165	-42.5312
ZINC02093286	-37.6588	-42.5306
ZINC08744016	-47.4090	-42.5298
ZINC08744016	-47.3043	-42.5298
ZINC19872033	-38.4143	-42.5297
ZINC19889694	-37.2788	-42.5297
ZINC05226011	-40.9446	-42.5233
ZINC05226011	-38.1470	-42.5233
ZINC00708233	-39.4220	-42.5223
ZINC06194088	-38.6558	-42.5188

ZINC06194088	-36.7046	-42.5188
ZINC00703369	-44.0607	-42.5188
ZINC00703369	-42.3863	-42.5188
ZINC09088193	-38.6777	-42.5181
ZINC00714851	-37.7447	-42.5172
ZINC09065433	-38.6426	-42.5170
ZINC09065433	-38.1639	-42.5170
ZINC05093458	-44.1606	-42.5168
ZINC17920874	-37.4401	-42.5126
ZINC22859275	-38.6076	-42.5124
ZINC18178922	-39.4333	-42.5103
ZINC19827245	-39.2277	-42.5076
ZINC19827245	-38.6057	-42.5076
ZINC00702697	-40.8910	-42.5049
ZINC06015625	-37.9316	-42.5048
ZINC05895006	-38.2484	-42.5047
ZINC04066419	-39.7694	-42.5044
ZINC04066419	-38.6693	-42.5044
ZINC08434868	-41.5458	-42.5031
ZINC08416181	-38.8594	-42.5026
ZINC06050171	-37.7475	-42.5016
ZINC06050171	-37.0521	-42.5016
ZINC19882150	-43.0713	-42.5013
ZINC00710232	-37.6285	-42.5009
ZINC06194144	-39.0641	-42.4997
ZINC02088138	-40.0361	-42.4965
ZINC00726590	-38.8011	-42.4960
ZINC04062147	-39.3130	-42.4956
ZINC04019666	-41.9543	-42.4945
ZINC08408184	-38.4636	-42.4927
ZINC04067752	-39.5223	-42.4911
ZINC08440608	-44.2937	-42.4910
ZINC08440608	-43.8848	-42.4910
ZINC04112061	-38.8592	-42.4903
ZINC04112061	-38.8514	-42.4903
ZINC20171824	-45.6086	-42.4896
ZINC08440815	-40.5016	-42.4856
ZINC19890007	-39.9653	-42.4847
ZINC18191608	-38.0791	-42.4816
ZINC04780888	-37.3928	-42.4800
ZINC13231930	-45.4779	-42.4790
ZINC04017080	-39.2758	-42.4770
ZINC04017080	-38.3071	-42.4770
ZINC04017080	-38.2149	-42.4770
ZINC01480038	-42.6237	-42.4770
ZINC01480038	-42.1455	-42.4770
ZINC00647197	-41.5359	-42.4761
ZINC08441841	-36.7897	-42.4754
ZINC05729441	-50.6695	-42.4709
ZINC08430082	-40.9758	-42.4707
ZINC08837244	-45.2602	-42.4703
ZINC08837244	-44.8406	-42.4703
ZINC18062885	-43.3504	-42.4698
ZINC00991573	-41.8247	-42.4691

ZINC00717197	-38.6331	-42.4672
ZINC04066540	-40.8323	-42.4665
ZINC00726294	-39.0503	-42.4664
ZINC00726294	-36.9647	-42.4664
ZINC08439805	-39.0876	-42.4657
ZINC08439805	-38.7093	-42.4657
ZINC08439805	-37.8276	-42.4657
ZINC19797023	-41.4266	-42.4613
ZINC19889479	-39.7969	-42.4612
ZINC05800510	-39.1104	-42.4596
ZINC00727825	-37.7432	-42.4581
ZINC00727825	-36.5193	-42.4581
ZINC00626271	-41.6526	-42.4572
ZINC06444267	-41.7137	-42.4569
ZINC06444267	-38.2745	-42.4569
ZINC08439704	-38.0975	-42.4558
ZINC19360237	-36.4501	-42.4556
ZINC19360237	-36.3672	-42.4556
ZINC04114898	-40.2271	-42.4553
ZINC04632294	-43.2216	-42.4535
ZINC04632294	-41.7592	-42.4535
ZINC04068050	-39.1614	-42.4527
ZINC04068050	-36.8413	-42.4527
ZINC08433395	-44.4136	-42.4511
ZINC00690411	-39.2788	-42.4442
ZINC00726640	-39.5629	-42.4432
ZINC00726640	-38.3639	-42.4432
ZINC03876750	-39.4879	-42.4409
ZINC00995684	-50.9519	-42.4393
ZINC19938349	-45.7832	-42.4390
ZINC06197417	-37.8083	-42.4342
ZINC08427629	-41.2315	-42.4339
ZINC08427629	-38.7793	-42.4339
ZINC08438686	-40.1023	-42.4339
ZINC08438686	-39.5747	-42.4339
ZINC13880266	-37.5426	-42.4332
ZINC04629634	-42.3285	-42.4318
ZINC04629634	-41.3933	-42.4318
ZINC19885512	-47.9322	-42.4292
ZINC04626425	-38.0249	-42.4280
ZINC01019935	-41.1754	-42.4266
ZINC01019935	-39.9492	-42.4266
ZINC00339907	-39.5976	-42.4256
ZINC00339907	-38.7839	-42.4256
ZINC04416014	-38.1835	-42.4249
ZINC04416014	-37.7945	-42.4249
ZINC04416014	-37.0556	-42.4249
ZINC06196072	-37.9769	-42.4236
ZINC17043303	-42.0529	-42.4221
ZINC09110383	-39.0702	-42.4191
ZINC05027694	-39.3356	-42.4186
ZINC05027694	-37.1633	-42.4186
ZINC19894480	-44.5932	-42.4178
ZINC08437411	-37.5361	-42.4175

ZINC00717199	-38.6597	-42.4170
ZINC08416169	-39.3673	-42.4164
ZINC08416169	-38.4130	-42.4164
ZINC06137277	-38.4461	-42.4149
ZINC04065949	-42.1391	-42.4147
ZINC04065949	-40.0134	-42.4147
ZINC00884699	-42.1462	-42.4145
ZINC00714849	-37.3597	-42.4143
ZINC02088184	-38.7071	-42.4099
ZINC00659260	-41.2865	-42.4088
ZINC05905868	-38.1901	-42.4086
ZINC08739431	-48.2964	-42.4081
ZINC09160476	-40.8981	-42.4057
ZINC09160476	-36.9989	-42.4057
ZINC06196438	-37.1019	-42.4049
ZINC19872140	-36.5550	-42.4031
ZINC05490600	-39.5076	-42.4027
ZINC19872054	-38.3690	-42.4022
ZINC19872224	-42.2156	-42.4021
ZINC08717078	-39.8552	-42.4017
ZINC08717078	-38.8095	-42.4017
ZINC06015435	-37.1400	-42.3999
ZINC00729266	-37.5714	-42.3974
ZINC08440200	-43.3729	-42.3962
ZINC08440200	-41.8304	-42.3962
ZINC09425263	-44.4747	-42.3952
ZINC08416315	-42.9207	-42.3934
ZINC08425423	-39.8686	-42.3933
ZINC08425423	-39.1544	-42.3933
ZINC08425423	-38.9474	-42.3933
ZINC19943625	-44.6873	-42.3928
ZINC08414924	-39.4021	-42.3918
ZINC06194521	-41.7801	-42.3912
ZINC00726489	-39.0789	-42.3902
ZINC00726489	-37.2800	-42.3902
ZINC08439433	-42.2314	-42.3898
ZINC08439433	-42.1277	-42.3898
ZINC08439433	-41.2135	-42.3898
ZINC06196221	-38.8760	-42.3898
ZINC02082123	-41.2209	-42.3892
ZINC17112562	-40.8314	-42.3891
ZINC19691217	-37.6033	-42.3849
ZINC04067480	-38.8788	-42.3841
ZINC04067480	-36.5018	-42.3841
ZINC04067480	-36.4219	-42.3841
ZINC09041238	-39.5787	-42.3824
ZINC12419638	-41.4826	-42.3784
ZINC04059723	-37.2549	-42.3775
ZINC04059723	-36.4711	-42.3775
ZINC36646056	-42.9207	-42.3772
ZINC17180710	-43.2626	-42.3768
ZINC17180710	-39.5561	-42.3768
ZINC06444302	-40.6228	-42.3729
ZINC06444302	-40.3763	-42.3729

ZINC19872096	-42.9039	-42.3721
ZINC02766984	-37.0076	-42.3701
ZINC04019575	-41.9808	-42.3692
ZINC04019575	-38.7112	-42.3692
ZINC04061018	-37.0524	-42.3674
ZINC00709147	-39.9806	-42.3667
ZINC06197168	-39.6405	-42.3661
ZINC01413468	-41.8009	-42.3656
ZINC01413468	-41.5869	-42.3656
ZINC19938294	-47.2109	-42.3652
ZINC06468813	-38.2315	-42.3639
ZINC08384290	-44.5929	-42.3635
ZINC05010283	-39.3207	-42.3616
ZINC05010283	-38.6829	-42.3616
ZINC05764799	-37.0465	-42.3612
ZINC08439823	-37.1239	-42.3610
ZINC37868335	-44.6884	-42.3609
ZINC08442175	-41.8495	-42.3584
ZINC08397404	-41.4647	-42.3581
ZINC08397404	-41.3013	-42.3581
ZINC13532874	-38.2760	-42.3573
ZINC17167289	-37.4959	-42.3569
ZINC00625733	-37.8065	-42.3551
ZINC00625733	-37.2673	-42.3551
ZINC04067976	-43.5044	-42.3543
ZINC04067976	-42.1699	-42.3543
ZINC01148725	-39.5461	-42.3532
ZINC09487350	-43.7319	-42.3532
ZINC09487350	-42.1673	-42.3532
ZINC08413888	-41.7118	-42.3530
ZINC08413888	-38.5747	-42.3530
ZINC08413888	-36.5184	-42.3530
ZINC23468460	-38.0355	-42.3522
ZINC00673870	-40.4357	-42.3503
ZINC00673870	-39.9397	-42.3503
ZINC00691615	-39.5434	-42.3498
ZINC09353155	-45.0113	-42.3488
ZINC09353155	-44.5105	-42.3488
ZINC09353155	-44.1484	-42.3488
ZINC09353155	-43.6700	-42.3488
ZINC08425610	-40.7932	-42.3487
ZINC08413066	-39.2367	-42.3485
ZINC08413066	-36.6504	-42.3485
ZINC20031238	-37.9771	-42.3483
ZINC06162388	-39.6355	-42.3474
ZINC06162388	-39.1490	-42.3474
ZINC06445928	-42.6256	-42.3470
ZINC06445928	-40.6056	-42.3470
ZINC11639552	-39.0337	-42.3467
ZINC19923549	-39.8812	-42.3451
ZINC08439343	-39.7546	-42.3450
ZINC08442036	-42.9680	-42.3449
ZINC06144549	-43.4072	-42.3423
ZINC00726508	-36.7076	-42.3423

ZINC04067062	-42.1720	-42.3418
ZINC04067062	-40.2003	-42.3418
ZINC00674915	-39.3780	-42.3408
ZINC18250800	-45.0170	-42.3407
ZINC18250800	-44.7706	-42.3407
ZINC18250800	-44.6172	-42.3407
ZINC18250800	-42.6748	-42.3407
ZINC08396815	-37.4705	-42.3406
ZINC08396815	-37.2633	-42.3406
ZINC02332324	-43.0085	-42.3400
ZINC00884694	-42.4138	-42.3397
ZINC00999459	-43.3109	-42.3391
ZINC13388461	-39.4337	-42.3389
ZINC04666905	-37.4731	-42.3376
ZINC01413500	-42.2040	-42.3373
ZINC06194196	-39.6051	-42.3369
ZINC08441359	-38.6252	-42.3364
ZINC02160016	-36.6339	-42.3357
ZINC85413268	-40.5414	-42.3350
ZINC08415663	-38.7756	-42.3326
ZINC02183628	-38.1936	-42.3318
ZINC04068045	-37.6068	-42.3284
ZINC01019888	-38.0481	-42.3281
ZINC04167558	-42.4177	-42.3276
ZINC04167558	-41.6989	-42.3276
ZINC06162322	-40.5933	-42.3253
ZINC06162322	-37.2997	-42.3253
ZINC09411262	-41.7236	-42.3246
ZINC08430498	-37.2059	-42.3244
ZINC08396291	-43.1790	-42.3240
ZINC19551589	-38.8776	-42.3223
ZINC17163311	-45.9924	-42.3221
ZINC08415356	-42.0972	-42.3215
ZINC08415356	-40.5009	-42.3215
ZINC00702725	-39.6593	-42.3201
ZINC04626473	-38.8883	-42.3178
ZINC13115849	-42.5455	-42.3148
ZINC08440488	-44.1725	-42.3146
ZINC09311862	-40.9732	-42.3129
ZINC05806950	-39.3626	-42.3123
ZINC05806950	-37.2560	-42.3123
ZINC19366917	-39.2812	-42.3102
ZINC19938651	-44.9540	-42.3082
ZINC06194194	-36.3469	-42.3081
ZINC07344193	-40.6415	-42.3074
ZINC07344193	-39.9632	-42.3074
ZINC05918861	-42.5331	-42.3074
ZINC08413305	-38.6513	-42.3046
ZINC00626162	-43.1708	-42.3015
ZINC08416162	-38.3765	-42.3006
ZINC19901103	-38.0928	-42.3004
ZINC06196095	-38.0443	-42.2934
ZINC13127029	-39.3825	-42.2898
ZINC06197186	-41.7532	-42.2853

ZINC06197186	-41.7118	-42.2853
ZINC00645849	-45.6851	-42.2846
ZINC08440726	-39.9499	-42.2845
ZINC08440726	-39.5885	-42.2845
ZINC08440726	-38.7677	-42.2845
ZINC02144567	-36.9533	-42.2840
ZINC06785940	-39.6791	-42.2827
ZINC08438536	-42.4944	-42.2825
ZINC04067494	-38.7908	-42.2825
ZINC04067494	-38.6010	-42.2825
ZINC04067494	-38.2035	-42.2825
ZINC02759585	-42.0565	-42.2823
ZINC09088717	-37.9617	-42.2808
ZINC09088717	-37.3896	-42.2808
ZINC04649845	-39.0403	-42.2804
ZINC01221289	-41.0808	-42.2752
ZINC00714613	-37.8255	-42.2748
ZINC00714613	-37.5471	-42.2748
ZINC02065561	-39.1187	-42.2742
ZINC00625745	-37.3798	-42.2736
ZINC00625745	-36.9636	-42.2736
ZINC09241555	-41.2539	-42.2728
ZINC09241555	-39.7500	-42.2728
ZINC09241555	-39.1401	-42.2728
ZINC08400611	-41.1334	-42.2693
ZINC08400611	-41.0811	-42.2693
ZINC08780045	-39.8612	-42.2684
ZINC19595545	-39.0782	-42.2678
ZINC00647316	-42.2742	-42.2665
ZINC06160851	-37.1086	-42.2621
ZINC04199801	-39.9509	-42.2604
ZINC19923532	-39.6978	-42.2596
ZINC02478365	-41.3528	-42.2580
ZINC02478365	-41.1542	-42.2580
ZINC20232417	-45.0632	-42.2571
ZINC18144241	-40.0622	-42.2568
ZINC00726634	-38.4546	-42.2530
ZINC02951124	-37.4840	-42.2526
ZINC03635587	-44.5583	-42.2518
ZINC04066305	-40.0583	-42.2513
ZINC00721232	-39.3382	-42.2500
ZINC00721232	-38.3639	-42.2500
ZINC08435041	-41.7797	-42.2487
ZINC08925952	-39.7974	-42.2472
ZINC04066256	-38.8791	-42.2465
ZINC04066256	-37.8912	-42.2465
ZINC18144051	-41.6967	-42.2464
ZINC04062672	-37.5561	-42.2459
ZINC08425318	-40.7312	-42.2427
ZINC00629528	-37.8675	-42.2422
ZINC04065927	-38.5826	-42.2370
ZINC04065927	-36.6641	-42.2370
ZINC17888704	-43.3743	-42.2368
ZINC17888704	-41.9202	-42.2368

ZINC18212576	-42.4965	-42.2364
ZINC06149855	-38.9147	-42.2358
ZINC13601781	-39.2458	-42.2352
ZINC13601781	-38.0116	-42.2352
ZINC08433252	-43.9499	-42.2346
ZINC08433252	-43.0237	-42.2346
ZINC08415537	-41.4062	-42.2312
ZINC06148579	-41.6854	-42.2288
ZINC09424273	-45.5131	-42.2287
ZINC31937220	-42.7131	-42.2282
ZINC08738685	-45.8668	-42.2281
ZINC08738685	-44.5682	-42.2281
ZINC08738685	-44.4501	-42.2281
ZINC08738685	-42.1702	-42.2281
ZINC33345173	-42.5677	-42.2268
ZINC06996091	-38.1487	-42.2257
ZINC00142239	-38.4298	-42.2251
ZINC06444374	-41.6888	-42.2240
ZINC00625949	-36.2615	-42.2233
ZINC04067867	-41.5895	-42.2230
ZINC04067867	-41.3653	-42.2230
ZINC06148681	-41.7602	-42.2221
ZINC08413989	-39.5604	-42.2214
ZINC08413989	-36.5350	-42.2214
ZINC08439281	-40.7365	-42.2212
ZINC08439281	-40.6083	-42.2212
ZINC09272135	-40.0756	-42.2212
ZINC04391381	-41.3996	-42.2209
ZINC02462260	-42.7998	-42.2204
ZINC04067813	-38.2111	-42.2203
ZINC04065581	-39.1531	-42.2197
ZINC04065581	-38.8059	-42.2197
ZINC04065581	-38.1978	-42.2197
ZINC04065856	-42.2056	-42.2181
ZINC04065856	-41.4889	-42.2181
ZINC33333558	-39.2071	-42.2150
ZINC05646855	-41.3341	-42.2146
ZINC05646855	-39.8879	-42.2146
ZINC05646855	-37.9049	-42.2146
ZINC05646855	-37.8344	-42.2146
ZINC06499092	-45.2672	-42.2138
ZINC20228412	-38.6025	-42.2127
ZINC08441911	-42.1353	-42.2114
ZINC08439394	-39.9509	-42.2107
ZINC00708236	-37.6760	-42.2101
ZINC00708236	-36.9767	-42.2101
ZINC06195737	-42.9858	-42.2100
ZINC06195737	-38.2232	-42.2100
ZINC12844992	-41.3480	-42.2034
ZINC08450348	-40.6085	-42.2034
ZINC19872029	-38.0815	-42.2016
ZINC17166834	-39.2403	-42.2003
ZINC06162488	-36.6838	-42.1995
ZINC19871526	-41.6382	-42.1985

ZINC02473365	-40.2528	-42.1981
ZINC02473365	-39.5501	-42.1981
ZINC09424308	-43.2904	-42.1973
ZINC04067342	-41.3897	-42.1961
ZINC04067342	-40.3741	-42.1961
ZINC02478229	-38.3687	-42.1941
ZINC02478229	-37.9290	-42.1941
ZINC02478229	-37.2975	-42.1941
ZINC19801792	-36.4684	-42.1933
ZINC09414344	-40.3972	-42.1916
ZINC08439431	-42.1259	-42.1913
ZINC08439431	-41.1698	-42.1913
ZINC08439431	-37.4217	-42.1913
ZINC00844248	-40.2510	-42.1895
ZINC00844248	-39.7579	-42.1895
ZINC04114835	-41.0080	-42.1893
ZINC08426366	-41.7006	-42.1885
ZINC08426366	-41.5515	-42.1885
ZINC09354154	-39.8858	-42.1884
ZINC09350140	-39.6597	-42.1883
ZINC09350140	-39.3628	-42.1883
ZINC04780872	-40.8975	-42.1847
ZINC00726532	-37.9080	-42.1842
ZINC13387890	-40.8761	-42.1839
ZINC06197384	-37.8436	-42.1823
ZINC17193920	-37.8364	-42.1779
ZINC17193920	-37.1365	-42.1779
ZINC08440081	-40.9775	-42.1779
ZINC08440761	-39.2651	-42.1774
ZINC08440761	-36.3355	-42.1774
ZINC00847632	-44.1583	-42.1747
ZINC00847632	-43.4939	-42.1747
ZINC08415412	-38.4709	-42.1723
ZINC04067778	-38.0455	-42.1698
ZINC04632293	-41.3284	-42.1694
ZINC08987719	-39.1414	-42.1662
ZINC08431064	-43.3234	-42.1661
ZINC04067511	-40.7659	-42.1661
ZINC04067511	-40.6894	-42.1661
ZINC04067511	-39.9707	-42.1661
ZINC08415880	-37.5095	-42.1657
ZINC08415880	-36.7266	-42.1657
ZINC06149862	-39.4116	-42.1655
ZINC06786081	-39.5830	-42.1648
ZINC06786081	-39.1485	-42.1648
ZINC00349369	-37.7533	-42.1641
ZINC00728574	-40.6833	-42.1629
ZINC01820707	-41.3559	-42.1625
ZINC04067906	-37.4097	-42.1617
ZINC04062302	-38.1228	-42.1607
ZINC04062302	-37.6229	-42.1607
ZINC08437189	-42.3186	-42.1601
ZINC08437189	-41.2403	-42.1601
ZINC06195586	-41.8211	-42.1596

ZINC06195586	-37.0646	-42.1596
ZINC02080815	-41.9129	-42.1588
ZINC09046769	-42.3550	-42.1576
ZINC04060855	-39.8849	-42.1569
ZINC04060855	-37.2931	-42.1569
ZINC04112074	-38.9874	-42.1559
ZINC04112074	-38.6476	-42.1559
ZINC03833726	-38.8739	-42.1551
ZINC06144554	-43.4848	-42.1550
ZINC06137197	-37.8247	-42.1547
ZINC08996637	-39.8082	-42.1546
ZINC00726605	-39.4306	-42.1538
ZINC00726605	-39.3754	-42.1538
ZINC17161167	-41.2947	-42.1538
ZINC17161167	-40.9820	-42.1538
ZINC32609490	-39.4374	-42.1453
ZINC08440124	-39.2788	-42.1441
ZINC08440124	-37.7364	-42.1441
ZINC09046515	-43.4238	-42.1435
ZINC09046515	-42.3210	-42.1435
ZINC06148666	-41.8437	-42.1412
ZINC06162369	-37.7485	-42.1402
ZINC09471275	-45.0693	-42.1400
ZINC09471275	-44.1508	-42.1400
ZINC09373744	-41.8251	-42.1400
ZINC04067609	-37.8105	-42.1393
ZINC36646058	-42.2866	-42.1374
ZINC08440738	-39.4953	-42.1360
ZINC08440738	-39.0140	-42.1360
ZINC08440738	-37.7406	-42.1360
ZINC02265397	-39.5180	-42.1359
ZINC03309148	-47.1536	-42.1341
ZINC09270705	-40.5005	-42.1309
ZINC01414752	-45.5549	-42.1291
ZINC01414752	-42.6248	-42.1291
ZINC04673796	-42.0909	-42.1290
ZINC36055686	-41.4388	-42.1288
ZINC04114961	-39.2114	-42.1277
ZINC04114961	-39.0232	-42.1277
ZINC03905784	-39.4231	-42.1252
ZINC03905784	-37.8721	-42.1252
ZINC08893723	-41.7334	-42.1248
ZINC10313189	-44.1579	-42.1229
ZINC08414835	-43.7605	-42.1224
ZINC08414835	-41.7864	-42.1224
ZINC04391413	-39.2354	-42.1210
ZINC06194518	-40.2140	-42.1175
ZINC19550249	-39.5420	-42.1163
ZINC02862061	-39.5269	-42.1152
ZINC03665951	-48.6816	-42.1147
ZINC17532961	-39.9980	-42.1128
ZINC19849361	-40.9486	-42.1124
ZINC05444277	-44.5573	-42.1113
ZINC09110925	-39.9457	-42.1094

ZINC08441678	-38.3447	-42.1091
ZINC08441678	-36.3001	-42.1091
ZINC04199812	-38.3215	-42.1080
ZINC19901310	-39.6823	-42.1074
ZINC08384367	-42.9114	-42.1067
ZINC09357144	-42.2310	-42.1056
ZINC09357144	-41.1094	-42.1056
ZINC00866534	-38.4027	-42.1056
ZINC00866534	-38.2452	-42.1056
ZINC00866534	-36.9888	-42.1056
ZINC08384695	-42.4269	-42.1033
ZINC08413335	-38.6169	-42.1014
ZINC08413335	-37.3741	-42.1014
ZINC04066480	-37.7385	-42.1013
ZINC04066480	-37.5309	-42.1013
ZINC04048237	-40.8124	-42.0986
ZINC04048237	-40.4333	-42.0986
ZINC04048237	-39.4557	-42.0986
ZINC06137355	-38.3204	-42.0982
ZINC06137355	-37.3144	-42.0982
ZINC17166832	-40.1281	-42.0980
ZINC17166832	-38.7641	-42.0980
ZINC08396755	-44.2820	-42.0978
ZINC08425316	-38.2624	-42.0978
ZINC17180660	-39.6050	-42.0976
ZINC09243615	-42.3840	-42.0973
ZINC09243615	-39.3205	-42.0973
ZINC00195057	-38.5129	-42.0948
ZINC00195057	-38.1493	-42.0948
ZINC13945117	-40.6666	-42.0946
ZINC32541285	-38.1984	-42.0938
ZINC06149881	-39.7262	-42.0919
ZINC05220332	-38.3486	-42.0914
ZINC05220332	-37.7981	-42.0914
ZINC04719165	-37.0410	-42.0912
ZINC00647542	-36.7658	-42.0898
ZINC06442984	-38.5316	-42.0834
ZINC08455852	-41.9322	-42.0825
ZINC00675027	-40.8976	-42.0825
ZINC04067588	-36.5943	-42.0812
ZINC04112167	-38.2225	-42.0801
ZINC04112167	-38.1141	-42.0801
ZINC04112167	-37.5653	-42.0801
ZINC19924427	-37.0421	-42.0787
ZINC04112203	-37.2277	-42.0766
ZINC10313227	-44.3719	-42.0757
ZINC13388257	-41.7152	-42.0752
ZINC13388257	-41.5359	-42.0752
ZINC06995623	-42.6558	-42.0751
ZINC09436613	-40.0344	-42.0725
ZINC19938468	-47.7349	-42.0719
ZINC13283112	-38.1647	-42.0708
ZINC00666662	-36.4889	-42.0706
ZINC13189497	-39.5849	-42.0697

ZINC04066259	-41.4050	-42.0665
ZINC02757921	-45.9579	-42.0657
ZINC00679184	-40.0969	-42.0652
ZINC00679184	-38.3999	-42.0652
ZINC06195942	-41.9231	-42.0645
ZINC05226006	-40.0608	-42.0585
ZINC05226006	-38.4651	-42.0585
ZINC00180996	-39.6947	-42.0584
ZINC09009886	-39.4557	-42.0535
ZINC09009886	-39.2932	-42.0535
ZINC09340002	-48.1854	-42.0533
ZINC09340002	-46.9681	-42.0533
ZINC09340002	-45.8074	-42.0533
ZINC08384602	-43.9960	-42.0507
ZINC00986265	-37.5346	-42.0500
ZINC00671377	-46.3456	-42.0494
ZINC02062693	-38.2187	-42.0484
ZINC06195983	-39.8650	-42.0461
ZINC06088805	-49.1639	-42.0435
ZINC09324694	-39.7427	-42.0426
ZINC04067822	-38.5237	-42.0415
ZINC17251522	-45.6232	-42.0405
ZINC19210607	-39.4573	-42.0398
ZINC04067564	-37.2757	-42.0378
ZINC09186730	-40.0559	-42.0373
ZINC01824545	-41.3570	-42.0371
ZINC08894847	-43.5283	-42.0325
ZINC08894847	-42.9392	-42.0325
ZINC13512954	-44.1949	-42.0322
ZINC06196512	-38.3256	-42.0319
ZINC19872461	-44.2541	-42.0309
ZINC04681717	-40.1302	-42.0294
ZINC00343275	-36.2673	-42.0293
ZINC09334403	-39.6050	-42.0286
ZINC09280207	-40.7842	-42.0286
ZINC09358326	-41.4359	-42.0286
ZINC00884701	-41.7235	-42.0279
ZINC05883674	-38.9445	-42.0276
ZINC05883674	-38.8426	-42.0276
ZINC57264337	-39.6394	-42.0276
ZINC06197140	-39.1159	-42.0253
ZINC08438769	-45.2429	-42.0231
ZINC08438769	-44.0305	-42.0231
ZINC08442497	-42.7286	-42.0226
ZINC06197420	-38.3179	-42.0217
ZINC06197420	-36.9234	-42.0217
ZINC08411517	-38.2844	-42.0208
ZINC08384313	-42.7979	-42.0207
ZINC18106210	-37.8745	-42.0197
ZINC18106210	-37.1976	-42.0197
ZINC49590249	-41.2178	-42.0173
ZINC18163890	-39.6916	-42.0155
ZINC08397389	-43.9989	-42.0143
ZINC08437710	-38.6316	-42.0132

ZINC00653460	-44.3193	-42.0131
ZINC04728516	-40.3828	-42.0087
ZINC04728516	-39.7516	-42.0087
ZINC06015621	-39.0774	-42.0078
ZINC09068788	-43.6920	-42.0073
ZINC09068788	-42.6916	-42.0073
ZINC09068788	-41.0017	-42.0073
ZINC09275488	-40.7648	-42.0069
ZINC02137069	-45.8283	-42.0065
ZINC04854517	-36.9054	-42.0064
ZINC09412211	-42.2756	-42.0060
ZINC08438662	-43.3283	-42.0029
ZINC08438662	-42.3391	-42.0029
ZINC09357146	-39.5011	-42.0016
ZINC09357146	-39.2770	-42.0016
ZINC08426349	-44.2449	-42.0009
ZINC08384774	-40.1785	-42.0005
ZINC09045307	-40.1944	-42.0004
ZINC00645718	-43.8569	-42.0004
ZINC05444161	-44.8145	-41.9999
ZINC04065822	-38.9706	-41.9998
ZINC04065822	-37.7482	-41.9998
ZINC09043436	-43.4874	-41.9990
ZINC03903141	-40.1071	-41.9974
ZINC03903141	-38.4727	-41.9974
ZINC08854615	-38.1872	-41.9954
ZINC08416147	-38.8690	-41.9940
ZINC06162008	-38.7782	-41.9938
ZINC15015773	-37.1280	-41.9933
ZINC00853774	-36.7356	-41.9925
ZINC06783927	-37.5241	-41.9900
ZINC17130908	-41.7979	-41.9899
ZINC06195687	-41.0123	-41.9891
ZINC09669265	-38.5199	-41.9890
ZINC57353340	-37.5762	-41.9884
ZINC04112201	-38.1304	-41.9881
ZINC04112201	-37.0999	-41.9881
ZINC19853567	-41.8027	-41.9867
ZINC09271671	-39.1145	-41.9847
ZINC04019669	-47.3750	-41.9841
ZINC04019669	-47.3021	-41.9841
ZINC04019669	-46.8275	-41.9841
ZINC13756332	-45.8197	-41.9837
ZINC13756332	-45.1019	-41.9837
ZINC19909096	-38.6198	-41.9824
ZINC19909096	-36.9174	-41.9824
ZINC71415909	-40.3970	-41.9823
ZINC75274205	-41.2477	-41.9816
ZINC08396823	-36.6205	-41.9813
ZINC08383806	-43.6299	-41.9790
ZINC19796807	-42.2194	-41.9788
ZINC19796807	-40.5098	-41.9788
ZINC00711910	-40.4529	-41.9777
ZINC05408985	-40.5245	-41.9774

ZINC05408985	-38.2418	-41.9774
ZINC06196439	-40.7761	-41.9762
ZINC04780903	-39.4496	-41.9690
ZINC04780903	-37.8054	-41.9690
ZINC04062335	-39.5581	-41.9689
ZINC08440062	-37.2266	-41.9684
ZINC19369716	-37.1768	-41.9665
ZINC16115622	-40.6933	-41.9647
ZINC09186774	-39.7972	-41.9624
ZINC05718957	-43.6200	-41.9619
ZINC17166611	-39.4276	-41.9607
ZINC17166611	-38.3998	-41.9607
ZINC04658581	-42.0144	-41.9583
ZINC08425577	-37.9953	-41.9581
ZINC08425577	-37.4847	-41.9581
ZINC00708007	-40.0883	-41.9569
ZINC00708007	-37.9256	-41.9569
ZINC00708007	-36.4249	-41.9569
ZINC02090340	-36.9730	-41.9568
ZINC06136963	-39.1334	-41.9553
ZINC05785575	-42.2468	-41.9548
ZINC05785575	-42.1149	-41.9548
ZINC08400179	-36.8887	-41.9531
ZINC08439613	-37.8766	-41.9523
ZINC08439613	-36.6025	-41.9523
ZINC08386661	-41.7868	-41.9493
ZINC08386661	-41.5339	-41.9493
ZINC00625908	-44.4585	-41.9486
ZINC08408056	-39.5852	-41.9484
ZINC09242413	-37.6781	-41.9482
ZINC00726528	-38.2740	-41.9456
ZINC06162452	-37.4520	-41.9439
ZINC06442927	-36.3077	-41.9434
ZINC08384100	-36.8047	-41.9433
ZINC08435058	-39.1741	-41.9413
ZINC04666866	-42.2187	-41.9393
ZINC04066608	-42.2130	-41.9381
ZINC04066608	-40.3419	-41.9381
ZINC12437146	-37.8851	-41.9375
ZINC18284787	-40.2514	-41.9333
ZINC18284787	-37.8278	-41.9333
ZINC09012259	-39.8190	-41.9331
ZINC19894472	-40.8248	-41.9326
ZINC20414410	-40.4516	-41.9309
ZINC09357504	-43.5309	-41.9308
ZINC02105642	-36.9507	-41.9295
ZINC08439327	-40.4864	-41.9289
ZINC00703097	-40.7978	-41.9286
ZINC00703097	-37.7990	-41.9286
ZINC06162345	-38.6487	-41.9265
ZINC08922005	-44.4084	-41.9244
ZINC04112205	-37.3285	-41.9233
ZINC04629624	-41.1956	-41.9216
ZINC04629624	-38.8107	-41.9216

ZINC04649900	-37.6365	-41.9209
ZINC04660857	-44.2895	-41.9201
ZINC08426801	-39.0325	-41.9200
ZINC08426801	-37.3634	-41.9200
ZINC04067921	-39.7364	-41.9178
ZINC04067921	-39.0807	-41.9178
ZINC05220338	-37.4073	-41.9176
ZINC05220338	-37.0132	-41.9176
ZINC08415797	-39.3559	-41.9169
ZINC08415797	-36.6224	-41.9169
ZINC08441956	-41.6910	-41.9155
ZINC06162016	-39.1416	-41.9149
ZINC06162016	-38.4497	-41.9149
ZINC19908698	-42.2263	-41.9105
ZINC19908698	-42.0965	-41.9105
ZINC16115366	-37.4811	-41.9102
ZINC00703070	-41.5453	-41.9071
ZINC00703070	-41.2019	-41.9071
ZINC68712919	-44.1199	-41.9071
ZINC06161985	-37.5131	-41.9058
ZINC08996689	-42.2052	-41.9029
ZINC08996689	-39.2469	-41.9029
ZINC09460781	-39.4948	-41.9025
ZINC04780905	-41.3608	-41.9016
ZINC04780905	-39.0730	-41.9016
ZINC59860292	-40.2287	-41.9011
ZINC08715679	-38.7138	-41.9006
ZINC08715679	-37.8298	-41.9006
ZINC09312386	-41.1997	-41.8991
ZINC08430695	-37.7328	-41.8950
ZINC08430695	-36.6323	-41.8950
ZINC08413615	-41.1921	-41.8944
ZINC08397408	-40.4810	-41.8935
ZINC08397408	-37.7661	-41.8935
ZINC06162386	-38.7929	-41.8931
ZINC08408047	-40.3876	-41.8909
ZINC08440528	-42.3772	-41.8904
ZINC00712354	-40.7372	-41.8899
ZINC18023752	-37.7930	-41.8898
ZINC00626518	-45.3069	-41.8892
ZINC00626518	-42.8021	-41.8892
ZINC08439465	-40.3424	-41.8877
ZINC08439465	-39.0552	-41.8877
ZINC08439465	-38.4219	-41.8877
ZINC00675216	-42.4993	-41.8875
ZINC04059813	-38.8261	-41.8869
ZINC04067394	-40.1971	-41.8860
ZINC04067394	-40.1644	-41.8860
ZINC06743616	-38.2871	-41.8858
ZINC09354147	-41.9921	-41.8857
ZINC09354147	-40.9731	-41.8857
ZINC04417622	-37.2490	-41.8857
ZINC08383725	-43.4588	-41.8819
ZINC15724302	-38.9436	-41.8818

ZINC00781176	-38.0791	-41.8817
ZINC00781176	-37.7702	-41.8817
ZINC09363626	-38.8767	-41.8801
ZINC09363626	-36.5866	-41.8801
ZINC08817926	-39.1080	-41.8773
ZINC06159424	-40.3823	-41.8770
ZINC04066546	-37.0123	-41.8766
ZINC08438562	-38.8642	-41.8746
ZINC08438562	-37.9355	-41.8746
ZINC04061968	-37.5617	-41.8745
ZINC04061968	-37.1311	-41.8745
ZINC00647507	-43.1726	-41.8711
ZINC04066656	-40.2911	-41.8707
ZINC04066656	-40.0763	-41.8707
ZINC09339553	-40.2699	-41.8698
ZINC09313150	-42.2009	-41.8673
ZINC09313150	-41.3908	-41.8673
ZINC12467802	-43.2846	-41.8655
ZINC08441711	-37.9837	-41.8654
ZINC09358401	-41.3635	-41.8642
ZINC08444996	-39.6789	-41.8635
ZINC06148591	-40.5454	-41.8629
ZINC06136819	-39.0107	-41.8624
ZINC06136819	-37.2540	-41.8624
ZINC06194587	-39.3566	-41.8616
ZINC06194587	-36.8572	-41.8616
ZINC08416140	-39.7502	-41.8609
ZINC08450706	-38.5460	-41.8554
ZINC04067625	-40.2743	-41.8539
ZINC04067625	-40.0672	-41.8539
ZINC04067625	-39.8003	-41.8539
ZINC04398347	-37.6618	-41.8531
ZINC18179966	-40.5106	-41.8523
ZINC00674258	-40.7044	-41.8499
ZINC08437247	-43.2523	-41.8492
ZINC13552845	-42.1678	-41.8455
ZINC19332968	-37.0529	-41.8450
ZINC00632696	-40.0574	-41.8444
ZINC00679178	-39.3492	-41.8442
ZINC00679178	-39.1439	-41.8442
ZINC04675313	-40.0834	-41.8423
ZINC04675313	-37.8167	-41.8423
ZINC08430290	-37.4693	-41.8403
ZINC08430290	-36.9227	-41.8403
ZINC00703146	-40.8554	-41.8382
ZINC00870889	-40.5189	-41.8373
ZINC00631201	-42.4473	-41.8341
ZINC00726593	-38.2820	-41.8318
ZINC00726593	-37.0044	-41.8318
ZINC00726593	-36.5993	-41.8318
ZINC08439275	-42.1161	-41.8294
ZINC15952855	-48.3162	-41.8284
ZINC08435346	-49.1212	-41.8282
ZINC06196083	-40.2581	-41.8278

ZINC09328508	-40.7392	-41.8273
ZINC09328508	-40.7056	-41.8273
ZINC17413581	-42.8249	-41.8268
ZINC04312145	-37.4781	-41.8265
ZINC04312145	-36.8471	-41.8265
ZINC16115362	-43.5596	-41.8237
ZINC16115362	-43.5555	-41.8237
ZINC16115362	-43.2282	-41.8237
ZINC19872521	-36.8381	-41.8230
ZINC04066355	-39.9676	-41.8210
ZINC04066355	-39.7065	-41.8210
ZINC03899604	-37.2089	-41.8207
ZINC00993681	-41.7859	-41.8206
ZINC03218681	-49.5263	-41.8185
ZINC00717017	-38.8893	-41.8183
ZINC00717017	-36.6541	-41.8183
ZINC00717017	-36.3854	-41.8183
ZINC08383625	-44.9491	-41.8168
ZINC03902582	-42.3410	-41.8168
ZINC02769158	-37.6722	-41.8166
ZINC08425392	-38.9661	-41.8156
ZINC08425392	-38.9655	-41.8156
ZINC08425392	-38.6912	-41.8156
ZINC04066897	-39.7495	-41.8150
ZINC04066897	-39.7365	-41.8150
ZINC09065542	-40.6829	-41.8140
ZINC09065542	-39.4589	-41.8140
ZINC06195416	-42.7501	-41.8134
ZINC09455301	-44.6754	-41.8129
ZINC16115401	-41.6023	-41.8120
ZINC16115401	-41.4533	-41.8120
ZINC17166634	-42.1757	-41.8114
ZINC13424228	-41.6283	-41.8089
ZINC13424228	-37.7049	-41.8089
ZINC00679190	-39.4154	-41.8080
ZINC00679190	-39.3596	-41.8080
ZINC06442938	-41.0449	-41.8075
ZINC08415734	-38.6753	-41.8061
ZINC08444548	-38.9730	-41.8055
ZINC08416050	-40.9758	-41.8050
ZINC08416050	-38.2194	-41.8050
ZINC00865835	-40.6116	-41.7971
ZINC13914149	-44.3556	-41.7962
ZINC00968743	-36.8791	-41.7956
ZINC08384162	-41.4862	-41.7948
ZINC08384162	-37.7778	-41.7948
ZINC05490780	-36.7713	-41.7948
ZINC04068049	-37.2744	-41.7922
ZINC04068049	-36.9995	-41.7922
ZINC04068049	-36.3304	-41.7922
ZINC08433434	-40.9714	-41.7916
ZINC08433434	-40.5716	-41.7916
ZINC08433434	-39.6134	-41.7916
ZINC08438660	-41.6430	-41.7909

ZINC00631184	-46.4272	-41.7909
ZINC04060859	-40.1847	-41.7907
ZINC04060859	-39.9563	-41.7907
ZINC04060859	-39.5602	-41.7907
ZINC08440862	-37.9310	-41.7901
ZINC08440862	-37.8199	-41.7901
ZINC06480567	-46.5843	-41.7901
ZINC04626419	-37.7172	-41.7882
ZINC19938437	-46.9585	-41.7858
ZINC57122058	-44.6438	-41.7794
ZINC05918459	-40.7175	-41.7780
ZINC06015585	-38.2622	-41.7753
ZINC00673747	-39.0775	-41.7737
ZINC00702391	-42.0381	-41.7732
ZINC00702391	-41.6165	-41.7732
ZINC06446605	-40.8146	-41.7726
ZINC05093140	-42.9727	-41.7723
ZINC04312084	-44.3768	-41.7717
ZINC00626139	-39.2230	-41.7629
ZINC22076064	-36.5734	-41.7617
ZINC04112060	-39.0743	-41.7614
ZINC04112060	-38.8285	-41.7614
ZINC02872236	-38.4555	-41.7613
ZINC85429818	-37.4943	-41.7609
ZINC04043880	-39.0329	-41.7605
ZINC04043880	-37.2985	-41.7605
ZINC04043880	-36.5331	-41.7605
ZINC13127853	-40.7479	-41.7588
ZINC19797683	-42.7026	-41.7578
ZINC13387849	-39.9642	-41.7561
ZINC19923866	-52.2291	-41.7560
ZINC19922840	-40.4009	-41.7526
ZINC13174338	-40.2430	-41.7462
ZINC08415639	-39.3395	-41.7431
ZINC06137294	-40.9778	-41.7424
ZINC08439781	-39.3639	-41.7417
ZINC08439781	-38.9101	-41.7417
ZINC08387541	-38.4401	-41.7408
ZINC09210203	-43.7059	-41.7407
ZINC09210203	-42.3538	-41.7407
ZINC09210203	-41.9474	-41.7407
ZINC13003084	-41.7461	-41.7405
ZINC13003084	-41.6760	-41.7405
ZINC05408360	-41.8601	-41.7400
ZINC08413997	-39.3376	-41.7378
ZINC08413997	-37.3839	-41.7378
ZINC02182466	-37.2605	-41.7377
ZINC00726691	-39.5627	-41.7375
ZINC00726691	-39.0222	-41.7375
ZINC00726691	-38.1559	-41.7375
ZINC04065916	-38.6703	-41.7348
ZINC04065916	-38.2515	-41.7348
ZINC00691598	-41.1444	-41.7348
ZINC09070630	-44.3322	-41.7327

ZINC09070630	-43.6299	-41.7327
ZINC06148678	-42.4302	-41.7324
ZINC08430728	-40.3209	-41.7319
ZINC13511517	-48.4681	-41.7285
ZINC00853845	-39.3676	-41.7279
ZINC00853845	-38.0932	-41.7279
ZINC00853845	-37.0004	-41.7279
ZINC20530523	-44.6008	-41.7266
ZINC20530523	-40.3529	-41.7266
ZINC08383632	-45.8158	-41.7261
ZINC33430173	-36.6806	-41.7258
ZINC33430173	-36.2785	-41.7258
ZINC06241793	-39.2851	-41.7256
ZINC08439705	-37.2700	-41.7249
ZINC00895270	-39.9085	-41.7228
ZINC08411217	-42.2775	-41.7223
ZINC08903497	-41.1310	-41.7223
ZINC08441569	-41.2371	-41.7223
ZINC08441569	-39.9890	-41.7223
ZINC00674296	-39.6343	-41.7212
ZINC08740571	-39.0452	-41.7188
ZINC08441384	-37.8068	-41.7185
ZINC08439614	-38.1006	-41.7177
ZINC08439614	-37.4750	-41.7177
ZINC08439614	-36.7623	-41.7177
ZINC04067030	-42.2082	-41.7171
ZINC04067030	-42.1722	-41.7171
ZINC04067030	-40.5981	-41.7171
ZINC08439844	-40.5584	-41.7167
ZINC08439844	-37.6459	-41.7167
ZINC04067278	-43.8391	-41.7164
ZINC04067278	-43.5116	-41.7164
ZINC04067278	-42.4729	-41.7164
ZINC06110433	-42.7282	-41.7164
ZINC08435257	-42.0158	-41.7159
ZINC00349360	-37.5308	-41.7158
ZINC00349360	-37.4782	-41.7158
ZINC18059790	-38.3252	-41.7156
ZINC06238828	-38.5540	-41.7148
ZINC13284112	-38.6436	-41.7132
ZINC04060808	-38.9860	-41.7108
ZINC04060808	-38.7754	-41.7108
ZINC04060808	-37.6918	-41.7108
ZINC27824245	-39.1808	-41.7101
ZINC08444492	-42.9300	-41.7093
ZINC00703147	-42.1314	-41.7086
ZINC04626403	-37.8782	-41.7085
ZINC04285169	-38.1244	-41.7079
ZINC04285169	-36.7940	-41.7079
ZINC36066013	-40.6689	-41.7069
ZINC04060778	-38.5076	-41.7068
ZINC04060778	-38.2584	-41.7068
ZINC04060778	-37.6437	-41.7068
ZINC03901743	-39.6558	-41.7040

ZINC03901743	-39.0441	-41.7040
ZINC03901743	-38.5506	-41.7040
ZINC00702991	-41.9794	-41.6999
ZINC06197425	-39.2143	-41.6974
ZINC06197425	-38.5597	-41.6974
ZINC18163725	-41.4631	-41.6971
ZINC18163725	-41.1945	-41.6971
ZINC18163725	-40.5039	-41.6971
ZINC18163725	-39.4694	-41.6971
ZINC18163725	-38.9667	-41.6971
ZINC18163725	-38.9488	-41.6971
ZINC18163725	-38.0928	-41.6971
ZINC15978698	-38.1040	-41.6951
ZINC08413415	-39.6661	-41.6937
ZINC08413415	-37.5914	-41.6937
ZINC00632474	-41.7997	-41.6936
ZINC02270225	-40.7667	-41.6931
ZINC04060137	-37.9815	-41.6922
ZINC04060137	-37.8157	-41.6922
ZINC08383719	-43.6670	-41.6916
ZINC06015660	-39.6066	-41.6910
ZINC08438550	-41.3834	-41.6908
ZINC04285206	-40.1478	-41.6895
ZINC04285206	-39.2839	-41.6895
ZINC08426336	-41.4191	-41.6876
ZINC08744137	-38.9040	-41.6873
ZINC19853410	-39.3086	-41.6851
ZINC00717758	-37.4392	-41.6840
ZINC13571516	-39.0791	-41.6839
ZINC13571516	-37.2782	-41.6839
ZINC17193925	-37.0407	-41.6821
ZINC17193925	-36.5460	-41.6821
ZINC06148575	-40.7566	-41.6761
ZINC06148575	-37.7027	-41.6761
ZINC06148575	-37.5436	-41.6761
ZINC09350139	-39.1818	-41.6760
ZINC00702439	-41.5533	-41.6743
ZINC00702439	-39.3502	-41.6743
ZINC06137476	-38.3480	-41.6732
ZINC08743914	-46.7147	-41.6725
ZINC08743914	-44.3435	-41.6725
ZINC04817825	-39.9730	-41.6724
ZINC04817825	-39.8126	-41.6724
ZINC05817202	-38.6690	-41.6724
ZINC00674938	-36.5247	-41.6723
ZINC00726654	-37.0481	-41.6720
ZINC00726654	-36.9758	-41.6720
ZINC13569554	-38.8532	-41.6717
ZINC02184883	-42.6873	-41.6699
ZINC00707976	-37.0017	-41.6695
ZINC00707976	-36.7234	-41.6695
ZINC04417593	-41.4083	-41.6692
ZINC08397058	-37.3157	-41.6688
ZINC08838237	-41.9758	-41.6685

ZINC04905344	-39.9746	-41.6667
ZINC06196085	-40.9457	-41.6665
ZINC04067572	-37.3500	-41.6617
ZINC19797168	-40.5576	-41.6585
ZINC06162381	-41.4542	-41.6550
ZINC08400638	-40.6394	-41.6542
ZINC08415585	-39.3877	-41.6542
ZINC06241785	-37.9368	-41.6532
ZINC19909093	-39.2706	-41.6496
ZINC02157539	-40.1905	-41.6490
ZINC09374972	-40.1250	-41.6490
ZINC06015326	-42.7755	-41.6486
ZINC06015326	-39.2242	-41.6486
ZINC00709415	-37.6475	-41.6467
ZINC00709415	-37.1529	-41.6467
ZINC00709415	-36.9772	-41.6467
ZINC04067212	-46.0037	-41.6448
ZINC04780884	-38.2333	-41.6437
ZINC08413421	-40.3647	-41.6433
ZINC08413421	-36.2581	-41.6433
ZINC04065655	-38.7829	-41.6432
ZINC09088876	-42.0137	-41.6427
ZINC09088876	-38.5252	-41.6427
ZINC04075412	-37.8828	-41.6426
ZINC04075412	-37.4151	-41.6426
ZINC00625761	-40.2185	-41.6421
ZINC13424225	-40.4641	-41.6419
ZINC13424225	-40.3956	-41.6419
ZINC08430638	-37.8726	-41.6416
ZINC04568529	-41.6989	-41.6393
ZINC08440370	-40.0630	-41.6381
ZINC08440370	-38.6709	-41.6381
ZINC19797160	-39.6518	-41.6377
ZINC15837044	-43.9996	-41.6371
ZINC04148569	-37.5237	-41.6368
ZINC06420230	-38.0885	-41.6356
ZINC06420230	-37.5682	-41.6356
ZINC03995654	-38.5149	-41.6330
ZINC03995654	-36.5991	-41.6330
ZINC08897840	-45.8887	-41.6330
ZINC08897840	-44.7229	-41.6330
ZINC08897840	-44.6263	-41.6330
ZINC08897840	-43.2672	-41.6330
ZINC08897840	-41.8268	-41.6330
ZINC09329847	-41.2655	-41.6321
ZINC08416262	-39.6822	-41.6303
ZINC08416262	-38.2039	-41.6303
ZINC33351920	-40.9931	-41.6292
ZINC00673875	-39.0638	-41.6278
ZINC02184857	-47.6741	-41.6272
ZINC04068115	-44.3531	-41.6267
ZINC04068115	-43.3409	-41.6267
ZINC04068115	-43.0119	-41.6267
ZINC04067763	-41.8583	-41.6265

ZINC04067763	-41.3130	-41.6265
ZINC00184201	-36.7366	-41.6241
ZINC09007707	-41.7714	-41.6232
ZINC09007707	-40.6357	-41.6232
ZINC04114975	-39.5048	-41.6224
ZINC04114975	-38.4293	-41.6224
ZINC05484116	-38.8761	-41.6219
ZINC08441497	-40.3774	-41.6190
ZINC08441497	-39.6470	-41.6190
ZINC13470615	-43.9015	-41.6190
ZINC08740953	-36.4129	-41.6173
ZINC08671820	-40.8537	-41.6144
ZINC00823436	-39.2312	-41.6140
ZINC00823436	-38.0161	-41.6140
ZINC08437373	-37.0005	-41.6126
ZINC04258314	-37.4755	-41.6120
ZINC06786612	-39.2381	-41.6090
ZINC06786612	-37.2678	-41.6090
ZINC08439580	-38.3529	-41.6082
ZINC08439580	-38.2256	-41.6082
ZINC08439580	-36.4874	-41.6082
ZINC00643447	-37.8644	-41.6072
ZINC08439780	-39.2410	-41.6069
ZINC08439780	-37.9766	-41.6069
ZINC36040331	-44.3770	-41.6063
ZINC06442994	-40.5820	-41.6052
ZINC08426280	-37.3135	-41.6042
ZINC08426280	-37.1607	-41.6042
ZINC08994131	-44.0970	-41.6032
ZINC13174415	-38.4029	-41.6031
ZINC00821073	-39.6161	-41.6013
ZINC00821073	-39.1850	-41.6013
ZINC00821073	-38.3089	-41.6013
ZINC08441693	-39.8014	-41.6013
ZINC04065905	-37.7464	-41.5969
ZINC08442501	-44.8919	-41.5952
ZINC08442501	-44.0786	-41.5952
ZINC08442501	-42.4404	-41.5952
ZINC19909750	-39.2748	-41.5932
ZINC06194363	-39.0871	-41.5930
ZINC06194363	-38.0393	-41.5930
ZINC08415644	-40.0099	-41.5930
ZINC12468149	-40.0654	-41.5929
ZINC08413973	-38.7804	-41.5924
ZINC08440600	-44.4002	-41.5901
ZINC08440600	-44.2804	-41.5901
ZINC00674171	-40.5832	-41.5886
ZINC06136884	-38.2054	-41.5885
ZINC17166799	-39.5170	-41.5881
ZINC17166799	-38.7593	-41.5881
ZINC17166799	-37.7602	-41.5881
ZINC08426346	-44.1264	-41.5858
ZINC31904040	-39.3563	-41.5842
ZINC17138989	-39.3128	-41.5840

ZINC04062822	-37.6181	-41.5827
ZINC04062822	-37.5216	-41.5827
ZINC19815575	-45.2270	-41.5824
ZINC19815575	-44.6023	-41.5824
ZINC06197172	-38.8128	-41.5798
ZINC00970801	-41.4137	-41.5793
ZINC08415302	-38.9882	-41.5792
ZINC08415302	-36.5055	-41.5792
ZINC09411199	-41.6263	-41.5777
ZINC00728999	-41.3549	-41.5774
ZINC19683216	-40.3042	-41.5774
ZINC02055085	-38.6289	-41.5757
ZINC02055085	-36.8546	-41.5757
ZINC08442217	-44.7937	-41.5743
ZINC05689996	-36.9069	-41.5742
ZINC18083003	-38.2603	-41.5739
ZINC18083003	-38.2117	-41.5739
ZINC04649850	-38.7282	-41.5727
ZINC02738878	-38.9438	-41.5716
ZINC39929503	-37.6210	-41.5677
ZINC02088456	-38.9154	-41.5667
ZINC02755344	-39.1786	-41.5655
ZINC08779300	-42.2769	-41.5630
ZINC05807248	-40.3122	-41.5627
ZINC05807248	-39.5894	-41.5627
ZINC05408602	-40.0987	-41.5618
ZINC04062127	-38.4612	-41.5581
ZINC04062127	-38.4314	-41.5581
ZINC08441414	-38.5799	-41.5539
ZINC08439430	-37.4260	-41.5533
ZINC20623579	-42.1853	-41.5504
ZINC13388585	-38.9832	-41.5500
ZINC08440089	-44.3260	-41.5495
ZINC08440089	-43.7912	-41.5495
ZINC19827981	-39.9731	-41.5490
ZINC19827981	-39.7222	-41.5490
ZINC05343458	-42.7838	-41.5484
ZINC05343458	-42.2615	-41.5484
ZINC00721238	-40.9285	-41.5442
ZINC00721238	-38.8795	-41.5442
ZINC04114803	-39.7031	-41.5440
ZINC08453867	-37.6880	-41.5427
ZINC06144059	-50.2154	-41.5401
ZINC13569421	-38.6845	-41.5395
ZINC13569421	-38.3129	-41.5395
ZINC09302017	-46.3009	-41.5388
ZINC09302017	-45.3732	-41.5388
ZINC18061908	-37.5931	-41.5362
ZINC19788525	-38.6609	-41.5309
ZINC00626691	-45.1423	-41.5309
ZINC00626691	-44.6779	-41.5309
ZINC06187867	-39.6099	-41.5305
ZINC13601779	-40.7279	-41.5304
ZINC13601779	-39.3565	-41.5304

ZINC68677339	-44.6101	-41.5287
ZINC08438683	-39.9347	-41.5284
ZINC08438683	-39.3313	-41.5284
ZINC17154701	-36.6146	-41.5265
ZINC04067674	-37.4993	-41.5261
ZINC00707956	-38.6202	-41.5245
ZINC00707956	-38.6027	-41.5245
ZINC00707956	-37.1357	-41.5245
ZINC04067947	-38.6175	-41.5221
ZINC04067659	-38.9725	-41.5193
ZINC04335101	-38.5337	-41.5190
ZINC13371052	-40.1667	-41.5185
ZINC13371052	-40.0102	-41.5185
ZINC13371052	-38.1050	-41.5185
ZINC04062859	-38.5158	-41.5181
ZINC09043755	-43.8774	-41.5180
ZINC09043755	-43.2149	-41.5180
ZINC04065037	-38.5280	-41.5179
ZINC36055688	-37.8190	-41.5176
ZINC00702691	-38.0742	-41.5172
ZINC01413485	-44.3674	-41.5171
ZINC01413485	-42.5884	-41.5171
ZINC04066374	-37.0325	-41.5164
ZINC04066374	-36.9468	-41.5164
ZINC12523686	-44.6549	-41.5132
ZINC01213878	-39.3747	-41.5128
ZINC19926689	-48.9834	-41.5120
ZINC19926689	-40.9337	-41.5120
ZINC12382603	-38.3099	-41.5100
ZINC04067814	-38.8605	-41.5090
ZINC19926567	-39.2406	-41.5088
ZINC13942149	-39.2926	-41.5052
ZINC17154917	-39.1393	-41.5043
ZINC04015493	-36.9709	-41.5041
ZINC01413448	-42.3848	-41.5032
ZINC01413448	-41.1940	-41.5032
ZINC02494902	-38.2187	-41.5025
ZINC02494902	-36.5151	-41.5025
ZINC02494902	-36.2996	-41.5025
ZINC00726598	-37.7974	-41.5023
ZINC00726598	-37.6028	-41.5023
ZINC00726598	-37.1769	-41.5023
ZINC08439522	-42.0298	-41.4977
ZINC08439522	-40.7498	-41.4977
ZINC19872459	-41.4735	-41.4973
ZINC02083577	-40.7577	-41.4961
ZINC09087740	-37.2898	-41.4960
ZINC09087740	-37.2898	-41.4960
ZINC09186800	-48.9364	-41.4951
ZINC09186800	-46.9739	-41.4951
ZINC04066553	-38.2647	-41.4944
ZINC04066553	-38.0495	-41.4944
ZINC20392270	-49.6425	-41.4933
ZINC09374621	-49.3188	-41.4931

ZINC09374621	-44.8865	-41.4931
ZINC09357785	-41.2854	-41.4921
ZINC10290747	-38.9328	-41.4920
ZINC09357137	-41.4705	-41.4884
ZINC09357137	-41.3424	-41.4884
ZINC04671130	-41.0191	-41.4879
ZINC02055844	-42.9502	-41.4874
ZINC17145465	-42.5779	-41.4856
ZINC17145465	-39.6965	-41.4856
ZINC19938477	-46.6615	-41.4849
ZINC85421470	-37.4492	-41.4842
ZINC09240257	-44.4452	-41.4839
ZINC09240257	-42.5632	-41.4839
ZINC09240257	-42.4759	-41.4839
ZINC04067563	-39.2980	-41.4801
ZINC04067563	-36.9097	-41.4801
ZINC04067563	-36.7872	-41.4801
ZINC13497418	-43.8174	-41.4793
ZINC13497418	-42.1441	-41.4793
ZINC08386302	-46.4887	-41.4787
ZINC08386302	-43.4823	-41.4787
ZINC00702437	-41.5085	-41.4774
ZINC00702437	-39.7790	-41.4774
ZINC00702437	-37.6226	-41.4774
ZINC08462320	-40.4735	-41.4755
ZINC06136876	-39.0816	-41.4728
ZINC06136876	-37.2044	-41.4728
ZINC01213872	-40.1768	-41.4724
ZINC08385207	-38.6846	-41.4720
ZINC08385207	-38.1805	-41.4720
ZINC04780865	-41.0716	-41.4715
ZINC06136878	-42.7716	-41.4714
ZINC06136878	-40.5241	-41.4714
ZINC06136878	-37.8451	-41.4714
ZINC06197101	-40.5952	-41.4710
ZINC08438703	-38.9247	-41.4706
ZINC09312722	-38.0564	-41.4696
ZINC06195977	-41.5191	-41.4691
ZINC06196513	-38.1962	-41.4671
ZINC19938358	-39.2569	-41.4657
ZINC13577212	-41.9779	-41.4634
ZINC30768719	-39.8464	-41.4628
ZINC04114800	-40.6744	-41.4605
ZINC04114800	-40.2280	-41.4605
ZINC19360126	-36.7073	-41.4593
ZINC04060783	-38.3034	-41.4580
ZINC04060783	-38.1393	-41.4580
ZINC04060783	-37.8175	-41.4580
ZINC00647149	-41.1220	-41.4537
ZINC06282986	-43.2853	-41.4516
ZINC06282986	-42.6015	-41.4516
ZINC00702757	-39.3139	-41.4490
ZINC04061892	-37.5807	-41.4485
ZINC05408492	-44.5595	-41.4481

ZINC16946511	-40.3410	-41.4478
ZINC09358837	-38.0257	-41.4466
ZINC09358837	-37.5247	-41.4466
ZINC20150084	-46.6515	-41.4450
ZINC06386085	-36.7383	-41.4398
ZINC08396806	-40.2800	-41.4374
ZINC08396806	-38.7905	-41.4374
ZINC02135970	-42.9305	-41.4353
ZINC19889490	-38.3023	-41.4353
ZINC00719483	-42.7314	-41.4351
ZINC08439517	-47.4224	-41.4343
ZINC08414876	-37.2883	-41.4304
ZINC09191239	-42.1707	-41.4303
ZINC09191239	-39.1991	-41.4303
ZINC19889689	-38.3828	-41.4286
ZINC19360201	-37.6766	-41.4280
ZINC09123615	-49.3582	-41.4267
ZINC09123615	-46.1224	-41.4267
ZINC04661376	-37.6691	-41.4262
ZINC00823623	-39.3720	-41.4253
ZINC00702751	-42.2428	-41.4250
ZINC00702751	-40.6046	-41.4250
ZINC05918665	-43.5696	-41.4242
ZINC08441928	-41.6502	-41.4239
ZINC01016895	-38.1461	-41.4192
ZINC19897408	-37.6885	-41.4189
ZINC19733058	-37.7499	-41.4182
ZINC19733058	-36.7816	-41.4182
ZINC18125355	-39.9344	-41.4179
ZINC18125355	-39.6763	-41.4179
ZINC18125355	-37.8799	-41.4179
ZINC19866303	-44.4088	-41.4179
ZINC01450471	-38.8204	-41.4160
ZINC08437192	-46.3700	-41.4119
ZINC19889462	-37.6825	-41.4104
ZINC19889462	-36.8435	-41.4104
ZINC08439800	-38.0017	-41.4097
ZINC08439800	-36.5945	-41.4097
ZINC08439268	-37.6268	-41.4092
ZINC08439268	-37.1897	-41.4092
ZINC00645589	-41.2514	-41.4091
ZINC06493160	-42.4806	-41.4091
ZINC06197523	-41.3728	-41.4088
ZINC06197523	-40.5440	-41.4088
ZINC00853758	-38.9725	-41.4088
ZINC00853758	-38.9620	-41.4088
ZINC00853758	-36.9105	-41.4088
ZINC18244369	-44.2249	-41.4061
ZINC08439797	-39.1446	-41.4045
ZINC08439797	-39.0208	-41.4045
ZINC19872260	-41.4785	-41.4040
ZINC09374296	-39.2684	-41.4021
ZINC09374296	-37.6275	-41.4021
ZINC08022184	-39.4738	-41.4006

ZINC00292352	-37.7894	-41.3994
ZINC18066214	-40.5199	-41.3983
ZINC06197556	-37.9790	-41.3975
ZINC17139061	-40.8455	-41.3959
ZINC17139061	-39.6132	-41.3959
ZINC06137002	-40.1462	-41.3941
ZINC09068366	-40.9575	-41.3940
ZINC09068366	-39.3652	-41.3940
ZINC18128405	-40.1196	-41.3926
ZINC18128405	-36.4885	-41.3926
ZINC04649834	-39.0259	-41.3914
ZINC08818093	-38.0458	-41.3913
ZINC08818093	-37.0075	-41.3913
ZINC22075172	-40.1014	-41.3900
ZINC04062102	-39.2571	-41.3880
ZINC08973465	-40.4738	-41.3869
ZINC06136923	-40.7907	-41.3853
ZINC02160022	-38.8990	-41.3851
ZINC02160022	-37.1834	-41.3851
ZINC00823669	-38.0172	-41.3847
ZINC09356821	-39.2298	-41.3846
ZINC09356821	-37.9476	-41.3846
ZINC00629050	-42.3364	-41.3841
ZINC09235799	-41.0810	-41.3836
ZINC09235799	-40.8778	-41.3836
ZINC08439391	-40.3297	-41.3834
ZINC17163076	-49.7274	-41.3833
ZINC04066270	-39.0764	-41.3833
ZINC04066270	-38.7878	-41.3833
ZINC00645062	-40.8738	-41.3831
ZINC00704364	-36.5876	-41.3820
ZINC19366893	-37.0287	-41.3814
ZINC06137519	-39.0460	-41.3809
ZINC08425245	-38.3694	-41.3807
ZINC06015500	-40.4443	-41.3800
ZINC06015500	-38.9108	-41.3800
ZINC06015500	-38.4874	-41.3800
ZINC04199800	-38.7646	-41.3786
ZINC09280994	-43.7250	-41.3764
ZINC09280994	-41.1760	-41.3764
ZINC01884447	-38.2984	-41.3743
ZINC04284841	-39.5149	-41.3743
ZINC19210610	-36.7070	-41.3741
ZINC04626475	-39.3797	-41.3719
ZINC36065987	-40.8187	-41.3712
ZINC08426798	-39.6909	-41.3684
ZINC08415992	-39.5149	-41.3681
ZINC08424922	-38.7764	-41.3678
ZINC18115627	-42.1598	-41.3661
ZINC08450377	-41.0678	-41.3659
ZINC13765611	-38.8968	-41.3651
ZINC19871610	-38.1811	-41.3647
ZINC09425084	-43.9079	-41.3631
ZINC68712463	-41.3735	-41.3630

ZINC04066207	-39.7487	-41.3625
ZINC04066207	-39.6241	-41.3625
ZINC09436313	-40.1421	-41.3604
ZINC08413153	-40.2254	-41.3603
ZINC06784146	-39.1243	-41.3599
ZINC06784146	-37.4009	-41.3599
ZINC06015483	-37.5567	-41.3597
ZINC13634075	-39.8708	-41.3566
ZINC08386593	-38.1123	-41.3563
ZINC08439948	-39.6042	-41.3553
ZINC13286630	-36.5083	-41.3545
ZINC05806949	-40.4239	-41.3540
ZINC05806949	-39.4903	-41.3540
ZINC05812715	-39.3101	-41.3536
ZINC19731295	-40.2375	-41.3519
ZINC09361112	-41.3800	-41.3518
ZINC06162018	-39.7565	-41.3516
ZINC19881859	-41.2020	-41.3512
ZINC19872114	-43.4899	-41.3501
ZINC02057955	-45.2072	-41.3499
ZINC08438527	-44.7167	-41.3489
ZINC08427602	-39.0417	-41.3486
ZINC06148783	-36.6775	-41.3482
ZINC19802172	-40.8173	-41.3454
ZINC19802172	-40.6440	-41.3454
ZINC00708048	-36.6686	-41.3445
ZINC00702748	-46.6577	-41.3408
ZINC00702748	-44.0354	-41.3408
ZINC04780866	-39.5014	-41.3400
ZINC17085379	-44.0549	-41.3374
ZINC15952860	-45.9131	-41.3367
ZINC05595981	-37.2945	-41.3333
ZINC05595981	-36.4658	-41.3333
ZINC19872025	-37.6227	-41.3327
ZINC09177787	-43.1074	-41.3322
ZINC09177787	-42.1848	-41.3322
ZINC09177787	-42.1777	-41.3322
ZINC08424560	-38.1052	-41.3315
ZINC36065989	-40.0768	-41.3311
ZINC32609479	-37.6840	-41.3301
ZINC00629969	-40.6222	-41.3280
ZINC08818034	-40.0926	-41.3234
ZINC08818034	-39.6311	-41.3234
ZINC13800412	-42.2423	-41.3209
ZINC02475455	-38.0325	-41.3204
ZINC00999295	-37.9731	-41.3203
ZINC08432405	-41.6396	-41.3173
ZINC08432405	-40.4070	-41.3173
ZINC06009196	-46.2242	-41.3168
ZINC06444363	-37.5000	-41.3166
ZINC00844275	-39.6310	-41.3160
ZINC00844275	-38.2970	-41.3160
ZINC00844275	-38.0508	-41.3160
ZINC09413166	-39.5900	-41.3120

ZINC09014952	-39.5310	-41.3111
ZINC08411415	-40.1813	-41.3091
ZINC08411415	-39.9428	-41.3091
ZINC05689998	-36.9443	-41.3091
ZINC04452950	-39.1269	-41.3088
ZINC04048078	-38.4561	-41.3086
ZINC09235371	-48.1249	-41.3084
ZINC09235371	-43.5829	-41.3084
ZINC04065162	-39.4187	-41.3056
ZINC04065162	-39.2360	-41.3056
ZINC04632426	-41.0531	-41.3038
ZINC06442961	-39.5245	-41.3037
ZINC00709416	-38.2250	-41.3019
ZINC08424923	-36.9280	-41.3013
ZINC04066140	-37.2841	-41.3006
ZINC01413506	-44.7764	-41.2989
ZINC06162352	-40.4119	-41.2981
ZINC06162352	-37.2010	-41.2981
ZINC08744149	-41.3511	-41.2960
ZINC09244247	-43.0743	-41.2939
ZINC09244247	-42.6188	-41.2939
ZINC08386447	-43.8500	-41.2933
ZINC08386447	-39.7753	-41.2933
ZINC08440682	-38.2721	-41.2920
ZINC08440682	-37.6138	-41.2920
ZINC08440682	-36.2564	-41.2920
ZINC06195794	-42.9576	-41.2896
ZINC08439967	-39.6017	-41.2893
ZINC09046383	-41.4856	-41.2892
ZINC09046383	-40.3370	-41.2892
ZINC08416165	-39.1104	-41.2872
ZINC08416165	-38.3781	-41.2872
ZINC19909099	-43.0727	-41.2848
ZINC17161126	-42.0124	-41.2846
ZINC00703043	-41.0668	-41.2824
ZINC00703043	-40.9674	-41.2824
ZINC00844222	-45.3175	-41.2820
ZINC00844222	-42.3916	-41.2820
ZINC06703806	-38.0740	-41.2809
ZINC05817921	-38.4024	-41.2795
ZINC00632469	-44.9510	-41.2777
ZINC19774391	-43.4430	-41.2777
ZINC19774391	-39.1368	-41.2777
ZINC04199811	-38.1863	-41.2765
ZINC19851101	-40.4991	-41.2743
ZINC06602998	-38.1456	-41.2741
ZINC08413985	-37.8877	-41.2695
ZINC08413985	-37.0851	-41.2695
ZINC06197164	-39.3829	-41.2671
ZINC09424649	-40.9255	-41.2665
ZINC09424649	-39.1469	-41.2665
ZINC08695238	-45.3993	-41.2664
ZINC05408916	-41.1475	-41.2656
ZINC05408916	-37.1908	-41.2656

ZINC08435575	-43.2518	-41.2651
ZINC08411905	-37.2279	-41.2651
ZINC08439881	-39.8049	-41.2643
ZINC08439881	-38.2834	-41.2643
ZINC08439881	-37.5599	-41.2643
ZINC13512231	-41.1983	-41.2640
ZINC06162285	-37.6923	-41.2617
ZINC08439635	-40.3009	-41.2614
ZINC04148803	-41.6951	-41.2608
ZINC27346180	-37.4918	-41.2595
ZINC00648828	-38.2455	-41.2584
ZINC17166898	-40.9725	-41.2583
ZINC17166898	-40.2946	-41.2583
ZINC08426800	-41.4409	-41.2578
ZINC00822773	-38.7893	-41.2569
ZINC22972830	-38.4508	-41.2551
ZINC22972830	-36.6631	-41.2551
ZINC13388518	-36.6616	-41.2551
ZINC00702307	-40.5038	-41.2541
ZINC00702307	-39.5278	-41.2541
ZINC09184849	-40.9092	-41.2521
ZINC00861259	-38.9974	-41.2517
ZINC00861259	-38.8142	-41.2517
ZINC08919344	-38.7015	-41.2515
ZINC00691601	-40.0894	-41.2505
ZINC04658803	-36.4309	-41.2503
ZINC09308696	-47.3234	-41.2475
ZINC09308696	-45.0363	-41.2475
ZINC09308696	-44.9125	-41.2475
ZINC09308696	-44.0785	-41.2475
ZINC09308696	-43.1162	-41.2475
ZINC08414781	-37.1240	-41.2445
ZINC09359269	-41.3014	-41.2425
ZINC06148662	-36.6345	-41.2406
ZINC00703355	-41.6772	-41.2404
ZINC00703355	-41.3787	-41.2404
ZINC09244106	-41.8678	-41.2400
ZINC09244106	-39.6773	-41.2400
ZINC17887393	-38.4344	-41.2397
ZINC04632287	-41.6441	-41.2392
ZINC00349184	-37.5740	-41.2385
ZINC13045505	-39.6460	-41.2385
ZINC08425567	-38.4595	-41.2383
ZINC08425567	-36.7344	-41.2383
ZINC00645590	-39.5482	-41.2368
ZINC06162309	-40.5554	-41.2363
ZINC00180993	-39.5818	-41.2361
ZINC06194084	-39.5343	-41.2357
ZINC09043678	-39.7409	-41.2336
ZINC08424745	-43.4219	-41.2321
ZINC06137646	-41.8891	-41.2319
ZINC06137646	-39.6184	-41.2319
ZINC00821071	-42.2786	-41.2308
ZINC00821071	-40.5259	-41.2308

ZINC00821071	-38.9374	-41.2308
ZINC06812882	-37.5194	-41.2304
ZINC09015081	-37.7022	-41.2300
ZINC08466162	-37.5502	-41.2289
ZINC08466162	-37.2225	-41.2289
ZINC04075305	-39.0136	-41.2274
ZINC04075305	-37.2058	-41.2274
ZINC04632422	-41.9784	-41.2233
ZINC04632422	-40.2195	-41.2233
ZINC04114804	-38.9811	-41.2175
ZINC04114804	-36.4300	-41.2175
ZINC04629610	-43.2479	-41.2138
ZINC04629610	-42.0591	-41.2138
ZINC68705507	-36.4710	-41.2129
ZINC08442426	-38.4641	-41.2124
ZINC08442426	-37.4791	-41.2124
ZINC04693736	-37.0562	-41.2122
ZINC08439682	-36.9353	-41.2111
ZINC06137158	-38.3153	-41.2096
ZINC20171523	-49.9959	-41.2090
ZINC06658691	-39.8246	-41.2072
ZINC08441142	-38.7983	-41.2066
ZINC08441142	-38.6649	-41.2066
ZINC08441142	-38.0767	-41.2066
ZINC04681731	-39.4045	-41.2053
ZINC04062542	-37.4208	-41.2053
ZINC04062542	-36.6552	-41.2053
ZINC09353937	-39.5361	-41.2032
ZINC08414967	-39.1004	-41.2016
ZINC08414967	-38.8373	-41.2016
ZINC33370498	-43.2413	-41.2014
ZINC00726560	-39.2551	-41.1998
ZINC06196054	-37.3970	-41.1998
ZINC08415865	-38.9788	-41.1980
ZINC04114993	-41.5761	-41.1957
ZINC04114993	-40.7792	-41.1957
ZINC06149895	-41.0376	-41.1955
ZINC08440705	-37.9727	-41.1951
ZINC08440705	-37.6942	-41.1951
ZINC08440705	-37.4612	-41.1951
ZINC02679169	-39.0897	-41.1949
ZINC00997734	-38.1417	-41.1946
ZINC00709902	-38.7628	-41.1918
ZINC09373800	-39.7183	-41.1914
ZINC09373800	-39.0230	-41.1914
ZINC09272924	-42.8795	-41.1907
ZINC09272924	-42.6440	-41.1907
ZINC09272924	-41.1883	-41.1907
ZINC08441316	-41.7908	-41.1892
ZINC19361932	-42.6740	-41.1867
ZINC04632421	-43.1120	-41.1863
ZINC04632421	-42.6217	-41.1863
ZINC08433374	-41.8026	-41.1851
ZINC09046843	-38.8375	-41.1847

ZINC09046843	-37.7393	-41.1847
ZINC06162497	-38.3325	-41.1830
ZINC04565858	-38.1384	-41.1827
ZINC06148790	-37.2611	-41.1823
ZINC19901205	-44.3291	-41.1812
ZINC20533609	-44.3124	-41.1797
ZINC00702745	-46.6330	-41.1773
ZINC00702745	-44.8419	-41.1773
ZINC18057152	-41.4564	-41.1767
ZINC00617765	-37.8245	-41.1752
ZINC08441472	-43.4997	-41.1701
ZINC08441472	-42.2899	-41.1701
ZINC08827783	-39.3932	-41.1668
ZINC04066094	-36.8290	-41.1654
ZINC00707934	-37.9621	-41.1651
ZINC08437338	-40.7296	-41.1643
ZINC85381099	-36.3051	-41.1641
ZINC00886780	-39.4228	-41.1606
ZINC16734717	-40.3401	-41.1584
ZINC08715667	-38.5811	-41.1553
ZINC08715667	-37.9921	-41.1553
ZINC04060697	-36.3742	-41.1549
ZINC05446205	-37.3503	-41.1522
ZINC05446205	-36.8648	-41.1522
ZINC04068012	-41.2831	-41.1520
ZINC04068012	-40.7747	-41.1520
ZINC04068012	-38.4560	-41.1520
ZINC09329454	-40.6132	-41.1500
ZINC04629647	-38.7366	-41.1499
ZINC04619464	-38.4566	-41.1491
ZINC02088123	-40.0345	-41.1490
ZINC19897468	-37.9377	-41.1483
ZINC00327942	-41.0680	-41.1481
ZINC08438674	-43.7267	-41.1479
ZINC08438674	-40.4329	-41.1479
ZINC01413446	-41.7010	-41.1455
ZINC01413446	-40.9179	-41.1455
ZINC17138119	-40.9222	-41.1449
ZINC04312257	-38.7394	-41.1402
ZINC04312257	-37.5880	-41.1402
ZINC01889820	-38.4136	-41.1398
ZINC17166783	-41.1294	-41.1365
ZINC17166783	-39.5992	-41.1365
ZINC06444326	-36.5553	-41.1355
ZINC09244010	-39.0126	-41.1346
ZINC09244010	-37.5365	-41.1346
ZINC06137291	-40.7461	-41.1341
ZINC08414785	-43.5139	-41.1334
ZINC08414785	-42.3712	-41.1334
ZINC06137649	-41.8405	-41.1301
ZINC18032194	-38.0779	-41.1301
ZINC09123063	-45.0902	-41.1258
ZINC06136925	-40.0886	-41.1235
ZINC17161129	-44.0683	-41.1226

ZINC04004947	-37.6967	-41.1205
ZINC04004947	-36.4648	-41.1205
ZINC18127070	-41.1877	-41.1202
ZINC09411247	-39.8151	-41.1185
ZINC04019584	-41.6313	-41.1172
ZINC04019584	-40.5567	-41.1172
ZINC00726628	-37.9187	-41.1168
ZINC00726628	-37.4920	-41.1168
ZINC08440103	-40.5836	-41.1113
ZINC04019571	-38.7020	-41.1091
ZINC04019571	-38.5082	-41.1091
ZINC06162289	-38.4579	-41.1082
ZINC00844273	-41.2007	-41.1078
ZINC00844273	-39.7060	-41.1078
ZINC04957402	-38.7819	-41.1069
ZINC00948653	-43.0961	-41.1044
ZINC19923624	-36.8759	-41.1029
ZINC04417641	-39.1719	-41.1029
ZINC04417641	-38.3343	-41.1029
ZINC08435259	-41.8337	-41.1019
ZINC19359996	-38.4358	-41.1004
ZINC05458355	-39.7761	-41.1002
ZINC04285155	-40.6571	-41.0999
ZINC00702762	-39.6896	-41.0998
ZINC08439879	-38.6110	-41.0978
ZINC13286581	-38.7589	-41.0972
ZINC08425309	-36.6989	-41.0956
ZINC08450295	-39.9421	-41.0953
ZINC08450295	-38.4836	-41.0953
ZINC16948816	-38.7149	-41.0946
ZINC02340018	-38.1224	-41.0943
ZINC00714913	-37.1638	-41.0938
ZINC08413455	-42.1447	-41.0924
ZINC08413455	-41.3740	-41.0924
ZINC08430093	-39.2279	-41.0918
ZINC08430093	-38.7852	-41.0918
ZINC06015553	-36.7692	-41.0918
ZINC02088459	-38.8411	-41.0916
ZINC17241934	-40.5625	-41.0900
ZINC09459130	-43.1392	-41.0888
ZINC09459130	-41.9817	-41.0888
ZINC02568864	-36.6246	-41.0882
ZINC04626400	-36.7440	-41.0876
ZINC05447786	-40.3046	-41.0870
ZINC09374926	-40.7455	-41.0846
ZINC09374926	-40.3345	-41.0846
ZINC02397010	-38.0850	-41.0832
ZINC06162093	-37.5349	-41.0818
ZINC04019568	-41.0608	-41.0812
ZINC04019568	-40.0374	-41.0812
ZINC09311926	-38.9090	-41.0792
ZINC06162479	-39.7593	-41.0777
ZINC04066563	-36.6841	-41.0775
ZINC08438521	-43.5106	-41.0762

ZINC08438546	-41.8557	-41.0761
ZINC08462268	-39.5544	-41.0754
ZINC09123632	-38.3442	-41.0716
ZINC85406441	-37.3268	-41.0716
ZINC04067427	-40.7282	-41.0711
ZINC02858813	-42.6765	-41.0681
ZINC06148625	-39.2643	-41.0660
ZINC04067119	-36.9222	-41.0632
ZINC04418235	-37.8024	-41.0616
ZINC08429896	-39.9581	-41.0616
ZINC16115297	-40.4864	-41.0587
ZINC06195602	-40.4975	-41.0573
ZINC06195602	-39.8830	-41.0573
ZINC19568259	-39.5500	-41.0545
ZINC04123655	-39.9275	-41.0543
ZINC08433246	-40.3316	-41.0527
ZINC02055154	-41.3547	-41.0526
ZINC02087925	-38.6900	-41.0516
ZINC00627951	-38.5971	-41.0515
ZINC04626486	-36.9360	-41.0508
ZINC04730776	-39.2800	-41.0496
ZINC04730776	-38.2354	-41.0496
ZINC18084637	-38.7808	-41.0451
ZINC18084637	-38.0147	-41.0451
ZINC06197642	-37.0608	-41.0435
ZINC08415738	-39.1832	-41.0427
ZINC19929453	-43.4050	-41.0418
ZINC02455434	-39.7211	-41.0399
ZINC02455434	-39.2311	-41.0399
ZINC02455434	-36.4411	-41.0399
ZINC14161598	-46.2195	-41.0382
ZINC08433290	-39.8251	-41.0371
ZINC08433290	-39.7227	-41.0371
ZINC00728227	-36.5431	-41.0366
ZINC08921252	-44.6938	-41.0347
ZINC55250674	-39.1313	-41.0344
ZINC04066694	-37.5713	-41.0341
ZINC02262448	-37.7651	-41.0337
ZINC06196401	-36.7484	-41.0326
ZINC08444993	-38.0810	-41.0287
ZINC09008466	-45.7915	-41.0277
ZINC09008466	-43.5544	-41.0277
ZINC09008466	-43.0134	-41.0277
ZINC09008466	-42.0342	-41.0277
ZINC09008466	-41.8337	-41.0277
ZINC09374793	-40.7249	-41.0277
ZINC13126271	-36.5065	-41.0273
ZINC00633298	-39.5086	-41.0261
ZINC12625288	-42.3346	-41.0251
ZINC12625288	-41.9313	-41.0251
ZINC04061127	-37.7923	-41.0210
ZINC08415419	-37.9479	-41.0160
ZINC08415419	-37.2257	-41.0160
ZINC01414783	-43.9271	-41.0148

ZINC01414783	-41.5909	-41.0148
ZINC05918477	-37.3377	-41.0120
ZINC00640027	-47.5077	-41.0119
ZINC00823434	-38.6187	-41.0102
ZINC00823434	-38.3425	-41.0102
ZINC08415884	-38.9533	-41.0087
ZINC13942151	-36.5432	-41.0057
ZINC06015478	-39.6737	-41.0051
ZINC08383424	-38.4921	-41.0051
ZINC08413294	-37.3728	-41.0051
ZINC04062684	-37.2400	-41.0051
ZINC08413294	-36.8626	-41.0051
ZINC08413294	-36.8538	-41.0051
ZINC04062684	-36.7985	-41.0051
ZINC04043948	-38.2516	-41.0036
ZINC04043948	-36.9589	-41.0036
ZINC19850937	-40.5408	-41.0010
ZINC03656723	-46.5580	-40.9993
ZINC13569869	-45.8390	-40.9980
ZINC13569869	-44.2654	-40.9980
ZINC04067144	-40.3043	-40.9966
ZINC04067144	-40.2058	-40.9966
ZINC07602447	-38.5285	-40.9959
ZINC19791711	-40.2061	-40.9952
ZINC09110171	-39.9882	-40.9950
ZINC15356959	-36.6226	-40.9939
ZINC04780828	-36.4864	-40.9931
ZINC00645507	-38.4815	-40.9915
ZINC08985994	-39.6914	-40.9912
ZINC09353936	-38.5753	-40.9911
ZINC09329514	-37.2623	-40.9906
ZINC02064479	-40.6457	-40.9902
ZINC06137165	-38.8610	-40.9877
ZINC06137165	-37.6176	-40.9877
ZINC00381998	-36.8995	-40.9843
ZINC19872328	-39.3900	-40.9841
ZINC06197166	-38.5282	-40.9835
ZINC08437292	-43.3748	-40.9800
ZINC04063195	-37.2015	-40.9791
ZINC13402468	-44.6801	-40.9768
ZINC08441469	-37.7665	-40.9751
ZINC08441469	-37.6989	-40.9751
ZINC08440640	-39.7434	-40.9749
ZINC09009036	-40.6041	-40.9744
ZINC09009036	-38.3787	-40.9744
ZINC05408892	-37.0926	-40.9743
ZINC05408892	-37.0906	-40.9743
ZINC04905426	-38.0335	-40.9664
ZINC00703357	-43.5718	-40.9661
ZINC00703357	-41.8735	-40.9661
ZINC19897464	-41.0260	-40.9654
ZINC08914745	-40.0849	-40.9648
ZINC08914745	-39.5526	-40.9648
ZINC09160473	-42.2537	-40.9617

ZINC09160473	-38.1509	-40.9617
ZINC00702368	-42.5485	-40.9600
ZINC00702368	-41.2839	-40.9600
ZINC08894831	-44.3637	-40.9583
ZINC04062305	-38.6991	-40.9580
ZINC04062305	-38.2299	-40.9580
ZINC05807017	-38.8119	-40.9574
ZINC16661313	-37.6139	-40.9571
ZINC08444432	-38.6417	-40.9564
ZINC06382186	-40.1982	-40.9548
ZINC06196027	-37.8266	-40.9530
ZINC04068145	-40.2408	-40.9526
ZINC08440703	-39.3548	-40.9507
ZINC08440703	-38.7857	-40.9507
ZINC08440703	-38.1663	-40.9507
ZINC19938467	-42.3990	-40.9498
ZINC04066452	-38.9766	-40.9496
ZINC04066452	-38.1374	-40.9496
ZINC09184893	-38.5122	-40.9484
ZINC08437232	-40.7813	-40.9480
ZINC04620447	-43.6218	-40.9462
ZINC04620447	-38.0639	-40.9462
ZINC00987734	-51.2787	-40.9455
ZINC15015784	-36.7627	-40.9445
ZINC06143942	-39.4653	-40.9429
ZINC13388350	-37.5490	-40.9429
ZINC13388350	-37.4519	-40.9429
ZINC05463332	-43.0622	-40.9426
ZINC02332322	-40.2577	-40.9402
ZINC00969335	-38.9406	-40.9400
ZINC12138625	-38.2972	-40.9399
ZINC00079488	-37.5664	-40.9395
ZINC08437405	-36.5584	-40.9363
ZINC09088744	-37.6825	-40.9353
ZINC19832189	-39.9999	-40.9351
ZINC08441398	-43.3979	-40.9350
ZINC08441398	-39.5889	-40.9350
ZINC06665586	-44.4408	-40.9350
ZINC06148750	-37.4266	-40.9350
ZINC17154765	-38.3519	-40.9330
ZINC04067041	-38.6004	-40.9329
ZINC17166868	-39.6496	-40.9320
ZINC17166868	-38.1119	-40.9320
ZINC17166868	-37.5131	-40.9320
ZINC06162002	-38.8831	-40.9317
ZINC04062142	-39.9802	-40.9314
ZINC09312879	-40.1106	-40.9305
ZINC09312879	-37.4107	-40.9305
ZINC00703096	-43.4892	-40.9303
ZINC00703096	-42.7275	-40.9303
ZINC04168854	-37.4913	-40.9296
ZINC08438782	-39.9712	-40.9288
ZINC08440499	-40.6385	-40.9280
ZINC06162017	-40.5741	-40.9265

ZINC06162017	-39.5639	-40.9265
ZINC00629946	-39.0986	-40.9249
ZINC00726620	-36.4194	-40.9248
ZINC20233915	-45.6898	-40.9223
ZINC08439947	-39.4473	-40.9208
ZINC13388482	-37.5207	-40.9198
ZINC18181480	-36.5613	-40.9162
ZINC03998252	-37.7912	-40.9157
ZINC01413496	-43.4796	-40.9155
ZINC08439752	-44.3089	-40.9149
ZINC08439752	-39.4183	-40.9149
ZINC18056923	-39.1382	-40.9143
ZINC04593043	-39.3479	-40.9131
ZINC06196069	-38.4514	-40.9093
ZINC04099262	-37.8712	-40.9080
ZINC00821068	-38.9610	-40.9034
ZINC00821068	-38.8854	-40.9034
ZINC00821068	-37.0964	-40.9034
ZINC08456704	-40.5384	-40.9029
ZINC08456704	-37.7764	-40.9029
ZINC00866537	-40.8535	-40.8989
ZINC00866537	-40.0234	-40.8989
ZINC08433400	-44.3893	-40.8986
ZINC05929516	-46.1490	-40.8980
ZINC08706620	-50.0215	-40.8974
ZINC08706620	-41.7098	-40.8974
ZINC13570647	-47.8396	-40.8973
ZINC13570647	-46.9090	-40.8973
ZINC17154921	-38.9216	-40.8963
ZINC08440140	-37.1949	-40.8951
ZINC20058769	-37.9078	-40.8941
ZINC18210980	-38.5351	-40.8936
ZINC18210980	-36.9884	-40.8936
ZINC06148680	-40.7884	-40.8936
ZINC09359662	-40.0172	-40.8889
ZINC09359662	-38.8672	-40.8889
ZINC12417836	-39.4609	-40.8883
ZINC12417836	-38.8794	-40.8883
ZINC00996661	-39.1281	-40.8863
ZINC13826119	-37.1080	-40.8852
ZINC00710875	-39.2952	-40.8847
ZINC00710875	-38.6249	-40.8847
ZINC08437722	-38.3583	-40.8846
ZINC08445386	-50.6393	-40.8841
ZINC17159934	-39.0985	-40.8839
ZINC17159934	-39.0196	-40.8839
ZINC19363164	-36.7125	-40.8833
ZINC00702683	-41.7885	-40.8785
ZINC00702683	-41.2789	-40.8785
ZINC06148524	-41.7357	-40.8784
ZINC04066241	-39.1399	-40.8775
ZINC04066241	-38.1957	-40.8775
ZINC08440063	-36.3989	-40.8757
ZINC08415107	-38.9519	-40.8728

ZINC08415107	-37.8124	-40.8728
ZINC08415107	-36.6370	-40.8728
ZINC00629941	-38.4830	-40.8722
ZINC08416390	-38.2377	-40.8714
ZINC08416390	-37.3639	-40.8714
ZINC05532781	-36.4579	-40.8694
ZINC00065593	-36.5000	-40.8675
ZINC19815578	-37.4161	-40.8665
ZINC06162042	-39.5993	-40.8659
ZINC06162042	-39.1241	-40.8659
ZINC00725738	-39.5290	-40.8657
ZINC00623262	-40.2328	-40.8630
ZINC13552857	-40.5627	-40.8630
ZINC08384505	-42.2362	-40.8617
ZINC04014109	-40.1955	-40.8617
ZINC06162009	-39.2750	-40.8616
ZINC00726502	-38.2251	-40.8615
ZINC00726502	-36.9357	-40.8615
ZINC00726502	-36.6762	-40.8615
ZINC01771875	-36.5914	-40.8601
ZINC04626402	-38.0422	-40.8579
ZINC13569871	-45.6939	-40.8578
ZINC13569871	-43.5026	-40.8578
ZINC00853740	-38.6117	-40.8571
ZINC16114950	-39.8184	-40.8546
ZINC00726488	-38.5374	-40.8534
ZINC00726488	-38.1450	-40.8534
ZINC00726488	-37.0484	-40.8534
ZINC18066212	-39.3520	-40.8498
ZINC05053267	-38.9429	-40.8488
ZINC05053267	-36.7835	-40.8488
ZINC06195798	-37.4927	-40.8476
ZINC04065864	-37.9779	-40.8473
ZINC04415938	-38.1471	-40.8442
ZINC04415938	-37.6665	-40.8442
ZINC00349400	-36.5323	-40.8425
ZINC12436609	-45.8418	-40.8414
ZINC12436609	-45.4099	-40.8414
ZINC12436609	-42.8145	-40.8414
ZINC12436609	-42.6841	-40.8414
ZINC12436609	-40.6404	-40.8414
ZINC00725685	-38.4338	-40.8409
ZINC08981618	-43.1658	-40.8399
ZINC08981618	-42.3350	-40.8399
ZINC08981618	-41.8304	-40.8399
ZINC08981618	-41.4404	-40.8399
ZINC08416358	-41.6695	-40.8398
ZINC09089036	-37.4153	-40.8396
ZINC06196076	-40.0821	-40.8383
ZINC04062797	-36.5606	-40.8382
ZINC00631015	-46.6790	-40.8377
ZINC06136825	-38.4502	-40.8369
ZINC06136825	-36.4627	-40.8369
ZINC06593623	-36.2773	-40.8362

ZINC09008300	-46.5548	-40.8356
ZINC19570478	-37.5827	-40.8337
ZINC19570478	-37.0599	-40.8337
ZINC36646256	-40.0489	-40.8335
ZINC00707868	-38.2311	-40.8323
ZINC06148610	-39.2681	-40.8313
ZINC16115874	-38.0160	-40.8309
ZINC00707932	-40.3525	-40.8307
ZINC00707932	-40.1461	-40.8307
ZINC00707932	-40.0303	-40.8307
ZINC09065546	-40.1410	-40.8296
ZINC00812649	-37.9862	-40.8288
ZINC00870367	-38.1780	-40.8286
ZINC13108194	-37.3900	-40.8282
ZINC06196091	-44.9042	-40.8256
ZINC08442506	-48.9224	-40.8241
ZINC08442506	-48.7693	-40.8241
ZINC00645595	-45.2564	-40.8236
ZINC05432294	-36.6927	-40.8236
ZINC08715665	-38.6151	-40.8214
ZINC08715665	-37.8577	-40.8214
ZINC09357140	-41.0213	-40.8190
ZINC04679335	-38.7288	-40.8189
ZINC04679335	-36.5354	-40.8189
ZINC08413559	-38.9715	-40.8182
ZINC08413559	-38.1665	-40.8182
ZINC08413559	-37.9226	-40.8182
ZINC08383894	-42.6905	-40.8173
ZINC08383894	-42.6662	-40.8173
ZINC00673979	-39.0935	-40.8160
ZINC19938466	-45.5158	-40.8138
ZINC06195809	-41.8321	-40.8133
ZINC00035919	-37.2861	-40.8107
ZINC00688917	-40.0431	-40.8105
ZINC09110924	-40.0348	-40.8098
ZINC04059818	-37.8611	-40.8088
ZINC04059818	-37.5730	-40.8088
ZINC08462354	-38.7291	-40.8082
ZINC85413281	-37.9183	-40.8082
ZINC04460168	-37.8596	-40.8038
ZINC04460168	-37.4833	-40.8038
ZINC19871616	-40.7462	-40.8009
ZINC04312214	-39.2743	-40.7988
ZINC04312214	-38.4573	-40.7988
ZINC08415345	-36.6715	-40.7988
ZINC04075152	-41.9728	-40.7946
ZINC04075152	-41.2388	-40.7946
ZINC02057290	-41.1248	-40.7945
ZINC08384563	-45.7900	-40.7940
ZINC06197099	-39.0679	-40.7908
ZINC08437444	-36.6491	-40.7886
ZINC00707978	-36.9040	-40.7878
ZINC00853494	-36.4664	-40.7877
ZINC04512860	-36.7491	-40.7863

ZINC08441938	-40.3876	-40.7848
ZINC06162246	-38.2829	-40.7838
ZINC00710564	-39.7666	-40.7828
ZINC09087741	-37.7094	-40.7815
ZINC00625693	-39.5114	-40.7803
ZINC08437176	-39.6384	-40.7799
ZINC08439429	-38.9036	-40.7799
ZINC09067280	-48.1740	-40.7796
ZINC09067280	-47.1497	-40.7796
ZINC09067280	-44.9337	-40.7796
ZINC08439325	-40.3005	-40.7793
ZINC08439325	-39.3910	-40.7793
ZINC18145955	-39.7062	-40.7784
ZINC19802176	-42.7090	-40.7783
ZINC19802176	-41.1552	-40.7783
ZINC08415126	-36.9386	-40.7778
ZINC08415126	-36.5826	-40.7778
ZINC04635180	-41.3718	-40.7774
ZINC04635180	-41.0811	-40.7774
ZINC19208998	-39.2231	-40.7770
ZINC18106441	-42.6575	-40.7767
ZINC04065828	-37.2197	-40.7757
ZINC04065828	-36.8403	-40.7757
ZINC04631398	-39.5555	-40.7757
ZINC00726569	-38.7448	-40.7748
ZINC00726569	-38.6824	-40.7748
ZINC00726569	-37.1658	-40.7748
ZINC06137650	-39.8892	-40.7744
ZINC06137650	-39.7663	-40.7744
ZINC15777269	-49.2918	-40.7728
ZINC15777269	-48.3207	-40.7728
ZINC00826271	-36.4243	-40.7727
ZINC05409069	-38.2859	-40.7711
ZINC05409069	-37.4496	-40.7711
ZINC06195973	-38.2755	-40.7691
ZINC08387543	-38.2033	-40.7691
ZINC04065876	-37.9919	-40.7685
ZINC04065876	-37.4361	-40.7685
ZINC04065876	-37.2450	-40.7685
ZINC13424227	-41.3495	-40.7680
ZINC13424227	-39.6101	-40.7680
ZINC06162038	-37.2408	-40.7667
ZINC08411113	-38.0177	-40.7655
ZINC00034870	-38.2860	-40.7651
ZINC05917787	-42.0593	-40.7640
ZINC05917787	-39.8841	-40.7640
ZINC04048076	-36.5179	-40.7626
ZINC19871700	-40.2471	-40.7613
ZINC36646206	-41.1567	-40.7607
ZINC00726500	-38.4828	-40.7597
ZINC00726500	-37.4996	-40.7597
ZINC06162486	-40.7810	-40.7583
ZINC68713696	-44.0164	-40.7567
ZINC13119814	-39.1005	-40.7567

ZINC08444433	-38.8739	-40.7563
ZINC19329518	-36.4304	-40.7522
ZINC09301207	-37.9606	-40.7520
ZINC02475452	-38.1610	-40.7515
ZINC02475452	-36.5211	-40.7515
ZINC02475452	-36.4577	-40.7515
ZINC05343174	-42.0814	-40.7508
ZINC05343174	-37.2973	-40.7508
ZINC19860362	-38.9949	-40.7501
ZINC57092969	-38.2436	-40.7486
ZINC57092969	-37.7071	-40.7486
ZINC05053254	-38.9500	-40.7480
ZINC05053254	-36.8795	-40.7480
ZINC04658458	-37.7001	-40.7475
ZINC19938616	-36.8500	-40.7470
ZINC08415727	-37.3999	-40.7464
ZINC08415727	-36.5220	-40.7464
ZINC08838105	-40.1878	-40.7462
ZINC04905389	-39.3248	-40.7451
ZINC00861222	-37.9891	-40.7434
ZINC00861222	-37.0286	-40.7434
ZINC09235094	-42.3538	-40.7416
ZINC06194528	-38.0626	-40.7403
ZINC08416375	-37.6512	-40.7396
ZINC01271999	-44.4623	-40.7391
ZINC08397264	-38.9323	-40.7387
ZINC08922541	-39.1379	-40.7385
ZINC20876076	-37.7218	-40.7374
ZINC02077397	-49.3838	-40.7368
ZINC08441728	-39.5403	-40.7366
ZINC04417594	-39.3214	-40.7360
ZINC08860017	-40.8886	-40.7351
ZINC00702768	-40.2794	-40.7348
ZINC01268606	-41.4169	-40.7324
ZINC08415655	-41.4583	-40.7323
ZINC08415655	-38.3370	-40.7323
ZINC06270523	-45.3579	-40.7303
ZINC09122084	-38.7231	-40.7268
ZINC06197236	-36.6648	-40.7252
ZINC09009197	-43.2915	-40.7241
ZINC09110196	-39.3144	-40.7234
ZINC09234826	-39.1529	-40.7230
ZINC09234826	-38.4768	-40.7230
ZINC06137270	-38.0029	-40.7218
ZINC09271655	-46.1385	-40.7217
ZINC09271655	-45.7599	-40.7217
ZINC09271655	-44.5592	-40.7217
ZINC09271655	-44.4382	-40.7217
ZINC09271655	-43.9649	-40.7217
ZINC06162100	-38.4218	-40.7169
ZINC05624798	-37.0272	-40.7164
ZINC00693179	-39.0450	-40.7157
ZINC01471224	-37.8324	-40.7131
ZINC08425385	-37.2942	-40.7119

ZINC09271363	-41.9097	-40.7113
ZINC06162324	-40.1096	-40.7108
ZINC06162324	-39.8711	-40.7108
ZINC19872204	-41.2016	-40.7096
ZINC04646694	-39.5663	-40.7094
ZINC17166801	-43.5220	-40.7073
ZINC17166801	-41.6058	-40.7073
ZINC17166801	-40.0471	-40.7073
ZINC19872211	-40.6064	-40.7070
ZINC07077628	-40.2129	-40.7066
ZINC09270973	-45.7752	-40.7064
ZINC09270973	-45.3150	-40.7064
ZINC09270973	-41.3879	-40.7064
ZINC04067894	-41.7363	-40.7064
ZINC04067894	-40.8630	-40.7064
ZINC04067894	-38.4435	-40.7064
ZINC00674029	-40.5954	-40.7060
ZINC06149898	-38.9891	-40.7056
ZINC19938397	-40.2272	-40.7034
ZINC06194264	-37.3491	-40.7030
ZINC06197553	-38.3639	-40.7028
ZINC00626535	-45.3618	-40.7026
ZINC00626535	-42.6080	-40.7026
ZINC08444992	-39.7436	-40.7014
ZINC04065659	-38.1644	-40.7007
ZINC09271437	-41.5929	-40.7003
ZINC09271437	-41.5712	-40.7003
ZINC13124115	-43.7312	-40.6993
ZINC04692898	-38.2301	-40.6956
ZINC08425568	-39.3921	-40.6936
ZINC08425568	-38.0752	-40.6936
ZINC08425568	-38.0407	-40.6936
ZINC19872222	-41.0644	-40.6935
ZINC00729508	-37.2764	-40.6923
ZINC00729508	-36.6286	-40.6923
ZINC00647313	-40.1788	-40.6914
ZINC02061461	-46.9301	-40.6908
ZINC09089347	-37.7465	-40.6889
ZINC20573739	-40.2425	-40.6881
ZINC20573739	-40.1143	-40.6881
ZINC20573739	-37.4035	-40.6881
ZINC08437266	-40.7736	-40.6866
ZINC04719123	-38.9322	-40.6850
ZINC04719123	-38.3791	-40.6850
ZINC00679180	-38.8684	-40.6841
ZINC00679180	-38.4906	-40.6841
ZINC19314628	-40.5960	-40.6827
ZINC09110989	-38.0527	-40.6811
ZINC00679392	-41.7164	-40.6806
ZINC04993675	-39.5634	-40.6801
ZINC04993675	-38.0346	-40.6801
ZINC06995625	-44.1212	-40.6794
ZINC85384924	-39.3685	-40.6776
ZINC09437085	-40.4485	-40.6766

ZINC05053279	-38.5699	-40.6765
ZINC05053279	-37.4893	-40.6765
ZINC06195771	-37.4581	-40.6750
ZINC06195771	-36.5944	-40.6750
ZINC36646207	-44.0704	-40.6744
ZINC02180541	-39.3379	-40.6735
ZINC04666049	-40.1082	-40.6673
ZINC00077145	-39.7238	-40.6672
ZINC01856818	-36.7939	-40.6666
ZINC06442947	-38.1814	-40.6643
ZINC05432303	-38.0895	-40.6637
ZINC17186283	-37.1351	-40.6630
ZINC27823079	-37.4850	-40.6605
ZINC09359422	-41.1558	-40.6602
ZINC08440786	-39.0320	-40.6592
ZINC04074990	-40.5612	-40.6585
ZINC04074990	-40.2502	-40.6585
ZINC13551952	-40.0972	-40.6583
ZINC04009322	-38.6521	-40.6579
ZINC04009322	-38.2779	-40.6579
ZINC17166777	-36.7752	-40.6562
ZINC19332972	-37.9537	-40.6558
ZINC09461227	-40.9255	-40.6554
ZINC09461227	-40.4443	-40.6554
ZINC02455402	-36.7924	-40.6549
ZINC57364348	-39.2493	-40.6537
ZINC00988126	-43.7579	-40.6521
ZINC04417962	-39.3232	-40.6518
ZINC08383701	-46.4038	-40.6513
ZINC00702692	-39.8935	-40.6468
ZINC00702692	-39.6305	-40.6468
ZINC06137196	-39.5922	-40.6438
ZINC00359406	-37.5770	-40.6377
ZINC09045949	-41.0231	-40.6366
ZINC09463217	-48.0142	-40.6357
ZINC02055879	-40.1566	-40.6344
ZINC19287617	-37.6146	-40.6341
ZINC08439432	-41.2922	-40.6338
ZINC08438777	-44.2896	-40.6329
ZINC06137147	-37.2438	-40.6324
ZINC06137147	-36.2960	-40.6324
ZINC04066040	-39.1153	-40.6323
ZINC04043899	-39.5832	-40.6322
ZINC08414831	-39.6204	-40.6316
ZINC08414831	-39.0351	-40.6316
ZINC08416329	-40.4797	-40.6315
ZINC08413868	-37.2847	-40.6314
ZINC08413868	-37.1033	-40.6314
ZINC05446163	-42.4813	-40.6312
ZINC00850069	-43.7345	-40.6310
ZINC19832330	-41.6423	-40.6291
ZINC02064477	-42.7028	-40.6278
ZINC00995188	-36.9967	-40.6256
ZINC08715766	-43.3189	-40.6241

ZINC16115403	-37.0338	-40.6238
ZINC08836057	-41.7870	-40.6220
ZINC08439878	-40.7278	-40.6210
ZINC17147259	-38.3828	-40.6193
ZINC17147259	-37.6740	-40.6193
ZINC13231940	-41.6060	-40.6187
ZINC06195388	-40.8539	-40.6187
ZINC04285212	-39.4731	-40.6180
ZINC04285212	-38.1248	-40.6180
ZINC19552402	-37.0525	-40.6176
ZINC08426339	-39.9608	-40.6158
ZINC09015452	-40.0626	-40.6142
ZINC06196052	-38.7769	-40.6140
ZINC05408353	-42.4815	-40.6132
ZINC68755241	-39.2879	-40.6113
ZINC08426071	-38.2600	-40.6110
ZINC09292881	-41.3272	-40.6107
ZINC09292881	-38.8492	-40.6107
ZINC08415571	-38.9401	-40.6082
ZINC08996874	-45.3329	-40.6061
ZINC08996874	-43.5044	-40.6061
ZINC08996874	-43.3322	-40.6061
ZINC08996874	-42.6423	-40.6061
ZINC08996874	-41.6801	-40.6061
ZINC09177182	-38.7410	-40.6059
ZINC09177182	-37.9073	-40.6059
ZINC05338368	-37.3144	-40.6057
ZINC19926522	-43.4229	-40.6047
ZINC08733406	-38.2108	-40.6042
ZINC00651280	-40.4858	-40.6039
ZINC02137598	-47.7925	-40.5996
ZINC17167357	-40.8601	-40.5981
ZINC09425159	-43.5453	-40.5980
ZINC08386051	-37.0678	-40.5966
ZINC09042829	-39.4245	-40.5960
ZINC13126517	-38.6241	-40.5946
ZINC13126517	-36.5322	-40.5946
ZINC00848133	-38.0314	-40.5939
ZINC04067364	-38.2524	-40.5936
ZINC04067364	-37.4249	-40.5936
ZINC35287284	-42.3948	-40.5935
ZINC00702966	-49.7470	-40.5932
ZINC00702966	-46.5336	-40.5932
ZINC00702966	-46.2846	-40.5932
ZINC00844242	-40.8006	-40.5919
ZINC00844242	-38.7416	-40.5919
ZINC00844242	-38.6206	-40.5919
ZINC13286632	-39.9251	-40.5906
ZINC13286632	-39.6828	-40.5906
ZINC08969913	-39.9604	-40.5894
ZINC02091675	-40.1968	-40.5893
ZINC01413447	-42.6057	-40.5854
ZINC01413447	-41.2269	-40.5854
ZINC01002798	-37.6758	-40.5839

ZINC09089202	-45.6020	-40.5825
ZINC09089202	-44.6018	-40.5825
ZINC06230238	-40.8921	-40.5807
ZINC15015952	-37.9356	-40.5804
ZINC15015952	-36.6971	-40.5804
ZINC06162410	-39.6545	-40.5789
ZINC06162410	-36.3802	-40.5789
ZINC00709417	-38.2186	-40.5777
ZINC00709417	-37.6798	-40.5777
ZINC00709417	-36.5767	-40.5777
ZINC00666741	-42.3108	-40.5760
ZINC19889496	-37.1932	-40.5756
ZINC06229108	-48.0896	-40.5749
ZINC22936826	-37.6625	-40.5747
ZINC19789410	-40.3535	-40.5741
ZINC19789410	-37.4749	-40.5741
ZINC04061561	-36.4869	-40.5737
ZINC01109604	-37.4697	-40.5726
ZINC17730072	-37.6151	-40.5714
ZINC17730072	-37.2720	-40.5714
ZINC04062893	-40.0847	-40.5713
ZINC04062893	-38.9892	-40.5713
ZINC09311927	-41.3520	-40.5712
ZINC09311927	-41.1243	-40.5712
ZINC04062811	-37.5396	-40.5695
ZINC08972399	-41.4676	-40.5694
ZINC08972399	-38.6720	-40.5694
ZINC00625864	-37.3419	-40.5668
ZINC06197282	-43.0557	-40.5647
ZINC06197282	-39.2251	-40.5647
ZINC08384233	-40.0199	-40.5641
ZINC05409051	-38.2382	-40.5617
ZINC05409051	-38.2097	-40.5617
ZINC04060266	-37.7472	-40.5612
ZINC04060266	-36.9714	-40.5612
ZINC04060266	-36.6968	-40.5612
ZINC04019613	-40.1101	-40.5607
ZINC04019613	-38.2758	-40.5607
ZINC00726673	-38.8761	-40.5599
ZINC31936864	-40.8249	-40.5588
ZINC04406346	-38.0658	-40.5574
ZINC17118206	-40.0316	-40.5547
ZINC04061399	-37.5738	-40.5533
ZINC02478441	-40.7175	-40.5528
ZINC02478441	-40.0339	-40.5528
ZINC04271954	-37.0440	-40.5528
ZINC19972589	-47.5105	-40.5526
ZINC75280188	-36.4105	-40.5489
ZINC16115364	-37.7818	-40.5480
ZINC06162147	-38.5345	-40.5455
ZINC07602432	-40.6243	-40.5450
ZINC00844226	-40.0187	-40.5447
ZINC00844226	-39.1224	-40.5447
ZINC00844226	-37.7900	-40.5447

ZINC49590070	-44.3093	-40.5424
ZINC04502797	-39.7255	-40.5417
ZINC02173317	-42.3175	-40.5360
ZINC06136887	-39.1094	-40.5357
ZINC06136887	-37.4938	-40.5357
ZINC06136887	-36.7786	-40.5357
ZINC09186711	-40.2242	-40.5354
ZINC00287879	-37.8373	-40.5331
ZINC05409284	-37.5658	-40.5317
ZINC05409284	-36.5915	-40.5317
ZINC00656859	-41.9201	-40.5310
ZINC00626334	-40.6914	-40.5300
ZINC00845264	-37.9501	-40.5285
ZINC08744017	-45.1172	-40.5238
ZINC08744017	-42.5688	-40.5238
ZINC08743191	-37.7183	-40.5230
ZINC08439769	-37.4359	-40.5217
ZINC08439769	-36.9494	-40.5217
ZINC08441268	-38.1523	-40.5215
ZINC04066421	-39.2409	-40.5212
ZINC04066421	-37.2081	-40.5212
ZINC06195839	-37.8252	-40.5210
ZINC19938353	-46.1189	-40.5204
ZINC36646104	-39.1884	-40.5172
ZINC08441633	-38.7641	-40.5162
ZINC08441633	-38.5466	-40.5162
ZINC17180192	-37.2583	-40.5141
ZINC08714272	-44.0197	-40.5135
ZINC06855402	-39.0159	-40.5072
ZINC05014459	-37.4785	-40.5057
ZINC05014459	-37.4314	-40.5057
ZINC06015635	-37.4484	-40.5056
ZINC06015635	-36.6329	-40.5056
ZINC06015635	-36.5783	-40.5056
ZINC06015367	-40.0584	-40.5052
ZINC19773131	-41.0393	-40.5050
ZINC13388480	-38.4833	-40.5047
ZINC19901648	-37.4168	-40.5026
ZINC05488665	-40.6838	-40.5000
ZINC08408146	-38.9325	-40.4993
ZINC04066895	-38.0398	-40.4975
ZINC04066895	-37.8137	-40.4975
ZINC17166865	-39.3309	-40.4967
ZINC17166865	-38.1876	-40.4967
ZINC18037224	-44.8925	-40.4956
ZINC18037224	-43.8862	-40.4956
ZINC09110710	-41.6802	-40.4913
ZINC00702767	-38.9292	-40.4902
ZINC00702426	-41.2758	-40.4899
ZINC00702426	-40.4023	-40.4899
ZINC04649835	-37.0641	-40.4896
ZINC00988833	-38.4154	-40.4887
ZINC06194280	-40.7557	-40.4882
ZINC08416380	-38.5136	-40.4867

ZINC08416380	-37.7028	-40.4867
ZINC08416380	-36.9962	-40.4867
ZINC00792933	-38.9399	-40.4864
ZINC00792933	-37.8102	-40.4864
ZINC08462860	-41.1060	-40.4858
ZINC08462860	-40.5502	-40.4858
ZINC00728225	-39.6474	-40.4855
ZINC00728225	-38.7526	-40.4855
ZINC00728225	-38.6212	-40.4855
ZINC06812899	-39.3981	-40.4844
ZINC04068032	-41.5155	-40.4839
ZINC04068032	-40.2626	-40.4839
ZINC08435258	-41.9742	-40.4837
ZINC08994130	-43.8458	-40.4833
ZINC08994130	-41.7498	-40.4833
ZINC04628354	-37.1838	-40.4811
ZINC04060834	-38.3122	-40.4797
ZINC04060834	-37.6942	-40.4797
ZINC08415725	-36.2658	-40.4779
ZINC06195786	-41.7436	-40.4748
ZINC00726583	-38.6623	-40.4746
ZINC08442012	-42.1220	-40.4744
ZINC06196056	-39.9620	-40.4719
ZINC04067811	-38.6770	-40.4716
ZINC23253846	-40.4446	-40.4704
ZINC08439946	-36.9197	-40.4704
ZINC08424662	-36.7897	-40.4700
ZINC08439846	-39.5757	-40.4697
ZINC08439846	-37.4428	-40.4697
ZINC08439846	-37.3668	-40.4697
ZINC09007984	-44.5639	-40.4685
ZINC09007984	-43.8950	-40.4685
ZINC09007984	-43.7528	-40.4685
ZINC09007984	-43.7009	-40.4685
ZINC06015526	-36.4658	-40.4683
ZINC06162420	-39.8226	-40.4666
ZINC12447605	-44.4575	-40.4645
ZINC02053419	-39.6997	-40.4642
ZINC19909831	-37.3444	-40.4635
ZINC08462149	-41.3567	-40.4612
ZINC02057997	-39.9048	-40.4599
ZINC08440522	-38.4221	-40.4581
ZINC08440522	-37.0687	-40.4581
ZINC04061015	-37.0382	-40.4579
ZINC08450290	-41.1885	-40.4566
ZINC08450290	-40.6874	-40.4566
ZINC08450290	-39.9244	-40.4566
ZINC08415846	-39.7334	-40.4562
ZINC08415846	-39.6198	-40.4562
ZINC00726690	-36.4023	-40.4539
ZINC04060580	-37.3008	-40.4534
ZINC02135501	-42.6332	-40.4506
ZINC04663032	-38.3438	-40.4500
ZINC04663032	-37.4358	-40.4500

ZINC02252328	-39.1465	-40.4465
ZINC08395994	-44.7097	-40.4443
ZINC17719765	-37.5192	-40.4441
ZINC00702474	-39.3069	-40.4430
ZINC01464100	-41.5344	-40.4417
ZINC01464100	-41.0553	-40.4417
ZINC18136442	-48.3204	-40.4381
ZINC18136442	-47.9563	-40.4381
ZINC05800492	-37.1484	-40.4362
ZINC05800492	-36.4384	-40.4362
ZINC08837245	-45.1352	-40.4360
ZINC08837245	-41.5024	-40.4360
ZINC05408490	-45.9700	-40.4348
ZINC09110318	-38.4503	-40.4347
ZINC19832237	-40.8227	-40.4330
ZINC08384154	-42.3797	-40.4301
ZINC08671819	-41.5034	-40.4295
ZINC09089325	-37.8254	-40.4279
ZINC04066591	-38.0634	-40.4264
ZINC04066591	-36.4225	-40.4264
ZINC03903289	-37.3336	-40.4243
ZINC03903289	-36.4292	-40.4243
ZINC19797155	-39.6492	-40.4239
ZINC00205141	-36.6940	-40.4238
ZINC08440781	-37.2218	-40.4230
ZINC04067661	-39.5888	-40.4216
ZINC04067661	-38.2186	-40.4216
ZINC04067350	-39.7571	-40.4213
ZINC06137364	-39.4400	-40.4210
ZINC06148531	-38.6170	-40.4188
ZINC06720040	-37.0341	-40.4188
ZINC18249885	-41.1598	-40.4183
ZINC18249885	-40.3464	-40.4183
ZINC06162365	-36.6066	-40.4177
ZINC08742619	-41.4224	-40.4177
ZINC15228761	-42.0047	-40.4174
ZINC03903284	-38.1546	-40.4162
ZINC03903284	-37.2757	-40.4162
ZINC85389499	-40.9330	-40.4131
ZINC08384508	-43.9742	-40.4120
ZINC00844160	-38.0372	-40.4094
ZINC06195745	-39.1964	-40.4047
ZINC08905396	-41.1684	-40.4037
ZINC08905396	-40.9801	-40.4037
ZINC17264670	-41.5117	-40.4022
ZINC09015458	-39.9796	-40.4009
ZINC02483507	-42.9038	-40.4007
ZINC04065156	-44.6919	-40.3996
ZINC04065156	-44.0134	-40.3996
ZINC00861136	-39.6666	-40.3986
ZINC00861136	-38.8368	-40.3986
ZINC00861136	-38.4785	-40.3986
ZINC02180636	-36.5523	-40.3980
ZINC08406484	-37.5170	-40.3961

ZINC08439970	-42.4872	-40.3933
ZINC18097872	-40.3377	-40.3897
ZINC18097872	-37.9929	-40.3897
ZINC18097872	-37.5649	-40.3897
ZINC00985303	-38.3826	-40.3873
ZINC00865833	-38.3812	-40.3846
ZINC04780889	-37.6113	-40.3843
ZINC04780889	-37.0898	-40.3843
ZINC00726679	-38.1622	-40.3828
ZINC13137025	-37.8340	-40.3815
ZINC04067075	-36.6552	-40.3809
ZINC85406435	-37.5530	-40.3796
ZINC19956629	-39.8495	-40.3784
ZINC08440992	-39.4330	-40.3778
ZINC08437288	-37.7275	-40.3763
ZINC19832198	-39.8216	-40.3759
ZINC01022737	-41.0917	-40.3758
ZINC08439341	-38.4778	-40.3749
ZINC06137540	-40.1510	-40.3746
ZINC08442030	-41.2088	-40.3743
ZINC08440206	-41.7336	-40.3741
ZINC06381236	-39.4122	-40.3711
ZINC06148789	-36.5422	-40.3686
ZINC01019954	-39.6019	-40.3677
ZINC06161958	-38.9633	-40.3670
ZINC06161958	-38.6877	-40.3670
ZINC06196057	-38.4317	-40.3662
ZINC08413383	-36.8582	-40.3661
ZINC04060613	-36.3387	-40.3661
ZINC04062331	-37.8694	-40.3658
ZINC19832188	-40.6459	-40.3650
ZINC08413905	-39.3015	-40.3638
ZINC08413905	-36.7104	-40.3638
ZINC07055225	-40.1730	-40.3637
ZINC00717025	-36.7491	-40.3625
ZINC00865772	-40.3187	-40.3600
ZINC00847283	-37.0085	-40.3595
ZINC00847283	-36.2881	-40.3595
ZINC04065160	-39.5611	-40.3591
ZINC04065160	-38.4919	-40.3591
ZINC08441183	-42.4883	-40.3583
ZINC00781174	-39.1022	-40.3503
ZINC06668083	-38.8175	-40.3493
ZINC13080645	-36.6736	-40.3490
ZINC19358969	-40.9388	-40.3474
ZINC19358969	-40.6388	-40.3474
ZINC00726681	-39.9839	-40.3470
ZINC00726681	-39.6374	-40.3470
ZINC00726681	-38.8485	-40.3470
ZINC01019885	-37.6610	-40.3442
ZINC02135417	-42.9698	-40.3405
ZINC08685001	-38.2858	-40.3404
ZINC08383926	-43.2353	-40.3396
ZINC00721230	-39.6415	-40.3389

ZINC00721230	-38.6317	-40.3389
ZINC06194202	-39.3107	-40.3382
ZINC00689244	-39.2377	-40.3370
ZINC13153887	-38.4000	-40.3362
ZINC13153887	-37.7058	-40.3362
ZINC08439966	-37.0411	-40.3341
ZINC08414791	-39.2150	-40.3312
ZINC08414791	-38.5988	-40.3312
ZINC18127433	-36.4265	-40.3299
ZINC08415555	-37.5545	-40.3296
ZINC08413980	-37.1823	-40.3295
ZINC06197137	-38.1575	-40.3280
ZINC09176747	-43.6775	-40.3261
ZINC08817022	-47.0735	-40.3253
ZINC08817022	-44.8754	-40.3253
ZINC06148627	-37.5686	-40.3249
ZINC08415119	-39.5771	-40.3233
ZINC08415119	-37.7490	-40.3233
ZINC02057299	-41.3597	-40.3215
ZINC18276214	-40.5253	-40.3188
ZINC18276214	-39.4408	-40.3188
ZINC02769156	-36.2807	-40.3182
ZINC00625900	-42.5400	-40.3174
ZINC00625900	-42.2721	-40.3174
ZINC08894844	-40.9387	-40.3169
ZINC08894844	-40.6098	-40.3169
ZINC19816426	-39.9247	-40.3167
ZINC08384250	-39.9663	-40.3156
ZINC00708201	-38.1669	-40.3140
ZINC08817285	-39.4004	-40.3127
ZINC17145905	-43.3833	-40.3109
ZINC17145905	-40.6008	-40.3109
ZINC08413075	-38.3921	-40.3089
ZINC08413075	-38.3586	-40.3089
ZINC08413075	-38.1651	-40.3089
ZINC09460993	-40.9216	-40.3080
ZINC16115454	-42.1556	-40.3057
ZINC16115454	-41.5551	-40.3057
ZINC16115454	-41.0544	-40.3057
ZINC18023697	-44.0941	-40.3055
ZINC08437173	-38.5476	-40.3043
ZINC17166878	-36.8714	-40.3035
ZINC37857518	-41.1209	-40.3024
ZINC19801932	-36.8955	-40.2994
ZINC19909825	-39.3106	-40.2991
ZINC08413215	-38.6068	-40.2982
ZINC08413215	-37.1739	-40.2982
ZINC17139058	-38.8468	-40.2981
ZINC17139058	-36.2814	-40.2981
ZINC00617076	-37.8418	-40.2978
ZINC08414788	-37.1325	-40.2968
ZINC08414788	-36.7379	-40.2968
ZINC18118569	-39.8282	-40.2965
ZINC18118569	-38.0176	-40.2965

ZINC05408367	-41.0797	-40.2920
ZINC04993087	-40.0772	-40.2917
ZINC08441935	-39.8309	-40.2916
ZINC08441935	-37.6357	-40.2916
ZINC09076146	-40.9308	-40.2906
ZINC09076146	-40.3079	-40.2906
ZINC06148615	-40.8517	-40.2887
ZINC06148615	-39.0973	-40.2887
ZINC06148615	-36.9515	-40.2887
ZINC05409040	-37.6648	-40.2871
ZINC05409040	-37.4012	-40.2871
ZINC01464101	-42.4332	-40.2839
ZINC01464101	-40.7888	-40.2839
ZINC19922918	-37.8569	-40.2837
ZINC13569419	-38.3601	-40.2829
ZINC04649727	-40.6248	-40.2772
ZINC04667612	-38.5764	-40.2769
ZINC04360765	-40.9338	-40.2749
ZINC08715663	-38.0605	-40.2747
ZINC08715663	-37.5348	-40.2747
ZINC08450280	-44.7842	-40.2742
ZINC08450280	-42.6088	-40.2742
ZINC09008980	-38.5969	-40.2727
ZINC09008980	-37.8980	-40.2727
ZINC19872220	-41.3058	-40.2707
ZINC04066999	-36.6507	-40.2702
ZINC09070662	-41.2357	-40.2700
ZINC09424996	-41.9873	-40.2678
ZINC08440615	-39.7545	-40.2672
ZINC09463003	-42.5452	-40.2664
ZINC09463003	-40.0106	-40.2664
ZINC20609008	-40.0598	-40.2647
ZINC02910682	-36.4900	-40.2638
ZINC05130802	-36.4916	-40.2603
ZINC08440041	-37.5464	-40.2600
ZINC08440041	-37.2730	-40.2600
ZINC05595688	-37.6798	-40.2592
ZINC17195190	-38.8206	-40.2531
ZINC17195190	-38.3292	-40.2531
ZINC01869323	-38.6497	-40.2531
ZINC19797022	-43.9800	-40.2508
ZINC05634093	-39.5334	-40.2507
ZINC08444006	-45.0630	-40.2485
ZINC85381124	-42.9530	-40.2474
ZINC06197544	-36.5795	-40.2464
ZINC06137173	-39.8670	-40.2462
ZINC06195807	-40.7572	-40.2456
ZINC19911939	-37.8012	-40.2434
ZINC09089038	-37.3327	-40.2426
ZINC08413536	-37.5080	-40.2405
ZINC00703091	-43.9082	-40.2396
ZINC00863253	-37.7332	-40.2387
ZINC19938470	-46.7416	-40.2385
ZINC06195970	-38.8816	-40.2382

ZINC09456062	-42.6662	-40.2375
ZINC00799455	-38.8072	-40.2369
ZINC00799455	-36.4390	-40.2369
ZINC00799455	-36.4025	-40.2369
ZINC06442974	-39.1195	-40.2366
ZINC00656826	-40.1561	-40.2313
ZINC06442946	-37.4196	-40.2295
ZINC06015300	-41.1414	-40.2270
ZINC06015300	-40.1905	-40.2270
ZINC08399037	-38.4049	-40.2259
ZINC08462574	-39.1566	-40.2230
ZINC09424168	-45.0298	-40.2220
ZINC08857867	-38.7637	-40.2211
ZINC06137009	-40.8262	-40.2194
ZINC00726485	-38.7794	-40.2172
ZINC00726485	-37.8408	-40.2172
ZINC00726485	-37.6343	-40.2172
ZINC13231928	-42.9098	-40.2166
ZINC06136915	-41.0440	-40.2166
ZINC13549934	-38.0420	-40.2146
ZINC06015292	-39.5420	-40.2113
ZINC06015292	-38.5499	-40.2113
ZINC00844535	-36.3374	-40.2093
ZINC09177781	-43.8397	-40.2066
ZINC09177781	-41.4765	-40.2066
ZINC18010444	-38.1634	-40.2063
ZINC00853716	-38.7026	-40.2063
ZINC00853716	-38.1047	-40.2063
ZINC22587362	-45.1312	-40.2055
ZINC22587362	-44.9580	-40.2055
ZINC22587362	-44.9000	-40.2055
ZINC22587362	-43.9371	-40.2055
ZINC22587362	-40.7441	-40.2055
ZINC08465627	-37.1339	-40.2047
ZINC13127759	-49.6247	-40.2042
ZINC04067920	-37.9248	-40.2042
ZINC19797095	-40.9114	-40.2015
ZINC00714910	-37.4295	-40.2012
ZINC06195763	-43.2554	-40.1984
ZINC05576768	-40.1135	-40.1957
ZINC18006923	-36.3241	-40.1956
ZINC08440766	-38.9112	-40.1939
ZINC08440766	-38.3241	-40.1939
ZINC04066385	-39.1957	-40.1928
ZINC04059882	-38.2120	-40.1925
ZINC04066696	-38.1650	-40.1924
ZINC04066696	-37.9855	-40.1924
ZINC04066696	-37.0329	-40.1924
ZINC08896124	-38.6377	-40.1919
ZINC08896124	-38.4893	-40.1919
ZINC33707576	-42.7777	-40.1915
ZINC00673855	-37.3238	-40.1892
ZINC06195741	-38.3835	-40.1872
ZINC06195741	-37.5496	-40.1872

ZINC09089051	-40.8050	-40.1864
ZINC06162274	-37.6680	-40.1845
ZINC06162274	-37.4670	-40.1845
ZINC01439632	-37.9321	-40.1805
ZINC01439632	-37.8797	-40.1805
ZINC04123175	-38.4626	-40.1799
ZINC19880702	-39.3503	-40.1797
ZINC19880702	-38.8091	-40.1797
ZINC08399645	-41.3221	-40.1751
ZINC00641706	-40.4569	-40.1731
ZINC04473282	-39.8804	-40.1724
ZINC00702476	-37.9805	-40.1686
ZINC00702476	-36.6121	-40.1686
ZINC08413231	-36.6015	-40.1650
ZINC06197142	-38.8453	-40.1639
ZINC00124032	-37.2952	-40.1631
ZINC08439582	-37.0601	-40.1625
ZINC08439582	-36.8145	-40.1625
ZINC08439582	-36.5907	-40.1625
ZINC00727557	-37.4121	-40.1599
ZINC00690301	-37.1820	-40.1572
ZINC12468152	-41.2494	-40.1571
ZINC04064260	-37.8644	-40.1560
ZINC04064260	-37.2696	-40.1560
ZINC04064260	-37.1668	-40.1560
ZINC12850012	-38.1500	-40.1560
ZINC04565872	-38.7141	-40.1557
ZINC04565872	-38.3546	-40.1557
ZINC13382055	-37.4818	-40.1497
ZINC08914724	-39.9006	-40.1496
ZINC08914724	-39.3175	-40.1496
ZINC00726553	-36.7714	-40.1474
ZINC00702764	-40.5924	-40.1459
ZINC00702764	-37.9804	-40.1459
ZINC30742749	-44.2757	-40.1443
ZINC06197539	-38.4239	-40.1438
ZINC00678555	-40.0445	-40.1437
ZINC09014216	-38.5042	-40.1424
ZINC22075211	-41.5603	-40.1423
ZINC22075211	-38.3243	-40.1423
ZINC16115799	-39.0470	-40.1421
ZINC04780898	-39.5177	-40.1414
ZINC04780898	-37.1715	-40.1414
ZINC01414769	-43.2149	-40.1407
ZINC01414769	-38.1917	-40.1407
ZINC19691223	-37.0650	-40.1402
ZINC20311492	-38.8983	-40.1389
ZINC09274400	-39.0911	-40.1382
ZINC08414802	-39.5954	-40.1313
ZINC08414802	-36.9756	-40.1313
ZINC04066162	-37.7740	-40.1300
ZINC04066162	-37.5292	-40.1300
ZINC06148801	-36.9536	-40.1260
ZINC19872118	-39.5883	-40.1256

ZINC32511581	-37.2069	-40.1255
ZINC08416225	-37.4682	-40.1228
ZINC05490911	-37.6043	-40.1221
ZINC17123871	-45.6032	-40.1211
ZINC06458451	-39.4626	-40.1195
ZINC06458451	-38.5129	-40.1195
ZINC12407461	-44.5737	-40.1192
ZINC00829845	-40.2799	-40.1182
ZINC06148578	-41.3528	-40.1177
ZINC00855631	-38.0069	-40.1166
ZINC00709144	-40.9323	-40.1153
ZINC08439812	-37.5642	-40.1134
ZINC00726512	-38.8449	-40.1131
ZINC00726512	-36.6956	-40.1131
ZINC00726512	-36.5304	-40.1131
ZINC08425666	-38.2305	-40.1099
ZINC08425666	-38.1184	-40.1099
ZINC04908337	-36.7452	-40.1098
ZINC09071666	-42.6581	-40.1093
ZINC09071666	-41.4056	-40.1093
ZINC04626479	-36.6334	-40.1066
ZINC08440560	-38.0410	-40.1061
ZINC00728228	-40.3783	-40.1009
ZINC00728228	-38.4422	-40.1009
ZINC00728228	-37.5420	-40.1009
ZINC04675566	-38.1186	-40.1001
ZINC05951517	-36.6800	-40.0999
ZINC19794404	-36.7922	-40.0981
ZINC13646664	-45.4442	-40.0981
ZINC00853874	-39.7980	-40.0980
ZINC02366334	-47.7902	-40.0979
ZINC04062282	-38.9253	-40.0976
ZINC08441029	-37.1273	-40.0971
ZINC08441029	-36.5971	-40.0971
ZINC36049229	-41.4199	-40.0971
ZINC36049229	-40.2366	-40.0971
ZINC36049229	-39.2980	-40.0971
ZINC05812688	-38.5490	-40.0963
ZINC02755342	-40.6106	-40.0961
ZINC19851617	-42.5822	-40.0943
ZINC06444405	-38.2656	-40.0941
ZINC68755501	-40.4529	-40.0939
ZINC10294868	-38.1325	-40.0929
ZINC00792716	-43.1615	-40.0925
ZINC05695303	-41.2817	-40.0906
ZINC17166900	-45.1854	-40.0903
ZINC17166900	-44.3818	-40.0903
ZINC17166900	-43.9767	-40.0903
ZINC13634078	-40.0931	-40.0901
ZINC13634078	-37.4234	-40.0901
ZINC19926686	-42.1916	-40.0888
ZINC19926686	-41.9728	-40.0888
ZINC19900811	-37.4060	-40.0887
ZINC00717196	-39.2181	-40.0884

ZINC37857517	-39.9957	-40.0882
ZINC37857517	-36.6067	-40.0882
ZINC02055403	-37.6952	-40.0869
ZINC20134982	-45.0533	-40.0865
ZINC09272152	-41.5805	-40.0848
ZINC19938422	-48.7013	-40.0837
ZINC09046913	-37.9423	-40.0829
ZINC04066150	-39.3056	-40.0818
ZINC04066150	-38.5970	-40.0818
ZINC19938637	-39.4916	-40.0816
ZINC09271654	-46.0904	-40.0806
ZINC09271654	-45.1341	-40.0806
ZINC09271654	-43.8874	-40.0806
ZINC09271654	-43.8106	-40.0806
ZINC09271654	-43.2173	-40.0806
ZINC06686621	-43.2026	-40.0800
ZINC00625757	-41.2876	-40.0782
ZINC06195427	-42.6108	-40.0733
ZINC06195427	-40.2426	-40.0733
ZINC17136183	-39.5784	-40.0724
ZINC17136183	-36.9484	-40.0724
ZINC00702736	-38.8543	-40.0710
ZINC00702736	-38.4687	-40.0710
ZINC13786472	-39.1176	-40.0682
ZINC04681740	-38.2271	-40.0679
ZINC36066000	-38.0729	-40.0609
ZINC08439378	-39.5988	-40.0603
ZINC08439378	-39.3572	-40.0603
ZINC04905499	-38.9776	-40.0593
ZINC08435182	-41.2547	-40.0577
ZINC09506873	-41.4895	-40.0568
ZINC09506873	-40.6468	-40.0568
ZINC08440394	-38.8881	-40.0547
ZINC08440394	-37.4450	-40.0547
ZINC05905909	-39.3677	-40.0544
ZINC05905909	-37.5305	-40.0544
ZINC13116388	-38.2142	-40.0517
ZINC13116388	-36.7255	-40.0517
ZINC04067624	-38.0250	-40.0512
ZINC04067624	-37.7690	-40.0512
ZINC04067624	-37.6702	-40.0512
ZINC04693666	-36.6079	-40.0499
ZINC06556697	-40.2548	-40.0493
ZINC08440676	-43.0358	-40.0475
ZINC08440676	-42.8642	-40.0475
ZINC01480037	-41.7657	-40.0455
ZINC01480037	-41.6529	-40.0455
ZINC06149705	-42.1101	-40.0455
ZINC00647043	-42.6046	-40.0448
ZINC19536340	-39.7195	-40.0444
ZINC19372939	-37.0397	-40.0440
ZINC08741070	-41.4939	-40.0437
ZINC08741070	-38.0000	-40.0437
ZINC06015358	-39.4613	-40.0424

ZINC06015358	-36.2825	-40.0424
ZINC04629602	-41.4692	-40.0410
ZINC04629602	-41.3547	-40.0410
ZINC08383811	-43.5469	-40.0405
ZINC04065974	-37.5121	-40.0396
ZINC09311860	-39.9179	-40.0393
ZINC09311860	-39.4642	-40.0393
ZINC04649755	-44.3050	-40.0345
ZINC19938607	-38.2990	-40.0335
ZINC06015298	-39.6007	-40.0319
ZINC06015298	-38.9056	-40.0319
ZINC06015444	-36.5655	-40.0304
ZINC05425015	-42.1159	-40.0298
ZINC00375720	-39.7500	-40.0297
ZINC00674933	-36.7852	-40.0283
ZINC08439838	-39.8910	-40.0262
ZINC08439838	-38.1117	-40.0262
ZINC06162151	-42.5097	-40.0239
ZINC09066839	-46.8184	-40.0210
ZINC09066839	-45.1543	-40.0210
ZINC09066839	-44.6162	-40.0210
ZINC08415334	-41.3038	-40.0209
ZINC08385082	-38.2230	-40.0197
ZINC13285545	-39.4803	-40.0192
ZINC22790716	-37.8392	-40.0191
ZINC22790716	-36.5627	-40.0191
ZINC05615485	-38.9749	-40.0173
ZINC08442102	-38.8308	-40.0149
ZINC08442102	-38.7168	-40.0149
ZINC04032165	-36.9602	-40.0143
ZINC04632291	-36.5475	-40.0113
ZINC04626477	-36.4815	-40.0113
ZINC06195975	-38.4789	-40.0090
ZINC00674256	-37.7706	-40.0083
ZINC00726471	-36.2655	-40.0056
ZINC04626401	-38.1298	-40.0049
ZINC12649160	-39.5696	-40.0029
ZINC04183436	-37.2529	-40.0026
ZINC09057742	-38.5519	-40.0011
ZINC09057742	-37.8170	-40.0011
ZINC08969596	-36.6790	-40.0001
ZINC04014746	-38.5946	-39.9996
ZINC00674251	-37.7382	-39.9980
ZINC08837810	-43.3223	-39.9979
ZINC01148728	-38.6417	-39.9975
ZINC09271308	-41.0223	-39.9969
ZINC08402538	-41.2985	-39.9953
ZINC06136959	-38.0842	-39.9947
ZINC18068387	-36.8903	-39.9943
ZINC08439296	-44.5789	-39.9927
ZINC08453193	-37.7140	-39.9915
ZINC17166816	-37.1773	-39.9915
ZINC17166816	-36.5701	-39.9915
ZINC04068002	-39.1263	-39.9897

ZINC08429950	-39.7713	-39.9892
ZINC09272776	-38.6724	-39.9889
ZINC09272776	-37.1854	-39.9889
ZINC06015543	-37.0041	-39.9878
ZINC09046596	-45.6017	-39.9870
ZINC08913829	-42.0644	-39.9865
ZINC00702610	-42.0366	-39.9847
ZINC00702610	-41.0699	-39.9847
ZINC05175849	-44.4489	-39.9847
ZINC08920507	-42.1922	-39.9839
ZINC13388403	-36.3347	-39.9836
ZINC06195837	-40.2093	-39.9829
ZINC08442128	-40.2680	-39.9821
ZINC13640734	-47.2183	-39.9757
ZINC13640734	-47.0526	-39.9757
ZINC13640734	-44.6387	-39.9757
ZINC13640734	-44.3616	-39.9757
ZINC06015323	-37.3043	-39.9745
ZINC06015323	-37.2310	-39.9745
ZINC06015323	-36.8969	-39.9745
ZINC20567132	-39.2319	-39.9735
ZINC04114801	-39.7928	-39.9728
ZINC04114801	-39.3682	-39.9728
ZINC02473345	-39.4944	-39.9721
ZINC02473345	-38.3719	-39.9721
ZINC02473345	-36.3650	-39.9721
ZINC04167551	-43.4382	-39.9719
ZINC04167551	-41.6095	-39.9719
ZINC07077752	-41.3064	-39.9718
ZINC00674262	-41.1977	-39.9717
ZINC22863056	-40.0203	-39.9688
ZINC22863056	-39.7779	-39.9688
ZINC22863056	-38.4670	-39.9688
ZINC02478439	-38.6080	-39.9666
ZINC02478439	-38.4243	-39.9666
ZINC02478439	-37.6510	-39.9666
ZINC19890004	-38.5679	-39.9659
ZINC00997733	-37.3222	-39.9610
ZINC04459103	-40.8676	-39.9605
ZINC30750470	-46.2518	-39.9591
ZINC18058253	-36.6740	-39.9585
ZINC31907719	-41.1848	-39.9584
ZINC09007506	-41.3347	-39.9583
ZINC06195751	-40.6079	-39.9578
ZINC09275707	-46.6189	-39.9571
ZINC09275707	-46.3331	-39.9571
ZINC09275707	-44.4682	-39.9571
ZINC08442489	-36.3391	-39.9548
ZINC04061194	-38.1254	-39.9539
ZINC04061194	-37.6481	-39.9539
ZINC04061194	-36.9863	-39.9539
ZINC08435661	-43.7356	-39.9512
ZINC06194337	-37.0509	-39.9505
ZINC04312259	-37.9695	-39.9502

ZINC08407912	-43.6560	-39.9497
ZINC08407912	-40.2837	-39.9497
ZINC00708005	-38.3897	-39.9483
ZINC00708005	-37.4663	-39.9483
ZINC00708005	-36.8489	-39.9483
ZINC06015319	-38.7138	-39.9478
ZINC06015319	-37.2626	-39.9478
ZINC06015319	-36.5609	-39.9478
ZINC05313283	-43.8099	-39.9466
ZINC06196441	-37.2081	-39.9452
ZINC08441654	-41.7312	-39.9450
ZINC06195648	-41.3805	-39.9440
ZINC00645588	-38.0375	-39.9438
ZINC08838104	-41.9365	-39.9404
ZINC00726533	-37.2174	-39.9390
ZINC19909560	-42.3850	-39.9386
ZINC09324699	-39.5775	-39.9384
ZINC00821069	-39.9307	-39.9361
ZINC00821069	-39.8418	-39.9361
ZINC00821069	-36.3703	-39.9361
ZINC04065901	-39.6645	-39.9340
ZINC22936485	-36.8256	-39.9332
ZINC06693060	-40.1366	-39.9311
ZINC06693060	-39.8155	-39.9311
ZINC08450714	-37.0423	-39.9305
ZINC19872361	-40.6430	-39.9295
ZINC00848125	-38.4508	-39.9268
ZINC00848125	-37.4841	-39.9268
ZINC00848125	-37.1660	-39.9268
ZINC01471190	-38.7801	-39.9254
ZINC03995655	-37.5356	-39.9240
ZINC03995655	-37.1629	-39.9240
ZINC04059726	-38.8601	-39.9238
ZINC04059726	-38.0055	-39.9238
ZINC13174644	-36.4458	-39.9207
ZINC09071624	-45.6215	-39.9188
ZINC09071624	-44.6680	-39.9188
ZINC09071624	-44.1627	-39.9188
ZINC09071624	-43.8450	-39.9188
ZINC09071624	-43.6477	-39.9188
ZINC08739432	-43.9922	-39.9186
ZINC08739432	-43.5637	-39.9186
ZINC19922799	-40.4488	-39.9174
ZINC06194559	-37.7444	-39.9159
ZINC00728569	-40.9972	-39.9145
ZINC00728569	-40.8626	-39.9145
ZINC00728569	-40.6771	-39.9145
ZINC19827248	-37.2320	-39.9133
ZINC19827248	-36.6145	-39.9133
ZINC09374960	-39.1685	-39.9133
ZINC13601677	-36.2762	-39.9131
ZINC16945409	-39.8498	-39.9124
ZINC08400028	-37.3618	-39.9102
ZINC08400028	-37.2158	-39.9102

ZINC09271466	-37.6862	-39.9062
ZINC09271466	-36.2554	-39.9062
ZINC08440559	-42.0846	-39.9061
ZINC08444549	-37.4116	-39.9038
ZINC01428447	-45.4300	-39.8995
ZINC00073924	-36.3447	-39.8979
ZINC17136284	-37.1470	-39.8979
ZINC04131039	-38.2587	-39.8934
ZINC16115018	-41.3302	-39.8909
ZINC16115018	-40.5474	-39.8909
ZINC08440827	-43.5819	-39.8902
ZINC08440827	-42.4014	-39.8902
ZINC00708202	-36.9580	-39.8869
ZINC00972981	-39.4821	-39.8858
ZINC00972981	-38.6132	-39.8858
ZINC19535821	-36.7437	-39.8817
ZINC19871946	-37.3432	-39.8791
ZINC15913905	-37.4314	-39.8779
ZINC04062757	-36.5430	-39.8768
ZINC08437265	-39.3749	-39.8765
ZINC05577598	-38.4880	-39.8764
ZINC05728160	-38.1221	-39.8753
ZINC09108961	-36.9696	-39.8751
ZINC04067496	-37.5856	-39.8747
ZINC38246114	-39.6624	-39.8744
ZINC05446162	-43.9635	-39.8732
ZINC05445706	-38.1362	-39.8729
ZINC00707960	-38.3700	-39.8712
ZINC00707960	-36.3228	-39.8712
ZINC19802436	-37.5644	-39.8711
ZINC00726527	-38.0312	-39.8705
ZINC05969672	-41.5788	-39.8696
ZINC08438648	-41.0303	-39.8679
ZINC19910897	-37.4525	-39.8678
ZINC00978697	-36.5136	-39.8672
ZINC04387662	-39.3210	-39.8658
ZINC13427514	-44.1366	-39.8649
ZINC13427514	-42.9282	-39.8649
ZINC01029906	-42.1697	-39.8626
ZINC00198068	-37.5152	-39.8622
ZINC09463219	-46.6169	-39.8579
ZINC09463219	-43.6465	-39.8579
ZINC09463219	-43.4881	-39.8579
ZINC09463219	-43.1449	-39.8579
ZINC08440708	-38.0570	-39.8529
ZINC04066564	-37.9467	-39.8528
ZINC04066564	-37.4837	-39.8528
ZINC04065578	-37.3768	-39.8510
ZINC09425132	-40.9319	-39.8501
ZINC09425132	-38.3727	-39.8501
ZINC00823949	-37.1205	-39.8498
ZINC00827954	-39.8549	-39.8481
ZINC08415381	-38.2831	-39.8472
ZINC08817570	-46.6374	-39.8428

ZINC08817570	-42.1139	-39.8428
ZINC08415649	-39.2470	-39.8426
ZINC02370101	-38.2508	-39.8426
ZINC05806948	-39.2749	-39.8414
ZINC05806948	-38.3869	-39.8414
ZINC19360139	-36.7648	-39.8396
ZINC00626287	-38.3852	-39.8386
ZINC08830089	-38.4870	-39.8360
ZINC08721082	-38.2676	-39.8354
ZINC08715664	-39.1007	-39.8345
ZINC08715664	-38.8729	-39.8345
ZINC08424896	-43.8282	-39.8316
ZINC08424896	-43.5334	-39.8316
ZINC08424896	-38.8669	-39.8316
ZINC08385427	-38.8030	-39.8297
ZINC08385427	-38.3675	-39.8297
ZINC17072701	-37.9764	-39.8286
ZINC04065920	-36.5327	-39.8285
ZINC09272445	-38.9573	-39.8254
ZINC09272445	-37.7559	-39.8254
ZINC06144561	-40.1407	-39.8238
ZINC08448133	-43.0703	-39.8233
ZINC08413460	-42.2446	-39.8230
ZINC08413460	-39.6130	-39.8230
ZINC19901312	-41.2804	-39.8217
ZINC10232707	-37.4554	-39.8213
ZINC04042906	-38.7646	-39.8174
ZINC06444764	-42.0173	-39.8152
ZINC06781561	-37.1510	-39.8111
ZINC19972590	-45.9595	-39.8106
ZINC09089064	-37.7442	-39.8092
ZINC20477622	-36.7465	-39.8090
ZINC09292880	-43.4484	-39.8071
ZINC09292880	-39.3477	-39.8071
ZINC01019887	-38.6141	-39.8062
ZINC04068084	-36.8130	-39.8052
ZINC08396304	-40.6454	-39.8040
ZINC19797189	-38.7840	-39.8036
ZINC08397396	-39.5732	-39.8025
ZINC08397396	-38.4844	-39.8025
ZINC00703028	-40.6258	-39.8009
ZINC19881395	-45.8960	-39.8008
ZINC00717018	-37.8723	-39.8007
ZINC00861573	-36.9901	-39.7997
ZINC00861573	-36.6924	-39.7997
ZINC09463369	-42.2839	-39.7977
ZINC00627140	-40.8506	-39.7965
ZINC08437713	-36.4960	-39.7948
ZINC04649003	-38.9727	-39.7916
ZINC08413611	-38.9272	-39.7889
ZINC08413611	-37.7599	-39.7889
ZINC04060668	-37.4059	-39.7887
ZINC08415753	-38.9951	-39.7854
ZINC08415753	-38.0435	-39.7854

ZINC19872122	-36.7898	-39.7853
ZINC18190797	-42.8097	-39.7852
ZINC04417611	-37.7202	-39.7812
ZINC19872344	-38.0748	-39.7806
ZINC08433289	-41.0966	-39.7803
ZINC08433289	-39.7464	-39.7803
ZINC00661581	-38.8774	-39.7796
ZINC22910001	-39.2911	-39.7778
ZINC22910001	-36.3591	-39.7778
ZINC04060810	-38.8839	-39.7771
ZINC00642394	-39.4943	-39.7751
ZINC02088124	-39.8374	-39.7743
ZINC00716222	-36.5768	-39.7733
ZINC09379794	-40.3179	-39.7729
ZINC09379794	-39.3900	-39.7729
ZINC12907702	-38.3429	-39.7711
ZINC08434967	-44.3240	-39.7708
ZINC13388342	-38.8731	-39.7666
ZINC04473281	-41.9403	-39.7646
ZINC12461963	-40.6974	-39.7638
ZINC05446061	-36.5070	-39.7631
ZINC05491433	-38.4259	-39.7608
ZINC00726584	-38.3788	-39.7596
ZINC00726584	-36.8320	-39.7596
ZINC04649844	-38.0464	-39.7590
ZINC08438558	-43.2816	-39.7581
ZINC00726680	-38.2795	-39.7568
ZINC00726680	-37.5921	-39.7568
ZINC04632224	-43.4460	-39.7566
ZINC04632224	-43.1245	-39.7566
ZINC18077919	-42.1358	-39.7563
ZINC06162312	-36.7210	-39.7554
ZINC19872158	-36.6866	-39.7542
ZINC04067576	-37.5537	-39.7499
ZINC04067576	-36.4794	-39.7499
ZINC06162415	-36.3152	-39.7496
ZINC09175607	-39.4814	-39.7493
ZINC00376410	-37.2522	-39.7475
ZINC00673994	-39.1432	-39.7474
ZINC00865790	-40.0211	-39.7474
ZINC04626481	-36.3098	-39.7467
ZINC01264394	-38.3328	-39.7428
ZINC00710233	-37.3684	-39.7424
ZINC08413569	-38.9642	-39.7422
ZINC08413569	-36.7914	-39.7422
ZINC19923551	-37.9771	-39.7416
ZINC06136966	-37.7145	-39.7415
ZINC06445217	-36.5085	-39.7398
ZINC06197394	-40.6640	-39.7391
ZINC06197086	-36.7499	-39.7368
ZINC08438644	-39.3299	-39.7325
ZINC04065843	-39.2098	-39.7310
ZINC06194476	-36.8770	-39.7306
ZINC04043903	-40.4543	-39.7295

ZINC04043903	-40.3085	-39.7295
ZINC08439845	-36.4283	-39.7286
ZINC08439845	-36.3778	-39.7286
ZINC08439490	-37.5506	-39.7253
ZINC04419221	-38.6373	-39.7251
ZINC04395696	-38.3165	-39.7237
ZINC01414751	-45.0245	-39.7233
ZINC08433262	-41.6127	-39.7201
ZINC08396811	-36.6388	-39.7187
ZINC00627976	-38.9512	-39.7185
ZINC18107036	-36.5315	-39.7173
ZINC02088444	-42.5272	-39.7168
ZINC02135644	-42.4843	-39.7125
ZINC08995438	-40.0949	-39.7121
ZINC02102642	-38.2986	-39.7102
ZINC04665813	-36.3035	-39.7098
ZINC00673860	-38.1611	-39.7097
ZINC05917780	-38.6093	-39.7096
ZINC05917780	-37.5300	-39.7096
ZINC06023578	-45.8485	-39.7090
ZINC19938471	-47.0770	-39.7078
ZINC06148669	-38.3489	-39.7078
ZINC00858244	-37.7604	-39.7069
ZINC09011967	-40.4884	-39.7061
ZINC00717892	-39.0578	-39.7057
ZINC00717892	-39.0203	-39.7057
ZINC08444360	-40.7807	-39.7055
ZINC08444360	-39.8411	-39.7055
ZINC09374957	-38.8271	-39.7009
ZINC00804806	-36.6795	-39.7003
ZINC06194525	-37.6521	-39.6960
ZINC10313188	-42.4380	-39.6955
ZINC00727826	-36.5188	-39.6949
ZINC08416238	-37.3689	-39.6948
ZINC08416238	-37.0454	-39.6948
ZINC09271016	-40.7462	-39.6946
ZINC09271016	-40.6857	-39.6946
ZINC19582947	-38.2838	-39.6918
ZINC04114757	-36.7439	-39.6899
ZINC20263642	-47.3578	-39.6857
ZINC02363507	-37.4309	-39.6845
ZINC19900995	-44.3366	-39.6827
ZINC04629606	-41.2053	-39.6796
ZINC04629606	-39.0494	-39.6796
ZINC08893729	-39.4287	-39.6790
ZINC08893729	-39.0806	-39.6790
ZINC05817920	-37.4107	-39.6769
ZINC09471298	-41.9440	-39.6752
ZINC08440465	-44.5203	-39.6745
ZINC08440465	-43.2612	-39.6745
ZINC08439703	-37.2303	-39.6737
ZINC05398018	-37.2557	-39.6733
ZINC15724305	-36.9418	-39.6724
ZINC06137465	-39.4146	-39.6712

ZINC09108960	-36.4106	-39.6709
ZINC22918919	-37.7223	-39.6698
ZINC22918919	-37.3589	-39.6698
ZINC00708157	-37.4730	-39.6695
ZINC08444743	-37.9316	-39.6687
ZINC00702689	-38.8285	-39.6686
ZINC00702689	-37.9050	-39.6686
ZINC09014472	-38.8460	-39.6683
ZINC00821066	-38.0734	-39.6666
ZINC00821066	-36.3382	-39.6666
ZINC08413182	-37.6027	-39.6654
ZINC06148570	-36.9508	-39.6637
ZINC09425007	-41.1838	-39.6616
ZINC09425007	-40.9874	-39.6616
ZINC06137292	-38.4293	-39.6615
ZINC08413218	-37.2721	-39.6601
ZINC08413218	-37.2721	-39.6601
ZINC08413218	-36.5232	-39.6601
ZINC08413218	-36.5232	-39.6601
ZINC00711852	-37.4831	-39.6586
ZINC05728190	-37.1838	-39.6580
ZINC08408080	-39.1117	-39.6574
ZINC00702367	-42.4851	-39.6545
ZINC00702367	-40.2469	-39.6545
ZINC09358361	-37.6798	-39.6525
ZINC09358361	-37.5419	-39.6525
ZINC08440892	-36.8351	-39.6510
ZINC17166903	-38.4333	-39.6492
ZINC19871619	-38.5574	-39.6480
ZINC05445703	-37.4826	-39.6474
ZINC05445703	-36.3421	-39.6474
ZINC09013477	-39.0492	-39.6465
ZINC04665736	-36.3181	-39.6455
ZINC09360194	-41.1496	-39.6443
ZINC09360194	-39.1159	-39.6443
ZINC00726492	-37.7985	-39.6436
ZINC00726492	-37.1682	-39.6436
ZINC04065985	-38.8537	-39.6431
ZINC04065985	-38.3879	-39.6431
ZINC19801744	-36.8734	-39.6422
ZINC08440706	-38.4965	-39.6415
ZINC08440706	-37.4583	-39.6415
ZINC08441056	-38.7931	-39.6407
ZINC08441056	-38.7495	-39.6407
ZINC08441056	-37.8306	-39.6407
ZINC06148605	-37.8134	-39.6388
ZINC02058603	-40.4472	-39.6378
ZINC08413894	-36.6798	-39.6347
ZINC08413894	-36.4409	-39.6347
ZINC19884631	-43.6228	-39.6343
ZINC19884631	-41.2445	-39.6343
ZINC08440783	-38.6092	-39.6342
ZINC08440783	-38.0479	-39.6342
ZINC00823533	-36.3291	-39.6336

ZINC08437175	-41.5262	-39.6324
ZINC02490604	-36.2783	-39.6323
ZINC09274061	-39.7035	-39.6296
ZINC09274061	-36.8515	-39.6296
ZINC12850221	-42.9841	-39.6294
ZINC00978442	-38.7901	-39.6273
ZINC04285170	-38.9323	-39.6263
ZINC04285170	-38.7262	-39.6263
ZINC04044226	-38.0262	-39.6259
ZINC04044226	-36.4252	-39.6259
ZINC08435144	-37.8771	-39.6247
ZINC06407305	-38.3266	-39.6235
ZINC01890423	-43.0182	-39.6219
ZINC06786102	-38.6458	-39.6219
ZINC06136967	-36.7047	-39.6208
ZINC00093114	-36.3497	-39.6202
ZINC00852837	-39.4932	-39.6187
ZINC08442108	-36.5773	-39.6148
ZINC12532006	-39.7310	-39.6146
ZINC00702986	-39.5926	-39.6145
ZINC05483702	-40.3219	-39.6088
ZINC05483702	-36.4249	-39.6088
ZINC12647447	-42.1715	-39.6073
ZINC05490899	-39.0464	-39.6059
ZINC05490899	-36.8914	-39.6059
ZINC04312260	-36.3060	-39.6047
ZINC02511884	-38.1101	-39.6038
ZINC06162454	-38.5471	-39.6035
ZINC19938476	-44.6216	-39.6032
ZINC08440123	-40.1907	-39.6030
ZINC33349154	-37.1246	-39.6029
ZINC06194411	-37.4959	-39.6003
ZINC08396385	-40.4465	-39.5976
ZINC13080653	-38.1190	-39.5943
ZINC13231933	-42.9413	-39.5929
ZINC05409071	-38.1546	-39.5922
ZINC06137524	-36.5143	-39.5918
ZINC08439802	-38.1178	-39.5917
ZINC08439802	-37.7690	-39.5917
ZINC08439802	-36.6659	-39.5917
ZINC04065868	-37.8609	-39.5896
ZINC04065868	-36.2835	-39.5896
ZINC06059935	-39.8162	-39.5889
ZINC06015450	-37.5325	-39.5883
ZINC06015450	-37.1754	-39.5883
ZINC00726645	-38.7766	-39.5877
ZINC00726645	-37.5527	-39.5877
ZINC13230700	-47.4181	-39.5873
ZINC02755341	-38.2641	-39.5866
ZINC19450366	-37.0338	-39.5862
ZINC08439763	-38.1128	-39.5832
ZINC08439763	-37.4217	-39.5832
ZINC08439763	-36.7101	-39.5832
ZINC06420238	-37.0015	-39.5812

ZINC04674279	-40.8914	-39.5800
ZINC04674279	-38.6856	-39.5800
ZINC32654855	-38.7425	-39.5785
ZINC50582379	-39.6661	-39.5781
ZINC02841042	-45.5551	-39.5771
ZINC02841042	-44.1107	-39.5771
ZINC02841042	-42.2320	-39.5771
ZINC06161994	-41.0230	-39.5766
ZINC08439848	-37.5219	-39.5762
ZINC08439848	-37.0611	-39.5762
ZINC08439848	-36.5574	-39.5762
ZINC09356819	-41.2928	-39.5757
ZINC12462720	-38.5318	-39.5757
ZINC00673923	-38.8326	-39.5726
ZINC04075304	-37.4644	-39.5708
ZINC04075304	-37.1642	-39.5708
ZINC06194513	-37.6784	-39.5705
ZINC06194513	-36.7107	-39.5705
ZINC04060850	-39.2875	-39.5699
ZINC00647055	-38.8391	-39.5677
ZINC06162268	-39.9685	-39.5674
ZINC04014867	-39.5259	-39.5672
ZINC01018804	-49.6742	-39.5660
ZINC09380452	-41.3332	-39.5630
ZINC00821072	-38.5196	-39.5592
ZINC00821072	-37.4347	-39.5592
ZINC08450711	-39.0043	-39.5590
ZINC16667395	-46.6776	-39.5568
ZINC00674123	-38.7819	-39.5548
ZINC08440286	-38.6641	-39.5547
ZINC08455446	-42.9012	-39.5530
ZINC02937031	-42.6019	-39.5513
ZINC19782456	-39.5690	-39.5511
ZINC08432507	-40.2839	-39.5494
ZINC04472978	-46.0441	-39.5481
ZINC15229015	-39.3499	-39.5480
ZINC04357471	-38.0544	-39.5473
ZINC04357471	-36.8184	-39.5473
ZINC13468910	-41.8462	-39.5466
ZINC06162414	-37.1599	-39.5466
ZINC15880038	-46.0036	-39.5458
ZINC06015490	-36.8983	-39.5432
ZINC19369721	-42.3874	-39.5432
ZINC12462691	-39.0903	-39.5421
ZINC18100369	-40.9385	-39.5402
ZINC08383933	-42.2325	-39.5398
ZINC02069343	-41.8565	-39.5392
ZINC05014439	-43.7699	-39.5375
ZINC19898770	-40.6130	-39.5363
ZINC06196074	-38.2617	-39.5350
ZINC08413885	-37.7369	-39.5345
ZINC01052726	-50.0170	-39.5336
ZINC19901207	-42.0386	-39.5312
ZINC19901207	-37.9466	-39.5312

ZINC06197487	-37.5985	-39.5297
ZINC04285213	-38.4414	-39.5278
ZINC04285213	-37.1609	-39.5278
ZINC13130664	-39.5710	-39.5272
ZINC04059694	-37.8823	-39.5264
ZINC06197494	-36.6856	-39.5263
ZINC33649115	-38.6055	-39.5255
ZINC06148590	-38.3467	-39.5254
ZINC08706579	-39.2562	-39.5240
ZINC04060687	-36.8844	-39.5234
ZINC04060687	-36.5733	-39.5234
ZINC09046661	-39.5129	-39.5209
ZINC08415856	-37.3816	-39.5202
ZINC02105619	-38.1210	-39.5191
ZINC04312175	-39.0231	-39.5149
ZINC08426161	-36.2913	-39.5125
ZINC08437443	-38.2656	-39.5116
ZINC08437443	-36.8253	-39.5116
ZINC00725682	-37.8384	-39.5115
ZINC00725682	-36.4889	-39.5115
ZINC02088457	-38.2469	-39.5071
ZINC02735164	-44.9893	-39.5047
ZINC09065286	-38.5118	-39.5043
ZINC09065286	-38.4766	-39.5043
ZINC05045024	-42.1012	-39.5015
ZINC05045024	-41.8149	-39.5015
ZINC05045024	-40.6860	-39.5015
ZINC08440096	-42.4924	-39.5014
ZINC08440096	-38.9908	-39.5014
ZINC00703724	-37.1563	-39.4995
ZINC00861100	-36.2830	-39.4985
ZINC00708203	-38.5059	-39.4972
ZINC04285201	-38.4547	-39.4953
ZINC04285201	-37.9606	-39.4953
ZINC05464071	-38.3689	-39.4949
ZINC85388612	-36.5017	-39.4944
ZINC19922640	-38.7735	-39.4907
ZINC04855446	-36.3056	-39.4907
ZINC04065857	-38.9978	-39.4897
ZINC04312258	-37.4367	-39.4871
ZINC01439633	-36.8927	-39.4865
ZINC01439633	-36.8215	-39.4865
ZINC06015296	-39.0651	-39.4848
ZINC06015296	-37.8826	-39.4848
ZINC02474003	-37.0264	-39.4815
ZINC13638626	-48.3442	-39.4804
ZINC13638626	-44.6699	-39.4804
ZINC09110566	-37.3035	-39.4784
ZINC08439839	-36.2979	-39.4753
ZINC16134266	-50.1244	-39.4750
ZINC16134266	-45.0322	-39.4750
ZINC16134266	-44.6520	-39.4750
ZINC16134266	-44.2025	-39.4750
ZINC16134266	-41.4847	-39.4750

ZINC08439240	-40.8795	-39.4721
ZINC09233309	-39.2115	-39.4708
ZINC00866535	-37.3004	-39.4681
ZINC17146424	-40.7712	-39.4678
ZINC17146424	-39.5943	-39.4678
ZINC18010312	-39.1276	-39.4668
ZINC02064528	-40.0193	-39.4650
ZINC04114899	-38.0315	-39.4636
ZINC08996790	-37.0805	-39.4614
ZINC00633077	-43.7224	-39.4572
ZINC00847282	-37.7385	-39.4559
ZINC00847282	-37.2460	-39.4559
ZINC00847282	-37.1355	-39.4559
ZINC00690680	-39.0705	-39.4522
ZINC00853825	-39.4744	-39.4506
ZINC00853825	-36.3312	-39.4506
ZINC08440216	-39.5883	-39.4499
ZINC08440216	-38.0645	-39.4499
ZINC02486656	-38.2547	-39.4433
ZINC08413263	-36.6409	-39.4432
ZINC04066891	-38.7259	-39.4397
ZINC04066891	-38.1118	-39.4397
ZINC19943353	-40.6037	-39.4395
ZINC19943353	-40.0048	-39.4395
ZINC00665929	-39.3205	-39.4393
ZINC08450715	-38.5066	-39.4340
ZINC00717019	-38.3514	-39.4318
ZINC00717019	-37.3910	-39.4318
ZINC08415339	-37.7739	-39.4304
ZINC08415339	-36.4659	-39.4304
ZINC04060262	-36.9638	-39.4280
ZINC04060262	-36.5089	-39.4280
ZINC04060262	-36.3043	-39.4280
ZINC01413484	-42.7528	-39.4242
ZINC01413484	-40.8086	-39.4242
ZINC09240215	-43.9241	-39.4242
ZINC09240215	-43.8413	-39.4242
ZINC09240215	-43.2517	-39.4242
ZINC09240215	-42.5862	-39.4242
ZINC09240215	-42.4272	-39.4242
ZINC09240215	-42.2547	-39.4242
ZINC09240215	-42.1485	-39.4242
ZINC09240215	-41.0321	-39.4242
ZINC09240215	-40.6591	-39.4242
ZINC08397280	-37.5673	-39.4234
ZINC09454435	-42.3804	-39.4218
ZINC85421482	-39.2304	-39.4215
ZINC17885542	-38.1791	-39.4212
ZINC17885542	-37.3195	-39.4212
ZINC19897407	-37.5765	-39.4199
ZINC09012763	-39.1243	-39.4194
ZINC06444475	-39.3284	-39.4184
ZINC17750863	-40.0208	-39.4183
ZINC17750863	-37.0436	-39.4183

ZINC00709146	-38.8473	-39.4176
ZINC00702587	-38.5797	-39.4172
ZINC04066352	-37.4426	-39.4137
ZINC04066352	-36.8121	-39.4137
ZINC08414976	-37.2755	-39.4123
ZINC08440844	-40.9854	-39.4104
ZINC08768812	-38.8310	-39.4069
ZINC06267411	-48.7460	-39.4052
ZINC19881403	-50.2771	-39.4051
ZINC08414881	-36.9271	-39.4050
ZINC06813220	-36.9307	-39.4046
ZINC00726484	-36.6596	-39.4038
ZINC09184894	-37.6784	-39.4037
ZINC13284111	-42.4833	-39.4036
ZINC13284111	-41.8292	-39.4036
ZINC04905530	-37.4241	-39.4031
ZINC18206093	-38.3797	-39.4029
ZINC18206093	-36.7069	-39.4029
ZINC00368096	-38.8470	-39.4029
ZINC09110000	-37.1705	-39.4024
ZINC20192523	-36.4021	-39.4006
ZINC00706598	-36.6475	-39.4004
ZINC00828777	-41.3598	-39.3991
ZINC06137169	-37.2676	-39.3988
ZINC06137169	-37.2265	-39.3988
ZINC04350871	-41.2005	-39.3953
ZINC04350871	-39.6162	-39.3953
ZINC04350871	-38.4879	-39.3953
ZINC06703656	-36.7687	-39.3927
ZINC00674400	-42.3553	-39.3920
ZINC02484843	-39.5570	-39.3920
ZINC00703364	-44.6034	-39.3907
ZINC00703364	-41.2215	-39.3907
ZINC08415491	-38.3832	-39.3892
ZINC08415491	-37.5247	-39.3892
ZINC26844918	-39.8920	-39.3871
ZINC04067497	-38.0809	-39.3868
ZINC04067497	-36.7833	-39.3868
ZINC06446742	-37.3574	-39.3867
ZINC08396361	-43.0664	-39.3844
ZINC08396361	-42.5364	-39.3844
ZINC09236781	-39.2043	-39.3844
ZINC09236781	-38.8362	-39.3844
ZINC19840876	-42.1385	-39.3833
ZINC04068123	-39.6113	-39.3831
ZINC04068123	-38.9264	-39.3831
ZINC04068123	-38.4606	-39.3831
ZINC08900820	-38.0917	-39.3820
ZINC08900820	-37.9130	-39.3820
ZINC00678558	-40.6128	-39.3806
ZINC04649848	-37.7566	-39.3803
ZINC05490617	-37.1271	-39.3799
ZINC00726602	-38.4715	-39.3793
ZINC00726602	-37.4627	-39.3793

ZINC00726602	-36.7741	-39.3793
ZINC15880040	-46.1199	-39.3783
ZINC04060742	-36.5488	-39.3783
ZINC68739728	-39.9868	-39.3782
ZINC18068455	-38.9715	-39.3773
ZINC12138615	-37.0269	-39.3764
ZINC09015459	-39.3330	-39.3718
ZINC06162090	-37.7931	-39.3682
ZINC09008459	-42.4265	-39.3657
ZINC08413054	-38.1686	-39.3646
ZINC05905841	-37.0301	-39.3602
ZINC06196033	-40.9524	-39.3600
ZINC09329990	-37.1833	-39.3557
ZINC08925956	-40.6229	-39.3557
ZINC08925956	-39.1614	-39.3557
ZINC02995417	-48.5733	-39.3555
ZINC06144544	-38.9734	-39.3544
ZINC08425609	-39.5411	-39.3544
ZINC01196771	-38.9719	-39.3536
ZINC01196771	-38.5885	-39.3536
ZINC00726505	-37.4066	-39.3509
ZINC00726505	-36.9435	-39.3509
ZINC00726505	-36.6437	-39.3509
ZINC04062848	-37.2668	-39.3508
ZINC04062848	-37.2610	-39.3508
ZINC06812908	-38.8361	-39.3502
ZINC06812908	-36.8359	-39.3502
ZINC06015508	-37.1896	-39.3475
ZINC00633294	-39.7360	-39.3439
ZINC06782331	-36.7282	-39.3430
ZINC17246309	-38.7050	-39.3430
ZINC17246309	-37.4679	-39.3430
ZINC09374276	-43.1859	-39.3423
ZINC09374276	-38.3773	-39.3423
ZINC08440057	-40.7127	-39.3418
ZINC08440057	-38.4406	-39.3418
ZINC08440057	-36.6633	-39.3418
ZINC06162354	-38.7421	-39.3412
ZINC08816969	-43.7478	-39.3388
ZINC19938301	-41.8460	-39.3382
ZINC00717445	-36.2694	-39.3358
ZINC19938436	-46.5975	-39.3357
ZINC19894471	-37.8718	-39.3331
ZINC02088415	-37.1276	-39.3330
ZINC09243238	-39.7106	-39.3316
ZINC09243238	-37.4599	-39.3316
ZINC08432506	-40.3589	-39.3300
ZINC20390985	-44.1654	-39.3254
ZINC13647585	-45.1833	-39.3241
ZINC17993847	-45.6128	-39.3234
ZINC19923547	-38.1564	-39.3225
ZINC01795873	-38.2802	-39.3213
ZINC09324700	-40.2499	-39.3191
ZINC05943179	-40.5074	-39.3166

ZINC04418346	-38.1282	-39.3164
ZINC04418346	-37.6671	-39.3164
ZINC06137374	-36.6302	-39.3159
ZINC08440359	-37.8093	-39.3155
ZINC08430626	-39.0975	-39.3151
ZINC08430626	-38.8624	-39.3151
ZINC08430626	-37.0110	-39.3151
ZINC22577107	-37.6726	-39.3126
ZINC22577107	-36.6527	-39.3126
ZINC00726517	-40.7829	-39.3112
ZINC00726517	-39.0753	-39.3112
ZINC00633268	-41.1082	-39.3085
ZINC09065062	-39.4292	-39.3079
ZINC17241926	-39.8217	-39.3068
ZINC17241926	-36.7579	-39.3068
ZINC00446273	-38.0691	-39.3061
ZINC09089562	-36.3106	-39.3057
ZINC08441421	-39.5467	-39.3043
ZINC00653848	-38.5760	-39.3006
ZINC08440068	-40.4216	-39.3004
ZINC08440068	-39.8854	-39.3004
ZINC08440035	-36.9264	-39.3003
ZINC08440035	-36.7088	-39.3003
ZINC08607231	-38.8436	-39.2984
ZINC08440801	-39.2847	-39.2983
ZINC20026410	-40.8426	-39.2947
ZINC00641036	-39.2801	-39.2929
ZINC02055263	-39.4072	-39.2911
ZINC12362949	-41.6433	-39.2883
ZINC12615858	-42.9565	-39.2861
ZINC12615858	-42.9089	-39.2861
ZINC00702980	-45.4822	-39.2856
ZINC00702980	-44.5314	-39.2856
ZINC00702980	-44.4968	-39.2856
ZINC08444503	-39.1278	-39.2851
ZINC04062913	-37.6706	-39.2849
ZINC04062913	-37.2809	-39.2849
ZINC00989497	-38.2666	-39.2832
ZINC08905104	-44.5911	-39.2831
ZINC09360327	-36.6126	-39.2805
ZINC05807246	-39.6966	-39.2805
ZINC05807246	-37.4154	-39.2805
ZINC05408624	-40.5732	-39.2773
ZINC04735854	-41.1854	-39.2760
ZINC00710700	-38.5490	-39.2756
ZINC22936522	-40.8368	-39.2746
ZINC01414771	-42.7308	-39.2726
ZINC01414771	-41.8020	-39.2726
ZINC06144546	-41.7408	-39.2726
ZINC00969943	-38.5275	-39.2708
ZINC01213880	-39.6196	-39.2694
ZINC06413637	-38.0741	-39.2690
ZINC05890773	-41.1605	-39.2680
ZINC02896406	-43.5551	-39.2674

ZINC08438646	-41.2235	-39.2646
ZINC09272121	-40.6456	-39.2637
ZINC09272121	-40.2122	-39.2637
ZINC19897647	-36.3529	-39.2632
ZINC04632225	-39.7045	-39.2592
ZINC04632225	-39.6341	-39.2592
ZINC00669616	-41.9481	-39.2567
ZINC19904613	-45.4833	-39.2560
ZINC16115512	-42.2522	-39.2559
ZINC16115512	-41.7057	-39.2559
ZINC16115512	-39.8542	-39.2559
ZINC08426799	-37.8070	-39.2554
ZINC08414955	-36.3859	-39.2539
ZINC00853839	-37.8223	-39.2536
ZINC00853839	-37.7189	-39.2536
ZINC05409067	-37.4196	-39.2527
ZINC05409067	-37.3072	-39.2527
ZINC05409067	-36.8917	-39.2527
ZINC06194458	-39.6405	-39.2523
ZINC17123314	-37.3317	-39.2512
ZINC04068001	-36.6113	-39.2510
ZINC06194479	-37.1201	-39.2506
ZINC06194479	-37.0054	-39.2506
ZINC13388418	-38.1000	-39.2487
ZINC02055229	-37.1100	-39.2478
ZINC04666050	-39.0544	-39.2472
ZINC04067765	-41.2022	-39.2471
ZINC04067765	-40.2271	-39.2471
ZINC04067765	-39.8794	-39.2471
ZINC08437718	-37.8590	-39.2462
ZINC08437718	-37.4690	-39.2462
ZINC08437718	-37.1792	-39.2462
ZINC04681729	-36.6257	-39.2447
ZINC12468073	-39.8081	-39.2446
ZINC06197139	-38.6454	-39.2431
ZINC04059817	-36.9357	-39.2417
ZINC05175869	-42.1775	-39.2404
ZINC05175869	-41.5032	-39.2404
ZINC08397769	-41.1601	-39.2358
ZINC19923131	-41.7368	-39.2341
ZINC08440785	-37.1963	-39.2327
ZINC08440785	-36.9149	-39.2327
ZINC01221131	-46.5791	-39.2325
ZINC00723606	-36.9885	-39.2290
ZINC00844165	-39.2561	-39.2290
ZINC00844165	-37.7252	-39.2290
ZINC04658538	-37.3290	-39.2284
ZINC17730069	-39.3920	-39.2282
ZINC17730069	-38.6393	-39.2282
ZINC08440944	-38.1627	-39.2279
ZINC08440944	-37.4872	-39.2279
ZINC00717893	-38.5023	-39.2268
ZINC00717893	-38.4604	-39.2268
ZINC09088384	-37.3279	-39.2263

ZINC00640980	-39.4373	-39.2250
ZINC08715458	-36.5961	-39.2243
ZINC08413072	-36.9110	-39.2235
ZINC19923565	-36.9821	-39.2228
ZINC18189096	-37.6727	-39.2203
ZINC04884457	-36.3998	-39.2173
ZINC00970966	-44.9056	-39.2153
ZINC00644469	-38.4133	-39.2143
ZINC06197157	-37.8276	-39.2127
ZINC08383685	-44.2507	-39.2106
ZINC01439634	-37.3212	-39.2102
ZINC04646696	-42.6512	-39.2079
ZINC08997026	-38.8040	-39.2060
ZINC09464855	-42.8172	-39.2057
ZINC08383496	-42.3109	-39.2053
ZINC08383496	-41.4484	-39.2053
ZINC02073202	-37.5403	-39.2006
ZINC06196516	-37.6902	-39.1982
ZINC19871721	-38.5912	-39.1957
ZINC06196063	-40.6160	-39.1951
ZINC04061567	-36.5964	-39.1950
ZINC04061567	-36.2906	-39.1950
ZINC09007668	-37.2557	-39.1948
ZINC04648997	-36.3768	-39.1944
ZINC09360154	-40.3489	-39.1938
ZINC06442954	-40.1433	-39.1926
ZINC04424884	-42.1063	-39.1900
ZINC54225151	-36.5254	-39.1900
ZINC08426351	-43.1502	-39.1888
ZINC06136965	-38.2916	-39.1873
ZINC06136965	-37.8960	-39.1873
ZINC06136965	-37.3100	-39.1873
ZINC08383491	-41.7967	-39.1846
ZINC08383491	-40.7072	-39.1846
ZINC08439581	-39.9272	-39.1846
ZINC08439581	-39.0153	-39.1846
ZINC68705091	-38.8910	-39.1835
ZINC09019774	-42.1836	-39.1791
ZINC08462355	-40.1620	-39.1789
ZINC08415476	-37.3922	-39.1784
ZINC09110675	-38.7967	-39.1779
ZINC20193514	-37.4054	-39.1762
ZINC09461131	-39.9293	-39.1734
ZINC09461131	-39.5971	-39.1734
ZINC00999159	-38.9543	-39.1720
ZINC08441572	-45.5532	-39.1710
ZINC08441572	-43.1992	-39.1710
ZINC06197102	-38.1654	-39.1709
ZINC17889198	-40.6191	-39.1696
ZINC09009156	-38.1331	-39.1677
ZINC09009156	-37.8905	-39.1677
ZINC04066494	-36.4433	-39.1671
ZINC08415424	-37.1721	-39.1661
ZINC08415424	-37.1029	-39.1661

ZINC08415424	-36.6993	-39.1661
ZINC08425972	-37.3035	-39.1654
ZINC08425972	-36.6839	-39.1654
ZINC00714634	-38.5391	-39.1645
ZINC00714634	-38.2459	-39.1645
ZINC00714634	-36.9146	-39.1645
ZINC20479867	-37.4150	-39.1641
ZINC00469716	-40.3394	-39.1604
ZINC04061443	-37.3836	-39.1600
ZINC04061443	-37.2461	-39.1600
ZINC08462894	-41.8655	-39.1592
ZINC19376308	-37.8426	-39.1579
ZINC06166133	-38.6394	-39.1567
ZINC08438778	-45.0684	-39.1560
ZINC00993572	-37.7390	-39.1535
ZINC00993572	-37.3469	-39.1535
ZINC19889362	-40.3221	-39.1533
ZINC06242903	-39.0676	-39.1532
ZINC04665714	-37.5429	-39.1484
ZINC06137239	-36.2721	-39.1479
ZINC35287343	-38.1266	-39.1444
ZINC00384727	-43.5412	-39.1441
ZINC19872031	-36.9763	-39.1377
ZINC13566726	-39.8085	-39.1366
ZINC00661550	-38.8974	-39.1362
ZINC13113673	-36.4181	-39.1352
ZINC19871448	-40.7521	-39.1336
ZINC04114958	-36.3875	-39.1310
ZINC05126770	-39.8468	-39.1296
ZINC05126770	-38.2532	-39.1296
ZINC19227943	-37.6310	-39.1276
ZINC19871614	-41.7786	-39.1256
ZINC09232469	-37.5189	-39.1234
ZINC08903236	-39.5934	-39.1225
ZINC08903236	-36.3400	-39.1225
ZINC08694283	-40.6664	-39.1211
ZINC04062216	-36.7882	-39.1192
ZINC04626404	-36.5705	-39.1173
ZINC01889816	-38.9532	-39.1074
ZINC04016761	-40.2841	-39.1060
ZINC04114935	-37.6668	-39.1052
ZINC18084649	-40.3627	-39.1051
ZINC18084649	-39.4464	-39.1051
ZINC00879727	-50.7494	-39.1025
ZINC04063009	-37.8102	-39.1023
ZINC04063009	-36.9200	-39.1023
ZINC04063009	-36.7524	-39.1023
ZINC00681659	-37.4705	-39.1011
ZINC04066961	-38.6712	-39.1011
ZINC08415732	-39.0976	-39.1006
ZINC08415732	-38.4169	-39.1006
ZINC08739000	-43.8313	-39.0968
ZINC08739000	-42.4760	-39.0968
ZINC02072018	-39.7534	-39.0938

ZINC00702428	-44.9157	-39.0914
ZINC00702428	-42.6767	-39.0914
ZINC19881399	-47.5190	-39.0902
ZINC02184907	-40.1494	-39.0897
ZINC06137167	-37.2004	-39.0884
ZINC02055280	-42.2641	-39.0881
ZINC09413162	-38.2911	-39.0860
ZINC09413162	-36.3290	-39.0860
ZINC04473272	-37.6372	-39.0853
ZINC02494957	-37.2304	-39.0847
ZINC00726601	-37.0610	-39.0836
ZINC00726601	-36.5816	-39.0836
ZINC00702732	-41.5706	-39.0829
ZINC00702732	-37.3496	-39.0829
ZINC15923747	-42.5272	-39.0817
ZINC04452951	-37.7494	-39.0809
ZINC04452951	-37.0577	-39.0809
ZINC05062064	-39.2545	-39.0780
ZINC18136853	-38.9320	-39.0772
ZINC18136853	-38.8018	-39.0772
ZINC13111047	-36.5604	-39.0766
ZINC08397430	-40.1874	-39.0762
ZINC09456659	-43.5594	-39.0744
ZINC09456659	-42.1514	-39.0744
ZINC09363614	-38.0638	-39.0742
ZINC19818217	-38.1005	-39.0725
ZINC19818217	-36.9512	-39.0725
ZINC09374512	-40.2820	-39.0725
ZINC04416111	-39.8081	-39.0695
ZINC17027802	-37.8737	-39.0685
ZINC02090338	-36.3632	-39.0682
ZINC06194214	-37.0961	-39.0661
ZINC06137219	-36.8136	-39.0649
ZINC06162355	-37.9226	-39.0637
ZINC06162355	-37.6140	-39.0637
ZINC06786389	-38.4357	-39.0636
ZINC06786389	-38.1338	-39.0636
ZINC08437431	-45.1186	-39.0632
ZINC01413486	-43.3975	-39.0589
ZINC01413486	-41.6214	-39.0589
ZINC00702756	-36.6346	-39.0537
ZINC04060776	-37.0347	-39.0527
ZINC04060776	-36.9443	-39.0527
ZINC08441184	-37.8834	-39.0497
ZINC08441184	-37.8583	-39.0497
ZINC00702699	-40.2383	-39.0491
ZINC00092423	-37.6123	-39.0487
ZINC15974555	-37.5384	-39.0474
ZINC15974555	-36.9458	-39.0474
ZINC15974555	-36.8171	-39.0474
ZINC06162291	-39.0034	-39.0470
ZINC08442429	-37.8609	-39.0462
ZINC09067311	-39.3905	-39.0460
ZINC09067311	-38.2620	-39.0460

ZINC00984601	-36.6944	-39.0445
ZINC20189619	-46.2756	-39.0438
ZINC39932823	-38.5782	-39.0401
ZINC19923154	-43.6891	-39.0397
ZINC17015350	-40.5095	-39.0394
ZINC13388484	-36.4270	-39.0391
ZINC00626103	-37.7073	-39.0352
ZINC04065035	-36.3424	-39.0347
ZINC08438525	-43.2765	-39.0347
ZINC09046579	-41.8488	-39.0346
ZINC06160533	-38.7670	-39.0345
ZINC19802469	-39.7999	-39.0329
ZINC19802469	-38.5864	-39.0329
ZINC01830671	-41.2925	-39.0325
ZINC01145118	-40.2621	-39.0321
ZINC01145118	-38.0943	-39.0321
ZINC20234076	-45.5847	-39.0299
ZINC00645063	-39.6888	-39.0295
ZINC19922796	-40.9317	-39.0251
ZINC19923536	-38.2482	-39.0211
ZINC08385096	-36.2743	-39.0201
ZINC09464726	-41.7076	-39.0197
ZINC09464726	-41.1753	-39.0197
ZINC00803814	-39.2635	-39.0158
ZINC08442504	-45.9401	-39.0155
ZINC08442504	-43.3180	-39.0155
ZINC08450378	-37.4158	-39.0139
ZINC08744138	-41.1632	-39.0100
ZINC08413403	-37.2459	-39.0095
ZINC00811411	-39.6066	-39.0086
ZINC06144060	-45.0277	-39.0077
ZINC15952856	-42.8616	-39.0063
ZINC13116665	-36.9569	-39.0061
ZINC08430361	-37.5479	-39.0045
ZINC00674235	-36.3711	-39.0039
ZINC18077916	-41.0161	-39.0038
ZINC08440712	-36.9474	-39.0025
ZINC00972982	-39.0007	-39.0019
ZINC13127855	-38.1463	-39.0013
ZINC09270919	-38.2378	-39.0003
ZINC08425756	-39.1976	-38.9997
ZINC08918904	-46.1494	-38.9992
ZINC08918904	-43.2843	-38.9992
ZINC00702634	-41.9640	-38.9979
ZINC04736963	-37.2473	-38.9976
ZINC09334096	-45.0405	-38.9976
ZINC13510080	-45.8033	-38.9963
ZINC09085991	-41.7590	-38.9960
ZINC09085991	-40.8346	-38.9960
ZINC04019915	-42.8887	-38.9954
ZINC00726071	-39.9040	-38.9949
ZINC08426034	-43.9864	-38.9937
ZINC06137162	-39.6915	-38.9928
ZINC06137162	-38.7494	-38.9928

ZINC08437169	-41.2399	-38.9928
ZINC00853836	-36.4739	-38.9926
ZINC05595686	-39.2115	-38.9913
ZINC06195790	-39.2885	-38.9913
ZINC19872359	-41.0178	-38.9911
ZINC09042970	-44.3093	-38.9911
ZINC09042970	-40.0460	-38.9911
ZINC07602310	-37.7372	-38.9868
ZINC00702760	-39.0950	-38.9862
ZINC00702760	-38.8867	-38.9862
ZINC09008418	-41.1956	-38.9851
ZINC04418247	-36.8210	-38.9846
ZINC04418247	-36.4240	-38.9846
ZINC09363613	-38.9960	-38.9835
ZINC00678327	-39.0295	-38.9823
ZINC59817709	-37.7002	-38.9820
ZINC19872017	-39.1751	-38.9801
ZINC00678144	-38.0966	-38.9769
ZINC08921248	-40.0571	-38.9757
ZINC02455395	-37.4214	-38.9732
ZINC13756312	-37.6824	-38.9673
ZINC13756312	-36.9552	-38.9673
ZINC20065916	-46.0422	-38.9648
ZINC19860363	-36.9533	-38.9628
ZINC20392273	-42.9952	-38.9628
ZINC05094951	-38.6923	-38.9612
ZINC08400314	-39.1727	-38.9611
ZINC08413079	-37.6259	-38.9599
ZINC00994389	-41.1444	-38.9578
ZINC08415375	-39.9643	-38.9540
ZINC08415375	-36.6118	-38.9540
ZINC05408986	-37.8202	-38.9531
ZINC05408986	-37.5540	-38.9531
ZINC08994132	-40.1377	-38.9515
ZINC08994132	-38.7283	-38.9515
ZINC06195757	-40.4035	-38.9488
ZINC18029565	-37.5176	-38.9472
ZINC18029565	-37.3807	-38.9472
ZINC20262882	-43.1516	-38.9463
ZINC08426406	-37.2915	-38.9453
ZINC01196773	-40.4437	-38.9448
ZINC01196773	-39.0712	-38.9448
ZINC08439534	-43.8247	-38.9438
ZINC08439534	-43.0274	-38.9438
ZINC08440753	-38.8553	-38.9420
ZINC08440753	-38.4640	-38.9420
ZINC19871446	-39.4419	-38.9396
ZINC08439816	-36.8273	-38.9388
ZINC19938538	-46.5846	-38.9372
ZINC36066012	-37.6063	-38.9352
ZINC19368288	-39.8883	-38.9312
ZINC06442942	-37.6320	-38.9310
ZINC09110510	-37.3960	-38.9296
ZINC09329199	-39.1631	-38.9276

ZINC09329199	-38.4701	-38.9276
ZINC19799425	-37.9400	-38.9275
ZINC08986188	-38.8143	-38.9272
ZINC08816710	-38.2680	-38.9270
ZINC04626472	-37.5287	-38.9258
ZINC17162197	-38.7923	-38.9250
ZINC18212644	-38.1883	-38.9247
ZINC02751883	-42.1491	-38.9241
ZINC08383036	-37.7173	-38.9217
ZINC06197155	-37.4490	-38.9211
ZINC01012846	-39.6142	-38.9207
ZINC05481699	-46.5499	-38.9175
ZINC15952857	-45.9955	-38.9130
ZINC08437215	-38.1756	-38.9116
ZINC00644726	-47.5350	-38.9112
ZINC04593047	-40.1836	-38.9105
ZINC09110468	-36.7161	-38.9101
ZINC59817719	-38.9201	-38.9093
ZINC00717029	-36.4635	-38.9086
ZINC19802105	-36.5028	-38.9071
ZINC04041541	-41.0445	-38.9065
ZINC06137026	-38.4683	-38.9054
ZINC09271679	-48.6471	-38.9044
ZINC09271679	-45.4631	-38.9044
ZINC09123114	-42.1962	-38.9043
ZINC09123114	-41.4529	-38.9043
ZINC08439743	-39.0847	-38.9032
ZINC08439743	-38.2480	-38.9032
ZINC05014437	-43.1642	-38.9025
ZINC10313249	-43.3558	-38.8986
ZINC16115293	-38.9485	-38.8954
ZINC00640029	-45.6312	-38.8948
ZINC06162319	-36.3435	-38.8864
ZINC19853446	-39.7696	-38.8854
ZINC08455445	-41.1526	-38.8850
ZINC06197372	-37.2650	-38.8840
ZINC09110445	-39.1340	-38.8833
ZINC08438529	-43.5478	-38.8830
ZINC19582944	-36.6202	-38.8823
ZINC09008748	-44.9509	-38.8806
ZINC09008748	-41.8738	-38.8806
ZINC09008748	-40.7261	-38.8806
ZINC00874241	-45.3434	-38.8772
ZINC02709617	-37.3719	-38.8745
ZINC00360707	-36.7233	-38.8730
ZINC09045851	-44.1570	-38.8723
ZINC04780891	-37.5541	-38.8714
ZINC04780891	-36.7995	-38.8714
ZINC06136861	-38.4210	-38.8713
ZINC18210473	-38.3951	-38.8702
ZINC18210473	-37.1981	-38.8702
ZINC19909569	-39.2600	-38.8696
ZINC02455436	-36.3359	-38.8685
ZINC06059933	-39.9786	-38.8681

ZINC00691596	-37.4102	-38.8659
ZINC01004546	-37.0658	-38.8615
ZINC75279572	-36.6271	-38.8573
ZINC19938386	-47.1695	-38.8568
ZINC00826322	-38.7441	-38.8558
ZINC00628071	-37.8086	-38.8540
ZINC08996688	-38.1251	-38.8527
ZINC09424792	-41.8755	-38.8519
ZINC16734735	-36.7703	-38.8514
ZINC75279573	-37.4747	-38.8511
ZINC02088833	-40.8319	-38.8509
ZINC08440896	-43.2524	-38.8491
ZINC06137013	-36.8933	-38.8466
ZINC06137013	-36.7934	-38.8466
ZINC08410681	-40.4815	-38.8451
ZINC05807016	-38.4182	-38.8448
ZINC05807016	-37.5430	-38.8448
ZINC04060980	-37.6653	-38.8448
ZINC04060980	-37.1142	-38.8448
ZINC04060980	-36.8776	-38.8448
ZINC09414101	-41.3722	-38.8439
ZINC13120289	-42.6970	-38.8439
ZINC04473529	-36.5751	-38.8434
ZINC19872023	-37.6114	-38.8429
ZINC27489714	-41.6210	-38.8418
ZINC06407218	-38.1896	-38.8416
ZINC04662951	-36.8550	-38.8412
ZINC09334897	-37.1892	-38.8399
ZINC06137396	-37.5560	-38.8396
ZINC08444491	-38.4744	-38.8375
ZINC00726594	-37.7513	-38.8359
ZINC00726594	-36.6681	-38.8359
ZINC04065865	-36.8072	-38.8346
ZINC18250483	-37.5573	-38.8344
ZINC00303489	-36.3018	-38.8309
ZINC08384142	-43.1483	-38.8304
ZINC08384142	-41.6204	-38.8304
ZINC05785546	-41.4224	-38.8303
ZINC06194421	-37.0041	-38.8284
ZINC00673997	-37.3170	-38.8271
ZINC19908641	-38.4591	-38.8243
ZINC08416246	-38.8306	-38.8233
ZINC08416246	-38.6516	-38.8233
ZINC08416246	-38.5488	-38.8233
ZINC68706805	-36.2839	-38.8229
ZINC00702741	-43.2445	-38.8160
ZINC00702741	-40.6963	-38.8160
ZINC04646693	-44.6960	-38.8154
ZINC08429882	-38.4955	-38.8146
ZINC08429882	-37.8708	-38.8146
ZINC01108672	-38.9017	-38.8133
ZINC06283004	-42.5922	-38.8114
ZINC06283004	-41.6815	-38.8114
ZINC06283004	-41.0383	-38.8114

ZINC08416124	-37.9601	-38.8111
ZINC19938609	-38.2474	-38.8087
ZINC08444746	-39.7595	-38.8086
ZINC04786048	-36.4106	-38.8077
ZINC07344126	-38.5441	-38.8061
ZINC18152143	-40.1603	-38.8051
ZINC18152143	-38.3572	-38.8051
ZINC10295012	-36.6087	-38.8021
ZINC04066826	-37.3537	-38.8017
ZINC04066826	-36.6786	-38.8017
ZINC00703107	-42.1698	-38.8002
ZINC00703107	-39.3703	-38.8002
ZINC00159244	-37.5952	-38.7980
ZINC09271700	-37.4212	-38.7977
ZINC09271700	-36.8034	-38.7977
ZINC04067396	-38.1748	-38.7976
ZINC06480569	-40.6492	-38.7974
ZINC09015582	-38.3736	-38.7966
ZINC00984533	-38.5271	-38.7918
ZINC03903142	-37.7057	-38.7911
ZINC03903142	-37.1884	-38.7911
ZINC03903142	-37.1714	-38.7911
ZINC00675022	-39.9491	-38.7907
ZINC05408524	-37.8453	-38.7900
ZINC05408524	-37.7918	-38.7900
ZINC00865770	-38.5817	-38.7885
ZINC08904712	-44.2323	-38.7871
ZINC00673976	-36.7024	-38.7852
ZINC01789904	-41.1237	-38.7849
ZINC04067573	-42.3448	-38.7834
ZINC17166807	-36.4899	-38.7792
ZINC02501404	-37.4395	-38.7768
ZINC02750326	-36.3344	-38.7763
ZINC12468775	-41.9348	-38.7752
ZINC06137006	-38.5814	-38.7743
ZINC06137006	-37.9366	-38.7743
ZINC06137006	-37.5231	-38.7743
ZINC04681741	-36.6594	-38.7717
ZINC18084862	-44.1419	-38.7712
ZINC18084862	-43.3596	-38.7712
ZINC00917403	-41.7764	-38.7699
ZINC05409232	-37.7595	-38.7687
ZINC06194134	-36.9951	-38.7645
ZINC20101992	-37.1758	-38.7641
ZINC00723827	-38.5711	-38.7615
ZINC26460505	-48.8997	-38.7594
ZINC09357330	-39.6165	-38.7541
ZINC06492326	-39.1211	-38.7533
ZINC09312127	-41.6019	-38.7529
ZINC19897299	-36.5384	-38.7513
ZINC04114623	-36.7430	-38.7512
ZINC19370703	-39.0499	-38.7510
ZINC05919712	-39.3264	-38.7507
ZINC09374556	-40.6535	-38.7483

ZINC22972107	-36.8168	-38.7464
ZINC06015493	-38.0639	-38.7458
ZINC06015493	-37.6172	-38.7458
ZINC06015493	-37.3247	-38.7458
ZINC01439635	-36.5646	-38.7443
ZINC32573560	-45.8134	-38.7439
ZINC09123633	-37.4578	-38.7427
ZINC02262450	-37.9672	-38.7419
ZINC08433208	-40.0170	-38.7412
ZINC08433208	-39.5848	-38.7412
ZINC09271444	-38.4602	-38.7406
ZINC09271444	-38.1411	-38.7406
ZINC04066896	-37.3657	-38.7367
ZINC04075165	-37.8383	-38.7361
ZINC19938431	-47.4602	-38.7358
ZINC00662949	-37.3592	-38.7358
ZINC08413188	-37.0564	-38.7341
ZINC08384349	-39.7949	-38.7339
ZINC20623824	-38.1738	-38.7289
ZINC05490366	-38.0902	-38.7286
ZINC05490366	-36.7262	-38.7286
ZINC36065978	-37.7573	-38.7283
ZINC06004740	-39.6335	-38.7276
ZINC06137359	-38.1326	-38.7269
ZINC06137359	-36.4290	-38.7269
ZINC04066036	-36.7096	-38.7237
ZINC04060264	-36.9059	-38.7226
ZINC04060264	-36.5913	-38.7226
ZINC08439739	-40.0339	-38.7222
ZINC08439739	-38.3445	-38.7222
ZINC08439739	-38.1207	-38.7222
ZINC00688099	-37.6019	-38.7217
ZINC04062296	-37.2011	-38.7216
ZINC08437271	-40.9019	-38.7193
ZINC18212769	-40.1954	-38.7167
ZINC02455487	-38.8775	-38.7126
ZINC02455487	-37.0601	-38.7126
ZINC01414731	-42.6532	-38.7118
ZINC01414731	-40.9272	-38.7118
ZINC19872248	-42.1559	-38.7108
ZINC08817023	-46.7123	-38.7085
ZINC08817023	-44.4742	-38.7085
ZINC08817023	-44.2776	-38.7085
ZINC08817023	-42.8123	-38.7085
ZINC08996815	-37.1524	-38.7077
ZINC08739259	-42.7816	-38.7072
ZINC08926693	-42.0661	-38.7047
ZINC05782093	-39.8765	-38.7045
ZINC05409054	-36.6908	-38.7014
ZINC09008280	-41.8395	-38.6995
ZINC04714011	-37.0776	-38.6983
ZINC06015308	-39.1013	-38.6944
ZINC06015308	-37.2637	-38.6944
ZINC04044518	-37.6815	-38.6877

ZINC19853491	-40.2005	-38.6846
ZINC08739068	-39.5011	-38.6837
ZINC12406155	-37.2685	-38.6828
ZINC19797125	-37.6446	-38.6814
ZINC00702370	-40.8794	-38.6812
ZINC00702370	-40.2467	-38.6812
ZINC13151297	-41.9559	-38.6811
ZINC13151297	-37.4146	-38.6811
ZINC13151297	-36.9209	-38.6811
ZINC08414945	-38.7716	-38.6802
ZINC05409063	-37.3754	-38.6782
ZINC05409063	-36.9075	-38.6782
ZINC00821067	-42.0166	-38.6781
ZINC00821067	-39.4168	-38.6781
ZINC00821067	-39.3354	-38.6781
ZINC06137302	-38.0613	-38.6768
ZINC04674281	-36.4582	-38.6767
ZINC00471236	-39.3556	-38.6761
ZINC06015507	-36.3384	-38.6758
ZINC09240255	-44.9076	-38.6719
ZINC09240255	-44.8887	-38.6719
ZINC05408519	-39.2097	-38.6709
ZINC05408519	-38.8595	-38.6709
ZINC19831202	-42.6114	-38.6698
ZINC06197548	-36.8237	-38.6696
ZINC18141403	-37.3786	-38.6658
ZINC06149886	-38.9801	-38.6646
ZINC18203694	-42.2335	-38.6605
ZINC08921715	-40.5735	-38.6604
ZINC08914746	-39.2188	-38.6587
ZINC08914746	-39.1305	-38.6587
ZINC05175848	-43.9582	-38.6581
ZINC08430731	-39.0057	-38.6571
ZINC05446334	-36.5069	-38.6569
ZINC04662067	-37.6787	-38.6556
ZINC00821013	-37.6446	-38.6553
ZINC20057668	-44.8101	-38.6544
ZINC04066393	-36.7714	-38.6543
ZINC04737888	-37.5910	-38.6529
ZINC06162387	-38.0879	-38.6529
ZINC00673926	-37.2327	-38.6489
ZINC02058002	-37.0779	-38.6479
ZINC17166886	-37.0890	-38.6467
ZINC01002510	-36.5072	-38.6467
ZINC06015620	-36.7135	-38.6464
ZINC19828009	-39.9834	-38.6463
ZINC06156996	-39.4659	-38.6446
ZINC38246118	-39.0439	-38.6445
ZINC08715456	-36.4264	-38.6427
ZINC08425325	-37.2825	-38.6422
ZINC08425325	-36.4385	-38.6422
ZINC09458158	-43.4985	-38.6412
ZINC09458158	-43.0117	-38.6412
ZINC09013974	-38.2941	-38.6410

ZINC08440305	-37.9996	-38.6403
ZINC19938582	-45.6373	-38.6387
ZINC09380636	-39.0299	-38.6373
ZINC09380636	-37.8384	-38.6373
ZINC08454409	-37.5294	-38.6371
ZINC19642844	-40.8446	-38.6355
ZINC19642844	-40.2807	-38.6355
ZINC16115016	-37.1455	-38.6340
ZINC04780917	-36.4667	-38.6337
ZINC06194589	-38.9393	-38.6329
ZINC06194589	-38.9323	-38.6329
ZINC08442136	-39.3490	-38.6320
ZINC16944377	-45.8797	-38.6278
ZINC16944377	-42.9564	-38.6278
ZINC08435580	-38.2933	-38.6272
ZINC02055126	-38.5462	-38.6269
ZINC06137648	-38.1243	-38.6263
ZINC12648857	-38.4002	-38.6256
ZINC08438749	-37.6277	-38.6253
ZINC08440039	-37.3468	-38.6247
ZINC08440039	-36.4255	-38.6247
ZINC05576825	-40.4968	-38.6232
ZINC04066061	-38.2469	-38.6216
ZINC08438681	-42.1268	-38.6213
ZINC08438681	-40.8403	-38.6213
ZINC36646179	-41.2709	-38.6198
ZINC19801851	-39.4826	-38.6170
ZINC20232422	-46.6723	-38.6162
ZINC08425976	-39.3037	-38.6153
ZINC07601438	-36.3616	-38.6122
ZINC08438566	-39.0571	-38.6110
ZINC08438566	-38.7394	-38.6110
ZINC00781173	-37.9552	-38.6107
ZINC19972591	-45.7137	-38.6104
ZINC02058313	-37.0734	-38.6102
ZINC00726670	-36.5309	-38.6082
ZINC00117170	-36.7962	-38.6058
ZINC00865688	-38.7109	-38.6040
ZINC05806978	-36.5440	-38.6039
ZINC05806978	-36.4704	-38.6039
ZINC19372718	-36.5912	-38.6029
ZINC19797585	-42.0489	-38.5976
ZINC19815581	-36.8850	-38.5943
ZINC06196053	-39.1246	-38.5930
ZINC01795564	-42.7846	-38.5930
ZINC00841901	-39.6388	-38.5912
ZINC00841901	-39.1267	-38.5912
ZINC04629617	-42.0260	-38.5889
ZINC19849996	-41.7985	-38.5882
ZINC00814333	-39.6765	-38.5880
ZINC19909104	-42.3972	-38.5874
ZINC06197138	-36.9500	-38.5869
ZINC13388416	-37.3393	-38.5859
ZINC00673822	-37.2698	-38.5840

ZINC09334505	-45.3632	-38.5837
ZINC09334505	-45.0814	-38.5837
ZINC09334505	-43.5913	-38.5837
ZINC09334505	-42.7476	-38.5837
ZINC09334505	-42.0325	-38.5837
ZINC09349853	-43.1660	-38.5823
ZINC09349853	-38.1173	-38.5823
ZINC33824727	-37.8902	-38.5820
ZINC08437441	-37.2876	-38.5806
ZINC08384656	-39.9158	-38.5805
ZINC09329563	-38.0902	-38.5757
ZINC06194278	-38.4039	-38.5724
ZINC04452614	-38.6419	-38.5710
ZINC12468080	-39.0682	-38.5700
ZINC19908722	-36.6301	-38.5685
ZINC36646218	-41.9191	-38.5683
ZINC00655595	-41.0922	-38.5670
ZINC09462841	-39.9255	-38.5665
ZINC19590277	-37.8274	-38.5663
ZINC08435555	-39.0249	-38.5662
ZINC08413292	-36.4679	-38.5652
ZINC09242664	-47.0044	-38.5650
ZINC08440579	-40.9455	-38.5645
ZINC05998581	-39.3507	-38.5611
ZINC06136852	-39.2776	-38.5589
ZINC08413924	-36.6827	-38.5575
ZINC08396411	-40.6696	-38.5573
ZINC19878899	-37.5279	-38.5569
ZINC19878899	-36.8218	-38.5569
ZINC08440729	-37.6956	-38.5524
ZINC05646068	-40.2304	-38.5519
ZINC08413890	-37.3611	-38.5503
ZINC08715755	-38.6968	-38.5489
ZINC06196107	-39.2714	-38.5449
ZINC06444687	-37.2299	-38.5445
ZINC08439659	-39.3696	-38.5427
ZINC08439659	-36.8527	-38.5427
ZINC15986218	-41.7847	-38.5418
ZINC15986218	-41.5647	-38.5418
ZINC15986218	-39.8722	-38.5418
ZINC15986218	-36.4877	-38.5418
ZINC15986218	-36.4510	-38.5418
ZINC19782400	-36.4009	-38.5405
ZINC12384560	-37.1420	-38.5399
ZINC00646679	-37.5999	-38.5396
ZINC05130505	-42.9268	-38.5383
ZINC19923864	-48.6534	-38.5368
ZINC08384556	-43.3699	-38.5365
ZINC08410672	-39.8149	-38.5321
ZINC06197545	-36.5705	-38.5320
ZINC20052307	-37.6830	-38.5307
ZINC17138283	-44.3637	-38.5295
ZINC19815631	-39.8062	-38.5292
ZINC08414979	-37.7780	-38.5274

ZINC04066325	-38.1686	-38.5261
ZINC13116663	-38.7473	-38.5253
ZINC18096094	-37.4150	-38.5242
ZINC09271364	-42.3283	-38.5240
ZINC06136855	-39.8912	-38.5225
ZINC18169849	-44.6381	-38.5199
ZINC04125959	-40.7586	-38.5166
ZINC04125959	-40.5226	-38.5166
ZINC08817921	-36.7200	-38.5160
ZINC17950006	-42.5700	-38.5147
ZINC00721234	-37.9667	-38.5122
ZINC00721234	-37.4371	-38.5122
ZINC09089037	-36.9754	-38.5103
ZINC19872497	-38.5632	-38.5086
ZINC05409059	-38.3283	-38.5078
ZINC06195761	-38.1765	-38.5067
ZINC06267359	-46.4971	-38.5066
ZINC06195955	-39.4090	-38.5058
ZINC00673746	-37.4330	-38.4997
ZINC04060745	-38.1824	-38.4994
ZINC04060745	-37.9991	-38.4994
ZINC09014101	-36.7611	-38.4992
ZINC08973375	-37.6504	-38.4978
ZINC08437166	-42.0848	-38.4962
ZINC08441631	-38.5277	-38.4961
ZINC05409068	-36.3901	-38.4954
ZINC09007666	-37.6410	-38.4951
ZINC05446080	-37.0275	-38.4949
ZINC05408969	-37.3765	-38.4925
ZINC04659130	-36.5017	-38.4911
ZINC08455009	-44.5253	-38.4900
ZINC09271020	-42.1079	-38.4893
ZINC09271020	-40.7232	-38.4893
ZINC09271020	-40.4858	-38.4893
ZINC05444348	-37.0582	-38.4877
ZINC06194362	-37.4404	-38.4849
ZINC06194362	-36.5762	-38.4849
ZINC07709557	-39.1780	-38.4842
ZINC06015631	-36.9688	-38.4836
ZINC02757439	-41.2515	-38.4821
ZINC01414785	-41.9655	-38.4817
ZINC01414785	-41.8998	-38.4817
ZINC04473008	-38.1477	-38.4774
ZINC08706556	-36.7011	-38.4770
ZINC04130839	-36.3307	-38.4768
ZINC00717667	-36.5069	-38.4764
ZINC09312724	-37.1597	-38.4748
ZINC02740589	-39.2147	-38.4743
ZINC09068302	-39.4586	-38.4741
ZINC09235702	-42.5966	-38.4722
ZINC09235702	-41.6347	-38.4722
ZINC09235702	-41.0691	-38.4722
ZINC09235702	-38.7672	-38.4722
ZINC05490791	-37.1695	-38.4702

ZINC35980676	-38.3117	-38.4701
ZINC06813183	-36.2781	-38.4690
ZINC09354265	-45.4868	-38.4670
ZINC09354265	-44.4130	-38.4670
ZINC19972588	-45.9309	-38.4666
ZINC19894477	-41.1681	-38.4665
ZINC08384248	-40.0247	-38.4663
ZINC15952863	-46.9021	-38.4651
ZINC00855628	-37.5941	-38.4644
ZINC02375440	-38.6387	-38.4614
ZINC18179036	-37.3315	-38.4596
ZINC09353848	-38.3332	-38.4594
ZINC09353848	-37.7355	-38.4594
ZINC04067703	-40.0055	-38.4583
ZINC55568012	-39.2030	-38.4582
ZINC00844158	-36.6891	-38.4577
ZINC06480578	-40.7836	-38.4575
ZINC00710234	-37.1692	-38.4551
ZINC19376307	-36.6884	-38.4546
ZINC13635389	-45.7594	-38.4545
ZINC08415112	-38.2079	-38.4523
ZINC08415112	-37.9544	-38.4523
ZINC08385425	-37.1083	-38.4517
ZINC06015510	-37.0774	-38.4509
ZINC06015510	-36.8309	-38.4509
ZINC06015510	-36.5039	-38.4509
ZINC02064453	-41.1129	-38.4493
ZINC13108676	-48.0208	-38.4480
ZINC08413115	-37.8905	-38.4460
ZINC16948996	-36.7072	-38.4456
ZINC08450319	-36.7881	-38.4432
ZINC13388514	-39.4352	-38.4425
ZINC13388514	-36.5320	-38.4425
ZINC13555358	-39.2472	-38.4420
ZINC04043949	-40.4216	-38.4419
ZINC00829827	-38.6239	-38.4407
ZINC06137399	-36.5712	-38.4398
ZINC22029684	-37.4226	-38.4370
ZINC09046687	-37.2316	-38.4351
ZINC17950249	-36.9555	-38.4341
ZINC00809362	-36.2953	-38.4333
ZINC09329562	-37.4457	-38.4329
ZINC08441308	-36.9780	-38.4321
ZINC08441308	-36.9029	-38.4321
ZINC01101493	-38.2007	-38.4299
ZINC20079954	-37.8340	-38.4277
ZINC36646219	-40.4946	-38.4276
ZINC19535819	-37.5089	-38.4274
ZINC01900391	-46.0348	-38.4257
ZINC08440541	-38.2879	-38.4249
ZINC01414732	-42.7362	-38.4240
ZINC01414732	-39.2054	-38.4240
ZINC04061178	-36.5230	-38.4156
ZINC04061178	-36.3994	-38.4156

ZINC68740976	-40.4714	-38.4154
ZINC19872434	-41.8301	-38.4133
ZINC05687022	-36.7138	-38.4132
ZINC06813255	-39.0437	-38.4105
ZINC08434966	-43.0192	-38.4101
ZINC00726646	-36.3834	-38.4096
ZINC06137358	-37.5986	-38.4093
ZINC06136824	-36.4759	-38.4093
ZINC20589594	-41.0315	-38.4049
ZINC20589594	-39.7766	-38.4049
ZINC02057289	-38.7539	-38.4045
ZINC06621788	-41.3260	-38.4023
ZINC09424208	-39.4936	-38.3986
ZINC00702696	-38.1018	-38.3984
ZINC05396620	-36.7993	-38.3961
ZINC06197364	-37.4372	-38.3941
ZINC05807120	-36.6396	-38.3939
ZINC09241005	-40.6654	-38.3935
ZINC09241005	-40.3233	-38.3935
ZINC01890564	-39.1242	-38.3931
ZINC13646663	-43.7737	-38.3930
ZINC00838163	-39.8689	-38.3880
ZINC08971508	-38.6091	-38.3862
ZINC08817647	-38.0750	-38.3859
ZINC06148747	-36.7652	-38.3858
ZINC08435260	-41.2741	-38.3852
ZINC08439532	-41.4118	-38.3843
ZINC08439532	-40.3489	-38.3843
ZINC08439532	-38.3662	-38.3843
ZINC04044229	-36.6154	-38.3828
ZINC04044229	-36.5564	-38.3828
ZINC19313431	-36.8944	-38.3796
ZINC05490371	-37.5515	-38.3759
ZINC19923543	-37.2913	-38.3752
ZINC08416250	-36.8559	-38.3747
ZINC08462428	-41.6633	-38.3736
ZINC08424354	-37.8656	-38.3682
ZINC05432961	-37.0677	-38.3681
ZINC06137160	-38.7265	-38.3661
ZINC06137160	-38.7172	-38.3661
ZINC06137160	-37.4789	-38.3661
ZINC08444589	-40.0741	-38.3641
ZINC12468919	-37.5989	-38.3588
ZINC19938478	-43.0464	-38.3570
ZINC12507972	-45.0185	-38.3511
ZINC02073201	-36.9023	-38.3501
ZINC05905852	-38.0998	-38.3500
ZINC19313441	-37.9752	-38.3458
ZINC08415323	-38.1852	-38.3454
ZINC09111322	-44.0945	-38.3444
ZINC09111322	-40.6386	-38.3444
ZINC06015499	-39.0893	-38.3439
ZINC08414901	-36.5579	-38.3439
ZINC00703108	-40.8039	-38.3380

ZINC00703108	-40.6894	-38.3380
ZINC04115291	-37.1594	-38.3347
ZINC04115291	-36.7375	-38.3347
ZINC00671321	-47.8068	-38.3342
ZINC05764827	-44.3332	-38.3319
ZINC05764827	-42.2152	-38.3319
ZINC33291602	-39.6972	-38.3317
ZINC20264711	-45.3097	-38.3313
ZINC17967237	-37.4321	-38.3311
ZINC17967237	-36.5634	-38.3311
ZINC09236113	-44.8928	-38.3307
ZINC09236113	-42.3175	-38.3307
ZINC09236113	-41.9255	-38.3307
ZINC09236113	-40.7458	-38.3307
ZINC09236113	-38.9222	-38.3307
ZINC20588749	-41.3731	-38.3261
ZINC03903285	-36.6475	-38.3253
ZINC03903285	-36.3017	-38.3253
ZINC00182551	-37.6731	-38.3253
ZINC06149874	-37.0286	-38.3249
ZINC06149874	-36.7966	-38.3249
ZINC08437383	-37.1351	-38.3246
ZINC00060829	-38.8752	-38.3221
ZINC19923540	-37.0831	-38.3219
ZINC00626194	-36.6293	-38.3211
ZINC00678336	-40.1405	-38.3174
ZINC04065579	-38.4671	-38.3165
ZINC04065579	-37.9834	-38.3165
ZINC04065579	-37.8148	-38.3165
ZINC06161962	-37.9800	-38.3155
ZINC75280057	-41.6544	-38.3151
ZINC19894476	-40.8232	-38.3140
ZINC00708829	-38.6039	-38.3136
ZINC00708829	-37.9933	-38.3136
ZINC00708829	-37.9833	-38.3136
ZINC04113088	-42.7772	-38.3115
ZINC04113088	-40.0319	-38.3115
ZINC04113088	-39.4264	-38.3115
ZINC31903918	-40.5863	-38.3112
ZINC19720764	-36.6812	-38.3103
ZINC32541286	-37.8187	-38.3102
ZINC09360980	-37.0490	-38.3050
ZINC08845064	-42.6164	-38.3047
ZINC06194415	-38.5665	-38.3039
ZINC13285524	-39.3058	-38.3037
ZINC13285524	-36.3113	-38.3037
ZINC09110323	-36.5103	-38.3028
ZINC00678141	-38.2346	-38.3021
ZINC19849832	-36.9297	-38.3021
ZINC08441347	-42.0258	-38.3012
ZINC08424622	-37.8102	-38.3009
ZINC08424622	-37.0538	-38.3009
ZINC19909822	-40.1899	-38.2970
ZINC06194255	-37.8466	-38.2967

ZINC00641565	-37.4789	-38.2956
ZINC06448966	-39.1614	-38.2913
ZINC00726635	-36.2611	-38.2912
ZINC06136968	-37.1196	-38.2871
ZINC13571380	-47.2430	-38.2852
ZINC13571380	-44.3615	-38.2852
ZINC13571380	-42.7564	-38.2852
ZINC05126023	-39.2759	-38.2845
ZINC12647637	-38.3536	-38.2834
ZINC08441238	-37.2196	-38.2812
ZINC06139003	-36.7924	-38.2799
ZINC00645591	-39.0001	-38.2797
ZINC08413387	-37.2939	-38.2787
ZINC09362781	-41.8050	-38.2778
ZINC09362781	-39.6978	-38.2778
ZINC09110529	-37.2937	-38.2776
ZINC08440549	-38.5051	-38.2764
ZINC08440549	-37.4800	-38.2764
ZINC04060650	-36.4467	-38.2728
ZINC06162367	-37.7834	-38.2712
ZINC09015122	-37.4236	-38.2664
ZINC08722868	-46.3901	-38.2650
ZINC08905102	-44.0041	-38.2645
ZINC08905102	-39.7488	-38.2645
ZINC08424620	-38.4734	-38.2643
ZINC08424620	-38.0863	-38.2643
ZINC08424620	-36.9571	-38.2643
ZINC04631658	-40.0496	-38.2625
ZINC08739150	-37.6379	-38.2622
ZINC04312146	-37.7596	-38.2614
ZINC12468483	-48.0670	-38.2588
ZINC75273123	-38.2672	-38.2552
ZINC08430375	-38.2271	-38.2546
ZINC15015783	-38.5384	-38.2543
ZINC15015783	-37.2416	-38.2543
ZINC09354095	-38.1173	-38.2518
ZINC05408982	-37.4618	-38.2498
ZINC05408982	-36.2874	-38.2498
ZINC08387540	-39.0784	-38.2497
ZINC04998966	-36.7955	-38.2492
ZINC17138281	-41.1946	-38.2490
ZINC17138281	-40.9307	-38.2490
ZINC00708205	-36.5954	-38.2489
ZINC00708205	-36.4437	-38.2489
ZINC19868116	-40.0592	-38.2465
ZINC19940206	-43.0959	-38.2462
ZINC19940206	-38.6188	-38.2462
ZINC05910574	-39.1483	-38.2451
ZINC00658071	-46.6568	-38.2445
ZINC04649854	-42.1459	-38.2433
ZINC08415982	-37.4273	-38.2421
ZINC20052304	-37.5456	-38.2413
ZINC08836236	-46.8022	-38.2402
ZINC08441115	-38.7875	-38.2383

ZINC08441115	-38.2488	-38.2383
ZINC09070860	-39.3921	-38.2373
ZINC04350653	-38.7788	-38.2331
ZINC18254442	-37.9998	-38.2325
ZINC18254442	-36.9905	-38.2325
ZINC08438519	-43.6294	-38.2315
ZINC02100246	-36.3078	-38.2308
ZINC00674299	-37.4367	-38.2304
ZINC00520789	-37.6505	-38.2272
ZINC00702726	-37.1276	-38.2262
ZINC08413250	-36.7614	-38.2256
ZINC08462859	-39.3478	-38.2254
ZINC08462859	-38.0603	-38.2254
ZINC04001706	-47.0016	-38.2251
ZINC08743798	-47.2236	-38.2231
ZINC08743798	-41.6433	-38.2231
ZINC08425303	-37.0405	-38.2220
ZINC08425303	-36.9669	-38.2220
ZINC00726678	-36.7221	-38.2212
ZINC08723368	-50.0600	-38.2211
ZINC08413942	-38.3349	-38.2204
ZINC08413942	-36.9395	-38.2204
ZINC00624703	-37.9200	-38.2187
ZINC08408092	-38.8910	-38.2116
ZINC00715098	-38.9119	-38.2106
ZINC00715098	-37.8499	-38.2106
ZINC04063191	-36.2738	-38.2072
ZINC19872015	-39.3225	-38.2052
ZINC00726072	-36.9686	-38.2023
ZINC05338684	-42.7104	-38.2004
ZINC08743986	-39.3201	-38.1998
ZINC08743986	-38.6713	-38.1998
ZINC06015306	-39.3719	-38.1995
ZINC06015306	-37.1521	-38.1995
ZINC06015306	-37.0119	-38.1995
ZINC08433264	-40.9160	-38.1971
ZINC08440034	-37.1282	-38.1971
ZINC06059938	-40.3850	-38.1971
ZINC08441854	-37.3501	-38.1946
ZINC09110446	-38.1664	-38.1940
ZINC19938583	-45.5292	-38.1939
ZINC08440734	-36.8984	-38.1880
ZINC06785178	-41.0326	-38.1828
ZINC02055484	-39.4507	-38.1818
ZINC02055404	-40.7784	-38.1804
ZINC06812806	-37.9647	-38.1796
ZINC06015322	-36.3761	-38.1784
ZINC18152720	-39.1222	-38.1774
ZINC00692146	-39.2834	-38.1771
ZINC00692146	-38.1624	-38.1771
ZINC00692146	-37.6151	-38.1771
ZINC19923545	-37.1935	-38.1770
ZINC04065576	-38.7276	-38.1762
ZINC04065576	-37.8994	-38.1762

ZINC04065576	-36.5837	-38.1762
ZINC06015482	-37.7600	-38.1756
ZINC06162096	-37.9011	-38.1755
ZINC08425421	-39.9156	-38.1753
ZINC08425421	-39.6798	-38.1753
ZINC08414996	-37.8823	-38.1724
ZINC09244014	-39.1749	-38.1723
ZINC09244014	-38.3110	-38.1723
ZINC19926504	-38.2980	-38.1720
ZINC00828776	-41.6011	-38.1718
ZINC12384030	-39.9748	-38.1666
ZINC17167264	-39.6057	-38.1634
ZINC08424899	-39.4780	-38.1619
ZINC06161976	-36.6128	-38.1594
ZINC12631543	-38.9785	-38.1539
ZINC12631543	-37.5272	-38.1539
ZINC00790685	-48.2114	-38.1535
ZINC03901559	-37.3644	-38.1533
ZINC03901559	-36.8352	-38.1533
ZINC06194268	-36.2830	-38.1502
ZINC13771912	-36.2609	-38.1496
ZINC04577800	-36.3172	-38.1468
ZINC05424898	-37.7141	-38.1466
ZINC00726551	-36.5858	-38.1459
ZINC01117667	-45.9990	-38.1458
ZINC17752432	-46.5944	-38.1446
ZINC08437179	-40.9768	-38.1443
ZINC08437721	-38.0503	-38.1437
ZINC04320268	-37.6621	-38.1433
ZINC04065048	-37.7582	-38.1427
ZINC04102451	-38.5782	-38.1418
ZINC04102451	-38.4623	-38.1418
ZINC05817924	-36.6342	-38.1400
ZINC13564472	-42.1285	-38.1394
ZINC13564472	-41.6964	-38.1394
ZINC19938388	-44.2829	-38.1367
ZINC15974468	-37.7842	-38.1361
ZINC08430378	-39.1926	-38.1324
ZINC19790583	-39.4324	-38.1321
ZINC00702820	-39.3104	-38.1321
ZINC06136877	-39.2497	-38.1306
ZINC04068321	-38.3913	-38.1285
ZINC04068321	-37.6957	-38.1285
ZINC02062616	-41.3096	-38.1177
ZINC02062616	-40.9859	-38.1177
ZINC08451996	-37.1391	-38.1172
ZINC02730543	-40.2277	-38.1085
ZINC08716697	-38.4982	-38.1037
ZINC04163018	-38.0397	-38.1026
ZINC09042968	-45.2324	-38.0997
ZINC09042968	-44.1336	-38.0997
ZINC12427919	-39.4394	-38.0990
ZINC06015441	-36.8783	-38.0981
ZINC08904645	-39.3151	-38.0978

ZINC08904645	-39.1749	-38.0978
ZINC00989464	-40.2619	-38.0958
ZINC06137311	-38.1623	-38.0951
ZINC17730154	-36.5660	-38.0927
ZINC08921919	-45.6035	-38.0909
ZINC08921919	-43.8089	-38.0909
ZINC08921919	-43.2559	-38.0909
ZINC09334489	-43.5959	-38.0899
ZINC09334489	-42.5905	-38.0899
ZINC09334489	-42.5515	-38.0899
ZINC09334489	-42.5481	-38.0899
ZINC09334489	-41.5457	-38.0899
ZINC08434813	-38.7248	-38.0889
ZINC06137154	-36.2591	-38.0882
ZINC03664926	-41.4954	-38.0838
ZINC19802316	-37.6380	-38.0826
ZINC06136853	-37.5176	-38.0814
ZINC06138405	-36.2583	-38.0789
ZINC04780838	-37.4460	-38.0786
ZINC01414763	-40.4800	-38.0753
ZINC01414763	-38.1311	-38.0753
ZINC00680524	-38.7790	-38.0737
ZINC18209802	-39.8503	-38.0732
ZINC04626434	-37.2941	-38.0732
ZINC08434994	-43.0770	-38.0726
ZINC00704238	-37.1909	-38.0717
ZINC09360368	-38.5803	-38.0695
ZINC04083014	-39.0414	-38.0689
ZINC04083014	-38.7770	-38.0689
ZINC18188551	-36.3460	-38.0655
ZINC19872130	-38.5225	-38.0606
ZINC09013428	-37.8194	-38.0603
ZINC01019886	-36.5394	-38.0585
ZINC00814363	-37.7571	-38.0584
ZINC04067671	-39.4059	-38.0581
ZINC04067671	-38.8372	-38.0581
ZINC08440634	-44.0371	-38.0521
ZINC00691593	-37.5419	-38.0517
ZINC00821041	-44.2404	-38.0505
ZINC05409049	-37.4054	-38.0467
ZINC05409049	-36.7868	-38.0467
ZINC05409049	-36.4584	-38.0467
ZINC02841793	-36.6302	-38.0466
ZINC13382056	-38.5456	-38.0456
ZINC13382056	-37.7047	-38.0456
ZINC02055393	-36.5831	-38.0433
ZINC02064302	-39.1665	-38.0402
ZINC17241937	-37.0088	-38.0394
ZINC04075151	-36.3923	-38.0357
ZINC09271192	-43.7861	-38.0351
ZINC17995928	-41.8573	-38.0343
ZINC17995928	-39.9654	-38.0343
ZINC19237601	-41.9348	-38.0341
ZINC08438654	-40.2215	-38.0323

ZINC00198325	-36.9383	-38.0323
ZINC05446028	-41.6568	-38.0311
ZINC05446028	-39.0352	-38.0311
ZINC08715758	-39.1048	-38.0311
ZINC08918907	-43.3130	-38.0283
ZINC09009503	-37.1256	-38.0280
ZINC08415292	-37.2328	-38.0221
ZINC04196836	-45.2075	-38.0217
ZINC04067820	-39.9356	-38.0217
ZINC04067820	-39.6585	-38.0217
ZINC04067820	-38.9593	-38.0217
ZINC09329989	-36.5749	-38.0195
ZINC05408971	-36.8887	-38.0188
ZINC05408971	-36.7251	-38.0188
ZINC00716221	-37.4865	-38.0175
ZINC09356619	-39.1296	-38.0160
ZINC75261013	-40.0204	-38.0136
ZINC19852924	-36.6499	-38.0127
ZINC00702443	-37.9434	-38.0090
ZINC68755631	-41.1036	-38.0078
ZINC17162226	-39.2287	-38.0048
ZINC00702734	-37.6955	-38.0046
ZINC00702734	-36.8686	-38.0046
ZINC12362651	-40.1613	-38.0039
ZINC19771364	-39.9460	-38.0039
ZINC19771364	-39.8015	-38.0039
ZINC19371425	-39.1742	-38.0033
ZINC19371425	-38.5637	-38.0033
ZINC17166740	-39.1739	-38.0027
ZINC17166740	-37.5272	-38.0027
ZINC15018435	-36.9681	-37.9980
ZINC08440273	-42.0589	-37.9953
ZINC06136871	-36.8992	-37.9934
ZINC05806977	-38.9306	-37.9865
ZINC36646248	-41.4572	-37.9861
ZINC08918944	-47.5358	-37.9845
ZINC08918944	-45.0174	-37.9845
ZINC08918944	-43.7691	-37.9845
ZINC04285189	-40.2350	-37.9818
ZINC06062236	-38.1954	-37.9805
ZINC17969169	-39.8000	-37.9761
ZINC17969169	-39.0090	-37.9761
ZINC00690060	-37.8186	-37.9759
ZINC06197410	-37.6619	-37.9758
ZINC00631468	-44.2259	-37.9742
ZINC10232709	-36.7209	-37.9725
ZINC03002844	-36.7692	-37.9693
ZINC09123065	-47.6009	-37.9679
ZINC09123065	-44.6915	-37.9679
ZINC09123065	-43.9032	-37.9679
ZINC19815584	-40.4993	-37.9660
ZINC08413282	-38.4978	-37.9648
ZINC02182467	-38.7472	-37.9647
ZINC06782317	-36.8801	-37.9623

ZINC05042263	-37.3981	-37.9607
ZINC15931614	-37.4542	-37.9588
ZINC06160536	-36.4375	-37.9585
ZINC13555009	-45.3482	-37.9570
ZINC09109829	-38.8762	-37.9543
ZINC08384245	-40.7287	-37.9515
ZINC08415670	-39.0063	-37.9513
ZINC08415670	-38.1682	-37.9513
ZINC09065347	-39.5844	-37.9483
ZINC08440543	-41.3280	-37.9450
ZINC19872314	-39.3288	-37.9434
ZINC08997657	-36.9104	-37.9431
ZINC04629614	-40.0797	-37.9423
ZINC04629614	-39.8996	-37.9423
ZINC19889549	-36.3626	-37.9412
ZINC08438544	-36.9761	-37.9411
ZINC17167262	-38.3818	-37.9370
ZINC20367355	-37.9283	-37.9330
ZINC00702964	-45.3196	-37.9326
ZINC00702964	-44.9506	-37.9326
ZINC00702964	-43.7335	-37.9326
ZINC06136912	-38.5793	-37.9300
ZINC06136912	-38.3958	-37.9300
ZINC06136912	-38.3820	-37.9300
ZINC06195835	-37.2616	-37.9291
ZINC08739069	-38.0324	-37.9285
ZINC04629658	-43.0514	-37.9273
ZINC04629658	-42.9317	-37.9273
ZINC00792940	-37.3743	-37.9265
ZINC00792940	-36.4159	-37.9265
ZINC02060835	-38.9525	-37.9254
ZINC06703191	-36.2658	-37.9232
ZINC05488617	-39.6464	-37.9210
ZINC09462516	-43.3317	-37.9181
ZINC09462516	-43.2152	-37.9181
ZINC09462516	-41.6618	-37.9181
ZINC09462516	-41.6381	-37.9181
ZINC09462516	-39.7694	-37.9181
ZINC06148581	-37.7018	-37.9148
ZINC06194510	-37.5778	-37.9110
ZINC04060166	-36.4088	-37.9075
ZINC04060166	-36.3553	-37.9075
ZINC22945566	-39.8104	-37.9066
ZINC22945566	-39.6753	-37.9066
ZINC00728570	-39.4619	-37.9054
ZINC00728570	-37.2844	-37.9054
ZINC18006881	-36.9334	-37.9044
ZINC09089413	-38.3165	-37.9011
ZINC20177138	-36.3854	-37.8972
ZINC00702761	-41.8517	-37.8960
ZINC00702761	-38.8351	-37.8960
ZINC04060134	-36.5819	-37.8959
ZINC08437433	-46.0234	-37.8948
ZINC00829828	-38.8695	-37.8916

ZINC00644719	-42.2557	-37.8913
ZINC20171525	-45.1401	-37.8894
ZINC04615097	-42.7303	-37.8894
ZINC09015123	-38.3089	-37.8883
ZINC37857502	-36.7473	-37.8867
ZINC04114832	-37.7395	-37.8867
ZINC17993864	-40.3430	-37.8857
ZINC17993864	-36.6398	-37.8857
ZINC00809484	-38.8113	-37.8843
ZINC01770702	-37.1674	-37.8788
ZINC00663378	-43.7181	-37.8775
ZINC09008695	-40.3361	-37.8770
ZINC05408978	-38.0317	-37.8733
ZINC05408978	-37.4857	-37.8733
ZINC05408978	-37.0375	-37.8733
ZINC17091751	-36.3875	-37.8731
ZINC00789915	-43.8724	-37.8719
ZINC22941665	-40.4929	-37.8707
ZINC22941665	-39.0680	-37.8707
ZINC06858288	-39.5715	-37.8698
ZINC09374645	-36.8188	-37.8675
ZINC09275527	-41.8307	-37.8668
ZINC09275527	-40.3379	-37.8668
ZINC09108825	-38.1689	-37.8642
ZINC12601802	-43.5992	-37.8618
ZINC08496996	-39.0364	-37.8576
ZINC09007669	-36.7380	-37.8566
ZINC00702485	-41.4957	-37.8561
ZINC09374929	-41.3894	-37.8561
ZINC00725556	-37.3731	-37.8543
ZINC20190806	-43.7394	-37.8538
ZINC36646254	-37.4557	-37.8535
ZINC13045501	-38.1872	-37.8533
ZINC05881804	-46.6211	-37.8520
ZINC00702957	-45.6429	-37.8482
ZINC00702957	-45.2156	-37.8482
ZINC00702957	-43.3310	-37.8482
ZINC36066001	-40.0903	-37.8480
ZINC04065854	-36.8602	-37.8465
ZINC04065854	-36.7962	-37.8465
ZINC04065854	-36.7159	-37.8465
ZINC19871452	-42.5804	-37.8448
ZINC09464862	-43.9137	-37.8432
ZINC01019486	-38.4470	-37.8431
ZINC01019486	-37.8710	-37.8431
ZINC02058149	-36.7707	-37.8430
ZINC08996240	-36.5089	-37.8429
ZINC17166835	-37.9187	-37.8414
ZINC19872178	-37.7640	-37.8413
ZINC09124412	-42.6110	-37.8393
ZINC06137181	-36.4517	-37.8389
ZINC33248651	-40.2966	-37.8379
ZINC19889552	-39.4792	-37.8370
ZINC09110382	-40.3365	-37.8369

ZINC08438532	-42.1836	-37.8353
ZINC08438672	-41.0927	-37.8339
ZINC08438672	-40.2544	-37.8339
ZINC04285158	-40.3357	-37.8314
ZINC06015509	-36.8227	-37.8308
ZINC00876707	-38.1556	-37.8298
ZINC09234828	-36.2807	-37.8289
ZINC17293224	-38.2830	-37.8286
ZINC17293224	-36.5273	-37.8286
ZINC02082416	-41.1873	-37.8272
ZINC00848095	-37.1053	-37.8269
ZINC23114593	-36.7093	-37.8245
ZINC00726555	-37.5632	-37.8241
ZINC00726555	-36.9931	-37.8241
ZINC08425120	-38.2676	-37.8216
ZINC08425120	-36.5926	-37.8216
ZINC17167297	-37.5321	-37.8207
ZINC08437207	-40.8583	-37.8207
ZINC00626281	-41.8557	-37.8147
ZINC06133836	-41.0983	-37.8142
ZINC06554077	-37.5325	-37.8141
ZINC08399513	-40.3014	-37.8138
ZINC00702758	-37.2074	-37.8133
ZINC19881397	-47.7988	-37.8085
ZINC04060831	-37.0467	-37.8081
ZINC04060831	-36.7860	-37.8081
ZINC17167379	-40.5710	-37.8063
ZINC12462718	-36.8204	-37.8021
ZINC12532116	-37.5408	-37.8014
ZINC08426278	-36.9629	-37.8010
ZINC08426278	-36.6647	-37.8010
ZINC00702806	-45.6057	-37.7997
ZINC00702806	-45.3688	-37.7997
ZINC08437161	-39.1508	-37.7993
ZINC06195731	-36.8655	-37.7985
ZINC08774833	-37.2728	-37.7984
ZINC17028657	-37.1089	-37.7980
ZINC00844237	-41.8276	-37.7966
ZINC00844237	-41.6267	-37.7966
ZINC27824835	-36.5484	-37.7941
ZINC08440204	-40.5553	-37.7914
ZINC08438522	-42.3893	-37.7908
ZINC00724042	-37.8941	-37.7898
ZINC00724042	-37.3588	-37.7898
ZINC09463632	-43.7760	-37.7888
ZINC09463632	-42.9415	-37.7888
ZINC15952577	-43.4441	-37.7884
ZINC04066575	-37.2605	-37.7883
ZINC04066575	-37.2279	-37.7883
ZINC00626303	-37.0054	-37.7881
ZINC00381956	-37.3016	-37.7876
ZINC20263645	-44.2796	-37.7870
ZINC01108671	-36.5414	-37.7837
ZINC02490598	-39.6694	-37.7822

ZINC02490598	-37.6142	-37.7822
ZINC04285188	-40.5082	-37.7786
ZINC08463014	-41.0824	-37.7761
ZINC36646230	-38.5759	-37.7740
ZINC19904588	-44.6121	-37.7735
ZINC00661488	-37.1445	-37.7735
ZINC00626857	-36.5783	-37.7706
ZINC12468479	-45.9991	-37.7701
ZINC09007670	-43.7499	-37.7701
ZINC09007670	-39.8280	-37.7701
ZINC13216269	-44.7054	-37.7681
ZINC13216269	-42.7510	-37.7681
ZINC19816085	-37.1856	-37.7667
ZINC00675330	-37.2236	-37.7635
ZINC04660856	-48.1737	-37.7614
ZINC09507828	-41.2036	-37.7606
ZINC09507828	-38.5401	-37.7606
ZINC13607565	-41.0303	-37.7583
ZINC04113026	-36.6710	-37.7571
ZINC04133477	-37.4917	-37.7567
ZINC08740788	-37.7149	-37.7558
ZINC08740788	-37.5536	-37.7558
ZINC08414913	-37.3276	-37.7542
ZINC08440550	-40.6420	-37.7485
ZINC09019773	-36.8839	-37.7419
ZINC09019773	-36.5271	-37.7419
ZINC18127423	-38.4981	-37.7415
ZINC09067671	-38.4397	-37.7400
ZINC09067671	-38.2133	-37.7400
ZINC08397432	-40.3761	-37.7390
ZINC08424556	-37.4615	-37.7373
ZINC04626431	-37.1174	-37.7357
ZINC05919133	-41.4526	-37.7333
ZINC05919133	-37.5125	-37.7333
ZINC05425036	-37.5773	-37.7328
ZINC08411893	-37.0987	-37.7308
ZINC08921646	-41.1819	-37.7305
ZINC04855406	-36.2866	-37.7250
ZINC18082188	-36.7974	-37.7249
ZINC06137305	-38.5702	-37.7241
ZINC06137305	-38.4356	-37.7241
ZINC06137305	-38.3355	-37.7241
ZINC13615167	-38.5776	-37.7233
ZINC08715761	-39.2043	-37.7208
ZINC09012232	-36.7988	-37.7204
ZINC32603668	-39.4417	-37.7189
ZINC01822835	-36.4416	-37.7189
ZINC19923534	-37.1970	-37.7178
ZINC08438568	-40.0304	-37.7175
ZINC08438568	-39.3463	-37.7175
ZINC23114587	-40.6753	-37.7154
ZINC02475453	-36.3065	-37.7147
ZINC34953586	-36.4162	-37.7143
ZINC05998408	-37.9146	-37.7142

ZINC04162260	-37.9967	-37.7135
ZINC08438664	-42.6928	-37.7131
ZINC08438664	-42.3556	-37.7131
ZINC19370707	-37.5498	-37.7097
ZINC01152936	-39.0458	-37.7080
ZINC04652071	-43.9134	-37.7075
ZINC09350764	-40.9839	-37.7062
ZINC09350764	-39.8787	-37.7062
ZINC12849835	-40.9407	-37.7056
ZINC09242680	-42.8996	-37.7043
ZINC56984568	-44.0326	-37.7034
ZINC56984568	-39.1353	-37.7034
ZINC18137016	-38.5778	-37.7013
ZINC00273891	-37.0278	-37.7009
ZINC00646487	-39.7531	-37.7000
ZINC02055401	-36.8184	-37.6992
ZINC00711719	-43.8378	-37.6976
ZINC35982196	-42.3261	-37.6962
ZINC08414815	-37.9011	-37.6945
ZINC00662964	-42.2870	-37.6937
ZINC19853573	-38.2143	-37.6915
ZINC00469760	-36.8184	-37.6910
ZINC19938537	-45.2127	-37.6889
ZINC00082673	-36.5704	-37.6871
ZINC12411584	-42.8443	-37.6865
ZINC08396826	-37.1796	-37.6856
ZINC08396826	-36.3358	-37.6856
ZINC06500218	-40.3773	-37.6838
ZINC08921649	-41.5913	-37.6795
ZINC08921649	-40.8052	-37.6795
ZINC13555544	-40.7803	-37.6760
ZINC10232651	-36.5942	-37.6736
ZINC00661836	-36.5874	-37.6733
ZINC08921716	-40.2940	-37.6711
ZINC08921716	-36.4221	-37.6711
ZINC00726609	-36.7764	-37.6670
ZINC05408947	-36.3204	-37.6657
ZINC04059948	-36.7038	-37.6655
ZINC12650024	-36.2888	-37.6654
ZINC00674936	-36.3576	-37.6646
ZINC09354273	-40.9950	-37.6637
ZINC04730775	-37.4336	-37.6633
ZINC06270586	-44.8369	-37.6627
ZINC04083015	-36.8424	-37.6598
ZINC04001707	-46.2851	-37.6598
ZINC01890479	-38.4114	-37.6593
ZINC06196031	-38.3870	-37.6570
ZINC02100784	-40.8502	-37.6553
ZINC04473064	-40.3418	-37.6540
ZINC00906895	-41.7982	-37.6529
ZINC02072308	-37.3189	-37.6450
ZINC19329520	-37.1359	-37.6443
ZINC13471407	-38.5306	-37.6429
ZINC31936794	-38.6696	-37.6405

ZINC19872397	-38.2725	-37.6297
ZINC08413878	-36.4107	-37.6294
ZINC00826272	-36.6426	-37.6292
ZINC00826272	-36.3394	-37.6292
ZINC02135514	-39.5411	-37.6227
ZINC17143919	-37.1398	-37.6212
ZINC00682767	-36.4373	-37.6201
ZINC06813070	-36.2617	-37.6160
ZINC19802412	-37.7342	-37.6138
ZINC19802412	-37.0698	-37.6138
ZINC08743883	-40.7086	-37.6126
ZINC04631655	-40.9036	-37.6125
ZINC19853454	-41.2315	-37.6116
ZINC19909035	-37.0704	-37.6089
ZINC19909035	-36.9448	-37.6089
ZINC04646462	-39.2293	-37.6061
ZINC04646462	-38.7587	-37.6061
ZINC08838011	-41.6414	-37.6034
ZINC19798264	-38.3072	-37.6025
ZINC08462851	-39.6314	-37.5990
ZINC09270774	-40.8049	-37.5961
ZINC02075786	-40.0074	-37.5954
ZINC00702447	-41.1436	-37.5944
ZINC00702447	-40.6910	-37.5944
ZINC00702447	-40.2887	-37.5944
ZINC00702447	-40.2812	-37.5944
ZINC00702447	-39.5039	-37.5944
ZINC03894917	-38.4297	-37.5934
ZINC00703344	-37.0974	-37.5917
ZINC00703344	-36.4854	-37.5917
ZINC05445723	-38.0801	-37.5886
ZINC00646955	-39.2708	-37.5882
ZINC00704287	-38.2641	-37.5875
ZINC00645592	-44.2353	-37.5842
ZINC09375319	-37.5516	-37.5815
ZINC00027903	-38.4154	-37.5800
ZINC02455437	-36.3873	-37.5781
ZINC02064471	-42.7462	-37.5779
ZINC02088271	-36.6574	-37.5757
ZINC00708156	-36.4796	-37.5729
ZINC19853424	-36.9096	-37.5712
ZINC00729012	-40.2185	-37.5710
ZINC00729012	-39.9109	-37.5710
ZINC00729012	-39.8377	-37.5710
ZINC06553425	-40.2945	-37.5709
ZINC03833270	-37.9793	-37.5695
ZINC03833270	-37.9639	-37.5695
ZINC20308662	-45.4140	-37.5686
ZINC19892767	-38.5170	-37.5666
ZINC04067919	-36.6757	-37.5662
ZINC09461226	-41.5511	-37.5621
ZINC09461226	-40.5668	-37.5621
ZINC08427635	-38.3600	-37.5611
ZINC00723862	-40.5663	-37.5594

ZINC09363627	-36.4477	-37.5574
ZINC05764264	-43.6121	-37.5537
ZINC08438679	-42.2179	-37.5535
ZINC08438679	-37.6342	-37.5535
ZINC06015504	-37.7499	-37.5530
ZINC08385561	-46.4475	-37.5528
ZINC05491258	-36.3500	-37.5500
ZINC05294900	-43.1608	-37.5467
ZINC09243349	-41.8034	-37.5467
ZINC18212008	-41.0229	-37.5463
ZINC18212008	-37.8591	-37.5463
ZINC00865834	-36.9239	-37.5458
ZINC00647151	-37.7634	-37.5452
ZINC05445188	-39.4548	-37.5445
ZINC68755494	-39.7092	-37.5443
ZINC13152760	-37.6390	-37.5424
ZINC08396662	-46.6180	-37.5382
ZINC09311859	-40.3806	-37.5371
ZINC01162373	-38.1657	-37.5370
ZINC01162373	-36.4012	-37.5370
ZINC20477781	-36.8784	-37.5370
ZINC00984566	-36.2718	-37.5354
ZINC19904269	-39.1517	-37.5328
ZINC04998583	-36.7392	-37.5317
ZINC06148611	-38.0543	-37.5314
ZINC06148611	-37.1348	-37.5314
ZINC19923853	-41.4118	-37.5267
ZINC15880039	-44.3227	-37.5263
ZINC02486588	-40.6192	-37.5246
ZINC08439809	-37.0486	-37.5217
ZINC08439809	-36.8867	-37.5217
ZINC00870905	-38.5097	-37.5215
ZINC06161970	-39.2560	-37.5165
ZINC04062162	-36.3101	-37.5137
ZINC18043105	-36.8285	-37.5115
ZINC02060819	-37.4919	-37.5093
ZINC19840868	-36.7196	-37.5089
ZINC00836256	-39.5422	-37.5059
ZINC00836256	-38.3119	-37.5059
ZINC06194130	-36.5289	-37.5053
ZINC00674911	-37.2895	-37.5001
ZINC00884697	-41.0064	-37.4999
ZINC55436394	-39.6012	-37.4964
ZINC02082127	-43.2421	-37.4927
ZINC06148729	-36.4916	-37.4894
ZINC12342772	-38.8453	-37.4888
ZINC08424478	-41.0607	-37.4870
ZINC00034944	-37.0815	-37.4868
ZINC09160475	-38.8737	-37.4801
ZINC19923576	-36.2859	-37.4794
ZINC06137110	-41.0542	-37.4790
ZINC06137110	-40.2577	-37.4790
ZINC06137110	-40.2476	-37.4790
ZINC06194139	-36.3117	-37.4767

ZINC19802319	-36.3171	-37.4767
ZINC00726604	-37.8925	-37.4732
ZINC13130995	-38.5432	-37.4719
ZINC00727718	-37.4492	-37.4714
ZINC08437193	-38.3042	-37.4660
ZINC00823668	-37.3722	-37.4596
ZINC08817142	-36.5778	-37.4549
ZINC08385486	-49.7071	-37.4463
ZINC19368564	-39.8587	-37.4449
ZINC13465055	-39.3241	-37.4445
ZINC36646171	-39.0180	-37.4421
ZINC04067561	-38.0429	-37.4420
ZINC04067561	-37.5465	-37.4420
ZINC03901455	-38.2543	-37.4355
ZINC03901455	-36.3173	-37.4355
ZINC18085974	-37.1183	-37.4297
ZINC09462061	-39.2689	-37.4295
ZINC05446160	-43.3053	-37.4281
ZINC06196084	-39.1097	-37.4278
ZINC17195192	-38.0427	-37.4260
ZINC00067635	-36.9120	-37.4241
ZINC06195971	-36.2947	-37.4230
ZINC04816022	-41.9998	-37.4204
ZINC08997393	-42.9700	-37.4202
ZINC08997393	-39.9816	-37.4202
ZINC04123561	-37.5220	-37.4178
ZINC19370911	-37.7144	-37.4176
ZINC04170795	-36.5084	-37.4169
ZINC13756953	-37.3805	-37.4138
ZINC19872371	-37.1400	-37.4132
ZINC18249558	-39.3407	-37.4123
ZINC18249558	-38.7542	-37.4123
ZINC18249558	-38.6030	-37.4123
ZINC18249558	-38.1374	-37.4123
ZINC00844247	-40.9596	-37.4119
ZINC00844247	-40.0939	-37.4119
ZINC00844247	-39.6489	-37.4119
ZINC00725683	-36.9126	-37.4114
ZINC09235694	-42.7767	-37.4114
ZINC09235694	-42.2646	-37.4114
ZINC09235694	-41.3200	-37.4114
ZINC09176905	-36.9362	-37.4108
ZINC08717031	-37.5344	-37.4091
ZINC16944680	-44.3980	-37.4079
ZINC16944680	-43.8063	-37.4079
ZINC18275895	-38.0632	-37.4066
ZINC18275895	-37.4619	-37.4066
ZINC18275895	-37.0140	-37.4066
ZINC08442137	-37.5634	-37.4066
ZINC18211562	-39.0262	-37.4063
ZINC09271998	-39.3634	-37.4004
ZINC02492032	-36.9151	-37.4000
ZINC18060306	-38.9595	-37.3923
ZINC20263914	-44.1054	-37.3920

ZINC00629694	-42.9263	-37.3911
ZINC19370619	-36.5698	-37.3887
ZINC05764258	-43.8611	-37.3877
ZINC08441430	-36.3933	-37.3858
ZINC08413510	-36.8673	-37.3828
ZINC09271507	-36.8338	-37.3741
ZINC19938606	-38.3858	-37.3701
ZINC04472988	-37.9427	-37.3676
ZINC08444744	-37.1734	-37.3670
ZINC00627165	-37.0603	-37.3666
ZINC19898484	-39.1155	-37.3615
ZINC05446156	-43.3603	-37.3593
ZINC00986239	-36.8775	-37.3566
ZINC00702951	-46.1177	-37.3550
ZINC00702951	-43.7201	-37.3550
ZINC00702951	-42.9901	-37.3550
ZINC01010355	-37.6575	-37.3549
ZINC01020258	-40.5720	-37.3546
ZINC01020258	-39.1266	-37.3546
ZINC19872027	-36.7125	-37.3516
ZINC00823690	-36.6380	-37.3481
ZINC04955781	-40.8139	-37.3465
ZINC02088458	-36.9530	-37.3444
ZINC04210600	-36.5322	-37.3415
ZINC08440502	-41.5430	-37.3411
ZINC19210463	-38.7702	-37.3363
ZINC00644531	-41.4007	-37.3348
ZINC05489413	-36.4238	-37.3343
ZINC09271191	-39.1491	-37.3331
ZINC09271191	-38.6929	-37.3331
ZINC19852681	-39.2947	-37.3329
ZINC08437242	-39.8938	-37.3319
ZINC04075255	-38.2410	-37.3308
ZINC05408891	-39.3778	-37.3270
ZINC04017079	-39.1550	-37.3255
ZINC04017079	-38.4602	-37.3255
ZINC04017079	-36.4344	-37.3255
ZINC04855356	-36.3804	-37.3204
ZINC05928972	-41.0044	-37.3162
ZINC05928972	-39.7551	-37.3162
ZINC04387658	-37.3072	-37.3157
ZINC09065439	-37.4834	-37.3152
ZINC00645288	-43.0679	-37.3151
ZINC12757680	-43.5718	-37.3148
ZINC12757680	-41.3397	-37.3148
ZINC00687278	-41.2681	-37.3146
ZINC05800557	-36.4604	-37.3126
ZINC08433273	-40.6079	-37.3121
ZINC00703052	-40.7633	-37.3096
ZINC00703052	-40.5759	-37.3096
ZINC08441818	-36.3172	-37.3090
ZINC04957252	-46.4212	-37.3085
ZINC36053440	-39.6575	-37.3079
ZINC18179894	-38.1844	-37.3021

ZINC18179894	-37.5886	-37.3021
ZINC18179894	-36.9581	-37.3021
ZINC18179894	-36.7636	-37.3021
ZINC05014457	-37.0124	-37.2976
ZINC00626167	-37.1688	-37.2962
ZINC06195501	-37.0001	-37.2944
ZINC09350326	-37.4264	-37.2941
ZINC08439573	-44.7083	-37.2884
ZINC08439573	-43.0681	-37.2884
ZINC19897640	-37.5233	-37.2868
ZINC05929514	-45.3509	-37.2861
ZINC13633971	-38.0422	-37.2800
ZINC13633971	-37.7999	-37.2800
ZINC18037299	-39.8004	-37.2781
ZINC18037299	-38.1883	-37.2781
ZINC00876712	-36.9879	-37.2764
ZINC19815566	-38.7496	-37.2756
ZINC18038091	-39.8827	-37.2745
ZINC01132230	-40.7322	-37.2711
ZINC06148620	-39.7156	-37.2648
ZINC00669626	-37.1955	-37.2621
ZINC08894829	-40.1528	-37.2581
ZINC08440246	-47.2201	-37.2561
ZINC10232541	-36.4434	-37.2552
ZINC08415288	-38.6335	-37.2548
ZINC01248637	-37.3310	-37.2482
ZINC05045133	-42.7768	-37.2456
ZINC05409041	-36.7252	-37.2453
ZINC09176713	-38.2479	-37.2399
ZINC00663118	-37.6927	-37.2399
ZINC12419632	-40.9426	-37.2387
ZINC00683280	-39.5190	-37.2340
ZINC12463186	-37.7102	-37.2312
ZINC12463186	-37.6818	-37.2312
ZINC12463186	-37.3929	-37.2312
ZINC12463186	-36.6738	-37.2312
ZINC02136458	-48.0551	-37.2291
ZINC00645728	-48.4996	-37.2252
ZINC04067340	-39.3392	-37.2238
ZINC04067340	-38.6793	-37.2238
ZINC00673862	-37.1991	-37.2233
ZINC04780858	-37.1892	-37.2232
ZINC00726564	-36.4479	-37.2204
ZINC09446496	-43.7170	-37.2182
ZINC02063827	-37.0366	-37.2161
ZINC05483712	-37.2953	-37.2158
ZINC05483712	-37.0192	-37.2158
ZINC00702690	-40.0200	-37.2152
ZINC00702690	-40.0024	-37.2152
ZINC00630838	-43.5282	-37.2115
ZINC04067575	-40.6392	-37.2103
ZINC04067575	-38.6518	-37.2103
ZINC09312574	-37.9246	-37.2081
ZINC09312574	-37.6789	-37.2081

ZINC06196118	-40.2706	-37.2071
ZINC05445802	-38.1150	-37.2063
ZINC01251592	-44.9753	-37.2058
ZINC02375447	-37.2326	-37.2046
ZINC00369498	-42.2203	-37.1950
ZINC08739840	-38.5173	-37.1911
ZINC17163284	-36.6511	-37.1881
ZINC09014850	-37.3330	-37.1836
ZINC03094404	-40.6633	-37.1794
ZINC19938439	-45.6846	-37.1772
ZINC04626405	-36.3011	-37.1765
ZINC09240617	-45.9719	-37.1744
ZINC09240617	-45.8730	-37.1744
ZINC05764840	-36.4237	-37.1722
ZINC09236441	-45.2897	-37.1719
ZINC09236441	-44.1790	-37.1719
ZINC09236441	-44.0056	-37.1719
ZINC08438779	-43.2427	-37.1715
ZINC19832196	-37.6477	-37.1714
ZINC04333218	-38.0502	-37.1705
ZINC04333218	-36.7656	-37.1705
ZINC02056405	-37.4790	-37.1672
ZINC06197371	-36.7740	-37.1655
ZINC08684357	-39.1848	-37.1632
ZINC08684357	-38.6951	-37.1632
ZINC00703625	-38.1953	-37.1582
ZINC00627131	-42.0495	-37.1569
ZINC19872453	-36.9483	-37.1538
ZINC09070718	-38.9779	-37.1534
ZINC04066824	-37.2137	-37.1507
ZINC06015299	-39.4976	-37.1490
ZINC06015299	-38.5329	-37.1490
ZINC17246312	-36.2813	-37.1483
ZINC04371961	-37.7749	-37.1479
ZINC04333224	-36.6998	-37.1468
ZINC08441096	-36.8184	-37.1461
ZINC19872441	-37.7796	-37.1458
ZINC12757837	-41.7616	-37.1457
ZINC05450668	-36.8360	-37.1449
ZINC00646689	-46.7880	-37.1435
ZINC00641489	-38.3781	-37.1434
ZINC08396656	-36.2666	-37.1425
ZINC00708856	-36.4314	-37.1409
ZINC18173714	-36.2617	-37.1405
ZINC00483203	-39.2058	-37.1404
ZINC36636404	-40.8153	-37.1392
ZINC17172965	-36.8110	-37.1371
ZINC09369954	-38.5039	-37.1338
ZINC06194181	-38.5424	-37.1322
ZINC00662979	-37.4091	-37.1202
ZINC19872156	-36.8666	-37.1193
ZINC13536184	-37.2910	-37.1147
ZINC00661822	-36.9541	-37.1147
ZINC19924582	-37.9715	-37.1134

ZINC00646445	-37.5612	-37.1129
ZINC08436921	-38.0185	-37.1112
ZINC05045096	-36.9064	-37.1100
ZINC12437172	-39.6892	-37.1048
ZINC00070755	-36.5102	-37.1034
ZINC00677867	-50.1006	-37.1025
ZINC04065165	-37.4099	-37.1017
ZINC04065165	-37.3993	-37.1017
ZINC13122026	-40.3255	-37.0980
ZINC08414000	-37.4376	-37.0959
ZINC08702143	-41.0779	-37.0950
ZINC19938519	-43.6101	-37.0909
ZINC08437429	-48.5194	-37.0896
ZINC10313276	-41.8847	-37.0894
ZINC02135388	-38.7929	-37.0878
ZINC04123548	-39.0406	-37.0872
ZINC04123548	-36.3148	-37.0872
ZINC00696122	-42.7364	-37.0846
ZINC05919715	-36.5244	-37.0830
ZINC01308156	-40.1916	-37.0818
ZINC09360193	-41.6724	-37.0802
ZINC08437196	-43.1296	-37.0798
ZINC05278383	-40.8330	-37.0772
ZINC09453034	-39.1047	-37.0743
ZINC09453034	-38.5840	-37.0743
ZINC10296868	-40.6979	-37.0738
ZINC00842345	-36.4073	-37.0713
ZINC00626580	-40.7175	-37.0712
ZINC05826174	-42.9638	-37.0689
ZINC18087221	-37.9963	-37.0672
ZINC18087221	-37.9836	-37.0672
ZINC00851296	-36.4625	-37.0668
ZINC19770446	-40.3222	-37.0658
ZINC05127395	-39.9579	-37.0590
ZINC05127395	-39.5621	-37.0590
ZINC00123978	-37.3707	-37.0562
ZINC00123978	-36.9652	-37.0562
ZINC33398081	-36.6029	-37.0545
ZINC06194516	-36.6684	-37.0543
ZINC08397425	-41.2280	-37.0533
ZINC06136859	-38.3034	-37.0529
ZINC20234081	-45.6672	-37.0518
ZINC08744478	-40.0134	-37.0469
ZINC09312898	-45.4750	-37.0462
ZINC09312898	-44.7117	-37.0462
ZINC09312898	-44.4486	-37.0462
ZINC09312898	-43.1831	-37.0462
ZINC02088132	-36.9564	-37.0457
ZINC00702953	-48.1290	-37.0454
ZINC00702953	-46.5208	-37.0454
ZINC00702953	-46.1077	-37.0454
ZINC00702953	-45.8979	-37.0454
ZINC00702953	-43.4139	-37.0454
ZINC05359859	-37.1812	-37.0415

ZINC00675304	-38.1036	-37.0371
ZINC19938605	-39.0719	-37.0344
ZINC09014884	-37.5791	-37.0329
ZINC01004231	-36.5722	-37.0313
ZINC13497409	-43.0056	-37.0282
ZINC13497409	-41.5754	-37.0282
ZINC19802179	-41.0441	-37.0282
ZINC19802179	-40.0227	-37.0282
ZINC01117751	-41.9721	-37.0282
ZINC08715689	-38.7186	-37.0251
ZINC08715689	-38.4474	-37.0251
ZINC01021814	-40.3661	-37.0171
ZINC01021814	-37.1395	-37.0171
ZINC50582376	-39.1061	-37.0135
ZINC01795870	-40.1347	-37.0121
ZINC17208019	-38.9822	-37.0112
ZINC17208019	-37.4792	-37.0112
ZINC19889359	-38.5032	-37.0077
ZINC04286348	-39.0122	-37.0076
ZINC08450326	-38.0183	-37.0036
ZINC08450326	-37.0843	-37.0036
ZINC02484845	-38.9771	-37.0021
ZINC01109205	-36.5073	-36.9946
ZINC02836315	-38.1420	-36.9939
ZINC09088330	-40.8293	-36.9936
ZINC08926694	-39.2670	-36.9889
ZINC08926694	-39.1188	-36.9889
ZINC06136758	-39.6461	-36.9876
ZINC09331373	-37.1543	-36.9859
ZINC00626196	-38.7729	-36.9819
ZINC09271701	-36.3287	-36.9797
ZINC00641219	-36.7182	-36.9773
ZINC00191458	-37.1953	-36.9761
ZINC06416087	-36.5469	-36.9736
ZINC09235374	-44.2289	-36.9722
ZINC09235374	-43.9062	-36.9722
ZINC20265273	-45.5767	-36.9673
ZINC09186588	-39.5021	-36.9665
ZINC08397462	-38.5070	-36.9649
ZINC12966562	-39.8447	-36.9641
ZINC05921172	-38.4424	-36.9637
ZINC08440874	-40.0332	-36.9594
ZINC08440874	-37.9863	-36.9594
ZINC08437430	-42.9263	-36.9593
ZINC17160093	-44.6146	-36.9586
ZINC17160093	-44.0613	-36.9586
ZINC17160093	-41.7299	-36.9586
ZINC17160093	-41.6569	-36.9586
ZINC08995434	-41.1333	-36.9529
ZINC04075253	-37.0445	-36.9518
ZINC09186589	-42.1493	-36.9507
ZINC10296917	-41.3998	-36.9469
ZINC38246116	-40.3421	-36.9401
ZINC05483382	-38.7129	-36.9395

ZINC06136921	-37.2642	-36.9378
ZINC09165306	-40.4769	-36.9365
ZINC01070137	-37.0590	-36.9348
ZINC08441403	-40.4638	-36.9345
ZINC08441403	-38.9797	-36.9345
ZINC01114591	-36.4692	-36.9345
ZINC00675308	-37.6856	-36.9296
ZINC18181297	-39.0324	-36.9275
ZINC18181297	-38.9484	-36.9275
ZINC18181297	-38.8317	-36.9275
ZINC01302763	-37.4874	-36.9274
ZINC08384204	-41.1706	-36.9243
ZINC17196733	-40.4178	-36.9206
ZINC09008984	-36.5217	-36.9204
ZINC09008984	-36.4792	-36.9204
ZINC04623849	-37.1536	-36.9204
ZINC04623849	-37.1135	-36.9204
ZINC06758034	-43.9868	-36.9194
ZINC19882573	-43.1968	-36.9186
ZINC15837041	-48.2639	-36.9059
ZINC06136822	-37.8906	-36.9030
ZINC02252325	-36.9892	-36.9021
ZINC02094229	-41.9615	-36.9021
ZINC02094229	-41.8508	-36.9021
ZINC02094229	-40.2881	-36.9021
ZINC02094229	-39.6566	-36.9021
ZINC08836058	-41.6030	-36.8979
ZINC04460205	-38.2153	-36.8978
ZINC05409073	-37.0203	-36.8963
ZINC08384157	-40.4097	-36.8962
ZINC08384157	-39.6605	-36.8962
ZINC19702001	-36.8019	-36.8897
ZINC09015583	-37.7694	-36.8871
ZINC01248636	-38.4594	-36.8830
ZINC06015285	-36.8719	-36.8783
ZINC06015285	-36.4894	-36.8783
ZINC02115661	-38.1148	-36.8778
ZINC02262447	-37.8225	-36.8766
ZINC12559038	-47.8959	-36.8766
ZINC05130915	-40.6899	-36.8741
ZINC00664258	-38.4910	-36.8735
ZINC09241002	-38.9077	-36.8694
ZINC09241002	-38.2751	-36.8694
ZINC00664889	-40.1718	-36.8688
ZINC00703027	-41.6243	-36.8660
ZINC19909495	-39.2400	-36.8646
ZINC06406878	-38.7430	-36.8636
ZINC19841697	-38.7763	-36.8587
ZINC02441420	-36.5788	-36.8578
ZINC02377786	-38.0161	-36.8573
ZINC08441905	-40.5839	-36.8567
ZINC03368381	-46.6659	-36.8566
ZINC06144740	-38.4880	-36.8543
ZINC19802212	-36.9292	-36.8534

ZINC00702727	-41.5329	-36.8532
ZINC00673819	-41.5319	-36.8503
ZINC08715744	-38.0345	-36.8453
ZINC05130506	-41.4583	-36.8441
ZINC08438528	-40.2725	-36.8406
ZINC09044110	-38.2587	-36.8400
ZINC05127295	-36.3199	-36.8390
ZINC09375278	-38.1490	-36.8377
ZINC00386791	-37.7546	-36.8316
ZINC04623855	-39.5461	-36.8292
ZINC04623855	-36.6402	-36.8292
ZINC02262019	-37.0957	-36.8284
ZINC08845427	-40.5570	-36.8268
ZINC00645007	-43.8166	-36.8240
ZINC05130912	-42.3085	-36.8224
ZINC19370659	-43.1038	-36.8192
ZINC00641563	-36.2673	-36.8097
ZINC19923438	-44.8601	-36.8049
ZINC18045318	-37.0934	-36.8039
ZINC19815020	-37.9748	-36.8035
ZINC00719489	-40.1248	-36.8019
ZINC19938610	-36.5699	-36.8016
ZINC04067477	-38.9480	-36.8014
ZINC04067477	-37.0334	-36.8014
ZINC20308659	-43.4542	-36.8013
ZINC13685761	-36.8689	-36.7979
ZINC19871550	-38.6207	-36.7942
ZINC19938308	-45.5490	-36.7938
ZINC08378721	-36.2584	-36.7928
ZINC00717631	-41.0631	-36.7901
ZINC00717631	-37.2693	-36.7901
ZINC10294983	-36.7493	-36.7894
ZINC01829984	-36.7336	-36.7887
ZINC08440810	-40.7826	-36.7876
ZINC08440810	-39.4126	-36.7876
ZINC02135560	-37.6297	-36.7861
ZINC05886839	-39.3200	-36.7844
ZINC05886839	-38.9773	-36.7844
ZINC00630924	-42.1207	-36.7842
ZINC19923128	-43.4543	-36.7798
ZINC06149669	-41.7494	-36.7767
ZINC04114816	-36.3084	-36.7756
ZINC09210204	-45.6963	-36.7737
ZINC09210204	-44.8273	-36.7737
ZINC09210204	-43.7844	-36.7737
ZINC08818169	-37.1879	-36.7705
ZINC05610964	-37.1768	-36.7603
ZINC08439631	-37.4215	-36.7586
ZINC00666756	-38.4429	-36.7576
ZINC12759013	-43.2594	-36.7530
ZINC00480476	-36.3740	-36.7523
ZINC17971947	-39.1153	-36.7450
ZINC00363791	-38.2624	-36.7412
ZINC04728318	-37.1530	-36.7398

ZINC04728318	-36.6615	-36.7398
ZINC08836126	-41.5125	-36.7383
ZINC00645582	-37.1705	-36.7331
ZINC08424623	-37.4559	-36.7288
ZINC08424623	-37.0931	-36.7288
ZINC08424623	-36.9506	-36.7288
ZINC06194519	-37.2407	-36.7265
ZINC00189166	-37.0571	-36.7258
ZINC20101045	-42.9082	-36.7255
ZINC06195756	-36.3741	-36.7202
ZINC09008044	-40.1297	-36.7191
ZINC05448592	-39.2585	-36.7167
ZINC08438685	-42.1556	-36.7136
ZINC08438685	-41.9039	-36.7136
ZINC12138624	-36.5372	-36.7123
ZINC18209611	-37.0257	-36.7067
ZINC06197254	-38.8197	-36.7012
ZINC02057996	-37.5715	-36.7005
ZINC00673868	-37.1361	-36.7000
ZINC00641044	-36.3685	-36.6970
ZINC18122430	-37.4328	-36.6970
ZINC18055923	-36.9877	-36.6948
ZINC18055923	-36.4766	-36.6948
ZINC04168140	-36.3528	-36.6946
ZINC09186811	-44.7174	-36.6945
ZINC09186811	-43.2044	-36.6945
ZINC00666922	-41.2898	-36.6936
ZINC08410111	-36.6264	-36.6935
ZINC00671022	-41.5703	-36.6912
ZINC02886256	-47.0760	-36.6873
ZINC03901612	-36.4808	-36.6853
ZINC08413122	-39.0330	-36.6836
ZINC13896940	-38.8342	-36.6787
ZINC13896940	-37.7950	-36.6787
ZINC68741001	-36.3707	-36.6786
ZINC20227867	-46.2471	-36.6765
ZINC08972819	-40.3828	-36.6761
ZINC08972819	-40.0282	-36.6761
ZINC05130914	-40.9077	-36.6731
ZINC01000040	-42.8736	-36.6720
ZINC08426416	-37.4331	-36.6692
ZINC02103100	-39.9766	-36.6690
ZINC06195754	-38.1841	-36.6676
ZINC09009214	-46.8482	-36.6664
ZINC05014456	-39.1794	-36.6628
ZINC18061478	-38.2354	-36.6489
ZINC09123656	-37.0150	-36.6464
ZINC01302765	-38.3137	-36.6455
ZINC08996611	-36.3860	-36.6419
ZINC08996611	-36.3256	-36.6419
ZINC13607396	-39.1526	-36.6394
ZINC19706498	-38.3577	-36.6374
ZINC02088416	-37.1957	-36.6332
ZINC00677688	-36.4652	-36.6321

ZINC19789598	-36.2668	-36.6266
ZINC09046914	-37.8688	-36.6249
ZINC04611249	-39.2914	-36.6217
ZINC04611249	-37.7142	-36.6217
ZINC04611249	-37.4029	-36.6217
ZINC04611249	-36.7880	-36.6217
ZINC20081616	-46.6587	-36.6138
ZINC00722097	-37.6103	-36.6132
ZINC00702308	-38.4152	-36.6130
ZINC00702308	-37.8786	-36.6130
ZINC00681662	-37.4110	-36.6115
ZINC06162350	-36.7845	-36.6093
ZINC06162255	-37.7171	-36.6090
ZINC20192346	-37.0071	-36.6070
ZINC09357463	-48.5639	-36.6058
ZINC09357463	-46.7044	-36.6058
ZINC09313384	-40.4685	-36.6052
ZINC09313384	-36.7782	-36.6052
ZINC19897484	-37.5460	-36.5967
ZINC12849901	-38.5855	-36.5948
ZINC08438781	-38.4635	-36.5898
ZINC68712916	-41.4071	-36.5861
ZINC68712916	-38.9627	-36.5861
ZINC05959401	-36.3717	-36.5831
ZINC00703543	-41.7525	-36.5826
ZINC02239690	-39.3859	-36.5821
ZINC09459770	-41.5605	-36.5809
ZINC00853797	-37.9729	-36.5799
ZINC00853797	-37.8961	-36.5799
ZINC17123874	-41.6418	-36.5797
ZINC00987833	-40.5906	-36.5786
ZINC08453993	-39.5507	-36.5773
ZINC08715759	-37.4186	-36.5766
ZINC08715759	-37.1179	-36.5766
ZINC19922940	-37.0267	-36.5725
ZINC19922940	-36.8496	-36.5725
ZINC20027325	-42.5413	-36.5724
ZINC18269690	-36.9075	-36.5699
ZINC00625740	-37.9731	-36.5699
ZINC00625740	-36.8411	-36.5699
ZINC04067002	-36.6094	-36.5666
ZINC08904651	-37.1535	-36.5661
ZINC08904651	-37.0992	-36.5661
ZINC20263917	-44.5672	-36.5619
ZINC16944882	-37.8751	-36.5569
ZINC04019664	-46.0474	-36.5567
ZINC04019664	-45.4005	-36.5567
ZINC04019664	-44.8038	-36.5567
ZINC04019664	-44.6789	-36.5567
ZINC04019664	-43.6911	-36.5567
ZINC09167286	-41.4150	-36.5544
ZINC08385415	-37.7437	-36.5525
ZINC06194262	-36.9863	-36.5502
ZINC02252327	-38.2861	-36.5499

ZINC13127254	-47.3141	-36.5490
ZINC00726566	-36.3459	-36.5450
ZINC19815638	-37.9017	-36.5400
ZINC19815638	-36.5690	-36.5400
ZINC20116586	-36.4676	-36.5398
ZINC05360325	-41.2513	-36.5382
ZINC19370919	-36.5367	-36.5368
ZINC08385606	-37.5600	-36.5331
ZINC08465700	-47.1229	-36.5330
ZINC08465700	-45.4405	-36.5330
ZINC00727717	-38.6032	-36.5321
ZINC00983811	-37.4216	-36.5310
ZINC08439662	-38.0553	-36.5296
ZINC04610999	-36.7605	-36.5246
ZINC12850017	-38.3944	-36.5223
ZINC05200505	-46.5949	-36.5218
ZINC08433427	-41.9856	-36.5200
ZINC05576785	-37.4271	-36.5186
ZINC09334490	-47.0528	-36.5179
ZINC09334490	-42.7234	-36.5179
ZINC09334490	-41.7288	-36.5179
ZINC00634925	-38.2888	-36.5141
ZINC00678351	-41.3503	-36.5131
ZINC09370626	-36.5329	-36.5121
ZINC00127139	-36.7419	-36.5095
ZINC09243938	-40.1738	-36.5094
ZINC09243938	-39.6473	-36.5094
ZINC18120271	-39.3622	-36.5038
ZINC18120271	-38.5065	-36.5038
ZINC36065975	-38.0570	-36.5022
ZINC03903086	-39.1521	-36.4982
ZINC03903086	-38.7628	-36.4982
ZINC09272880	-40.5665	-36.4979
ZINC32497050	-38.6287	-36.4969
ZINC19938385	-45.4897	-36.4952
ZINC06786627	-36.8113	-36.4926
ZINC04068322	-38.1995	-36.4908
ZINC04068322	-37.3532	-36.4908
ZINC20171121	-45.9363	-36.4907
ZINC08439611	-37.4658	-36.4797
ZINC08439611	-37.3872	-36.4797
ZINC08439611	-37.0353	-36.4797
ZINC02072007	-47.8459	-36.4761
ZINC00655598	-41.5451	-36.4747
ZINC06194340	-36.9303	-36.4741
ZINC06162351	-37.9321	-36.4713
ZINC00673743	-39.1077	-36.4629
ZINC09089035	-36.9345	-36.4507
ZINC08440218	-41.3936	-36.4470
ZINC08440218	-40.1311	-36.4470
ZINC08440218	-39.8577	-36.4470
ZINC08440218	-38.6930	-36.4470
ZINC08440218	-38.5410	-36.4470
ZINC09271896	-38.9693	-36.4453

ZINC09271896	-38.8243	-36.4453
ZINC04062964	-37.0002	-36.4453
ZINC04062964	-36.8386	-36.4453
ZINC06015591	-36.8690	-36.4446
ZINC09123116	-45.5145	-36.4440
ZINC09123116	-44.6528	-36.4440
ZINC09374973	-38.7595	-36.4426
ZINC08462855	-41.5174	-36.4389
ZINC08462855	-39.5802	-36.4389
ZINC04473460	-40.5005	-36.4388
ZINC08828908	-44.0690	-36.4382
ZINC36636411	-39.2443	-36.4349
ZINC19815572	-38.3554	-36.4341
ZINC06195513	-37.3204	-36.4331
ZINC71405093	-46.2250	-36.4320
ZINC04312394	-38.6115	-36.4320
ZINC00728017	-37.7586	-36.4312
ZINC08328451	-38.3848	-36.4308
ZINC06194393	-37.5715	-36.4300
ZINC09008929	-45.9038	-36.4279
ZINC09008929	-45.8496	-36.4279
ZINC09043101	-43.0766	-36.4269
ZINC20759885	-45.5036	-36.4239
ZINC12465529	-36.6890	-36.4216
ZINC09375277	-38.3026	-36.4198
ZINC04374469	-36.4762	-36.4158
ZINC00668861	-37.4115	-36.4140
ZINC13552612	-43.4331	-36.4132
ZINC13552612	-42.0065	-36.4132
ZINC13552612	-40.7763	-36.4132
ZINC13372070	-37.5685	-36.4109
ZINC08441424	-38.5977	-36.4035
ZINC04148785	-36.8976	-36.4034
ZINC13372068	-38.6369	-36.3995
ZINC08836386	-39.0656	-36.3978
ZINC36646220	-38.9537	-36.3864
ZINC05045134	-39.9778	-36.3863
ZINC05045134	-38.4685	-36.3863
ZINC08715767	-39.7700	-36.3847
ZINC57717490	-40.0222	-36.3822
ZINC09329278	-37.6933	-36.3773
ZINC00678140	-37.1593	-36.3758
ZINC05483707	-39.3124	-36.3703
ZINC05483707	-36.6656	-36.3703
ZINC06137407	-39.0109	-36.3665
ZINC18159366	-36.3436	-36.3661
ZINC08827362	-45.0955	-36.3654
ZINC08827362	-44.5214	-36.3654
ZINC08827362	-43.7735	-36.3654
ZINC06445240	-36.7660	-36.3634
ZINC19923101	-43.2911	-36.3585
ZINC09008749	-43.7496	-36.3580
ZINC09008749	-40.5655	-36.3580
ZINC13550150	-36.3501	-36.3570

ZINC12475325	-40.1463	-36.3569
ZINC05917674	-37.0057	-36.3484
ZINC18032015	-37.2997	-36.3459
ZINC36646112	-39.5438	-36.3458
ZINC19852991	-37.1821	-36.3429
ZINC08442127	-38.8883	-36.3418
ZINC09008255	-41.8121	-36.3399
ZINC08410281	-36.9979	-36.3370
ZINC08306355	-38.3636	-36.3356
ZINC00673930	-38.0450	-36.3344
ZINC17242367	-41.2177	-36.3341
ZINC00994846	-36.9741	-36.3326
ZINC12437283	-38.9779	-36.3324
ZINC00665471	-42.3537	-36.3291
ZINC09244107	-41.9276	-36.3289
ZINC09244107	-40.2321	-36.3289
ZINC00836245	-39.1949	-36.3277
ZINC02701538	-38.8645	-36.3218
ZINC06194252	-37.5653	-36.3217
ZINC00711483	-38.9483	-36.3216
ZINC09243851	-38.4229	-36.3211
ZINC04155030	-37.3356	-36.3201
ZINC09012761	-38.9046	-36.3171
ZINC00111405	-36.2613	-36.3152
ZINC00628208	-37.7224	-36.3146
ZINC00647540	-37.1665	-36.3101
ZINC00709835	-38.3481	-36.3093
ZINC00414577	-38.6446	-36.3089
ZINC04950701	-37.8500	-36.3082
ZINC04950701	-37.7823	-36.3082
ZINC03991788	-50.0280	-36.3081
ZINC03991788	-50.0280	-36.3081
ZINC17167273	-37.8607	-36.3050
ZINC19923523	-36.7574	-36.2965
ZINC00681650	-37.2071	-36.2948
ZINC32602289	-36.2674	-36.2881
ZINC17143733	-37.6818	-36.2867
ZINC04065658	-39.2548	-36.2853
ZINC04065658	-37.7658	-36.2853
ZINC04065658	-36.8554	-36.2853
ZINC02060834	-40.0027	-36.2841
ZINC08383684	-39.1517	-36.2829
ZINC08440768	-38.3393	-36.2801
ZINC08440768	-36.3021	-36.2801
ZINC31854415	-39.3267	-36.2793
ZINC08440697	-42.4193	-36.2780
ZINC08739161	-44.5891	-36.2767
ZINC08739161	-44.0943	-36.2767
ZINC08739161	-43.6814	-36.2767
ZINC08739161	-43.4459	-36.2767
ZINC08739161	-40.2545	-36.2767
ZINC32496988	-39.3996	-36.2755
ZINC19551802	-36.6204	-36.2713
ZINC04060269	-36.9817	-36.2708

ZINC18068456	-37.8717	-36.2683
ZINC18068456	-37.7832	-36.2683
ZINC08717094	-41.2538	-36.2643
ZINC08426419	-36.5198	-36.2639
ZINC09357380	-39.1888	-36.2632
ZINC06196050	-37.8896	-36.2597
ZINC02066564	-37.0014	-36.2552
ZINC20233911	-45.7127	-36.2547
ZINC08440775	-36.5767	-36.2439
ZINC08437142	-37.8211	-36.2435
ZINC09243348	-45.3261	-36.2413
ZINC16944685	-50.5174	-36.2356
ZINC16944685	-48.9027	-36.2356
ZINC06088635	-44.5007	-36.2348
ZINC04631635	-36.6099	-36.2311
ZINC13111604	-42.3408	-36.2250
ZINC02077139	-53.3524	-36.2239
ZINC00679500	-39.0941	-36.2230
ZINC20265471	-43.1827	-36.2225
ZINC00703565	-43.6303	-36.2221
ZINC08383917	-36.7545	-36.2216
ZINC06745196	-36.5958	-36.2178
ZINC00716683	-36.9162	-36.2123
ZINC08426291	-38.8366	-36.2087
ZINC08920943	-42.2863	-36.2073
ZINC08920943	-40.8949	-36.2073
ZINC08920943	-36.2682	-36.2073
ZINC13120607	-40.4197	-36.2042
ZINC02135460	-38.0835	-36.2033
ZINC09089412	-38.0498	-36.2029
ZINC01055079	-48.2320	-36.2009
ZINC00716242	-37.6351	-36.1988
ZINC00716242	-37.0593	-36.1988
ZINC00865793	-36.3797	-36.1961
ZINC19872064	-45.6196	-36.1936
ZINC19938389	-45.3716	-36.1927
ZINC04168187	-36.9761	-36.1916
ZINC17145498	-46.5392	-36.1885
ZINC17145498	-42.8951	-36.1885
ZINC17145498	-41.8412	-36.1885
ZINC17145498	-40.7562	-36.1885
ZINC17145498	-40.7468	-36.1885
ZINC19878710	-42.0198	-36.1836
ZINC08766585	-36.3538	-36.1787
ZINC04320267	-37.0465	-36.1756
ZINC02520981	-39.1677	-36.1743
ZINC02709613	-36.5997	-36.1723
ZINC19555355	-38.5338	-36.1715
ZINC19555355	-36.5369	-36.1715
ZINC19872338	-36.8972	-36.1698
ZINC19923134	-46.8072	-36.1669
ZINC12411696	-45.4404	-36.1658
ZINC09271687	-40.9061	-36.1658
ZINC68740903	-47.9885	-36.1638

ZINC00729255	-47.0490	-36.1630
ZINC02060820	-37.3213	-36.1595
ZINC08441630	-39.9086	-36.1576
ZINC08441630	-38.7054	-36.1576
ZINC19329523	-36.5496	-36.1555
ZINC05490535	-37.7705	-36.1530
ZINC05490535	-36.7819	-36.1530
ZINC00727828	-37.0445	-36.1496
ZINC00051133	-37.8273	-36.1495
ZINC08415283	-38.2906	-36.1431
ZINC08415283	-38.0130	-36.1431
ZINC19797047	-38.8109	-36.1388
ZINC08433274	-40.8658	-36.1388
ZINC13570878	-38.5076	-36.1365
ZINC00727342	-41.8971	-36.1298
ZINC00681648	-38.0671	-36.1267
ZINC00850904	-40.8301	-36.1211
ZINC06015252	-39.3639	-36.1154
ZINC05491442	-38.6981	-36.1121
ZINC39929930	-36.7309	-36.1096
ZINC08384276	-42.3029	-36.1088
ZINC08384276	-42.0333	-36.1088
ZINC19872250	-39.2782	-36.1036
ZINC04187678	-36.5644	-36.1016
ZINC08400182	-37.8514	-36.0997
ZINC03635625	-42.4175	-36.0952
ZINC13370456	-38.8283	-36.0950
ZINC00850084	-39.9463	-36.0924
ZINC02191051	-38.6565	-36.0910
ZINC08435579	-37.5638	-36.0681
ZINC75273126	-37.2965	-36.0664
ZINC08433265	-40.8769	-36.0576
ZINC04067946	-37.0010	-36.0563
ZINC19871621	-39.0859	-36.0519
ZINC00727720	-40.3175	-36.0510
ZINC05053287	-40.5406	-36.0435
ZINC05053287	-36.9505	-36.0435
ZINC12373961	-39.1311	-36.0405
ZINC19852630	-39.3079	-36.0386
ZINC13551217	-42.5120	-36.0382
ZINC13551217	-41.1482	-36.0382
ZINC08996930	-36.2857	-36.0362
ZINC10294931	-37.6462	-36.0361
ZINC05488894	-37.1763	-36.0331
ZINC05488894	-37.1698	-36.0331
ZINC09363563	-36.4578	-36.0302
ZINC33336418	-43.6466	-36.0256
ZINC13544852	-37.7025	-36.0214
ZINC18119210	-36.4557	-36.0178
ZINC18119210	-36.4454	-36.0178
ZINC19909706	-37.8679	-36.0135
ZINC03876751	-37.6637	-36.0096
ZINC19801742	-38.6663	-36.0037
ZINC00846403	-41.8765	-36.0008

ZINC00627216	-39.9262	-36.0007
ZINC00116265	-37.4151	-35.9966
ZINC13575611	-36.5625	-35.9962
ZINC19797048	-39.0357	-35.9918
ZINC20065918	-43.6661	-35.9897
ZINC00847241	-37.8663	-35.9886
ZINC00255546	-40.5270	-35.9867
ZINC05409058	-37.9876	-35.9840
ZINC05409058	-36.7045	-35.9840
ZINC00061101	-36.5149	-35.9813
ZINC00673878	-38.7980	-35.9787
ZINC00673878	-38.6072	-35.9787
ZINC08442106	-36.6311	-35.9760
ZINC08766587	-37.1080	-35.9736
ZINC00647148	-39.6031	-35.9640
ZINC00428651	-40.7429	-35.9639
ZINC08400026	-37.6556	-35.9627
ZINC20229282	-45.4144	-35.9626
ZINC02314898	-36.4602	-35.9616
ZINC06137185	-36.7576	-35.9603
ZINC06137185	-36.5775	-35.9603
ZINC06137185	-36.5192	-35.9603
ZINC19287948	-37.1949	-35.9561
ZINC05490625	-37.0038	-35.9545
ZINC13370459	-39.4089	-35.9545
ZINC02586961	-38.0018	-35.9493
ZINC58146935	-37.7574	-35.9450
ZINC09302056	-42.5707	-35.9450
ZINC09302056	-41.9416	-35.9450
ZINC09302056	-40.0936	-35.9450
ZINC04068046	-37.3677	-35.9429
ZINC05445193	-36.3954	-35.9399
ZINC08408100	-36.7951	-35.9369
ZINC19691696	-36.4218	-35.9343
ZINC02056410	-36.7574	-35.9337
ZINC00846402	-40.1028	-35.9290
ZINC00374129	-40.0697	-35.9289
ZINC19938402	-38.1953	-35.9266
ZINC13382057	-38.0805	-35.9218
ZINC13382057	-37.6986	-35.9218
ZINC12660802	-36.6521	-35.9177
ZINC17166895	-36.9139	-35.9153
ZINC06565157	-40.5074	-35.9151
ZINC00624050	-36.3782	-35.9093
ZINC00677058	-37.8998	-35.9085
ZINC75251488	-36.4335	-35.9001
ZINC09275531	-40.5929	-35.8968
ZINC09275531	-40.0900	-35.8968
ZINC00876718	-36.4276	-35.8931
ZINC00669864	-44.9908	-35.8904
ZINC08437141	-41.9839	-35.8897
ZINC16971691	-37.3664	-35.8851
ZINC09358636	-37.5227	-35.8823
ZINC15985716	-37.9102	-35.8821

ZINC00937412	-42.8403	-35.8733
ZINC10313238	-39.5118	-35.8722
ZINC12416697	-36.8709	-35.8708
ZINC00983235	-37.1516	-35.8700
ZINC17193962	-41.4119	-35.8676
ZINC17193962	-40.5184	-35.8676
ZINC05491454	-38.3225	-35.8654
ZINC00647546	-37.6281	-35.8651
ZINC08440542	-39.7362	-35.8610
ZINC01019852	-36.5652	-35.8601
ZINC17154695	-40.6793	-35.8573
ZINC17154695	-36.6029	-35.8573
ZINC08442014	-47.0539	-35.8564
ZINC16115295	-36.5362	-35.8550
ZINC09332449	-36.5635	-35.8541
ZINC05731703	-41.1169	-35.8520
ZINC08397238	-39.0043	-35.8506
ZINC18176729	-36.3987	-35.8416
ZINC08441863	-37.2040	-35.8363
ZINC12970066	-36.7617	-35.8345
ZINC00999235	-40.7056	-35.8330
ZINC19877196	-37.4143	-35.8285
ZINC01145120	-36.8842	-35.8277
ZINC01145120	-36.6367	-35.8277
ZINC03635605	-45.4486	-35.8228
ZINC06136857	-37.0652	-35.8181
ZINC20175467	-37.7751	-35.8171
ZINC00674978	-37.6086	-35.8141
ZINC06136820	-38.5421	-35.8135
ZINC00628111	-36.5364	-35.8134
ZINC00645713	-41.9292	-35.8117
ZINC13030560	-39.4599	-35.8096
ZINC05126767	-38.9825	-35.8027
ZINC05126767	-38.3213	-35.8027
ZINC02180582	-37.6733	-35.8010
ZINC02081355	-38.9997	-35.8003
ZINC00673102	-37.4111	-35.7953
ZINC00674226	-39.1760	-35.7883
ZINC01019636	-44.5722	-35.7857
ZINC05408917	-38.8975	-35.7849
ZINC02055843	-43.0709	-35.7845
ZINC02135518	-39.5715	-35.7844
ZINC09236291	-44.3022	-35.7827
ZINC06162328	-38.0790	-35.7814
ZINC00702763	-43.7550	-35.7783
ZINC00702763	-40.1397	-35.7783
ZINC20190952	-43.4620	-35.7781
ZINC05447816	-38.8297	-35.7736
ZINC05905806	-39.5288	-35.7700
ZINC05905806	-37.0029	-35.7700
ZINC05446158	-42.2757	-35.7697
ZINC19909441	-36.6491	-35.7696
ZINC20231537	-45.5407	-35.7691
ZINC08440067	-38.1846	-35.7681

ZINC08440067	-36.9827	-35.7681
ZINC00647314	-38.0840	-35.7669
ZINC02057282	-38.2225	-35.7641
ZINC05490556	-38.0501	-35.7612
ZINC04312176	-37.6772	-35.7586
ZINC04312176	-36.4245	-35.7586
ZINC01272909	-37.4947	-35.7570
ZINC17166733	-37.3724	-35.7543
ZINC17166733	-37.3720	-35.7543
ZINC08837660	-42.6529	-35.7536
ZINC08837660	-42.2399	-35.7536
ZINC08837660	-41.9314	-35.7536
ZINC08837660	-40.7355	-35.7536
ZINC08837660	-39.8763	-35.7536
ZINC16973396	-37.1496	-35.7533
ZINC00823703	-36.4022	-35.7511
ZINC00846416	-39.1687	-35.7506
ZINC19721026	-42.3804	-35.7505
ZINC02064461	-39.7502	-35.7500
ZINC19872401	-37.2012	-35.7451
ZINC09359108	-37.3335	-35.7437
ZINC19872480	-38.3995	-35.7407
ZINC00675310	-37.3296	-35.7379
ZINC19368566	-40.0837	-35.7361
ZINC06144470	-37.3730	-35.7359
ZINC08414884	-36.7990	-35.7317
ZINC08425988	-36.5839	-35.7285
ZINC19923530	-36.9565	-35.7254
ZINC08407492	-38.3862	-35.7239
ZINC20025548	-38.4036	-35.7227
ZINC09014429	-42.0763	-35.7194
ZINC09014429	-41.6050	-35.7194
ZINC09014429	-39.6529	-35.7194
ZINC06783979	-38.8327	-35.7133
ZINC06783979	-37.6912	-35.7133
ZINC04391637	-39.2942	-35.7105
ZINC05408574	-37.4475	-35.7104
ZINC19872369	-37.4842	-35.7092
ZINC20028464	-38.3867	-35.7077
ZINC17179836	-40.2426	-35.7048
ZINC36646277	-41.8178	-35.7040
ZINC18249517	-40.8806	-35.7001
ZINC00625755	-36.6586	-35.6964
ZINC12371592	-36.6198	-35.6933
ZINC05408689	-37.0572	-35.6880
ZINC08415486	-36.6484	-35.6877
ZINC04075403	-36.6708	-35.6805
ZINC15973770	-37.6187	-35.6741
ZINC15973770	-37.2452	-35.6741
ZINC08715770	-36.7745	-35.6677
ZINC08715770	-36.3907	-35.6677
ZINC02137080	-45.1647	-35.6657
ZINC09012325	-36.7222	-35.6618
ZINC19370600	-37.0009	-35.6618

ZINC22972202	-36.9978	-35.6579
ZINC17819694	-37.2195	-35.6569
ZINC06015511	-36.7682	-35.6525
ZINC00727041	-36.2766	-35.6472
ZINC00987874	-36.4050	-35.6441
ZINC01001417	-36.4897	-35.6409
ZINC09334234	-43.7652	-35.6393
ZINC09334234	-42.9190	-35.6393
ZINC09334234	-42.6753	-35.6393
ZINC05130907	-36.5379	-35.6374
ZINC16972341	-36.5262	-35.6257
ZINC19313423	-36.5135	-35.6218
ZINC00633355	-36.9933	-35.6147
ZINC02757440	-37.8471	-35.6141
ZINC19758881	-37.2403	-35.6081
ZINC00625750	-37.3816	-35.6035
ZINC15015825	-38.7979	-35.5986
ZINC15015825	-38.5771	-35.5986
ZINC15015825	-38.1439	-35.5986
ZINC15015825	-36.8995	-35.5986
ZINC00988128	-38.4033	-35.5690
ZINC19801707	-38.0766	-35.5676
ZINC19801707	-37.1844	-35.5676
ZINC19801707	-36.5029	-35.5676
ZINC20265474	-45.5792	-35.5505
ZINC20588746	-41.8390	-35.5496
ZINC01003774	-42.4040	-35.5459
ZINC13468118	-38.9608	-35.5440
ZINC13468118	-37.9964	-35.5440
ZINC09456069	-37.8162	-35.5427
ZINC10013879	-41.6401	-35.5274
ZINC08439334	-50.6999	-35.5260
ZINC02060833	-39.5566	-35.5256
ZINC08845353	-41.8182	-35.5221
ZINC08988168	-42.2304	-35.5191
ZINC08988168	-42.2134	-35.5191
ZINC09042783	-38.5398	-35.5164
ZINC18163560	-40.0580	-35.5025
ZINC19872124	-36.7512	-35.4995
ZINC00723825	-37.3388	-35.4989
ZINC00676411	-46.4660	-35.4967
ZINC04322609	-39.4827	-35.4933
ZINC00195281	-37.7600	-35.4893
ZINC09437087	-37.7297	-35.4807
ZINC09437087	-36.8237	-35.4807
ZINC08450288	-38.1741	-35.4729
ZINC19882571	-40.5551	-35.4719
ZINC19814707	-37.2456	-35.4712
ZINC08900517	-46.8954	-35.4702
ZINC08900517	-44.2997	-35.4702
ZINC08900517	-43.4735	-35.4702
ZINC04658211	-38.0801	-35.4672
ZINC17299647	-38.6497	-35.4663
ZINC17299647	-37.8619	-35.4663

ZINC02135484	-38.4665	-35.4624
ZINC00725694	-37.9920	-35.4619
ZINC00725694	-36.5978	-35.4619
ZINC02088131	-36.5355	-35.4604
ZINC04114896	-36.8359	-35.4544
ZINC06136862	-37.0226	-35.4529
ZINC17196162	-37.3571	-35.4462
ZINC12849895	-37.5440	-35.4409
ZINC00706601	-37.2328	-35.4373
ZINC00865771	-36.6680	-35.4357
ZINC08396502	-39.0857	-35.4350
ZINC00729013	-38.3836	-35.4348
ZINC00729013	-38.1243	-35.4348
ZINC00729013	-37.8772	-35.4348
ZINC19872306	-36.7618	-35.4324
ZINC09459769	-39.8212	-35.4305
ZINC13955273	-40.8003	-35.4295
ZINC00675306	-36.6731	-35.4292
ZINC04065152	-38.1969	-35.4288
ZINC04065152	-37.0040	-35.4288
ZINC00688916	-40.1082	-35.4287
ZINC08426044	-38.4693	-35.4234
ZINC00625759	-37.1853	-35.4220
ZINC00864421	-39.8250	-35.4199
ZINC00864421	-36.8627	-35.4199
ZINC04061139	-36.6355	-35.4191
ZINC08440087	-39.0962	-35.4156
ZINC04116473	-36.8514	-35.4154
ZINC04116473	-36.3776	-35.4154
ZINC00351109	-36.6618	-35.4128
ZINC19904598	-42.2097	-35.4124
ZINC08715671	-38.6505	-35.4094
ZINC08715671	-38.1522	-35.4094
ZINC00711715	-38.4998	-35.4090
ZINC06161989	-36.3056	-35.4079
ZINC00626217	-38.3471	-35.4071
ZINC00705688	-39.6779	-35.4041
ZINC00850874	-39.0624	-35.4034
ZINC01271038	-37.1339	-35.4025
ZINC04060939	-37.5158	-35.3994
ZINC09008458	-44.0888	-35.3943
ZINC09008458	-42.5939	-35.3943
ZINC18007140	-37.4646	-35.3871
ZINC06444891	-36.6644	-35.3848
ZINC00644729	-45.3200	-35.3834
ZINC08715457	-36.4589	-35.3828
ZINC09353897	-44.3525	-35.3826
ZINC09353897	-43.2392	-35.3826
ZINC09353897	-41.6931	-35.3826
ZINC08442021	-43.7876	-35.3776
ZINC08442021	-41.9870	-35.3776
ZINC08414959	-37.1479	-35.3739
ZINC09349787	-41.9471	-35.3738
ZINC08385416	-37.5443	-35.3736

ZINC08385416	-37.0599	-35.3736
ZINC68732632	-39.5444	-35.3725
ZINC01030074	-36.4465	-35.3703
ZINC08437226	-41.6337	-35.3657
ZINC18245083	-39.7147	-35.3644
ZINC05408352	-38.0547	-35.3626
ZINC00894617	-38.2260	-35.3624
ZINC05426572	-38.6189	-35.3618
ZINC05426572	-37.9156	-35.3618
ZINC08437236	-40.5469	-35.3568
ZINC19938403	-36.8244	-35.3498
ZINC00384697	-36.3211	-35.3490
ZINC03894914	-36.8415	-35.3459
ZINC08837200	-44.5387	-35.3453
ZINC20228711	-37.6831	-35.3380
ZINC09357607	-42.0904	-35.3369
ZINC08442499	-45.6756	-35.3316
ZINC08442499	-44.4313	-35.3316
ZINC08442499	-42.4965	-35.3316
ZINC08440735	-37.1955	-35.3291
ZINC08438656	-39.5603	-35.3271
ZINC19815426	-38.2320	-35.3246
ZINC19683213	-37.6286	-35.3221
ZINC19816088	-36.7990	-35.3164
ZINC08425426	-37.6651	-35.3144
ZINC08425426	-37.4019	-35.3144
ZINC08988172	-41.1079	-35.3119
ZINC08988172	-40.9778	-35.3119
ZINC02266401	-36.5645	-35.3118
ZINC19904610	-42.4039	-35.3106
ZINC08440065	-38.9310	-35.3044
ZINC08440065	-37.4028	-35.3044
ZINC22874513	-36.6030	-35.3016
ZINC19804795	-36.8372	-35.3012
ZINC08817571	-43.0887	-35.2989
ZINC00665828	-41.1698	-35.2986
ZINC02486403	-37.5463	-35.2937
ZINC02694979	-45.1891	-35.2933
ZINC01213875	-38.4102	-35.2871
ZINC00713703	-38.8555	-35.2856
ZINC13127640	-45.2709	-35.2852
ZINC13127640	-40.1417	-35.2852
ZINC08435556	-39.2178	-35.2839
ZINC05408678	-36.5504	-35.2829
ZINC01308154	-40.1430	-35.2815
ZINC15937638	-41.0368	-35.2740
ZINC08439872	-38.8184	-35.2695
ZINC08715594	-39.9542	-35.2671
ZINC05409031	-42.9267	-35.2661
ZINC05409031	-41.4723	-35.2661
ZINC05409031	-40.4339	-35.2661
ZINC02055217	-37.7788	-35.2635
ZINC04649769	-37.2123	-35.2625
ZINC19904209	-39.2109	-35.2599

ZINC18045229	-37.9805	-35.2563
ZINC32603670	-36.6211	-35.2540
ZINC19771149	-36.6620	-35.2517
ZINC18096268	-47.2869	-35.2476
ZINC00674301	-37.3403	-35.2409
ZINC09008645	-40.3740	-35.2381
ZINC05488697	-39.0413	-35.2287
ZINC00665830	-43.9926	-35.2264
ZINC19883524	-42.4485	-35.2255
ZINC06136810	-37.4441	-35.2227
ZINC19938299	-38.2133	-35.2204
ZINC08439880	-36.5722	-35.2184
ZINC08438975	-41.2293	-35.2157
ZINC09124411	-44.0616	-35.2128
ZINC10232509	-37.3999	-35.2095
ZINC06195548	-37.7510	-35.2071
ZINC16944577	-41.1910	-35.2053
ZINC02088137	-38.8988	-35.2047
ZINC16671181	-36.8669	-35.2041
ZINC16671181	-36.7940	-35.2041
ZINC00631175	-44.5711	-35.2040
ZINC04067339	-41.3827	-35.1951
ZINC04067339	-38.8964	-35.1951
ZINC20477612	-39.0921	-35.1943
ZINC18089327	-38.1463	-35.1856
ZINC18089327	-36.8468	-35.1856
ZINC17180314	-44.8497	-35.1834
ZINC08995484	-38.9944	-35.1821
ZINC05433100	-39.4067	-35.1800
ZINC18208153	-37.9699	-35.1784
ZINC18208153	-37.8743	-35.1784
ZINC01248241	-37.1477	-35.1709
ZINC17139063	-42.0435	-35.1646
ZINC09273659	-44.1183	-35.1642
ZINC00703099	-40.2858	-35.1639
ZINC00703099	-39.8398	-35.1639
ZINC20233178	-45.3255	-35.1615
ZINC17166880	-36.2897	-35.1608
ZINC00778745	-45.0949	-35.1606
ZINC00661864	-37.5779	-35.1601
ZINC04416020	-37.1891	-35.1591
ZINC04416020	-36.8810	-35.1591
ZINC09042751	-43.1207	-35.1571
ZINC19815628	-36.5089	-35.1488
ZINC08425383	-36.7733	-35.1460
ZINC18118342	-46.4748	-35.1319
ZINC00988124	-40.6958	-35.1251
ZINC00620236	-39.2436	-35.1225
ZINC19938387	-44.8055	-35.1221
ZINC05749286	-37.7588	-35.1208
ZINC08969081	-36.9302	-35.1143
ZINC02057300	-38.9928	-35.1097
ZINC09008930	-43.1196	-35.1048
ZINC09008930	-43.1049	-35.1048

ZINC06162231	-37.8365	-35.0934
ZINC08413085	-36.5516	-35.0888
ZINC08413085	-36.3360	-35.0888
ZINC00674121	-38.9946	-35.0826
ZINC10232553	-39.0366	-35.0800
ZINC09358336	-47.4474	-35.0770
ZINC02573321	-36.4916	-35.0756
ZINC08439876	-37.6400	-35.0695
ZINC05409103	-36.8341	-35.0678
ZINC13800409	-40.2242	-35.0677
ZINC01002484	-39.5530	-35.0594
ZINC09311861	-36.6118	-35.0592
ZINC08986551	-38.7829	-35.0583
ZINC00726454	-39.0077	-35.0532
ZINC02140837	-43.7584	-35.0504
ZINC09008299	-44.7111	-35.0462
ZINC09008299	-42.6779	-35.0462
ZINC09008299	-41.2897	-35.0462
ZINC09008299	-40.1935	-35.0462
ZINC04278549	-42.6215	-35.0438
ZINC19796844	-36.8528	-35.0316
ZINC04065154	-39.2376	-35.0295
ZINC19938394	-44.1059	-35.0272
ZINC01213473	-43.0273	-35.0261
ZINC31853511	-37.0386	-35.0253
ZINC05425714	-37.4084	-35.0210
ZINC02237374	-36.5146	-35.0201
ZINC09178237	-39.5196	-35.0199
ZINC08425458	-49.3166	-35.0186
ZINC02262449	-37.2482	-35.0167
ZINC17179823	-40.9333	-35.0131
ZINC04997607	-45.3145	-35.0074
ZINC04997607	-36.7795	-35.0074
ZINC13286753	-40.6814	-35.0013
ZINC00959850	-38.0693	-35.0002
ZINC00959850	-37.7551	-35.0002
ZINC08996136	-37.0979	-34.9986
ZINC09008392	-40.7900	-34.9950
ZINC08437432	-47.3302	-34.9921
ZINC02484251	-48.0134	-34.9897
ZINC00645520	-41.7941	-34.9830
ZINC08430102	-41.9499	-34.9825
ZINC19923561	-36.7010	-34.9812
ZINC09175033	-38.1196	-34.9806
ZINC19370661	-43.2312	-34.9796
ZINC01004839	-40.8930	-34.9753
ZINC17160203	-41.1206	-34.9745
ZINC08413551	-37.4369	-34.9720
ZINC19832573	-48.9776	-34.9677
ZINC06144557	-39.1187	-34.9669
ZINC00703356	-41.9036	-34.9587
ZINC08686752	-42.0440	-34.9513
ZINC08686752	-38.3499	-34.9513
ZINC08686752	-36.8625	-34.9513

ZINC08425668	-37.3758	-34.9499
ZINC08425668	-36.8432	-34.9499
ZINC13125210	-37.4255	-34.9485
ZINC00215095	-36.7505	-34.9471
ZINC02807150	-38.6325	-34.9461
ZINC09008786	-41.0605	-34.9454
ZINC13607399	-40.6666	-34.9397
ZINC00630331	-36.7812	-34.9389
ZINC09411403	-37.6032	-34.9382
ZINC08714567	-37.2379	-34.9381
ZINC20057777	-37.0259	-34.9323
ZINC19313440	-36.2627	-34.9235
ZINC02191289	-38.8704	-34.9195
ZINC17160090	-43.3423	-34.9135
ZINC17160090	-41.5292	-34.9135
ZINC01661653	-40.2932	-34.9012
ZINC00384639	-37.0798	-34.8997
ZINC05014458	-38.8348	-34.8913
ZINC16667524	-37.8982	-34.8879
ZINC09370783	-36.8304	-34.8873
ZINC19808861	-38.3309	-34.8844
ZINC00870872	-38.9186	-34.8811
ZINC19370696	-39.2026	-34.8802
ZINC19973919	-44.9968	-34.8712
ZINC04123542	-36.9064	-34.8612
ZINC19908666	-36.9673	-34.8594
ZINC19908666	-36.6930	-34.8594
ZINC18210476	-38.2408	-34.8504
ZINC18210476	-36.4081	-34.8504
ZINC02055218	-39.3599	-34.8503
ZINC09089292	-37.2370	-34.8490
ZINC20263686	-42.1997	-34.8466
ZINC13569107	-42.7014	-34.8458
ZINC00673932	-36.5858	-34.8448
ZINC13139092	-46.2421	-34.8423
ZINC19889371	-36.2657	-34.8407
ZINC13769615	-37.2075	-34.8398
ZINC00842294	-38.1065	-34.8327
ZINC06195768	-38.2767	-34.8278
ZINC20264714	-44.8262	-34.8206
ZINC13387869	-41.3327	-34.8169
ZINC06498741	-40.7305	-34.8136
ZINC13555352	-38.7351	-34.8102
ZINC06137417	-36.3116	-34.8073
ZINC00673929	-37.9049	-34.8070
ZINC00828013	-36.3100	-34.8055
ZINC09174608	-41.6311	-34.8036
ZINC19814870	-37.9956	-34.7875
ZINC18181421	-37.8873	-34.7815
ZINC18181421	-36.2769	-34.7815
ZINC08715750	-37.5940	-34.7808
ZINC08715750	-36.4455	-34.7808
ZINC00641029	-38.6547	-34.7799
ZINC04189388	-37.9190	-34.7796

ZINC59796561	-43.7437	-34.7764
ZINC59796561	-38.6897	-34.7764
ZINC09460994	-38.9278	-34.7758
ZINC09460994	-37.3628	-34.7758
ZINC59796563	-43.8727	-34.7740
ZINC59796563	-38.5697	-34.7740
ZINC13139091	-45.3159	-34.7736
ZINC17750278	-36.9495	-34.7730
ZINC17750278	-36.6590	-34.7730
ZINC12649560	-40.6902	-34.7673
ZINC12649560	-39.7640	-34.7673
ZINC12438531	-39.3416	-34.7655
ZINC12438531	-39.2548	-34.7655
ZINC04719751	-36.5314	-34.7650
ZINC10232649	-37.0056	-34.7597
ZINC08846515	-40.6632	-34.7569
ZINC20027327	-41.7969	-34.7543
ZINC19938320	-38.1119	-34.7456
ZINC03876470	-39.4775	-34.7429
ZINC00627129	-42.6565	-34.7402
ZINC06499151	-40.8537	-34.7330
ZINC02982734	-39.1456	-34.7292
ZINC08462420	-38.1309	-34.7281
ZINC05445224	-38.9631	-34.7212
ZINC57724326	-37.9676	-34.7212
ZINC08437284	-36.6460	-34.7150
ZINC05898438	-38.0969	-34.7099
ZINC19790357	-37.1160	-34.7057
ZINC03991789	-49.0621	-34.7046
ZINC08845379	-40.1155	-34.7037
ZINC04648805	-38.0027	-34.6999
ZINC00683217	-43.1866	-34.6997
ZINC05724083	-38.4815	-34.6914
ZINC18083153	-48.1473	-34.6858
ZINC85433939	-38.2709	-34.6809
ZINC17196860	-43.8280	-34.6778
ZINC06162372	-36.4600	-34.6767
ZINC19894474	-41.3997	-34.6743
ZINC00984809	-37.0157	-34.6733
ZINC08397988	-40.4632	-34.6722
ZINC08397988	-37.1131	-34.6722
ZINC08426287	-37.5627	-34.6692
ZINC19923570	-36.7657	-34.6672
ZINC08836983	-43.6921	-34.6660
ZINC00657433	-47.3437	-34.6659
ZINC05409114	-36.3815	-34.6606
ZINC16267678	-37.7678	-34.6579
ZINC36646071	-36.3105	-34.6539
ZINC20308649	-39.2349	-34.6531
ZINC08462156	-42.7148	-34.6528
ZINC36646208	-39.9384	-34.6524
ZINC19855047	-39.7189	-34.6452
ZINC19904585	-40.2871	-34.6450
ZINC19815430	-38.0960	-34.6408

ZINC19904582	-42.5497	-34.6392
ZINC02594700	-37.2738	-34.6362
ZINC00879580	-42.0806	-34.6231
ZINC05483393	-36.6514	-34.6075
ZINC19841693	-37.3620	-34.6069
ZINC00678173	-36.5109	-34.6066
ZINC09308697	-44.5386	-34.6051
ZINC09308697	-42.1968	-34.6051
ZINC09308697	-41.9649	-34.6051
ZINC09280114	-37.8279	-34.5982
ZINC02064454	-39.7905	-34.5939
ZINC18244892	-39.0759	-34.5908
ZINC06195819	-38.3180	-34.5893
ZINC00625722	-36.8812	-34.5871
ZINC04285159	-39.9526	-34.5871
ZINC06791004	-44.3952	-34.5865
ZINC00678763	-39.7463	-34.5779
ZINC09271678	-44.9823	-34.5777
ZINC09271678	-41.7737	-34.5777
ZINC09044976	-36.2818	-34.5661
ZINC02055386	-39.5968	-34.5638
ZINC09186713	-43.0004	-34.5637
ZINC20166936	-44.8597	-34.5626
ZINC01016631	-36.4707	-34.5548
ZINC08913935	-38.1726	-34.5493
ZINC09071231	-44.8462	-34.5490
ZINC09071231	-41.7739	-34.5490
ZINC09071231	-41.2007	-34.5490
ZINC19972595	-44.2649	-34.5488
ZINC06266825	-44.2118	-34.5439
ZINC00913363	-38.5911	-34.5340
ZINC04188432	-38.5533	-34.5322
ZINC04067980	-36.3793	-34.5161
ZINC22790434	-37.4282	-34.5090
ZINC22790434	-36.9063	-34.5090
ZINC06088662	-42.6361	-34.5056
ZINC15919749	-37.6872	-34.4994
ZINC64635061	-41.2171	-34.4959
ZINC05634092	-39.4176	-34.4913
ZINC00631425	-36.7338	-34.4807
ZINC19872062	-44.4873	-34.4784
ZINC08435046	-36.5532	-34.4764
ZINC10313237	-43.8681	-34.4723
ZINC08440033	-38.5593	-34.4712
ZINC08440033	-37.6164	-34.4712
ZINC00384616	-39.1423	-34.4698
ZINC33347707	-44.5306	-34.4672
ZINC12420475	-40.4120	-34.4672
ZINC12420475	-37.6548	-34.4672
ZINC09271309	-40.0309	-34.4654
ZINC09271309	-39.9622	-34.4654
ZINC08440877	-39.3234	-34.4644
ZINC08440877	-39.2583	-34.4644
ZINC00205662	-36.9200	-34.4643

ZINC10232577	-38.9142	-34.4622
ZINC09272220	-39.0813	-34.4586
ZINC19943616	-40.9774	-34.4580
ZINC08433263	-39.2862	-34.4579
ZINC13122210	-42.6390	-34.4526
ZINC04993251	-36.3453	-34.4469
ZINC17166637	-39.9815	-34.4419
ZINC19815601	-37.3531	-34.4351
ZINC20264906	-43.4512	-34.4341
ZINC02064476	-38.4890	-34.4336
ZINC04116474	-36.4768	-34.4319
ZINC19823704	-37.8474	-34.4281
ZINC13427448	-38.0981	-34.4269
ZINC36646116	-40.8390	-34.4178
ZINC09089050	-38.9149	-34.4171
ZINC02096232	-41.0027	-34.4145
ZINC20308651	-46.2529	-34.4094
ZINC00821017	-38.7487	-34.4031
ZINC08715674	-37.8758	-34.4004
ZINC06744628	-40.3784	-34.3997
ZINC00719488	-43.0399	-34.3994
ZINC19168995	-37.1936	-34.3992
ZINC00755398	-38.5617	-34.3897
ZINC08438676	-41.8308	-34.3849
ZINC08438676	-41.6424	-34.3849
ZINC19938608	-36.6041	-34.3790
ZINC00792941	-37.8936	-34.3728
ZINC00792941	-36.5788	-34.3728
ZINC09186740	-41.1175	-34.3629
ZINC20265383	-41.8397	-34.3590
ZINC00657609	-40.6831	-34.3586
ZINC00657609	-40.4446	-34.3586
ZINC19972598	-43.0195	-34.3575
ZINC17145507	-36.5341	-34.3542
ZINC17145507	-36.3107	-34.3542
ZINC00623635	-36.8527	-34.3504
ZINC09363562	-36.6712	-34.3481
ZINC05409113	-37.0567	-34.3470
ZINC18084743	-38.9368	-34.3455
ZINC19849551	-40.4257	-34.3442
ZINC12647469	-38.0192	-34.3405
ZINC05014440	-37.1881	-34.3384
ZINC00979604	-46.7189	-34.3309
ZINC00711748	-37.2096	-34.3307
ZINC19872407	-37.3109	-34.3287
ZINC02179797	-37.6690	-34.3266
ZINC00645289	-43.6187	-34.3225
ZINC05285420	-40.2758	-34.3111
ZINC08462848	-39.4458	-34.3086
ZINC08462848	-38.0714	-34.3086
ZINC05678881	-37.2766	-34.3049
ZINC05678881	-36.7137	-34.3049
ZINC00673376	-36.4025	-34.2970
ZINC13549232	-48.2066	-34.2903

ZINC13108602	-38.1686	-34.2798
ZINC59796562	-41.6313	-34.2678
ZINC59796562	-39.1352	-34.2678
ZINC00669857	-40.7876	-34.2628
ZINC68739424	-41.4774	-34.2461
ZINC19638220	-39.6906	-34.2450
ZINC05408991	-37.9912	-34.2313
ZINC19872072	-43.6826	-34.2273
ZINC09370625	-36.5435	-34.2201
ZINC19818288	-38.1718	-34.2171
ZINC02182515	-45.5037	-34.2089
ZINC19535919	-38.2380	-34.2063
ZINC09007850	-39.9674	-34.2029
ZINC19973921	-44.8216	-34.2024
ZINC00702700	-39.1316	-34.1971
ZINC00702700	-37.7585	-34.1971
ZINC00478740	-37.7839	-34.1935
ZINC08985232	-40.9212	-34.1884
ZINC08985232	-40.8873	-34.1884
ZINC08985232	-40.8101	-34.1884
ZINC08985232	-40.3918	-34.1884
ZINC09436810	-39.1963	-34.1875
ZINC08715739	-36.3645	-34.1812
ZINC19872021	-38.4566	-34.1779
ZINC08450379	-44.0274	-34.1712
ZINC00869721	-37.0523	-34.1627
ZINC08438524	-40.6264	-34.1587
ZINC06144562	-37.8172	-34.1586
ZINC08450308	-38.5889	-34.1581
ZINC04391377	-36.5825	-34.1514
ZINC19938438	-43.1674	-34.1510
ZINC09272726	-38.5879	-34.1449
ZINC19883116	-41.2930	-34.1355
ZINC19848654	-38.0507	-34.1355
ZINC13108612	-44.8063	-34.1324
ZINC19815423	-38.1473	-34.1313
ZINC00702427	-40.8865	-34.1313
ZINC00702427	-39.6804	-34.1313
ZINC09007671	-40.3689	-34.1297
ZINC09007671	-40.1858	-34.1297
ZINC00086323	-38.3272	-34.1266
ZINC09471626	-37.5419	-34.1234
ZINC06720039	-37.3525	-34.1118
ZINC00631434	-37.1846	-34.1093
ZINC18056643	-37.6745	-34.0971
ZINC18056643	-36.3989	-34.0971
ZINC36046870	-38.0080	-34.0920
ZINC19923586	-37.1680	-34.0869
ZINC18247110	-37.4577	-34.0758
ZINC18247110	-37.0940	-34.0758
ZINC18247110	-36.3082	-34.0758
ZINC00669854	-44.9835	-34.0684
ZINC02063428	-40.0304	-34.0652
ZINC19815454	-39.5903	-34.0643

ZINC19815454	-37.4109	-34.0643
ZINC19536337	-37.6473	-34.0570
ZINC00677813	-40.0296	-34.0484
ZINC68740954	-39.2024	-34.0405
ZINC08424903	-37.8544	-34.0393
ZINC08424903	-37.4555	-34.0393
ZINC02055378	-37.9021	-34.0365
ZINC00865832	-38.7665	-34.0336
ZINC18245085	-38.9812	-34.0326
ZINC17207295	-41.5788	-34.0240
ZINC17207295	-41.0838	-34.0240
ZINC17207295	-40.8275	-34.0240
ZINC17207295	-40.1831	-34.0240
ZINC17207295	-40.1053	-34.0240
ZINC17207295	-39.4963	-34.0240
ZINC00710977	-37.3966	-34.0239
ZINC35647441	-38.5754	-34.0079
ZINC04385859	-38.4943	-34.0049
ZINC06444779	-39.9451	-34.0045
ZINC10297558	-36.6171	-34.0033
ZINC00680064	-42.6812	-33.9985
ZINC04913680	-38.3347	-33.9979
ZINC35610593	-37.1620	-33.9953
ZINC19990597	-37.9554	-33.9938
ZINC36532504	-43.2727	-33.9902
ZINC36532504	-36.4240	-33.9902
ZINC08996873	-43.8175	-33.9735
ZINC08996873	-41.8080	-33.9735
ZINC08996873	-38.9773	-33.9735
ZINC05095364	-37.3724	-33.9720
ZINC05432861	-38.2002	-33.9707
ZINC17143892	-37.9498	-33.9697
ZINC08715684	-38.1326	-33.9535
ZINC08715684	-36.9825	-33.9535
ZINC06565156	-39.0992	-33.9492
ZINC00898290	-46.6987	-33.9455
ZINC12849891	-42.2949	-33.9323
ZINC08441924	-38.6530	-33.9322
ZINC08430507	-36.3868	-33.9235
ZINC13570637	-37.9952	-33.9156
ZINC13570637	-37.6933	-33.9156
ZINC04387659	-39.1049	-33.9132
ZINC13523424	-37.3640	-33.9066
ZINC08439750	-40.1928	-33.9026
ZINC08439750	-38.2103	-33.9026
ZINC06265567	-38.3098	-33.9013
ZINC33700725	-39.2039	-33.8976
ZINC06015630	-37.5012	-33.8928
ZINC20190957	-40.5384	-33.8921
ZINC08442195	-39.7041	-33.8915
ZINC05448636	-36.2904	-33.8835
ZINC00414574	-36.6107	-33.8826
ZINC18155005	-37.7059	-33.8788
ZINC04473070	-37.4917	-33.8755

ZINC19872218	-43.0368	-33.8738
ZINC59796560	-38.7870	-33.8683
ZINC59796560	-37.7843	-33.8683
ZINC08739160	-43.6489	-33.8681
ZINC08739160	-43.0648	-33.8681
ZINC08739160	-42.5192	-33.8681
ZINC08739160	-41.5111	-33.8681
ZINC08739160	-40.9488	-33.8681
ZINC09041905	-37.9522	-33.8665
ZINC09009423	-39.3348	-33.8538
ZINC01018803	-47.1830	-33.8393
ZINC08385558	-40.5953	-33.8390
ZINC00725692	-36.5953	-33.8297
ZINC00725692	-36.5904	-33.8297
ZINC00680547	-38.9071	-33.8214
ZINC05446330	-36.3055	-33.8192
ZINC04019614	-39.0588	-33.8160
ZINC04019614	-38.0661	-33.8160
ZINC36636397	-37.3283	-33.8141
ZINC37857376	-37.6782	-33.8133
ZINC19637669	-39.7359	-33.7924
ZINC19815623	-38.4108	-33.7920
ZINC06015626	-36.4092	-33.7906
ZINC19872381	-39.2364	-33.7849
ZINC19938753	-38.5595	-33.7824
ZINC06279468	-39.7171	-33.7817
ZINC08845428	-42.5079	-33.7769
ZINC02064452	-36.9018	-33.7717
ZINC04950661	-37.2403	-33.7693
ZINC04950661	-37.2269	-33.7693
ZINC22972099	-36.6819	-33.7660
ZINC00983812	-37.8189	-33.7465
ZINC13147513	-40.6186	-33.7411
ZINC71405092	-46.0133	-33.7410
ZINC00702484	-37.5691	-33.7384
ZINC17180316	-44.7684	-33.7348
ZINC06142848	-43.4473	-33.7267
ZINC06144545	-38.8854	-33.7261
ZINC06645884	-41.3788	-33.7245
ZINC09043098	-45.1661	-33.7113
ZINC09043098	-42.0597	-33.7113
ZINC09043098	-41.9124	-33.7113
ZINC04284996	-39.6653	-33.7087
ZINC04284996	-38.6908	-33.7087
ZINC33286213	-50.2171	-33.6991
ZINC04285175	-37.4964	-33.6947
ZINC17720680	-38.1315	-33.6934
ZINC09109346	-39.8896	-33.6850
ZINC00674230	-38.5829	-33.6824
ZINC00674230	-36.4953	-33.6824
ZINC08462798	-36.6777	-33.6821
ZINC06415699	-39.3590	-33.6819
ZINC19923563	-38.8999	-33.6801
ZINC18037906	-37.4842	-33.6792

ZINC00164067	-36.8333	-33.6693
ZINC00898203	-40.3729	-33.6686
ZINC08714996	-41.1409	-33.6682
ZINC13108317	-46.1172	-33.6634
ZINC08715771	-36.4143	-33.6593
ZINC12340508	-37.4025	-33.6435
ZINC02690793	-41.2964	-33.6395
ZINC04714430	-38.8785	-33.6364
ZINC00634096	-42.5682	-33.6330
ZINC00629649	-36.9600	-33.6312
ZINC08817407	-36.9775	-33.6298
ZINC13122027	-42.2480	-33.6191
ZINC19923139	-43.1207	-33.6022
ZINC01029871	-38.7830	-33.6022
ZINC00662920	-37.4126	-33.5937
ZINC19853448	-39.1554	-33.5902
ZINC19872424	-36.5586	-33.5891
ZINC33794603	-36.6620	-33.5871
ZINC17118209	-39.0953	-33.5857
ZINC20263689	-43.6695	-33.5853
ZINC08438520	-40.9355	-33.5849
ZINC04714431	-38.5951	-33.5788
ZINC00481078	-41.6470	-33.5769
ZINC13108208	-41.7072	-33.5747
ZINC08440071	-38.2885	-33.5737
ZINC08440071	-37.0854	-33.5737
ZINC06267887	-41.5301	-33.5683
ZINC12403802	-43.7755	-33.5669
ZINC06162516	-36.2598	-33.5581
ZINC05408980	-37.5888	-33.5564
ZINC04452815	-37.0579	-33.5437
ZINC06501832	-37.5651	-33.5419
ZINC06501832	-36.4264	-33.5419
ZINC04398438	-40.1052	-33.5405
ZINC05408798	-40.6404	-33.5342
ZINC05408798	-40.1079	-33.5342
ZINC05408798	-39.7847	-33.5342
ZINC02772831	-39.1570	-33.5298
ZINC02772831	-37.4851	-33.5298
ZINC00727313	-41.7249	-33.5225
ZINC04065654	-38.2426	-33.5182
ZINC04065654	-37.9951	-33.5182
ZINC04065654	-37.4123	-33.5182
ZINC06013560	-41.1116	-33.5164
ZINC05045112	-37.3434	-33.5160
ZINC17242901	-36.2817	-33.5124
ZINC05819168	-37.9024	-33.4997
ZINC23138388	-36.4153	-33.4990
ZINC06144543	-40.4178	-33.4990
ZINC08714624	-39.9344	-33.4970
ZINC04038704	-36.7835	-33.4901
ZINC01891135	-42.6987	-33.4879
ZINC17242372	-39.1709	-33.4865
ZINC05444591	-39.0839	-33.4859

ZINC00632601	-44.7244	-33.4799
ZINC00704133	-42.3318	-33.4781
ZINC08442210	-37.3674	-33.4756
ZINC13161474	-40.8886	-33.4624
ZINC00995241	-36.8949	-33.4597
ZINC13553033	-43.5830	-33.4579
ZINC13553033	-41.2018	-33.4579
ZINC13553033	-40.6624	-33.4579
ZINC18115123	-36.4948	-33.4499
ZINC19637675	-38.6892	-33.4485
ZINC00384615	-36.5169	-33.4476
ZINC02180694	-45.3775	-33.4397
ZINC06786122	-36.4490	-33.4360
ZINC02078236	-51.2141	-33.4318
ZINC17147577	-36.5662	-33.4219
ZINC08400609	-43.0740	-33.4219
ZINC13003362	-42.1775	-33.4133
ZINC05446025	-44.1773	-33.4128
ZINC05446025	-39.0959	-33.4128
ZINC10296967	-36.5500	-33.4073
ZINC09471237	-38.1969	-33.3998
ZINC00676984	-39.6778	-33.3983
ZINC19872471	-37.4013	-33.3978
ZINC17201320	-44.2188	-33.3973
ZINC04189387	-40.9026	-33.3922
ZINC09166429	-40.3944	-33.3917
ZINC49765325	-36.3757	-33.3838
ZINC09046788	-44.7971	-33.3817
ZINC13712103	-40.2659	-33.3808
ZINC00844151	-37.0471	-33.3751
ZINC08624987	-37.3325	-33.3725
ZINC04060978	-37.9780	-33.3642
ZINC04060978	-37.8251	-33.3642
ZINC04060978	-37.6862	-33.3642
ZINC19938650	-44.9852	-33.3628
ZINC19938650	-44.4670	-33.3628
ZINC19938650	-43.9114	-33.3628
ZINC10297428	-36.3292	-33.3627
ZINC02029640	-39.8165	-33.3612
ZINC00715097	-37.0545	-33.3610
ZINC00715097	-36.7172	-33.3610
ZINC02486583	-37.7860	-33.3457
ZINC12647569	-36.6170	-33.3453
ZINC05433103	-39.0788	-33.3418
ZINC02064464	-39.9145	-33.3391
ZINC13555329	-38.4819	-33.3359
ZINC02061083	-37.2962	-33.3336
ZINC16579312	-37.3288	-33.3272
ZINC08462844	-38.1418	-33.3226
ZINC05956143	-38.0263	-33.3197
ZINC02191053	-37.2590	-33.3170
ZINC17167282	-36.7705	-33.3170
ZINC06142487	-38.8011	-33.3059
ZINC06142487	-36.8339	-33.3059

ZINC06144558	-42.2731	-33.3040
ZINC33248652	-41.3905	-33.2908
ZINC15974450	-38.0218	-33.2900
ZINC15974450	-37.6973	-33.2900
ZINC08440128	-37.1115	-33.2870
ZINC19938752	-38.8445	-33.2844
ZINC05818318	-37.4007	-33.2776
ZINC18140529	-42.3283	-33.2708
ZINC06826170	-37.8186	-33.2676
ZINC08715687	-38.2577	-33.2646
ZINC08715687	-36.7850	-33.2646
ZINC00822331	-37.0672	-33.2600
ZINC00844166	-37.3547	-33.2443
ZINC06343890	-37.0663	-33.2439
ZINC13124770	-36.7110	-33.2414
ZINC08715712	-36.3769	-33.2383
ZINC05423626	-38.3751	-33.2265
ZINC06136913	-36.6297	-33.2222
ZINC01890654	-38.9961	-33.2082
ZINC00168698	-40.4436	-33.2057
ZINC19883526	-38.5504	-33.2020
ZINC19831139	-38.4571	-33.1942
ZINC00625459	-38.8107	-33.1930
ZINC20057670	-43.4767	-33.1884
ZINC05409117	-36.9249	-33.1880
ZINC08437218	-42.0755	-33.1802
ZINC19370591	-46.7634	-33.1743
ZINC19370591	-46.6468	-33.1743
ZINC19370591	-46.2298	-33.1743
ZINC04135732	-36.8483	-33.1722
ZINC19535922	-38.3964	-33.1500
ZINC05919718	-37.7343	-33.1483
ZINC04649800	-36.9793	-33.1483
ZINC06863366	-36.3155	-33.1430
ZINC08385646	-38.6942	-33.1300
ZINC08838014	-36.7180	-33.1179
ZINC04123550	-36.4555	-33.1160
ZINC02135746	-44.1627	-33.1126
ZINC00631127	-42.1499	-33.1104
ZINC04187539	-39.3140	-33.1071
ZINC04187539	-38.5304	-33.1071
ZINC04187539	-37.9660	-33.1071
ZINC06744637	-41.5930	-33.1040
ZINC19904272	-38.2929	-33.1039
ZINC04937439	-38.3990	-33.1037
ZINC20189621	-41.4137	-33.0998
ZINC36646203	-41.0979	-33.0997
ZINC36646203	-39.5243	-33.0997
ZINC15989794	-39.7380	-33.0966
ZINC23114588	-37.0548	-33.0944
ZINC19872152	-37.3343	-33.0819
ZINC17193782	-40.3188	-33.0776
ZINC00631164	-40.6904	-33.0749
ZINC13387870	-41.3772	-33.0664

ZINC07425018	-39.7229	-33.0619
ZINC05422096	-36.3480	-33.0584
ZINC08743884	-39.5150	-33.0552
ZINC16944582	-41.2029	-33.0552
ZINC18059304	-39.6580	-33.0522
ZINC01029865	-39.6713	-33.0458
ZINC08744479	-37.6426	-33.0426
ZINC12437331	-46.5399	-33.0382
ZINC12437331	-46.4469	-33.0382
ZINC12437331	-43.3739	-33.0382
ZINC00702414	-37.7551	-33.0361
ZINC13590323	-36.8280	-33.0310
ZINC08462834	-38.4537	-33.0230
ZINC00625888	-38.3688	-33.0217
ZINC17970637	-38.2633	-33.0166
ZINC10313182	-42.8602	-33.0090
ZINC00672142	-44.0796	-32.9929
ZINC00672142	-43.9898	-32.9929
ZINC06269430	-40.9677	-32.9854
ZINC02061084	-37.4861	-32.9747
ZINC13555217	-41.3775	-32.9691
ZINC13555217	-39.7103	-32.9691
ZINC13555217	-39.1365	-32.9691
ZINC13555217	-38.2700	-32.9691
ZINC00432262	-39.8473	-32.9604
ZINC06025645	-40.0736	-32.9562
ZINC19904270	-38.5322	-32.9482
ZINC04113089	-38.1366	-32.9463
ZINC04113089	-37.9031	-32.9463
ZINC04113089	-37.0888	-32.9463
ZINC09411171	-37.9944	-32.9463
ZINC09411171	-36.4471	-32.9463
ZINC00672202	-42.9038	-32.9456
ZINC00672202	-42.6125	-32.9456
ZINC03900884	-42.6319	-32.9398
ZINC36638917	-39.5132	-32.9389
ZINC04387663	-40.7522	-32.9362
ZINC08411523	-37.1919	-32.9320
ZINC17194605	-42.7863	-32.9251
ZINC17194605	-42.7278	-32.9251
ZINC35592601	-39.0130	-32.9236
ZINC12403696	-38.2381	-32.9154
ZINC09008391	-38.9781	-32.9145
ZINC02082128	-41.2556	-32.9081
ZINC08817408	-36.5215	-32.9062
ZINC02523219	-38.5586	-32.8905
ZINC08439647	-42.6298	-32.8862
ZINC02055496	-40.1988	-32.8848
ZINC07425015	-43.6267	-32.8837
ZINC00384638	-38.8470	-32.8759
ZINC20066003	-42.3172	-32.8722
ZINC04067363	-39.7062	-32.8702
ZINC04067363	-38.3573	-32.8702
ZINC06195778	-38.8138	-32.8638

ZINC06195778	-36.5863	-32.8638
ZINC09073464	-37.3504	-32.8633
ZINC19938750	-40.2166	-32.8563
ZINC04247809	-36.4574	-32.8518
ZINC68732608	-37.0792	-32.8476
ZINC12427912	-38.1636	-32.8452
ZINC08396326	-36.5800	-32.8446
ZINC00629502	-43.6715	-32.8426
ZINC20025551	-42.4428	-32.8384
ZINC00642383	-38.9324	-32.8356
ZINC00728200	-37.5051	-32.8346
ZINC08440404	-42.4509	-32.8295
ZINC19904267	-37.7593	-32.8287
ZINC19815592	-38.2398	-32.8243
ZINC00702714	-38.2890	-32.8123
ZINC00702714	-36.9593	-32.8123
ZINC08424901	-40.3419	-32.8106
ZINC08424901	-39.4976	-32.8106
ZINC08424901	-39.4689	-32.8106
ZINC08455447	-39.9383	-32.8067
ZINC25107868	-40.2826	-32.8053
ZINC25107868	-38.8068	-32.8053
ZINC19909101	-40.3326	-32.7947
ZINC05316280	-37.4674	-32.7906
ZINC05409043	-36.7556	-32.7854
ZINC00673800	-39.4991	-32.7837
ZINC00384751	-38.5878	-32.7639
ZINC00384751	-37.5458	-32.7639
ZINC04666060	-38.6924	-32.7552
ZINC19904278	-38.1727	-32.7483
ZINC19535834	-40.1111	-32.7416
ZINC00683220	-38.6569	-32.7312
ZINC19848755	-44.5547	-32.7306
ZINC18213575	-37.6344	-32.7289
ZINC13108316	-48.6919	-32.7281
ZINC20030837	-37.3503	-32.7218
ZINC19904600	-37.2405	-32.7216
ZINC19884885	-41.4792	-32.7192
ZINC12378357	-51.1851	-32.7183
ZINC12378357	-49.2212	-32.7183
ZINC17179834	-41.1634	-32.7157
ZINC04452955	-37.8560	-32.7150
ZINC02890521	-40.2070	-32.7150
ZINC05447825	-36.7067	-32.7045
ZINC19320269	-36.8654	-32.6937
ZINC19320269	-36.4444	-32.6937
ZINC08437221	-38.6334	-32.6900
ZINC08437174	-38.0934	-32.6891
ZINC09272879	-37.5410	-32.6842
ZINC09272879	-36.3463	-32.6842
ZINC19972597	-42.4957	-32.6722
ZINC08437147	-39.2658	-32.6586
ZINC10034872	-42.9176	-32.6490
ZINC02135768	-45.3078	-32.6486

ZINC08430354	-36.5755	-32.6442
ZINC08430354	-36.5618	-32.6442
ZINC00719486	-43.0811	-32.6364
ZINC19884791	-39.4105	-32.6313
ZINC12464468	-38.1650	-32.6295
ZINC19848656	-38.5959	-32.6272
ZINC04950664	-37.0473	-32.6188
ZINC08396624	-41.0130	-32.6082
ZINC00654077	-41.2226	-32.6041
ZINC05790764	-39.2094	-32.5991
ZINC00850871	-36.4479	-32.5967
ZINC08440699	-38.7324	-32.5914
ZINC09067283	-44.7842	-32.5892
ZINC09067283	-43.1631	-32.5892
ZINC40715624	-38.1423	-32.5802
ZINC06744603	-40.9273	-32.5716
ZINC04301176	-44.8876	-32.5704
ZINC04301176	-44.6921	-32.5704
ZINC01795563	-38.4410	-32.5675
ZINC19883112	-42.3943	-32.5572
ZINC09175855	-38.3418	-32.5556
ZINC00661401	-40.0789	-32.5532
ZINC09704030	-37.9040	-32.5518
ZINC08715658	-43.6674	-32.5495
ZINC08715658	-42.1758	-32.5495
ZINC08715658	-41.2274	-32.5495
ZINC08715658	-40.8252	-32.5495
ZINC08715658	-37.5613	-32.5495
ZINC06014794	-37.8258	-32.5428
ZINC18210470	-36.3704	-32.5401
ZINC19550260	-37.8804	-32.5366
ZINC37384318	-38.8311	-32.5186
ZINC06328363	-40.2445	-32.5175
ZINC18276311	-37.5357	-32.5175
ZINC15961944	-38.2621	-32.5136
ZINC15961944	-36.5370	-32.5136
ZINC00625768	-36.8100	-32.5099
ZINC00142321	-43.7033	-32.5066
ZINC19904602	-36.6151	-32.5056
ZINC19938751	-36.4116	-32.5008
ZINC23114599	-37.7986	-32.4816
ZINC19815620	-37.5768	-32.4764
ZINC04113087	-37.1329	-32.4502
ZINC04113087	-36.9553	-32.4502
ZINC05818089	-36.8838	-32.4335
ZINC08430604	-44.1245	-32.4277
ZINC27823885	-37.6896	-32.4245
ZINC01448657	-39.7096	-32.4127
ZINC02069391	-39.6766	-32.4099
ZINC08398430	-46.5529	-32.4040
ZINC00360847	-36.9371	-32.3909
ZINC00342376	-43.6126	-32.3845
ZINC00986419	-44.2020	-32.3756
ZINC09302190	-43.0859	-32.3729

ZINC09302190	-39.6528	-32.3729
ZINC07344219	-37.0507	-32.3627
ZINC00631162	-38.9377	-32.3561
ZINC05432854	-37.4278	-32.3521
ZINC18164065	-36.5870	-32.3513
ZINC12966955	-37.4683	-32.3428
ZINC04631270	-37.4193	-32.3390
ZINC68740868	-39.4744	-32.3244
ZINC17440259	-38.3881	-32.3211
ZINC20265270	-41.6363	-32.3152
ZINC00865769	-37.7674	-32.3127
ZINC00645150	-41.4921	-32.3093
ZINC00727544	-36.6382	-32.2960
ZINC19923148	-43.2994	-32.2956
ZINC02187356	-37.5915	-32.2921
ZINC19371211	-36.4685	-32.2905
ZINC19371211	-36.3715	-32.2905
ZINC08396468	-37.1162	-32.2792
ZINC19904286	-37.4441	-32.2782
ZINC13800112	-39.8686	-32.2699
ZINC09176102	-41.1247	-32.2659
ZINC00374130	-39.7206	-32.2616
ZINC19897646	-40.9484	-32.2612
ZINC17251526	-40.3577	-32.2529
ZINC05764273	-40.3842	-32.2485
ZINC27824362	-40.2137	-32.2465
ZINC17112011	-36.9636	-32.2463
ZINC02074720	-40.2645	-32.2373
ZINC18189330	-37.3794	-32.2359
ZINC18189330	-36.5258	-32.2359
ZINC02061189	-37.7299	-32.2353
ZINC00674173	-37.7176	-32.2342
ZINC05409052	-36.5118	-32.2290
ZINC08436919	-39.2739	-32.2286
ZINC00657629	-46.3441	-32.2251
ZINC06796986	-38.1618	-32.2245
ZINC02064455	-40.5186	-32.2235
ZINC05408974	-36.8509	-32.2229
ZINC05408974	-36.8372	-32.2229
ZINC05408974	-36.5039	-32.2229
ZINC00476649	-40.0279	-32.2171
ZINC08707054	-37.4160	-32.2054
ZINC17161270	-38.8125	-32.2052
ZINC05121237	-39.5235	-32.1995
ZINC09241178	-37.7498	-32.1956
ZINC08714686	-38.5672	-32.1909
ZINC08396394	-39.3383	-32.1830
ZINC00048540	-38.5263	-32.1778
ZINC06087946	-40.5661	-32.1625
ZINC06087946	-38.0901	-32.1625
ZINC19848754	-43.0192	-32.1379
ZINC18322566	-39.1444	-32.1344
ZINC04472973	-40.3078	-32.1250
ZINC19853443	-37.4796	-32.1245

ZINC19853443	-37.4634	-32.1245
ZINC20264909	-43.4344	-32.1200
ZINC00694911	-40.6333	-32.1035
ZINC02055230	-40.2545	-32.0981
ZINC19909446	-40.1522	-32.0973
ZINC13552609	-41.5754	-32.0921
ZINC13552609	-40.9666	-32.0921
ZINC02755343	-38.4258	-32.0891
ZINC08437203	-41.3911	-32.0812
ZINC16719036	-38.8662	-32.0769
ZINC19904274	-37.2706	-32.0753
ZINC01120533	-39.9458	-32.0740
ZINC08492399	-37.0226	-32.0699
ZINC20262942	-42.9473	-32.0643
ZINC08837080	-40.6426	-32.0603
ZINC00986246	-41.5573	-32.0542
ZINC05343446	-41.7303	-32.0502
ZINC05343446	-41.6973	-32.0502
ZINC02585253	-37.4876	-32.0343
ZINC75286725	-43.8633	-32.0287
ZINC09370781	-37.5387	-32.0136
ZINC08612303	-43.1637	-32.0134
ZINC04396876	-37.7997	-31.9974
ZINC08612302	-42.0023	-31.9829
ZINC02179799	-38.1877	-31.9756
ZINC00678620	-37.8218	-31.9637
ZINC08845378	-38.9483	-31.9556
ZINC17015165	-36.8660	-31.9550
ZINC19849690	-39.7844	-31.9543
ZINC08441892	-39.1669	-31.9438
ZINC00645521	-37.6495	-31.9402
ZINC00714273	-46.2488	-31.9256
ZINC05819156	-36.9769	-31.9251
ZINC05819156	-36.3264	-31.9251
ZINC02157655	-44.0008	-31.9214
ZINC08439331	-48.2827	-31.9138
ZINC04666069	-37.7959	-31.9079
ZINC08437172	-36.9708	-31.9060
ZINC13497475	-39.4403	-31.9045
ZINC19898769	-40.1161	-31.8868
ZINC17163315	-41.8209	-31.8849
ZINC19851208	-39.5594	-31.8796
ZINC13108526	-42.9025	-31.8727
ZINC17136207	-38.8878	-31.8625
ZINC06088800	-44.2376	-31.8606
ZINC09186516	-41.3030	-31.8531
ZINC09186516	-40.3708	-31.8531
ZINC11613756	-42.6384	-31.8392
ZINC13115253	-38.0511	-31.8235
ZINC01059888	-43.7121	-31.8233
ZINC20018545	-37.7332	-31.8123
ZINC08439794	-36.4446	-31.7940
ZINC08439794	-36.4400	-31.7940
ZINC19923136	-41.9494	-31.7921

ZINC13111465	-37.2114	-31.7910
ZINC00666914	-38.6576	-31.7906
ZINC19883924	-41.4944	-31.7869
ZINC05409045	-37.4125	-31.7663
ZINC05409045	-36.3902	-31.7663
ZINC00978663	-41.1052	-31.7625
ZINC19898504	-39.9250	-31.7480
ZINC00857738	-40.6219	-31.7441
ZINC00665133	-37.8773	-31.7431
ZINC19370664	-42.9923	-31.7277
ZINC08430219	-36.5637	-31.7080
ZINC01118536	-41.7024	-31.7008
ZINC02858492	-38.0873	-31.6999
ZINC00647195	-39.8546	-31.6972
ZINC02406745	-38.2421	-31.6922
ZINC00651513	-38.9978	-31.6847
ZINC00625528	-39.2527	-31.6663
ZINC01164767	-42.6610	-31.6631
ZINC16651132	-40.0515	-31.6556
ZINC04472970	-44.6597	-31.6555
ZINC00672190	-42.0260	-31.6456
ZINC00672190	-41.4956	-31.6456
ZINC00917819	-44.9675	-31.6419
ZINC19898488	-39.9281	-31.6412
ZINC06400002	-37.3859	-31.6326
ZINC08973017	-40.3612	-31.6277
ZINC03656737	-49.7819	-31.6244
ZINC01212938	-41.1067	-31.6179
ZINC01212938	-40.7143	-31.6179
ZINC13111120	-37.3922	-31.6080
ZINC02053312	-39.3789	-31.6019
ZINC09046919	-38.7676	-31.5947
ZINC00717443	-38.2616	-31.5904
ZINC16647994	-40.5682	-31.5879
ZINC19898486	-38.9925	-31.5822
ZINC04130707	-44.5896	-31.5608
ZINC00801103	-40.6555	-31.5551
ZINC00991157	-39.2412	-31.5492
ZINC05042908	-36.6348	-31.5465
ZINC17161266	-41.2051	-31.5446
ZINC19815607	-38.2336	-31.5415
ZINC00645306	-41.3474	-31.5307
ZINC02184792	-43.4456	-31.5049
ZINC08462286	-38.3557	-31.4908
ZINC35980680	-37.9898	-31.4822
ZINC00630466	-40.5381	-31.4628
ZINC08438565	-36.9450	-31.4571
ZINC08438565	-36.5473	-31.4571
ZINC05295614	-36.8660	-31.4471
ZINC06270521	-42.0801	-31.4393
ZINC05408972	-37.0772	-31.4325
ZINC34902397	-42.1222	-31.4321
ZINC64635059	-40.4639	-31.4298
ZINC13479762	-37.9338	-31.4213

ZINC12343477	-42.3198	-31.4203
ZINC48044477	-40.0903	-31.4185
ZINC19569408	-41.0341	-31.4119
ZINC13123143	-43.8884	-31.4014
ZINC17201399	-39.5059	-31.3618
ZINC17201399	-39.0150	-31.3618
ZINC17201399	-36.8544	-31.3618
ZINC00249618	-38.1365	-31.3482
ZINC05409050	-36.7802	-31.3413
ZINC19883926	-39.0826	-31.3318
ZINC00709207	-38.6345	-31.3315
ZINC00709207	-38.2327	-31.3315
ZINC05408996	-36.6423	-31.3314
ZINC18080512	-39.5613	-31.3263
ZINC06270551	-38.8234	-31.3177
ZINC00387190	-37.4073	-31.3024
ZINC09002156	-39.6134	-31.2989
ZINC00625729	-36.2971	-31.2982
ZINC02503983	-36.9211	-31.2974
ZINC02503983	-36.5730	-31.2974
ZINC00844223	-40.3537	-31.2965
ZINC17139065	-39.4449	-31.2904
ZINC05432855	-37.3330	-31.2849
ZINC13127642	-42.2151	-31.2754
ZINC02406746	-37.5058	-31.2628
ZINC68740841	-37.5167	-31.2614
ZINC00651515	-36.8473	-31.2591
ZINC05446023	-39.0625	-31.2496
ZINC19872066	-43.0774	-31.2359
ZINC00670556	-41.1939	-31.2217
ZINC08438533	-38.4172	-31.2186
ZINC06744597	-39.1518	-31.2100
ZINC19832302	-39.0708	-31.2097
ZINC19832302	-37.6909	-31.2097
ZINC00987886	-37.2967	-31.2089
ZINC05445213	-37.5683	-31.2088
ZINC00135634	-39.6751	-31.1876
ZINC00646952	-37.2238	-31.1838
ZINC00843541	-36.7922	-31.1818
ZINC19884887	-41.0872	-31.1779
ZINC05042910	-36.4065	-31.1565
ZINC19898506	-41.7893	-31.1472
ZINC19923436	-40.6445	-31.1452
ZINC00380211	-37.8391	-31.1443
ZINC16649565	-37.0271	-31.1343
ZINC33291604	-37.8637	-31.1157
ZINC04631640	-37.2144	-31.1130
ZINC02055264	-39.5160	-31.1052
ZINC04649167	-36.5118	-31.0907
ZINC00702713	-36.4574	-31.0750
ZINC06198398	-37.7498	-31.0565
ZINC15930483	-37.8847	-31.0513
ZINC15930483	-37.8344	-31.0513
ZINC15930483	-37.0206	-31.0513

ZINC15930483	-36.6487	-31.0513
ZINC15930483	-36.4651	-31.0513
ZINC05427265	-40.2156	-31.0496
ZINC19911014	-39.2053	-31.0472
ZINC05118781	-40.9785	-31.0402
ZINC00869525	-42.0958	-31.0340
ZINC12032316	-38.4205	-31.0337
ZINC12032316	-37.7435	-31.0337
ZINC17194602	-38.4039	-31.0071
ZINC04698406	-41.6553	-31.0033
ZINC00646428	-37.3121	-31.0017
ZINC08716943	-43.2950	-30.9995
ZINC08716943	-39.9600	-30.9995
ZINC08716943	-39.7572	-30.9995
ZINC19884756	-38.0299	-30.9974
ZINC00944360	-39.2089	-30.9961
ZINC00642384	-38.4237	-30.9932
ZINC15941858	-47.1396	-30.9783
ZINC19883233	-39.8073	-30.9742
ZINC17179824	-40.6901	-30.9586
ZINC00639829	-45.9871	-30.9514
ZINC04728675	-36.6675	-30.9487
ZINC00631486	-41.3172	-30.9414
ZINC04503591	-37.1067	-30.9403
ZINC08715657	-41.2586	-30.9309
ZINC08715657	-40.1211	-30.9309
ZINC08715657	-39.3293	-30.9309
ZINC08715657	-39.1785	-30.9309
ZINC08715657	-38.6272	-30.9309
ZINC13483378	-38.4214	-30.9200
ZINC00384728	-37.9872	-30.9187
ZINC19884754	-39.5948	-30.8996
ZINC08430099	-47.2582	-30.8986
ZINC06265568	-39.5684	-30.8898
ZINC05408995	-36.6190	-30.8732
ZINC06719106	-37.7403	-30.8722
ZINC06719106	-36.9376	-30.8722
ZINC19314908	-40.7192	-30.8623
ZINC20265041	-42.3465	-30.8558
ZINC04629166	-38.4207	-30.8547
ZINC19904621	-40.0860	-30.8490
ZINC19904282	-37.1733	-30.8486
ZINC12649519	-38.3557	-30.8414
ZINC00202075	-36.4014	-30.8316
ZINC08439628	-40.7570	-30.8307
ZINC00669856	-42.7608	-30.8194
ZINC15974445	-36.5887	-30.8080
ZINC17197211	-40.7306	-30.7911
ZINC17197211	-39.8926	-30.7911
ZINC17197211	-39.6341	-30.7911
ZINC17197211	-39.6271	-30.7911
ZINC09275404	-38.5529	-30.7717
ZINC04937436	-39.5998	-30.7599
ZINC00632590	-42.4725	-30.7321

ZINC01131694	-37.8607	-30.7142
ZINC19872240	-38.5500	-30.7115
ZINC07053414	-37.2260	-30.7093
ZINC05446022	-37.6013	-30.7037
ZINC12403697	-38.2658	-30.6796
ZINC19788281	-44.9451	-30.6681
ZINC20081620	-43.7452	-30.6645
ZINC17162313	-40.2056	-30.6513
ZINC06028959	-45.9247	-30.6496
ZINC15894589	-43.6098	-30.6431
ZINC05130913	-42.5050	-30.6386
ZINC32601557	-42.0640	-30.6385
ZINC04147517	-40.5395	-30.6289
ZINC06023577	-40.5356	-30.6231
ZINC19535850	-38.5405	-30.6100
ZINC02752261	-41.4146	-30.5968
ZINC18227536	-37.9229	-30.5879
ZINC00863568	-37.1751	-30.5870
ZINC00645305	-37.3080	-30.5727
ZINC17180542	-42.4083	-30.5670
ZINC17180542	-42.2651	-30.5670
ZINC17180542	-42.2090	-30.5670
ZINC17180542	-41.5386	-30.5670
ZINC17180542	-41.2343	-30.5670
ZINC00969983	-39.6519	-30.5655
ZINC02064475	-39.4771	-30.5590
ZINC05408960	-41.3531	-30.5573
ZINC05408960	-40.0994	-30.5573
ZINC05408960	-39.6928	-30.5573
ZINC05408960	-39.1732	-30.5573
ZINC03893928	-40.7488	-30.5566
ZINC36030364	-37.2377	-30.5520
ZINC00672341	-42.1913	-30.5439
ZINC00672341	-41.5875	-30.5439
ZINC04457668	-39.4396	-30.5231
ZINC32601906	-38.8131	-30.5210
ZINC09289642	-39.9357	-30.5152
ZINC08492403	-36.8575	-30.5098
ZINC17146766	-37.9208	-30.5091
ZINC00674033	-37.4001	-30.4928
ZINC19883114	-38.8788	-30.4870
ZINC32573576	-38.8704	-30.4864
ZINC09008407	-44.4325	-30.4753
ZINC09008407	-44.1659	-30.4753
ZINC09008407	-43.3564	-30.4753
ZINC09008407	-42.2077	-30.4753
ZINC31854412	-37.6868	-30.4735
ZINC04114218	-37.1460	-30.4692
ZINC19898482	-37.2931	-30.4606
ZINC00439212	-38.7382	-30.4546
ZINC19872068	-42.3173	-30.4531
ZINC13689608	-47.1407	-30.4383
ZINC02991868	-41.5391	-30.4290
ZINC00387191	-36.4799	-30.4289

ZINC00665813	-37.5403	-30.3982
ZINC02134712	-45.2948	-30.3865
ZINC13111557	-41.2883	-30.3814
ZINC00484917	-37.0700	-30.3677
ZINC13116652	-43.7972	-30.3494
ZINC19872070	-43.6313	-30.3298
ZINC00626669	-37.4950	-30.3073
ZINC08838200	-39.0578	-30.3002
ZINC09242679	-41.3429	-30.2728
ZINC00892707	-36.3762	-30.2605
ZINC05845809	-40.3809	-30.2404
ZINC02182244	-37.0136	-30.2247
ZINC03909210	-40.6744	-30.2143
ZINC27823876	-38.0931	-30.1871
ZINC04473451	-37.5687	-30.1781
ZINC19898494	-37.6129	-30.1663
ZINC32506699	-40.3568	-30.1615
ZINC06493361	-37.0225	-30.1517
ZINC00318693	-36.8908	-30.1162
ZINC01110580	-37.7008	-30.1138
ZINC20171123	-41.7502	-30.1027
ZINC08463040	-39.5774	-30.0901
ZINC04631506	-38.2702	-30.0766
ZINC13120609	-39.4578	-30.0757
ZINC19883110	-39.7728	-30.0628
ZINC00702148	-39.0776	-30.0376
ZINC30947894	-41.6593	-30.0275
ZINC13116638	-38.5047	-30.0177
ZINC00703545	-36.6001	-30.0066
ZINC18143049	-38.8462	-29.9834
ZINC12507974	-37.8211	-29.9813
ZINC35047568	-41.6365	-29.9806
ZINC00381100	-37.8902	-29.9800
ZINC20227683	-41.5945	-29.9784
ZINC00623233	-36.3036	-29.9777
ZINC08450363	-39.6079	-29.9763
ZINC10294998	-36.6158	-29.9672
ZINC00651506	-37.2337	-29.9633
ZINC16791104	-38.3924	-29.9628
ZINC15016110	-37.2860	-29.9367
ZINC19872491	-38.8056	-29.9325
ZINC00098117	-39.2192	-29.9232
ZINC08437187	-39.8725	-29.9163
ZINC09012982	-39.4553	-29.9060
ZINC19923145	-40.0006	-29.9049
ZINC19814820	-41.3195	-29.9047
ZINC19996903	-38.9282	-29.9041
ZINC02191249	-49.1194	-29.8924
ZINC05764286	-40.4488	-29.8880
ZINC02896407	-36.8205	-29.8847
ZINC09012113	-38.4282	-29.8724
ZINC00103247	-38.4767	-29.8568
ZINC57241858	-38.2139	-29.8555
ZINC13161471	-39.8186	-29.8374

ZINC20229284	-42.6122	-29.8366
ZINC31903964	-42.3591	-29.8083
ZINC55462816	-39.8186	-29.8056
ZINC55462816	-39.3990	-29.8056
ZINC01075989	-37.3234	-29.7793
ZINC15015668	-40.3262	-29.7771
ZINC15015668	-38.1682	-29.7771
ZINC36646150	-38.9128	-29.7167
ZINC08845352	-39.7529	-29.7133
ZINC15016100	-36.6278	-29.6934
ZINC15016100	-36.4791	-29.6934
ZINC04631657	-39.1749	-29.6898
ZINC00977601	-45.6811	-29.6724
ZINC00987575	-36.4810	-29.6679
ZINC05014438	-36.8147	-29.6567
ZINC19770447	-36.3896	-29.6474
ZINC19923870	-48.2635	-29.6287
ZINC00481390	-37.9316	-29.6099
ZINC05898070	-37.2210	-29.6064
ZINC04825614	-37.4601	-29.5877
ZINC04825614	-36.8256	-29.5877
ZINC19901262	-38.0518	-29.5863
ZINC04285087	-43.4055	-29.5568
ZINC00968836	-38.3059	-29.5541
ZINC19972596	-42.6425	-29.5465
ZINC05898427	-37.4154	-29.5268
ZINC00719487	-39.9083	-29.5252
ZINC19943473	-37.0073	-29.5158
ZINC06719107	-36.9242	-29.5075
ZINC36636407	-37.3772	-29.4931
ZINC19901236	-36.6146	-29.4922
ZINC05807405	-41.5479	-29.4634
ZINC03656738	-46.2702	-29.4607
ZINC13147512	-37.2854	-29.4567
ZINC00671077	-42.6529	-29.4308
ZINC28051460	-39.6054	-29.4053
ZINC04386198	-40.2897	-29.3934
ZINC68300478	-36.9918	-29.3891
ZINC13712106	-41.3799	-29.3851
ZINC13712106	-41.2832	-29.3851
ZINC22782523	-37.5463	-29.3836
ZINC04041240	-36.4883	-29.3803
ZINC04452963	-38.3024	-29.3797
ZINC18124909	-45.0499	-29.3681
ZINC18124909	-39.9372	-29.3681
ZINC04453678	-38.4801	-29.3653
ZINC04453678	-36.5925	-29.3653
ZINC17138860	-40.4139	-29.3601
ZINC01236577	-38.5877	-29.3544
ZINC00481393	-37.1276	-29.3534
ZINC00652071	-37.4819	-29.3463
ZINC06492924	-37.1583	-29.3424
ZINC19822817	-40.4873	-29.3329
ZINC06783582	-38.5891	-29.3280

ZINC00795322	-39.8737	-29.2992
ZINC02093169	-38.8143	-29.2728
ZINC00164073	-36.6230	-29.2543
ZINC20028363	-40.6212	-29.2536
ZINC00683214	-40.8702	-29.2384
ZINC01662549	-36.3493	-29.2210
ZINC00646719	-43.5395	-29.2172
ZINC36053442	-37.9910	-29.2152
ZINC09111321	-39.4068	-29.2089
ZINC09111321	-37.0910	-29.2089
ZINC00481365	-38.6846	-29.2079
ZINC04567166	-43.6011	-29.1999
ZINC04567166	-42.3531	-29.1999
ZINC19832371	-43.4813	-29.1809
ZINC17146206	-38.5637	-29.1753
ZINC00234941	-39.6794	-29.1631
ZINC17918674	-38.8767	-29.1450
ZINC16791107	-39.1044	-29.1320
ZINC13108558	-43.5651	-29.1282
ZINC71415908	-38.0429	-29.1129
ZINC19898479	-38.6899	-29.1099
ZINC17185152	-36.8290	-29.0916
ZINC17185152	-36.7301	-29.0916
ZINC17185152	-36.5798	-29.0916
ZINC06493359	-36.6963	-29.0911
ZINC00704080	-40.8966	-29.0898
ZINC17138863	-39.0946	-29.0891
ZINC35647443	-37.5418	-29.0859
ZINC38534550	-38.6425	-29.0837
ZINC00816422	-44.5591	-29.0726
ZINC12372251	-37.7560	-29.0391
ZINC15952207	-41.9599	-29.0361
ZINC02055125	-38.5535	-29.0276
ZINC00811147	-39.1241	-29.0161
ZINC08453276	-43.5069	-29.0113
ZINC06138408	-37.6549	-29.0104
ZINC01025981	-36.8557	-29.0102
ZINC20010630	-37.8762	-28.9953
ZINC00646431	-36.8849	-28.9304
ZINC00626274	-40.7148	-28.9201
ZINC08442004	-37.6894	-28.8990
ZINC20529923	-42.0665	-28.8983
ZINC02069372	-36.4065	-28.8917
ZINC01236591	-38.9395	-28.8885
ZINC00382813	-39.0545	-28.8720
ZINC00382813	-36.4901	-28.8720
ZINC19938298	-36.9874	-28.8608
ZINC00713463	-36.3863	-28.8437
ZINC55539476	-40.4684	-28.8414
ZINC16677272	-38.5208	-28.8384
ZINC17146204	-39.2209	-28.8206
ZINC05898042	-36.4273	-28.8015
ZINC02061187	-37.0862	-28.7570
ZINC19597204	-41.3135	-28.7333

ZINC00631124	-38.8774	-28.7099
ZINC09095741	-40.9946	-28.7097
ZINC00726125	-38.1070	-28.7011
ZINC04631263	-40.6505	-28.6881
ZINC00374711	-36.5630	-28.6854
ZINC16648936	-38.5238	-28.6649
ZINC18032184	-41.8903	-28.6371
ZINC18032184	-40.5707	-28.6371
ZINC08437418	-37.9862	-28.6132
ZINC08437418	-37.0194	-28.6132
ZINC08437418	-36.2634	-28.6132
ZINC02077393	-50.8367	-28.5888
ZINC07344261	-36.6787	-28.5428
ZINC08997394	-39.6344	-28.5238
ZINC08997394	-36.9217	-28.5238
ZINC13119718	-37.0530	-28.5229
ZINC00850352	-39.3161	-28.5181
ZINC00318695	-40.8690	-28.5148
ZINC00239206	-38.4982	-28.4943
ZINC09289639	-37.5482	-28.4878
ZINC00651440	-37.4886	-28.4552
ZINC02064467	-38.5606	-28.4235
ZINC00142326	-40.3865	-28.4234
ZINC03656696	-43.7516	-28.4212
ZINC04023002	-40.7709	-28.4196
ZINC08441904	-37.2733	-28.4049
ZINC18244887	-37.1937	-28.3737
ZINC15894590	-41.3841	-28.3535
ZINC08973075	-36.9447	-28.3532
ZINC06013557	-41.8327	-28.3120
ZINC02055171	-36.9782	-28.2942
ZINC19855045	-37.9554	-28.2736
ZINC19897495	-46.4562	-28.2723
ZINC08836237	-40.6294	-28.2467
ZINC08836237	-40.3163	-28.2467
ZINC19898492	-37.2294	-28.2286
ZINC19799422	-38.4526	-28.2133
ZINC38378350	-40.2930	-28.2114
ZINC11990654	-37.4892	-28.2023
ZINC13596824	-42.6324	-28.1867
ZINC13596824	-41.2626	-28.1867
ZINC00645523	-36.4969	-28.1794
ZINC02063415	-37.7080	-28.1503
ZINC00725316	-37.9585	-28.1458
ZINC00651434	-36.3651	-28.1223
ZINC20166934	-43.4342	-28.1195
ZINC19938395	-42.2920	-28.1146
ZINC00631418	-38.6161	-28.0967
ZINC05826173	-37.3178	-28.0870
ZINC17124459	-38.3870	-28.0633
ZINC00816420	-47.1335	-28.0315
ZINC00644717	-37.3544	-28.0300
ZINC17079807	-42.3406	-28.0055
ZINC17079807	-38.0841	-28.0055

ZINC19872817	-41.0617	-28.0046
ZINC19535853	-37.4234	-27.9854
ZINC00445165	-37.0930	-27.9816
ZINC08442186	-38.6842	-27.9754
ZINC08442186	-37.7673	-27.9754
ZINC08442186	-36.9282	-27.9754
ZINC17143760	-36.7720	-27.9517
ZINC20588755	-39.6293	-27.9477
ZINC17058256	-36.8887	-27.9261
ZINC06553180	-40.3134	-27.9247
ZINC19796892	-36.5722	-27.9227
ZINC08462839	-36.6397	-27.9076
ZINC08462839	-36.4046	-27.9076
ZINC00665833	-45.6194	-27.8871
ZINC04523833	-43.5349	-27.8755
ZINC19881737	-41.6341	-27.8560
ZINC00634748	-37.8538	-27.8466
ZINC06557164	-36.9211	-27.8150
ZINC04001813	-41.1037	-27.7490
ZINC01075993	-37.5073	-27.7443
ZINC00645302	-38.7427	-27.7307
ZINC11565873	-39.6529	-27.7058
ZINC09360347	-37.8904	-27.6793
ZINC01236579	-37.6288	-27.6754
ZINC06141464	-45.0830	-27.6654
ZINC04649756	-37.8023	-27.6521
ZINC16791109	-38.3360	-27.6437
ZINC00815450	-40.2474	-27.6428
ZINC20192845	-37.5240	-27.6292
ZINC02184810	-42.8446	-27.6072
ZINC04567346	-42.4674	-27.5827
ZINC18322815	-36.7957	-27.5764
ZINC02064426	-39.4060	-27.5656
ZINC00702142	-38.5604	-27.5394
ZINC19926583	-38.1913	-27.5121
ZINC19883235	-38.9148	-27.5078
ZINC00479485	-36.8470	-27.5073
ZINC00820923	-40.5768	-27.4966
ZINC00423503	-36.8880	-27.4918
ZINC16791105	-38.7845	-27.4799
ZINC20028364	-37.7353	-27.4676
ZINC02927255	-39.8365	-27.4330
ZINC19909426	-40.8785	-27.4221
ZINC19872821	-40.3237	-27.4117
ZINC08399955	-38.2135	-27.3909
ZINC06694171	-39.6152	-27.3518
ZINC06694171	-37.3060	-27.3518
ZINC37867318	-37.5910	-27.3422
ZINC49580461	-36.6467	-27.2960
ZINC18208426	-37.6829	-27.2725
ZINC19926700	-43.3823	-27.2121
ZINC18122346	-36.8246	-27.1287
ZINC06885450	-36.7319	-27.1271
ZINC00944090	-37.8411	-27.0834

ZINC12338665	-43.5405	-27.0814
ZINC12338665	-40.4074	-27.0814
ZINC02180695	-39.3018	-27.0709
ZINC04453680	-38.8252	-27.0611
ZINC04453680	-38.5799	-27.0611
ZINC00726747	-36.5514	-27.0536
ZINC01236589	-38.9995	-27.0133
ZINC00353640	-36.6093	-26.9855
ZINC05285421	-39.1212	-26.9798
ZINC15961957	-40.9730	-26.9645
ZINC15961957	-40.7415	-26.9645
ZINC06702951	-37.6732	-26.9170
ZINC20212997	-36.8481	-26.9126
ZINC00834423	-37.1756	-26.8468
ZINC17723208	-41.7255	-26.8459
ZINC15777253	-36.5863	-26.8298
ZINC15777253	-36.5129	-26.8298
ZINC20101629	-38.1196	-26.8182
ZINC01225906	-39.0792	-26.6885
ZINC85423403	-37.7591	-26.6825
ZINC19923142	-41.2728	-26.6763
ZINC08690303	-36.2605	-26.6422
ZINC08714634	-36.5214	-26.6350
ZINC19923872	-47.7351	-26.5987
ZINC08714716	-41.6534	-26.5941
ZINC20027324	-38.8939	-26.5684
ZINC16651349	-41.9125	-26.4833
ZINC01802997	-38.9166	-26.4559
ZINC06173913	-36.4248	-26.4198
ZINC19872154	-37.1654	-26.3589
ZINC13043426	-39.0288	-26.3004
ZINC13688084	-36.7339	-26.2938
ZINC05488752	-36.9775	-26.2769
ZINC16995704	-36.4523	-26.2737
ZINC08442190	-37.3568	-26.2434
ZINC08442190	-37.2666	-26.2434
ZINC00645323	-46.4328	-26.2042
ZINC20231535	-41.7210	-26.1674
ZINC06813211	-37.8610	-26.1632
ZINC08440274	-40.8390	-26.1618
ZINC08714769	-37.4388	-26.1131
ZINC09289634	-38.8662	-26.0795
ZINC08715627	-42.3343	-26.0364
ZINC19368225	-38.8473	-26.0118
ZINC19368225	-37.0413	-26.0118
ZINC00484687	-36.3468	-26.0096
ZINC13108806	-36.3489	-25.9738
ZINC02501579	-36.6920	-25.8852
ZINC02690734	-45.9381	-25.8525
ZINC19938363	-39.2527	-25.8398
ZINC00998652	-42.9077	-25.8328
ZINC00998652	-37.8509	-25.8328
ZINC04385458	-38.1155	-25.8256
ZINC27823983	-36.9283	-25.7942

ZINC19901554	-39.5141	-25.7826
ZINC19901554	-39.1100	-25.7826
ZINC00725847	-39.1516	-25.7687
ZINC01305604	-37.0408	-25.6759
ZINC18240564	-38.0463	-25.6124
ZINC18240564	-37.8798	-25.6124
ZINC18240564	-37.0657	-25.6124
ZINC00386937	-37.1927	-25.5501
ZINC00629294	-39.3606	-25.5203
ZINC02069232	-36.4890	-25.4826
ZINC00142323	-43.0410	-25.4793
ZINC33710766	-36.7641	-25.4639
ZINC32573591	-38.3656	-25.4503
ZINC04645989	-36.7143	-25.3898
ZINC00571015	-38.3515	-25.3731
ZINC19996899	-37.3744	-25.3720
ZINC02760403	-36.3370	-25.3551
ZINC01009030	-36.7038	-25.3308
ZINC19314911	-37.6410	-25.3067
ZINC20215552	-42.0285	-25.2353
ZINC32601617	-38.7217	-25.1809
ZINC08438531	-39.2427	-25.0858
ZINC35592602	-37.6599	-25.0826
ZINC08438526	-39.2040	-25.0045
ZINC19822758	-38.2456	-24.9911
ZINC00665578	-44.5394	-24.9686
ZINC11566020	-37.0890	-24.9595
ZINC04174793	-37.0334	-24.9431
ZINC16958532	-38.7660	-24.9138
ZINC04615061	-38.1581	-24.9001
ZINC19815496	-36.5681	-24.9000
ZINC04911515	-40.0028	-24.8216
ZINC32601651	-38.6731	-24.6903
ZINC10312638	-36.9816	-24.6612
ZINC00366582	-37.2627	-24.6548
ZINC01054922	-37.1829	-24.5841
ZINC00076701	-39.2016	-24.5642
ZINC00987844	-40.9250	-24.5221
ZINC00987844	-40.3925	-24.5221
ZINC19815598	-37.7448	-24.5142
ZINC19815598	-36.5216	-24.5142
ZINC19904207	-39.3607	-24.5084
ZINC06519942	-36.5450	-24.5039
ZINC32601673	-41.8516	-24.4231
ZINC08714688	-39.3065	-24.3014
ZINC16136366	-39.9827	-24.2596
ZINC16136366	-39.8993	-24.2596
ZINC16136366	-39.8853	-24.2596
ZINC16136366	-39.4684	-24.2596
ZINC16136366	-39.4336	-24.2596
ZINC16136366	-36.8089	-24.2596
ZINC00638542	-42.7125	-24.2024
ZINC04647028	-38.1864	-24.1549
ZINC17117891	-41.7964	-24.0700

ZINC17117891	-37.9381	-24.0700
ZINC19815074	-40.7732	-23.9172
ZINC32601645	-37.9129	-23.9105
ZINC33331372	-36.2584	-23.8852
ZINC38534552	-37.2435	-23.8373
ZINC18271611	-40.8146	-23.7330
ZINC18271611	-40.6448	-23.7330
ZINC35592611	-38.8409	-23.5563
ZINC00384649	-38.9949	-23.4569
ZINC35592590	-37.1885	-23.3552
ZINC39929739	-38.3940	-23.2769
ZINC15986386	-38.8358	-23.2387
ZINC05234912	-38.3958	-23.2001
ZINC05446024	-37.1396	-23.0951
ZINC05446024	-37.0267	-23.0951
ZINC00366584	-36.3177	-22.9906
ZINC08444443	-40.5999	-22.8113
ZINC13555237	-42.1872	-22.7382
ZINC08397746	-48.3020	-22.4317
ZINC13598060	-40.2469	-22.3721
ZINC13598060	-40.0401	-22.3721
ZINC13598060	-39.6746	-22.3721
ZINC13598060	-38.9085	-22.3721
ZINC02055805	-40.4552	-22.3689
ZINC05684513	-39.4824	-22.3542
ZINC08714765	-36.7828	-22.2837
ZINC00339412	-36.9696	-22.1083
ZINC04660816	-40.3420	-21.8145
ZINC48044544	-39.6440	-21.7341
ZINC03187699	-36.7555	-21.4918
ZINC06814029	-36.6770	-21.4399
ZINC00671448	-39.7549	-20.7568
ZINC02077118	-48.0993	-20.5787
ZINC04631512	-36.5047	-20.5504
ZINC02184800	-41.8265	-20.5267
ZINC04112863	-37.9771	-19.9109
ZINC32601647	-38.8278	-19.9081
ZINC00628882	-39.9329	-19.8751
ZINC04285064	-40.5314	-19.2620
ZINC00572690	-36.3308	-18.9843
ZINC32601620	-37.1400	-18.8057
ZINC04285065	-39.2689	-18.5326
ZINC19797259	-36.4631	-18.4310
ZINC00575916	-36.6313	-18.1715
ZINC02064499	-44.3429	-17.9602
ZINC21363301	-40.7662	-17.9357
ZINC36046680	-39.6903	-17.4990
ZINC36046680	-38.0854	-17.4990
ZINC04660901	-36.2894	-15.9735
ZINC00575915	-38.6082	-15.8045
ZINC15885207	-41.1031	-15.5764
ZINC15885207	-40.3434	-15.5764
ZINC04681147	-44.6507	-14.9934
ZINC32601589	-36.3550	-14.3978

ZINC32601555	-39.3035	-13.8114
ZINC00258723	-37.4902	-13.0795
ZINC02064500	-42.5419	-12.9571
ZINC05728931	-39.9519	-11.9321
ZINC19897353	-36.4009	-4.4747
ZINC19911511	-38.8490	2.8545
ZINC19900847	-45.9927	36.6012
ZINC19900847	-44.2614	36.6012
