# Supplementary material for: In silico identification of potential inhibitors targeting Streptococcus mutans sortase A
Source: Int J Oral Sci. 2017 Mar 30;9(1):53–62. doi: 10.1038/ijos.2016.58 (PMC5379162; doi:10.1038/ijos.2016.58)
Supplement: Supplementary Table S2 [file ijos201658x3.pdf]

Table S2 Top 60% ranked compounds in TONGTIAN library (the yellow fill color of scores are top 20 of each score).

Compound	Gride score	Hawkins GB/SA score	Flavonoids	Possible reasons
ZINC95098840	-56. 1525	-59. 2159		
ZINC83439137	-51. 4643	-56. 7569		
ZINC43450324	-50. 6498	-55. 4551		
ZINC21297660	-48. 9315	-54. 2875		
ZINC98230413	-55. 4630	-53. 5970		
ZINC70688139	-56. 8413	-53. 5298		
ZINC44460325	-51. 2913	-52. 7933		
ZINC15267534	-48. 2846	-52. 6984		
ZINC08234223	-38. 6124	-52. 0256		
ZINC06857614	-38. 1251	-51. 6440		
ZINC83439135	-46. 5850	-51. 6013		
ZINC13377892	-50. 7759	-51. 4761		
ZINC43450326	-49. 7887	-51. 3351		
ZINC08234227	-37. 3250	-51. 0728		
ZINC08681509	-52. 7755	-50. 9599	Naringin	low binding free energy
ZINC96023873	-47. 8948	-50. 4994		
ZINC43919185	-45. 2729	-50. 2989		
ZINC08234225	-38. 8861	-50. 0969		
ZINC71789779	-48. 4392	-49. 5143		
ZINC98230123	-48. 6497	-49. 2124		
ZINC71617415	-43. 9278	-49. 1448		
ZINC95098838	-57. 6850	-49. 0580		
ZINC31261437	-46. 0455	-49. 0213		
ZINC02522581	-45. 4144	-48. 9410		
ZINC02522581	-45. 4144	-48. 9410		
ZINC00597372	-44. 9673	-48. 8044		
ZINC59728187	-47. 3542	-48. 7758	Calycosin-7-O-β-D-glucosid	low binding free energy
ZINC98246400	-48. 6199	-48. 6090		
ZINC08234300	-49. 8883	-48. 6064		
ZINC08214880	-36. 4876	-48. 5214		
ZINC26011099	-48. 1538	-48. 4989		
ZINC98230122	-49. 4002	-48. 4234		
ZINC71789778	-47. 3655	-48. 3909		

ZINC95542869	-51.7338	-48.3399
ZINC31156634	-50.5459	-48.3243
ZINC17255287	-45.6866	-48.2918
ZINC72099499	-47.6289	-48.2077
ZINC13377891	-48.7655	-47.9547
ZINC15113472	-45.0487	-47.8206
ZINC04098749	-47.0262	-47.7833
ZINC38513295	-48.0254	-47.3923
ZINC00899824	-45.9672	-47.2217
ZINC33954928	-42.0290	-47.2085
ZINC44460324	-49.0339	-47.0995
ZINC08382286	-53.0881	-47.0469
ZINC59211730	-48.2653	-46.9752
ZINC31456743	-40.2808	-46.9255
ZINC08214776	-47.4981	-46.9236
ZINC08143568	-47.2865	-46.6834
ZINC38139712	-50.2176	-46.6559
ZINC04098737	-50.3142	-46.5813
ZINC85504689	-57.2324	-46.5604
ZINC08234302	-48.7424	-46.3014
ZINC01530575	-45.1453	-46.1651
ZINC04098672	-42.2113	-46.1479
ZINC59772579	-45.8531	-46.0583
ZINC05999071	-43.3892	-45.9857
ZINC72131064	-52.0499	-45.7244
ZINC04216676	-49.7075	-45.6869
ZINC08218968	-35.5928	-45.6737
ZINC95661168	-48.1475	-45.6554
ZINC85552319	-50.1364	-45.6330
ZINC17654711	-50.5420	-45.4829
ZINC77286302	-38.4750	-45.4481
ZINC87493308	-39.6124	-45.3644
ZINC04136964	-46.9206	-45.3380
ZINC59762889	-48.0705	-45.3110
ZINC44166798	-47.3907	-45.2815

ZINC13377893	-47.2887	-45.2602
ZINC13549420	-47.2026	-45.2407
ZINC38611992	-38.8242	-45.2396
ZINC13888876	-41.6133	-45.2348
ZINC44460322	-48.6066	-45.2274
ZINC38143679	-44.4664	-45.1388
ZINC00967733	-41.6178	-45.1303
ZINC04349764	-43.7083	-45.1057
ZINC13548856	-46.7052	-45.0759
ZINC71789472	-43.8440	-45.0645
ZINC29412110	-44.3085	-45.0274
ZINC08143604	-47.4096	-44.9796
ZINC08681473	-43.9544	-44.9268
ZINC05820844	-41.6594	-44.9151
ZINC04096846	-51.5990	-44.8847
ZINC67902505	-48.4974	-44.7556
ZINC44460323	-47.9052	-44.7122
ZINC39351839	-46.4100	-44.6805
ZINC08217495	-48.0059	-44.6670
ZINC13888877	-42.7073	-44.6647
ZINC03872686	-38.5255	-44.6581
ZINC98181787	-40.8183	-44.6456
ZINC33963983	-46.4122	-44.6422
ZINC59728190	-47.3179	-44.5926
ZINC22058780	-40.8994	-44.4802
ZINC71789776	-46.0807	-44.4418
ZINC08681472	-44.4242	-44.4403
ZINC77286295	-37.8915	-44.4245
ZINC08214885	-38.6890	-44.2787
ZINC70647110	-50.9704	-44.2717
ZINC26892394	-44.2031	-43.9499
ZINC04098838	-47.1217	-43.9438
ZINC04517652	-40.5714	-43.9416
ZINC38139713	-50.3923	-43.9191
ZINC85531309	-46.3390	-43.8658

ZINC70466449	-44.1992	-43.7923		
ZINC08214484	-49.0040	-43.7821		
ZINC08382360	-34.9959	-43.7426		
ZINC49898130	-49.2569	-43.7420		
ZINC39741105	-37.5680	-43.7214		
ZINC01531857	-38.9209	-43.6780		
ZINC00001087	-38.9881	-43.6614		
ZINC12501593	-46.0440	-43.6474		
ZINC87493307	-39.1155	-43.6127		
ZINC85473531	-41.6796	-43.4629		
ZINC31169743	-48.2470	-43.4473		
ZINC08217494	-48.5439	-43.4388		
ZINC04098355	-43.3230	-43.4286		
ZINC65748638	-44.8001	-43.4256		
ZINC36377990	-44.3652	-43.4151		
ZINC21992892	-46.7219	-43.3752		
ZINC04098521	-44.0458	-43.3548	Hinokiflavone	steric clash
ZINC98574923	-49.1111	-43.2587		
ZINC15121938	-37.8609	-43.2519		
ZINC01531664	-41.4478	-43.2066	Ginkgetin	steric clash
ZINC86860231	-35.0737	-43.1372		
ZINC08214752	-48.8297	-43.1116		
ZINC00777954	-37.7199	-43.1080		
ZINC08681508	-50.8363	-43.1035		
ZINC17044430	-44.1092	-43.0916		
ZINC13132546	-45.4266	-42.9909		
ZINC15657761	-45.6929	-42.8907		
ZINC36377991	-42.8610	-42.8206		
ZINC85473528	-44.2214	-42.7717		
ZINC15449158	-42.7337	-42.7662		
ZINC26892384	-42.7144	-42.7334		
ZINC85743655	-45.5910	-42.7293		
ZINC04099013	-38.4958	-42.6910		
ZINC08681833	-43.6693	-42.6909		
ZINC95098839	-53.4323	-42.6397		

ZINC15120527	-39.7324	-42.5708
ZINC08234358	-43.9221	-42.5483
ZINC05119406	-46.0256	-42.5027
ZINC04404499	-46.7798	-42.3934
ZINC06858611	-47.4157	-42.3303
ZINC13514961	-42.2180	-42.3067
ZINC00900152	-37.0797	-42.2788
ZINC38140514	-35.7880	-42.2533
ZINC04349762	-43.5152	-42.2315
ZINC38139417	-35.9376	-42.2190
ZINC95098860	-49.1324	-42.2155
ZINC04098728	-48.5444	-42.1998
ZINC76945764	-34.4166	-42.1612
ZINC71789471	-45.0261	-42.1547
ZINC12888720	-45.3535	-42.0756
ZINC12153441	-43.6449	-42.0617
ZINC70647111	-41.8250	-42.0184
ZINC04404500	-46.7257	-42.0161
ZINC04027246	-40.8571	-42.0130
ZINC09147119	-43.1416	-42.0039
ZINC71789474	-43.0381	-41.9639
ZINC08214722	-36.5641	-41.9401
ZINC71789777	-45.4095	-41.9027
ZINC05119408	-44.0759	-41.8466
ZINC59774193	-49.6343	-41.8169
ZINC00598071	-38.9095	-41.8101
ZINC03775646	-39.1501	-41.8080
ZINC95098843	-34.9522	-41.8006
ZINC08214878	-35.7341	-41.8005
ZINC05004611	-44.8045	-41.7532
ZINC03874885	-37.8867	-41.7360
ZINC85743647	-43.0852	-41.7165
ZINC04098354	-39.5197	-41.7111
ZINC71789802	-44.0535	-41.6767
ZINC05140293	-36.7804	-41.6404

ZINC12374719	-45.9138	-41.6086		
ZINC12495605	-44.6580	-41.6080		
ZINC05119394	-47.5177	-41.5809		
ZINC59817095	-49.1104	-41.5771		
ZINC95098773	-54.7345	-41.5671		
ZINC77286303	-36.9073	-41.5575		
ZINC04349372	-44.5662	-41.5555	Isoquercitrin	low binding free energy
ZINC04349442	-43.0120	-41.5141		
ZINC86007381	-46.3676	-41.5082		
ZINC95098844	-34.9173	-41.4960		
ZINC12374720	-45.5496	-41.4811		
ZINC44004957	-46.8482	-41.4547		
ZINC04098747	-41.5651	-41.4523		
ZINC14984516	-42.7062	-41.4508		
ZINC04404431	-34.5331	-41.4341		
ZINC08214883	-35.0528	-41.4181		
ZINC13311346	-35.7241	-41.4102		
ZINC03874884	-38.1282	-41.3629		
ZINC12374721	-42.7477	-41.3279		
ZINC86860240	-36.4078	-41.2778		
ZINC13301703	-36.0506	-41.2551		
ZINC03861150	-32.7030	-41.2514		
ZINC01691515	-33.4882	-41.1964		
ZINC38940718	-42.7546	-41.1354		
ZINC30731533	-46.0237	-41.1213		
ZINC05157983	-38.4093	-41.0776		
ZINC72206342	-47.7772	-41.0752		
ZINC04098657	-43.1268	-41.0692		
ZINC15657801	-41.0418	-41.0077		
ZINC77286300	-33.0269	-40.9866		
ZINC01081322	-41.2848	-40.9385		
ZINC95098765	-39.5513	-40.9301		
ZINC14690026	-49.5765	-40.9173		
ZINC95098841	-34.3375	-40.8642		
ZINC04097723	-36.2857	-40.8048		

ZINC86007384	−42.8529	−40.7693		
ZINC31169747	−44.3157	−40.7673		
ZINC05115722	−41.5487	−40.7353		
ZINC14684607	−41.5769	−40.7156		
ZINC14695963	−36.5783	−40.7043		
ZINC04098610	−43.6062	−40.6488		
ZINC04404502	−42.0728	−40.6452		
ZINC01587485	−35.9082	−40.6447		
ZINC04692015	−42.3963	−40.6075		
ZINC04349785	−44.4679	−40.5854		
ZINC31494867	−36.6774	−40.5785		
ZINC98230407	−34.6756	−40.5555		
ZINC02548962	−36.2591	−40.5450		
ZINC04098604	−48.7622	−40.5429		
ZINC86860234	−34.2869	−40.5029		
ZINC85599244	−43.0302	−40.4844		
ZINC59762891	−47.6813	−40.4529		
ZINC08382361	−34.4367	−40.4506		
ZINC15270760	−38.3656	−40.4279		
ZINC59775376	−41.8890	−40.4247		
ZINC04081455	−32.1864	−40.3584		
ZINC35644653	−43.0797	−40.3275		
ZINC04348671	−36.0505	−40.2606	Bavachinin	steric clash
ZINC14444766	−42.2635	−40.1905		
ZINC06143120	−44.7770	−40.1485		
ZINC04081606	−36.3588	−40.1422		
ZINC05368587	−36.2665	−40.1152		
ZINC04349780	−43.8931	−40.0353		
ZINC04349394	−43.5399	−40.0327		
ZINC08382362	−32.0221	−39.9846		
ZINC13424731	−41.3943	−39.9822		
ZINC01651126	−38.9468	−39.9536		
ZINC04217426	−49.9770	−39.9418		
ZINC77292042	−42.5630	−39.8829		
ZINC33831794	−43.1181	−39.8677	Calycosin-7-O-β-D-glucosid	low binding free energy

ZINC13536861	−34.3406	−39.8619		
ZINC70466451	−43.6942	−39.8494		
ZINC13515662	−45.9105	−39.8469	Quercetin 3-glucuronide	HIA-
ZINC15657768	−41.5250	−39.8130		
ZINC12429154	−43.0525	−39.7642		
ZINC86860229	−34.4854	−39.7179		
ZINC03197535	−42.2607	−39.6961	Isoginkgetin	steric clash
ZINC13859683	−42.5422	−39.6957		
ZINC59728197	−44.5539	−39.6850		
ZINC03977894	−41.0818	−39.6733		
ZINC00899902	−35.7599	−39.6694		
ZINC06037228	−33.9763	−39.6609		
ZINC31156210	−35.2604	−39.6558		
ZINC03978650	−31.8421	−39.6383		
ZINC95098842	−33.4241	−39.6376		
ZINC04098602	−47.7042	−39.6375		
ZINC31430475	−38.7979	−39.6130		
ZINC04654817	−47.4759	−39.6103	Baimaside	steric clash
ZINC02558155	−32.4407	−39.5877		
ZINC85589560	−45.1163	−39.5834		
ZINC04214317	−44.1712	−39.5722		
ZINC04349262	−46.9065	−39.5620		
ZINC85673505	−35.2743	−39.5538		
ZINC05641945	−37.8097	−39.4803		
ZINC05665357	−34.9678	−39.4172		
ZINC04104917	−40.4393	−39.4144		
ZINC22055345	−34.2388	−39.3979		
ZINC31430473	−38.7123	−39.3813		
ZINC04098352	−41.6479	−39.3721		
ZINC85599246	−42.3748	−39.3173		
ZINC03874671	−43.9365	−39.3104		
ZINC33949418	−42.6635	−39.3009		
ZINC04098822	−36.6278	−39.2713		
ZINC13827710	−40.6968	−39.2691		
ZINC85475129	−42.3893	−39.2210		

ZINC66166674	-42.5446	-39.1941
ZINC12429156	-43.5724	-39.1767
ZINC03874952	-32.6959	-39.1506
ZINC03874952	-32.6959	-39.1506
ZINC12358620	-46.7300	-39.1150
ZINC59817101	-47.9602	-39.1084
ZINC04097976	-32.9163	-39.0515
ZINC30727894	-33.4122	-39.0373
ZINC95754342	-46.7784	-39.0260
ZINC13119573	-36.1073	-38.9640
ZINC08234294	-45.4110	-38.9340
ZINC05273634	-39.4370	-38.8976
ZINC85914710	-50.5399	-38.8888
ZINC08214406	-36.0523	-38.8845
ZINC85539971	-44.7621	-38.8318
ZINC59774197	-46.8831	-38.8189
ZINC05195808	-37.2116	-38.8170
ZINC33832439	-41.0554	-38.8107
ZINC08214884	-32.0396	-38.8085
ZINC06092613	-41.5002	-38.7849
ZINC06092613	-41.5002	-38.7849
ZINC05369368	-38.3841	-38.7781
ZINC27646589	-37.5169	-38.7715
ZINC12888715	-40.6461	-38.7647
ZINC05998954	-42.5100	-38.7454
ZINC05735995	-36.5807	-38.7215
ZINC70466442	-36.1397	-38.6561
ZINC98574912	-40.9218	-38.6250
ZINC03831448	-42.2449	-38.5854
ZINC62236199	-51.5894	-38.5645
ZINC09147120	-40.8966	-38.5557
ZINC05179146	-35.6774	-38.5263
ZINC60292553	-31.9933	-38.5184
ZINC08674071	-40.5096	-38.4652
ZINC12501595	-41.3429	-38.4538

ZINC86865007	-41.1311	-38.4517		
ZINC04416342	-41.5204	-38.4330		
ZINC00899797	-33.7394	-38.4320		
ZINC00477938	-35.8243	-38.4306		
ZINC15449112	-35.1545	-38.4287		
ZINC13424748	-40.6995	-38.3988		
ZINC98230408	-33.3009	-38.3704		
ZINC04098560	-40.1116	-38.3306		
ZINC00105309	-34.2891	-38.2744		
ZINC04097772	-52.0201	-38.2644		
ZINC18258326	-34.0313	-38.2421		
ZINC03793048	-40.2961	-38.2089		
ZINC03650002	-34.2221	-38.2085	Isobavachin	low binding free energy
ZINC14684605	-43.2576	-38.2062		
ZINC28524747	-34.5845	-38.2057		
ZINC39205825	-35.1298	-38.1992		
ZINC06143118	-43.2486	-38.1952		
ZINC05369367	-38.1010	-38.1757		
ZINC04349347	-40.5977	-38.1687		
ZINC04097789	-34.2272	-38.1534		
ZINC12153442	-42.2077	-38.0949		
ZINC04349776	-43.2073	-38.0873		
ZINC04098719	-33.8535	-38.0689		
ZINC95619383	-34.0003	-38.0214		
ZINC04098332	-39.9988	-38.0051		
ZINC13370792	-33.5497	-38.0022		
ZINC44699916	-40.7977	-37.9124		
ZINC33831557	-35.8038	-37.9105		
ZINC04349341	-42.2189	-37.8990		
ZINC76945759	-33.5581	-37.8772		
ZINC85743651	-43.0886	-37.8614		
ZINC34647724	-33.4970	-37.8546		
ZINC08952339	-40.1732	-37.8508		
ZINC70454883	-34.5484	-37.8505		
ZINC86860232	-33.2035	-37.8223		

ZINC95098766	-37.9307	-37.8179		
ZINC02570135	-34.6788	-37.8041	Neobavaisoflavone	low binding free energy
ZINC06569127	-40.6931	-37.7566		
ZINC08214882	-32.9457	-37.7537		
ZINC95630237	-53.8396	-37.7231		
ZINC03978503	-40.0347	-37.6749		
ZINC04102435	-39.2443	-37.6568		
ZINC59872849	-40.7095	-37.6299		
ZINC03871474	-43.1358	-37.5992		
ZINC13827716	-41.1927	-37.5468		
ZINC33831307	-42.6447	-37.4839		
ZINC31459237	-31.9655	-37.4488		
ZINC33832535	-40.9387	-37.3689		
ZINC04654810	-46.1834	-37.3451		
ZINC01644304	-32.9121	-37.3342		
ZINC04096845	-41.7012	-37.2989	Isoquercitrin	low binding free energy
ZINC04096845	-41.7012	-37.2989		
ZINC04349794	-42.1104	-37.2817		
ZINC01608669	-32.6059	-37.2776		
ZINC36227892	-42.0008	-37.2564		
ZINC03875408	-43.1812	-37.2530		
ZINC29786369	-43.9308	-37.2231		
ZINC20230445	-42.5320	-37.2230		
ZINC39204549	-33.9854	-37.2177		
ZINC05733294	-40.2628	-37.2071		
ZINC05733294	-40.2628	-37.2071		
ZINC13761324	-42.3506	-37.1665		
ZINC04692659	-36.1203	-37.1306		
ZINC04692659	-35.7790	-37.1306		
ZINC37866089	-33.4977	-37.1297		
ZINC03978792	-39.5725	-37.1152		
ZINC85473393	-35.7035	-37.1124		
ZINC04098745	-41.0152	-37.1058		
ZINC01725698	-34.8110	-37.0825		
ZINC01999512	-35.2068	-37.0767		

ZINC08214775	-48.8999	-37.0411		
ZINC12429155	-41.1421	-36.9614		
ZINC05733295	-37.9789	-36.9524		
ZINC05733295	-37.9789	-36.9524		
ZINC49600032	-33.7707	-36.9171		
ZINC86865005	-40.9711	-36.8737		
ZINC01530836	-32.6150	-36.8645		
ZINC13479082	-40.1549	-36.8323		
ZINC38515687	-35.7280	-36.8160		
ZINC03860715	-35.5969	-36.8016		
ZINC04097985	-37.5942	-36.8009		
ZINC04096342	-37.3923	-36.7899		
ZINC13424727	-41.7133	-36.7841	Isoquercitrin	low binding free energy
ZINC04098556	-41.4799	-36.7738		
ZINC59763264	-37.6284	-36.7569		
ZINC95098774	-56.1473	-36.7354		
ZINC04097836	-33.8349	-36.6975		
ZINC85599242	-39.5449	-36.6781		
ZINC15657732	-39.6504	-36.6714		
ZINC04692016	-40.6754	-36.6689		
ZINC03870414	-40.4668	-36.6612		
ZINC59733025	-35.8256	-36.6258		
ZINC08214476	-34.2244	-36.5968		
ZINC08234364	-33.9059	-36.5948		
ZINC19795974	-33.0598	-36.5860		
ZINC13481899	-33.0411	-36.5817	Methylophiopogonanone A	low binding free energy
ZINC38143877	-52.4915	-36.5769	Isochlorogenic acid A	steric clash, low binding free energy
ZINC03871891	-32.0131	-36.5611		
ZINC03873123	-33.8579	-36.5448		
ZINC05369366	-38.6408	-36.5395		
ZINC04349811	-37.6879	-36.5330		
ZINC15657718	-39.0925	-36.5135		
ZINC35328494	-41.7334	-36.4608		
ZINC25980356	-34.5066	-36.4599		
ZINC15449111	-31.6963	-36.4196		

ZINC02554900	-34.7967	-36.3705
ZINC04097913	-41.9697	-36.3638
ZINC71789807	-40.3251	-36.3194
ZINC95098876	-47.3855	-36.3161
ZINC14642853	-42.1532	-36.3105
ZINC04692658	-34.1493	-36.2881
ZINC04692658	-33.2693	-36.2881
ZINC33830337	-33.6330	-36.2390
ZINC16954710	-33.4109	-36.2311
ZINC44699288	-43.0900	-36.2141
ZINC85643466	-47.1000	-36.2076
ZINC04175638	-41.0084	-36.2022
ZINC01580260	-38.2055	-36.1955
ZINC14089767	-33.3089	-36.1228
ZINC36227889	-41.9105	-36.0830
ZINC04416344	-38.7938	-36.0811
ZINC45366404	-42.6700	-36.0701
ZINC59730688	-45.2368	-36.0635
ZINC03860441	-39.3951	-35.9927
ZINC30727228	-35.2130	-35.9700
ZINC30727228	-35.2130	-35.9700
ZINC34582505	-39.7768	-35.9689
ZINC13551787	-41.4725	-35.9398
ZINC05223934	-40.8050	-35.9329
ZINC14820584	-32.9201	-35.9206
ZINC05998802	-35.3470	-35.8981
ZINC98230409	-32.3323	-35.8970
ZINC00119434	-32.3452	-35.8478
ZINC04097706	-40.4301	-35.8467
ZINC29128982	-44.2027	-35.8449
ZINC04963990	-39.6215	-35.8202
ZINC04963990	-39.5102	-35.8202
ZINC95864967	-32.0984	-35.8093
ZINC14642850	-42.4522	-35.7830
ZINC12153073	-41.9392	-35.7434

ZINC01571580	−38.0318	−35.7390		
ZINC31165757	−31.9300	−35.7234		
ZINC12412601	−34.9308	−35.7061		
ZINC12412601	−34.5037	−35.7061		
ZINC05732375	−33.6676	−35.6791		
ZINC04164459	−34.0179	−35.6614		
ZINC15115057	−36.2174	−35.6516		
ZINC00338284	−34.4618	−35.6282		
ZINC33830335	−33.3648	−35.5683		
ZINC05430816	−33.9538	−35.5528	Isobavachin	low binding free energy
ZINC04096134	−33.6625	−35.5471		
ZINC39205823	−34.4913	−35.5342		
ZINC03979155	−32.6707	−35.5295		
ZINC70455423	−40.6430	−35.4828		
ZINC77312221	−33.2234	−35.4811		
ZINC14096305	−40.9412	−35.4571		
ZINC17027409	−33.7242	−35.4537		
ZINC00019968	−38.2104	−35.4419		
ZINC08829462	−38.4334	−35.4403		
ZINC33830716	−38.2674	−35.4210		
ZINC33830716	−38.2674	−35.4210		
ZINC04544252	−38.6449	−35.4075		
ZINC19795979	−32.1926	−35.3232		
ZINC03894277	−37.5266	−35.3195		
ZINC04098340	−38.1672	−35.3144		
ZINC95627886	−44.5760	−35.2924		
ZINC71789742	−34.8045	−35.2898		
ZINC21992887	−44.6260	−35.2718		
ZINC44699912	−39.6265	−35.2706		
ZINC05134835	−37.1382	−35.2450		
ZINC19893935	−40.1383	−35.2266		
ZINC08829464	−37.5640	−35.2262		
ZINC86860265	−37.9814	−35.2086		
ZINC03894278	−39.2179	−35.1695		
ZINC01591116	−37.1872	−35.1561		

ZINC04416338	−38.7383	−35.1225	
ZINC05854400	−36.3388	−35.1051	
ZINC03870415	−39.6853	−35.0544	
ZINC33861486	−46.3662	−35.0205	
ZINC32785894	−48.0255	−34.9940	
ZINC86028690	−36.0909	−34.9931	
ZINC05273633	−38.5815	−34.9535	
ZINC03984029	−32.5066	−34.9344	
ZINC71789563	−39.2027	−34.9139	
ZINC33832006	−41.5595	−34.8542	
ZINC38175116	−41.5021	−34.8243	
ZINC03882101	−40.1879	−34.8023	
ZINC05733292	−38.7357	−34.7901	
ZINC05733292	−38.7357	−34.7901	
ZINC04692014	−39.9334	−34.7725	
ZINC05733290	−39.8559	−34.6967	
ZINC05733290	−39.8559	−34.6967	
ZINC35566943	−39.4358	−34.6943	
ZINC18136415	−32.3323	−34.6688	
ZINC08681494	−39.7138	−34.6622	
ZINC08681494	−39.7138	−34.6622	
ZINC59763270	−39.3790	−34.6462	
ZINC13481900	−33.9841	−34.6226	Methylophiopogonanone A low binding free energy
ZINC15449241	−38.8430	−34.6108	
ZINC00338283	−32.6376	−34.5728	
ZINC03978825	−34.7173	−34.5700	
ZINC43574872	−36.8315	−34.5635	
ZINC04082157	−37.3933	−34.5380	
ZINC00002053	−34.3089	−34.5350	
ZINC00003225	−32.2989	−34.5349	
ZINC15115059	−34.8349	−34.5251	
ZINC38185595	−37.6074	−34.5159	
ZINC04245684	−38.6735	−34.4448	
ZINC04082214	−38.4403	−34.4253	
ZINC04082214	−38.4403	−34.4253	

ZINC35092275	-38.4474	-34.4102		
ZINC03973253	-42.8748	-34.3814		
ZINC14951118	-43.4620	-34.3803	Isoquercitrin	low binding free energy
ZINC12496173	-34.3030	-34.3748		
ZINC20470300	-32.7366	-34.3745		
ZINC12429157	-39.1079	-34.3472		
ZINC00039091	-33.0046	-34.3430		
ZINC21992198	-46.9545	-34.3429		
ZINC16954712	-34.7303	-34.3344		
ZINC31156436	-38.2748	-34.3289		
ZINC32296561	-34.7349	-34.2601		
ZINC38185597	-38.0047	-34.2474		
ZINC00047553	-31.7750	-34.2064		
ZINC71789804	-32.4266	-34.1755		
ZINC03894276	-38.8005	-34.1665		
ZINC14686820	-33.8307	-34.1533		
ZINC04097968	-35.3543	-34.1488		
ZINC03978829	-31.8759	-34.1316		
ZINC03875454	-33.5375	-34.1103		
ZINC21992836	-34.3490	-34.1079		
ZINC19795972	-33.0526	-34.0986		
ZINC01571579	-38.1190	-34.0651		
ZINC03978987	-32.2729	-34.0192		
ZINC03860825	-34.2525	-34.0169		
ZINC03860825	-34.2525	-34.0169		
ZINC38145807	-55.2925	-34.0029		
ZINC03643476	-36.1659	-33.9995		
ZINC18847037	-32.7490	-33.9582		
ZINC04919236	-32.0886	-33.9532		
ZINC08234298	-36.0986	-33.9516		
ZINC01608667	-32.4786	-33.9313		
ZINC04164657	-31.7307	-33.9310		
ZINC13424725	-40.4980	-33.9295		
ZINC04534390	-40.2191	-33.9290		
ZINC00899567	-32.2597	-33.9061		

ZINC01571581	-37.7105	-33.8949
ZINC28536379	-36.3112	-33.8703
ZINC03871358	-31.9403	-33.8459
ZINC04349814	-39.0494	-33.8323
ZINC03847505	-34.7527	-33.7799
ZINC01559620	-37.6271	-33.7626
ZINC04097773	-49.5871	-33.6800
ZINC00899614	-32.1060	-33.6682
ZINC04096248	-43.2625	-33.6553
ZINC31157290	-42.3287	-33.6551
ZINC82048449	-34.7567	-33.6512
ZINC27646582	-35.8688	-33.6170
ZINC03915684	-57.1123	-33.6112
ZINC03870412	-39.4789	-33.5622
ZINC00155886	-35.0747	-33.5374
ZINC05369365	-40.5061	-33.5266
ZINC40435394	-40.4319	-33.5141
ZINC06017816	-32.2542	-33.5127
ZINC14243543	-36.4512	-33.4658
ZINC03978049	-33.3694	-33.4597
ZINC03872147	-35.9326	-33.4471
ZINC03869521	-31.9460	-33.4056
ZINC33829907	-32.4766	-33.3958
ZINC43465475	-38.8207	-33.3783
ZINC13827726	-31.6748	-33.3602
ZINC21992895	-41.5103	-33.3143
ZINC14952515	-44.5319	-33.3131
ZINC03872688	-32.2815	-33.2883
ZINC95098781	-36.3468	-33.2808
ZINC08829476	-32.7946	-33.2715
ZINC71789441	-31.8995	-33.2687
ZINC33754036	-35.1162	-33.2326
ZINC13827713	-38.3195	-33.1339
ZINC14590441	-34.6570	-33.0829
ZINC04098344	-42.9622	-32.9751

ZINC00105082	-33.2719	-32.8618
ZINC06119283	-33.8908	-32.8611
ZINC03870339	-33.3088	-32.8599
ZINC19203131	-38.8093	-32.8584
ZINC00607997	-32.2262	-32.8089
ZINC04097775	-52.7938	-32.8045
ZINC15657693	-32.5553	-32.7899
ZINC70466441	-33.1028	-32.7878
ZINC39741117	-36.2523	-32.7730
ZINC03874929	-34.0830	-32.7374
ZINC14590442	-34.6723	-32.7322
ZINC05764767	-33.6343	-32.7252
ZINC02169830	-35.1477	-32.7042
ZINC35566941	-38.2650	-32.5667
ZINC71789466	-31.7234	-32.5358
ZINC13513730	-38.4715	-32.5278
ZINC00119978	-32.5888	-32.5023
ZINC03881790	-34.0956	-32.4500
ZINC85473389	-32.1681	-32.4434
ZINC44699918	-34.3348	-32.4202
ZINC06482592	-31.7250	-32.3889
ZINC87492934	-50.7523	-32.3657
ZINC04096945	-32.3397	-32.3435
ZINC38140515	-35.7391	-32.3251
ZINC04214775	-36.7642	-32.3077
ZINC00001419	-33.0583	-32.3056
ZINC33831946	-40.1070	-32.2785
ZINC04349040	-36.9547	-32.2115
ZINC03977785	-33.1271	-32.1219
ZINC34114798	-42.7612	-32.0948
ZINC00039092	-32.9524	-32.0827
ZINC71789668	-34.4929	-32.0190
ZINC14952519	-42.8276	-31.9510
ZINC01319796	-33.1557	-31.9222
ZINC86028695	-32.9531	-31.9117

ZINC33986547	-34.8344	-31.8946
ZINC28968107	-38.0863	-31.8671
ZINC00899870	-41.9631	-31.8391
ZINC13341184	-32.2734	-31.7944
ZINC85644480	-37.0841	-31.7889
ZINC31156206	-32.8450	-31.7779
ZINC33832060	-40.0533	-31.7481
ZINC85599241	-38.4524	-31.7434
ZINC95098890	-33.0498	-31.7122
ZINC00517261	-33.1435	-31.5115
ZINC04098322	-32.6339	-31.4973
ZINC56874786	-36.8597	-31.4520
ZINC03870336	-32.0400	-31.4485
ZINC44170832	-34.2905	-31.4314
ZINC05239485	-32.5683	-31.4230
ZINC03830179	-34.6351	-31.3333
ZINC13508092	-41.0856	-31.3158
ZINC04098735	-50.2378	-31.3019
ZINC03947454	-37.1587	-31.2573
ZINC28540146	-42.2359	-31.1876
ZINC04416340	-38.4246	-31.1782
ZINC83411013	-33.3237	-31.1187
ZINC86865006	-40.2725	-31.1148
ZINC13558220	-32.6269	-31.1119
ZINC06411540	-32.8587	-31.0732
ZINC05357395	-31.7079	-31.0427
ZINC39205909	-35.9342	-31.0190
ZINC13556374	-49.0908	-31.0040
ZINC21992890	-41.7279	-31.0011
ZINC05733652	-32.5382	-30.9986
ZINC08613125	-33.2006	-30.8834
ZINC21983181	-42.3635	-30.7791
ZINC04514131	-33.1383	-30.7025
ZINC01599733	-40.8986	-30.6794
ZINC04097650	-31.9780	-30.6721

ZINC39205910	−32.4264	−30.6632		
ZINC00000857	−33.2988	−30.6499		
ZINC14757120	−44.3604	−30.6064		
ZINC05234422	−32.8079	−30.5636		
ZINC86865004	−38.3447	−30.5443	Isoquercitrin	low binding free energy
ZINC05395839	−42.5684	−30.4325		
ZINC39741113	−33.1139	−30.3573		
ZINC00058116	−32.2292	−30.3507		
ZINC31156118	−39.1915	−30.2852		
ZINC86860237	−36.0033	−30.2725		
ZINC33980812	−32.8915	−30.2724	Quercetin	low binding free energy
ZINC33980812	−32.5169	−30.2724		
ZINC00895113	−33.4491	−30.2016		
ZINC00105076	−32.7922	−30.1818		
ZINC00119985	−32.0297	−30.1291		
ZINC33849103	−32.6294	−30.1200		
ZINC00518554	−33.4417	−30.1189		
ZINC31460607	−32.6403	−30.0530		
ZINC31519767	−33.4028	−29.9772		
ZINC36429694	−32.4984	−29.9692		
ZINC08214489	−42.1108	−29.9555		
ZINC06585367	−33.4268	−29.9394		
ZINC13451209	−49.5492	−29.9129		
ZINC71789806	−37.5564	−29.9029		
ZINC33949419	−43.3420	−29.8312		
ZINC28568062	−32.7363	−29.8193		
ZINC21992193	−42.4389	−29.7514		
ZINC71789457	−35.6207	−29.6720		
ZINC12360012	−32.4351	−29.5908		
ZINC95098790	−36.9013	−29.5864		
ZINC33949417	−42.2431	−29.5540		
ZINC04995158	−34.4695	−29.4996		
ZINC38545494	−38.1492	−29.3446		
ZINC04692013	−38.6691	−29.3213		
ZINC39205826	−32.1651	−29.3044		

ZINC19203128	−34.3405	−29.2539		
ZINC06524444	−32.5543	−29.1956		
ZINC06524444	−32.5543	−29.1956		
ZINC00338040	−32.9319	−29.0964		
ZINC00119983	−31.9797	−29.0052	Catechin	low binding free energy
ZINC00119983	−31.9797	−29.0052		
ZINC04823096	−32.9185	−28.9686		
ZINC21992201	−41.5920	−28.9192		
ZINC04102166	−39.4082	−28.9157		
ZINC03978047	−32.6860	−28.8769		
ZINC31158791	−36.2732	−28.8553		
ZINC05239480	−33.7730	−28.8174		
ZINC38349969	−36.0791	−28.8058		
ZINC02047673	−33.4796	−28.8029		
ZINC00105086	−32.3948	−28.7655		
ZINC33949416	−42.4094	−28.7374		
ZINC03870338	−32.7300	−28.6912		
ZINC03870338	−32.7300	−28.6912		
ZINC00896706	−32.2770	−28.6543		
ZINC03947453	−33.0182	−28.6114		
ZINC03978827	−33.4891	−28.5975		
ZINC01320090	−32.2604	−28.4705		
ZINC05395840	−43.3318	−28.4348		
ZINC18185774	−32.1441	−28.4071		
ZINC33833929	−33.1303	−28.4038		
ZINC08829484	−36.0534	−28.3595		
ZINC38605859	−34.9412	−28.3173		
ZINC04098704	−32.0103	−28.2814		
ZINC00039111	−32.2262	−28.2775		
ZINC06536276	−34.6494	−28.0767	Herbacetin	low binding free energy
ZINC03869685	−32.3822	−28.0481	Quercetin	low binding free energy
ZINC86864952	−38.7787	−28.0078		
ZINC36521966	−35.7068	−28.0002		
ZINC04082270	−34.9279	−27.9705		
ZINC98262385	−33.1429	−27.8879		

ZINC86860236	−34.8139	−27.8424		
ZINC04097774	−53.7176	−27.8296		
ZINC04097589	−34.8270	−27.8005		
ZINC00338043	−32.2837	−27.7503		
ZINC12153569	−40.8395	−27.7367		
ZINC03943903	−41.6551	−27.7341		
ZINC31154929	−39.1351	−27.6602		
ZINC03874317	−33.4020	−27.4145		
ZINC71789486	−41.8904	−27.2878		
ZINC21992187	−34.7458	−27.2766		
ZINC38141482	−69.4422	−27.1464		
ZINC38140516	−33.7890	−26.9248		
ZINC19795938	−36.0865	−26.8966		
ZINC86864954	−39.8959	−26.8770		
ZINC03978828	−33.3214	−26.8354		
ZINC08580514	−32.2801	−26.7065		
ZINC04523242	−31.6595	−26.6626		
ZINC95098891	−31.8729	−26.6319		
ZINC06567525	−39.0419	−26.3004		
ZINC05395841	−42.6070	−26.1926		
ZINC38140512	−36.7819	−26.0263		
ZINC04048240	−32.4572	−25.9618		
ZINC86864953	−41.7753	−25.9325		
ZINC21992196	−43.9119	−25.9169		
ZINC00135467	−34.1871	−25.3728		
ZINC86864951	−40.0808	−25.3346		
ZINC15206785	−45.1670	−25.1604		
ZINC08221271	−34.2622	−24.8983		
ZINC09213053	−44.2295	−24.7980		
ZINC34825997	−38.1781	−24.7378		
ZINC59206522	−43.6166	−24.7366		
ZINC13515661	−41.6435	−24.7279	Quercetin 3-glucuronide	HIA-, low binding free energy
ZINC21992916	−42.6140	−24.6637		
ZINC05842416	−31.9404	−24.4876		
ZINC03775158	−31.8835	−24.3625		

ZINC34825998	-38.7491	-24.3567
ZINC59736941	-40.8345	-23.5098
ZINC04273446	-33.9992	-22.8882
ZINC00135466	-34.3177	-22.8019
ZINC03846899	-37.2368	-21.5874
ZINC13512486	-37.3542	-21.3688
ZINC13549141	-40.4384	-21.3026
ZINC33830038	-43.9135	-21.2281
ZINC00056981	-31.6840	-20.9910
ZINC02138728	-40.4780	-19.1789
ZINC01562127	-32.1315	-19.0538
ZINC04228242	-43.6512	-18.8051
ZINC38141481	-67.8805	-18.5602
ZINC14690032	-47.0871	-18.1297
ZINC00155419	-32.9271	-17.9088
ZINC02389524	-40.4863	-17.6075
ZINC13527691	-43.4723	-16.3542
ZINC04097583	-44.1281	-14.9813
ZINC01529886	-31.7140	-8.2504
ZINC03871712	-31.8103	-6.8934
