# Supplementary figures and images for: In silico identification of potential inhibitors targeting Streptococcus mutans sortase A
Source: Int J Oral Sci. 2017 Mar 30;9(1):53–62. doi: 10.1038/ijos.2016.58 (PMC5379162; doi:10.1038/ijos.2016.58)

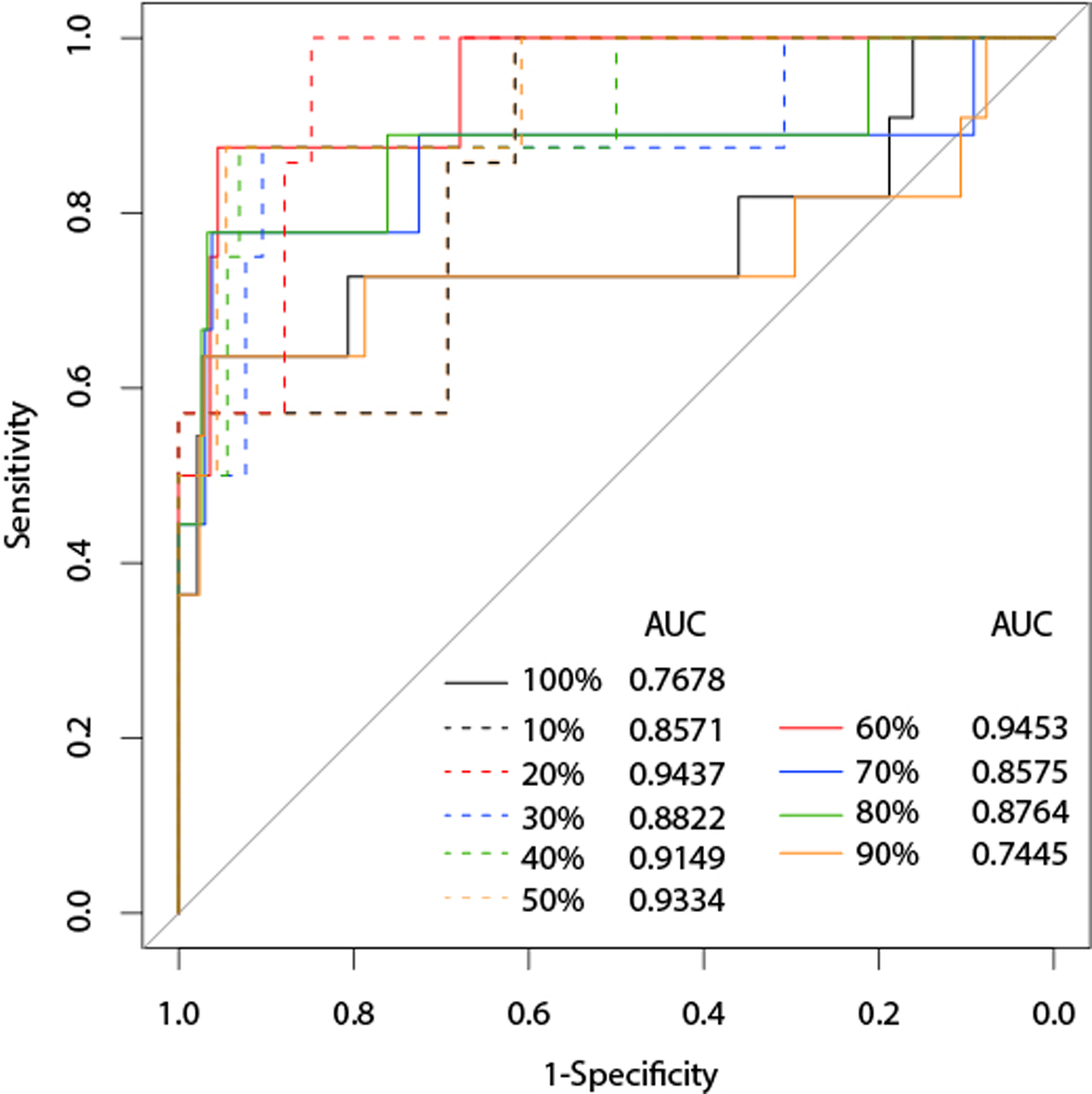

Supplement: Supplementary Figure S1 [file ijos201658x4.tif]

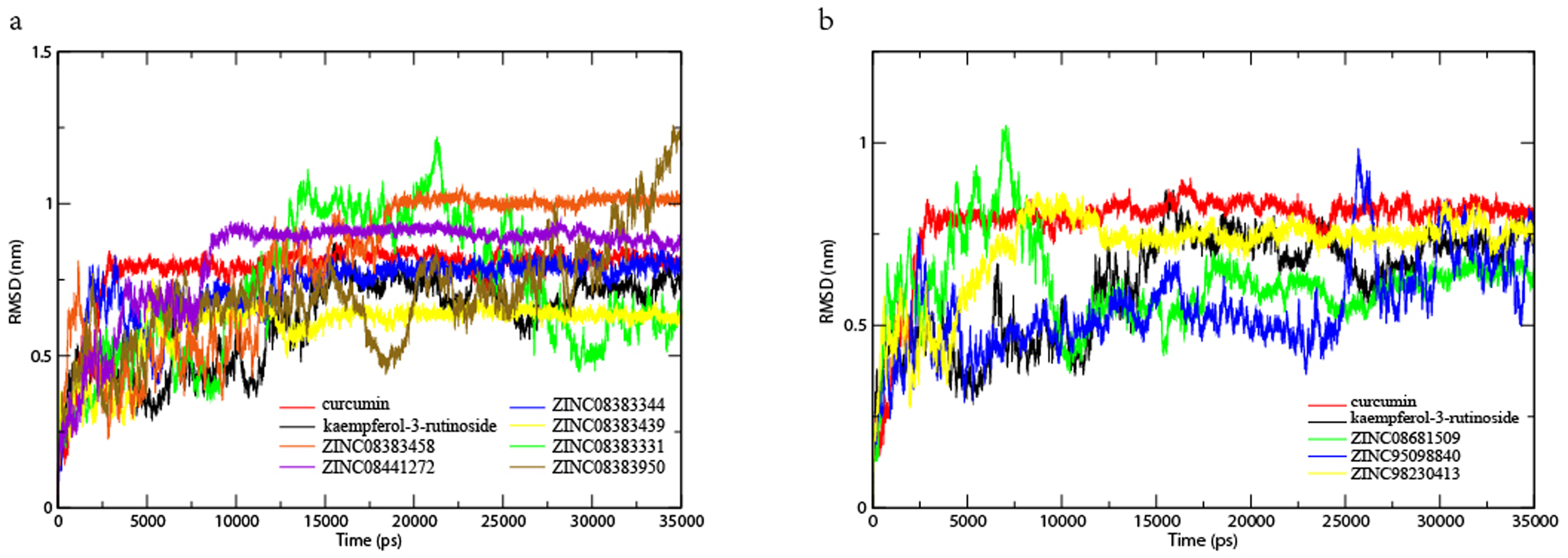

Supplement: Supplementary Figure S3 [file ijos201658x6.tif]
